# SEZ6-targeting antibody−drug conjugate ABBV-706 in advanced small cell lung cancer and solid tumors: a phase 1 trial

**DOI:** 10.1038/s41591-026-04452-0

**Published:** 2026-06-01

**Authors:** Lauren Averett Byers, Byoung Chul Cho, Alissa J. Cooper, Anne C. Chiang, Ji-Youn Han, Muhammad Furqan, Afshin Dowlati, Daniel Morgensztern, Kyriakos P. Papadopoulos, Noura J. Choudhury, María Vieito, Jair Bar, Joo-Hang Kim, Wallace Akerley, Tae Min Kim, Young-Chul Kim, Luis Paz-Ares, Myung-Ju Ahn, Hiroshi Yokouchi, Darius Meiman, Wijith Munasinghe, Oluwadamilola Ogunyankin, Nadine Jahchan, Song Wang, Cristiano Ferlini, Randy R. Robinson, Frederick J. Kohlhapp, Tammy Palenski, Guillermo Rivell, Pooja Hingorani, Sreenivasa Chandana

**Affiliations:** 1 The University of Texas MD Anderson Cancer Center, Houston, TX USA; 2 Division of Medical Oncology, Yonsei University College of Medicine, Yonsei Cancer Center, Seoul, Republic of Korea; 3 Department of Thoracic Oncology, Memorial Sloan Kettering Cancer Center, New York, NY USA; 4 Yale School of Medicine, New Haven, CT USA; 5 National Cancer Center, Gyeonggi-do, Republic of Korea; 6 University of Iowa Holden Comprehensive Cancer Center, Iowa City, IA USA; 7 University Hospitals Seidman Cancer Center and Case Western Reserve University, Cleveland, OH USA; 8 Washington University School of Medicine, St. Louis, MO USA; 9 START San Antonio, San Antonio, TX USA; 10 Department of Medicine, University of Chicago, Chicago, IL USA; 11 Department of Medical Oncology, Vall d’Hebron Barcelona Hospital Campus, Barcelona, Spain; 12 Vall d’Hebron Institute of Oncology, Barcelona, Spain; 13 Jusidman Cancer Center, Sheba Medical Center, Ramat Gan, Israel; 14 The Gray Faculty of Medical and Health Sciences, Tel Aviv University, Tel Aviv, Israel; 15 Department of Internal Medicine, CHA Bundang Medical Center, CHA University, Seongnam, Republic of Korea; 16 Division of Medical Oncology, Huntsman Cancer Institute, University of Utah, Salt Lake City, UT USA; 17 Department of Internal Medicine, Seoul National University Hospital, Seoul National University College of Medicine, Seoul, Republic of Korea; 18 Department of Internal Medicine, Chonnam National University Medical School, and Chonnam National University Hwasun Hospital, Jeonnam, Republic of Korea; 19 Hospital Universitario 12 de Octubre and Universidad Complutense, Madrid, Spain; 20 Section of Hematology-Oncology, Department of Medicine, Hanyang University Medical Center, Hanyang University, Seoul, Republic of Korea; 21 National Hospital Organization Hokkaido Cancer Center, Sapporo, Japan; 22 AbbVie Inc., North Chicago, IL USA; 23 START Midwest, Grand Rapids, MI USA

**Keywords:** Small-cell lung cancer, Cancer therapy

## Abstract

Seizure-related homolog 6 (SEZ6) is expressed in small cell lung cancer (SCLC) and neuroendocrine neoplasms. In an open label, phase 1 trial, ABBV-706, an antibody−drug conjugate with a SEZ6-directed antibody linked to topoisomerase-1 inhibitor (Top1i), was administered intravenously every 3 weeks (Q3W) to 288 patients with advanced solid tumors; 240 received monotherapy, including 124 with relapsed/refractory (R/R) SCLC. Primary objectives of dose escalation (part 1, advanced solid tumors), dose optimization and expansion (part 2, R/R SCLC only) and dose expansion (part 4, central nervous system tumors and high-grade neuroendocrine neoplasms only) were to evaluate the safety, tolerability, pharmacokinetics (PK), immunogenicity and antitumor activity of ABBV-706 monotherapy and, from parts 1 and 2, to determine the recommended phase 2 dose (RP2D) of ABBV-706 in R/R SCLC. In the monotherapy cohort (*N* = 240), the most common treatment-related adverse events (TRAEs) at any grade were anemia (61%) and fatigue (38%). Grade 3 or higher TRAEs occurred in 61% of patients and were dose dependent (39% at 1.8 mg kg^−1^ and 70% at 2.5 mg kg^−1^). In the R/R SCLC monotherapy cohort (*n* = 124), any-grade and grade 3 or higher TRAEs occurred in 93% and 61% of patients, respectively. ABBV-706 demonstrated promising preliminary efficacy in patients with R/R SCLC, with an objective response rate (ORR) of 52% (65/124). In patients with R/R SCLC receiving monotherapy in dose optimization and expansion part 2, ORR was similar between 1.8 mg kg^−1^ and 2.5 mg kg^−1^ doses (56% (23/41) and 59% (23/39), respectively), with a duration of response that was highest at the 1.8 mg kg^−1^ dose and with most patients achieving rapid and durable tumor reduction. Although exploratory, long-term efficacy measures were an important consideration in the RP2D determination, and in R/R SCLC monotherapy, overall survival (OS) was highest at the 1.8 mg kg^−1^ dose, with a median OS of 12.4 months. Based on the totality of available data, including, but not limited to, safety, preliminary efficacy measures and PK, 1.8 mg kg^−1^ Q3W was confirmed as the optimal RP2D for patients with R/R SCLC. ClinicalTrials.gov: NCT05599984.

## Abstract (web summary)

As presented at the 2026 ASCO Annual Meeting, in a phase 1 trial, treatment of patients with the seizure-related homolog protein 6 (SEZ6)-targeting antibody−drug conjugate ABBV-706 demonstrated a manageable safety profile and showed preliminary clinical efficacy in patients with resistant or refractory small cell lung cancer.

## Main

Small cell lung cancer (SCLC) accounts for about 11% of all lung cancers^1^ and is an aggressive neuroendocrine carcinoma characterized by rapid proliferation and early metastatic spread^2^. Most patients (69%) are diagnosed with extensive-stage disease^1^. Although most patients with SCLC initially respond to chemotherapy (with or without immunotherapy), relapse after first-line therapy is common, with nearly 50% of patients experiencing disease progression within 6 months and only approximately 10% remaining disease free after 2 years^3,4^. For patients with extensive-stage SCLC, the 5-year overall survival (OS) rate is only approximately 12%^4^. In the IMpower133 and CASPIAN trials, the addition of immune checkpoint inhibitors to platinum-etoposide chemotherapy modestly improved median OS to 12.3 months and 13.0 months, respectively, compared to 10.3 months with chemotherapy alone^5,6^. However, only approximately 15–23% of patients who responded to treatment had ongoing responses at 12 months, highlighting the limited durability of benefit^5,6^. These outcomes reinforce the unmet need for novel therapies with improved efficacy and tolerability for patients with extensive-stage SCLC.

SEZ6 is a type I transmembrane protein and a neural lineage marker involved in neuronal development and synaptic organization^7^. Although its role in oncogenesis has not been fully elucidated, transcriptomic and immunohistochemical profiling studies have shown that SEZ6 is selectively and highly expressed in SCLC and high-grade neuroendocrine carcinoma, with minimal expression in non-neuroendocrine malignancies and normal adult tissues^8^. The first-in-human study of ABBV-011, a first-generation SEZ6-targeting antibody−drug conjugate (ADC) conjugated to calicheamicin via a non-cleavable linker with a drug:antibody ratio of 2, demonstrated SEZ6 expression in more than 80% of SCLC tumor samples and preliminary clinical activity in patients with relapsed/refractory (R/R) SCLC^9^, supporting the therapeutic actionability of SEZ6 in this patient population.

ABBV-706 is a first-in-class ADC comprising a potent Top1i attached via a stable linker to a SEZ6-directed monoclonal antibody with a drug:antibody ratio of 6. Once internalized, the linker is cleaved in the lysosomal environment of the tumor cells, which releases Top1i and induces DNA damage and apoptosis. Preclinical studies in patient-derived xenograft models of SEZ6-positive SCLC demonstrated superior activity of ABBV-706 compared to ABBV-011 and standard-of-care chemotherapies^10^. Preliminary results from the ongoing first-in-human phase 1 trial (NCT05599984) to evaluate the safety, tolerability, pharmacokinetics (PK), immunogenicity and antitumor activity of ABBV-706 in patients with R/R solid tumors (defined as those patients whose disease had progressed on or after treatment with standard of care), including patients with R/R SCLC, demonstrated that ABBV-706 monotherapy had a manageable safety profile and promising antitumor activity, with a confirmed ORR of 43.8% (60.9% in patients with R/R SCLC)^11^. Here we present updated safety, tolerability, PK and immunogenicity results for all patients treated with ABBV-706 monotherapy, and efficacy and exploratory biomarker results for patients with R/R SCLC treated with ABBV-706 monotherapy, with an emphasis on those patients included in the randomized dose-optimization cohorts.

## Results

### Study design and patients

**Fig. 1:**
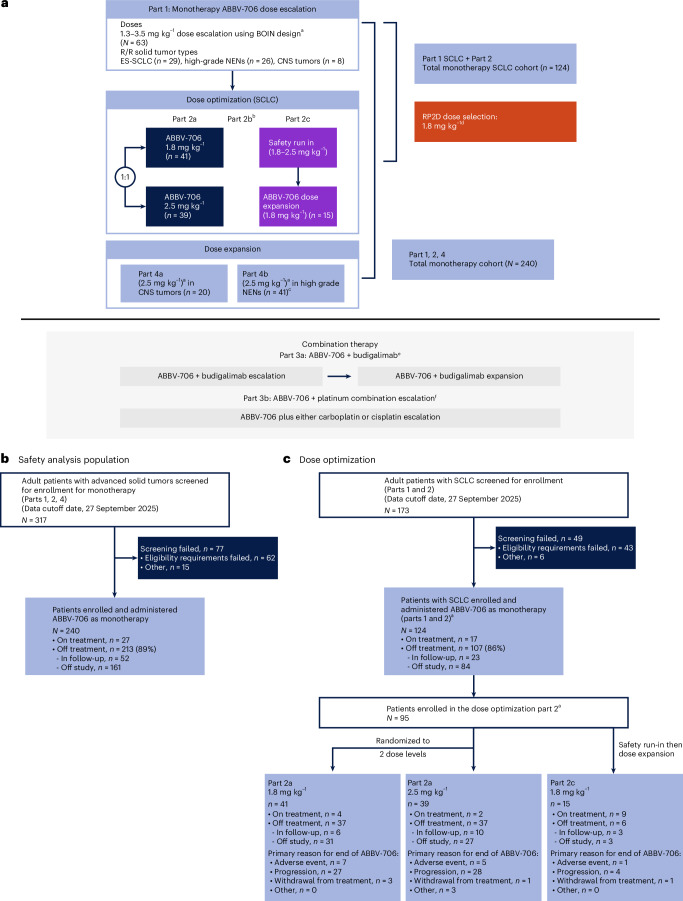
Study design. **a**, Study schema. CONSORT diagram disposition of all patients who received monotherapy comprising the safety analysis population (**b**) and of those in the dose-optimization cohorts who were included in the primary efficacy and RP2D analysis (**c**). This phase 1, open-label, dose-escalation and dose-expansion study of ABBV-706 in patients with advanced solid tumors with potential SEZ6 expression comprised four parts: part 1 was dose escalation of ABBV-706 monotherapy and included all advanced solid tumors; part 2 (a, b and c) was dose optimization/expansion of ABBV-706 monotherapy in R/R SCLC; and part 4 was dose expansion of ABBV-706 monotherapy in R/R CNS tumors (4a) and high-grade NENs (4b). Data from part 3 (ABBV-706 combination therapy) are not reported. ^a^IV Q3W in 21-day cycles until disease progression or unacceptable toxicity. ^b^Part 2b was not pursued. ^c^One patient in this cohort was ineligible. ^d^The totality of efficacy, safety and PK data from part 1 and part 2 confirmed 1.8 mg kg^−1^ as the optimal dose for patients with R/R SCLC. ^e^*n* = 30 patients were screened and enrolled in the ABBV-706 plus budigalimab combination cohorts, which are not reported here. ^f^ Part 3b was halted after enrollment of 18 patients; data from this cohort are not reported here. BOIN, Bayesian optimal interval; ES, extensive stage; IV, intravenous.

This was a first-in-human, open-label, phase 1 trial of ABBV-706 in adult patients with advanced solid tumors. ABBV-706 was administered intravenously every three weeks (Q3W) to eligible patients. The study included the following parts: part 1 included a dose-escalation cohort of patients with advanced solid tumors; part 2a was a dose optimization for patients with SCLC who progressed on or after post-platinum chemoimmunotherapy and enrolled two dose levels (1.8 mg kg^−1^ and 2.5 mg kg^−1^); part 2c included a safety lead-in and expansion in China for R/R SCLC; part 3a was a combination cohort with programmed death-ligand 1 (PD-L1) inhibitor budigalimab for R/R SCLC and high-grade neuroendocrine tumors/neuroendocrine carcinoma (NET/NEC); part 3b was an escalation and expansion in combination with platinum chemotherapy for R/R SCLC or high-grade NET/NEC; and part 4 was a dose expansion in patients with high-grade NET/NEC. The primary objectives of parts 1, 2 and 4, reported here, were to evaluate the safety, tolerability, PK, immunogenicity and efficacy of ABBV-706 monotherapy and, from parts 1 and 2, to determine the RP2D of ABBV-706 in R/R SCLC. Exploratory objectives included biomarker analysis. The first patient enrollment date was 28 December 2022 (part 1), and the last patient enrollment date was 29 August 2025 (part 2c). Approximately 317 patients were screened for monotherapy (parts 1, 2 and 4), reported here, and 48 patients were screened for part 3, which is not reported here (30 for budigalimab plus ABBV-706 combination (part 3a) and 18 for ABBV-706 plus platinum combination escalation (part 3b)) (Fig. 1).

As of 27 September 2025 (data cutoff), 288 patients with solid tumors were enrolled, which included 240 patients in monotherapy (parts 1, 2a, 2c and 4) for whom primary safety and PK data are presented. Of these 240 patients, ABBV-706 was administered as monotherapy to 124 patients with R/R SCLC (parts 1, 2a and 2c), 80 of whom were enrolled in the randomized dose-optimization part 2a (1.8 mg kg^−1^ cohort, *n* = 41; 2.5 mg kg^−1^ cohort, *n* = 39). The RP2D was derived from the totality of safety and efficacy data presented for parts 1 and 2, with an emphasis on efficacy in the randomized dose-optimization part 2a cohorts (Fig. 1).

To provide context for the safety data presented in all patients receiving at least one dose of ABBV-706 monotherapy (*N* = 240), baseline disease characteristics for this safety analysis population are presented in brief in Table 1a. In addition to the 124 patients with R/R SCLC who received monotherapy, the remaining patients who received monotherapy from parts 1 or 4 had high-grade neuroendocrine neoplasms (NENs) or central nervous system (CNS) advanced solid tumors.

**Table 1:** Demographics and baseline disease characteristics

a. All monotherapy	1.3 mg kg^−1^ *n* = 10	1.8 mg kg^−1^ *n* = 71	2.5 mg kg^−1^ *n* = 139	3.0 mg kg^−1^ *n* = 15	3.5 mg kg^−1^ *n* = 5	Total *N* = 240
Median age, years (range)	65 (52−76)	62 (35−85)	61 (18−86)	59 (32−78)	71 (51−73)	62 (18−86)
Sex, *n* (%)						
Male	3 (30)	49 (69)	81 (58)	10 (67)	1 (20)	144 (60)
Female	7 (70)	22 (31)	58 (42)	5 (33)	4 (80)	96 (40)
Race, *n* (%)						
Asian	0	33 (47)	31 (22)	4 (27)	0	68 (28)
Black/African American	1 (10)	0	5 (4)	2 (13)	1 (20)	9 (4)
White	7 (70)	36 (51)	98 (71)	9 (60)	4 (80)	154 (64)
Not reported	2 (20)	2 (3)	5 (4)	0	0	9 (4)
Tobacco use, *n* (%)						
Current	1 (10)	6 (9)	19 (14)	3 (20)	1 (20)	30 (13)
Former	9 (90)	57 (80)	59 (42)	5 (33)	2 (40)	132 (55)
Never	0	8 (11)	61 (44)	7 (47)	2 (40)	78 (33)
ECOG PS, *n* (%)						
0	3 (30)	12 (17)	37 (27)	5 (33)	0	57 (24)
1	7 (70)	59 (83)	102 (73)	10 (67)	5 (100)	183 (76)
Disease type, *n* (%)						
SCLC	8 (80)	63 (89)	44 (32)	7 (47)	2 (40)	124 (52)
High-grade NENs or CNS tumors	2 (20)	8 (11)	95 (68)	8 (53)	3 (60)	116 (48)

b. R/R SCLC monotherapy	Part 2a 1.8 mg kg^−1^ *n* = 41	Part 2a 1.8 mg kg^−1^ 2L *n* = 17	Part 2a 2.5 mg kg^−1^ *n* = 39	Part 1 + 2a + 2c (*n* = 124)
Median age, years (range)	64 (48−85)	62 (50−68)	66 (51−81)	64 (35−85)
Sex, *n* (%)				
Male	25 (61)	11 (65)	19 (49)	73 (59)
Female	16 (39)	6 (35)	20 (51)	51 (41)
Race, *n* (%)				
Asian	16 (39)	6 (39)	12 (31)	49 (40)
Black/African American	0	0	1 (3)	3 (2)
White	24 (59)	10 (59)	25 (64)	69 (56)
Not reported	1 (2)	1 (6)	1 (3)	3 (2)
Tobacco use, *n* (%)				
Current	4 (10)	2 (12)	8 (21)	21 (17)
Former	36 (88)	15 (89)	29 (74)	95 (77)
Never	1 (2)	0	2 (5)	8 (7)
ECOG PS, *n* (%)				
0	9 (22)	5 (29)	7 (18)	24 (19)
1	32 (78)	12 (71)	32 (82)	100 (81)
Stage IV at study entry, *n* (%)	35 (85)	15 (88)	36 (92)	111 (90)
Brain metastasis at study entry, *n* (%)	16 (39)	7 (41)	12 (31)	42 (34)
Whole-brain radiotherapy as most recent radiotherapy	7 (17)	4 (24)	7 (18)	19 (15)
Prior lines of therapy				
Median (range)	2 (1−6)	1 (1−1)	2 (1−6)	2 (1−6)
1, *n* (%)	17 (42)	17 (100)	14 (36)	43 (35)
≥2, *n* (%)	24 (59)	0	25 (64)	81 (65)
Select prior lines of therapy				
Top1i	12 (29)	1 (6)	11 (28)	35 (28)
Tarlatamab	3 (7)	0	0	8 (7)
Anti-PD-L1	33 (81)	13 (77)	31 (80)	103 (83)
Lurbinectedin	10 (24)	0	7 (18)	25 (20)
Platinum	41 (100)	17 (100)	39 (100)	124 (100)
Top2i	40 (98)	16 (94)	37 (95)	121 (98)
CTFI, *n* (%)				
≥90 days	12 (29)	4 (24)	18 (46)	44 (36)
<90 days	25 (61)	13 (77)	17 (44)	66 (53)
<30 days	11 (27)	4 (24)	8 (21)	29 (23)
Missing	4 (10)	0	4 (10)	14 (11)

**a**,**b**, Demographics and baseline disease characteristics for all monotherapy patients (**a**) and all patients in the R/R SCLC cohort who received monotherapy, including patients enrolled in the part 2a dose-optimization cohorts (**b**).

Baseline disease characteristics for all patients with R/R SCLC treated with monotherapy (*n* = 124, hereafter defined as ‘all R/R SCLC cohorts’); all patients with R/R SCLC in part 2a treated at 1.8 mg kg^−1^ Q3W (*n* = 41, hereafter defined as ‘1.8 mg kg^−1^’); a subset of part 2a patients with R/R SCLC treated at 1.8 mg kg^−1^ Q3W who received treatment in the second-line (2L) (*n* = 17, hereafter defined as ‘1.8 mg kg^−1^ 2L’); and part 2a patients treated at 2.5 mg kg^−1^ Q3W (*n* = 39, hereafter defined as ‘2.5 mg kg^−1^’) are presented in Table 1b. Across all R/R SCLC cohorts, median age was 64 (range, 35−85) years; 59% were male; 81% had Eastern Cooperative Oncology Group performance status (ECOG PS) of 1; and 34% had brain metastasis at study entry. Among the 1.8 mg kg^−1^ and 2.5 mg kg^−1^ cohorts, 39% and 31% had brain metastasis at study entry, respectively. Median number of prior lines of therapies was two (range, 1−6) for both the 1.8 mg kg^−1^ and 2.5 mg kg^−1^ cohorts. For the 1.8 mg kg^−1^ and 2.5 mg kg^−1^ cohorts, 59% and 64% of patients had two or more prior lines; 61% and 44% of patients had platinum-resistant disease (<90 days chemotherapy-free interval (CTFI)); 27% and 21% of patients were platinum refractory (<30 days CTFI); and 29% and 28% of patients had previously received treatment with a Top1i, respectively.

Median follow-up was 16.9 months at the data cutoff date across the safety analysis population (*N* = 240). Median follow-up was 16.9 months at data cutoff across all R/R SCLC cohorts (*n* = 124) and 17.5 months, 17.5 months and 16.8 months for the 1.8 mg kg^−1^, 1.8 mg kg^−1^ 2L and 2.5 mg kg^−1^ cohorts, respectively.

### Safety

In total, 222 out of 240 patients (93%) in the safety analysis population who received ABBV-706 monotherapy had one or more treatment-related adverse events (TRAEs), of whom 147 (61%) had a grade 3 or higher event, which were primarily hematological (Table 2a and Extended Data Table 1a). In determining the dose during the part 1 dose escalation, two dose-limiting toxicities occurred, including one event of grade 4 leukopenia and neutropenia lasting more than 7 days at the 3.0 mg kg^−1^ dose and one event of grade 4 thrombocytopenia at the 3.5 mg kg^−1^ dose, thereby establishing the maximum tolerated dose as 3.0 mg kg^−1^.

**Table 2:** Summary of TRAEs

a. All monotherapy												
	1.3 mg kg^−1^ *n* = 10		1.8 mg kg^−1^ *n* = 71		2.5 mg kg^−1^ *n* = 139		3.0 mg kg^−1^ *n* = 15		3.5 mg kg^−1^ *n* = 5		Total *N* = 240	
	Any grade	Grade ≥3	Any grade	Grade ≥3	Any grade	Grade ≥3	Any grade	Grade ≥3	Any grade	Grade ≥3	Any grade	Grade ≥3
Any TRAE, *n* (%)	8 (80)	4 (40)	63 (89)	28 (40)	131 (94)	97 (70)	15 (100)	13 (87)	5 (100)	5 (100)	222 (93)	147 (61)
TRAE leading to ABBV-706 discontinuation, *n* (%)	1 (10)		5 (7)		9 (7)		1 (7)		0		16 (7)	
TRAE leading to dose interruption, *n* (%)	1 (10)		17 (24)		71 (51)		9 (60)		4 (80)		102 (43)	
TRAE leading to dose reduction, *n* (%)	1 (10)		11 (16)		46 (33)		11 (73)		4 (80)		73 (30)	
TRAE leading to death, *n* (%)	0		3 (4)		0		0		0		3 (1)	
SAE related to ABBV-706, *n* (%)	1 (10)		9 (13)		18 (13)		1 (7)		1 (20)		30 (13)	
Hematologic TRAEs occurring with an incidence of ≥20% (with combined terms)												
Anemia	1 (10)	1 (10)	36 (51)	22 (31)	93 (67)	73 (53)	12 (80)	7 (47)	5 (100)	5 (100)	147 (61)	108 (45)
Red blood cell count decreased	0	0	1 (1)	0	0	0	0	0	0	0	1 (1)	0
Neutropenia	0	0	2 (2.8)	1 (1)	21 (15)	13 (9)	0	0	0	0	23 (10)	14 (6)
Neutrophil count decreased	0	0	20 (28)	10 (14)	54 (39)	44 (32)	11 (73)	11 (73)	5 (100)	4 (80)	90 (38)	69 (29)
Thrombocytopenia	0	0	2 (2.8)	0	16 (12)	4 (3)	1 (7)	1 (7)	0	0	19 (8)	5 (2)
Platelet count decreased	1 (10)	1 (1)	14 (20)	8 (11)	41 (30)	27 (19)	9 (60)	6 (40)	4 (80)	3 (60)	69 (29)	45 (19)
Leukopenia	0	0	1 (1)	0	2 (1)	0	0	0	0	0	3 (1)	0
White blood cell decreased	1 (10)	1 (10)	15 (21)	4 (6)	34 (25)	29 (21)	9 (60)	7 (47)	4 (80)	4 (80)	63 (26)	45 (19)
Non-hematologic TRAEs occurring with an incidence of ≥20%												
Fatigue	5 (50)	1 (10)	26 (37)	1 (1)	47 (34)	7 (5)	10 (67)	0	3 (60)	0	91 (38)	9 (4)
Nausea	5 (50)	0	27 (38)	0	51 (37)	2 (1)	3 (20)	0	0	0	86 (36)	2 (1)
Decreased appetite	4 (40)	0	12 (17)	1 (1)	32 (23)	2 (1)	1 (7)	0	1 (20)	0	50 (21)	3 (1)

b. R/R SCLC monotherapy						
	1.8 mg kg^−1^ (*n* = 41)		2.5 mg kg^−1^ (*n* = 39)		Part 1 + 2a + 2c (*n* = 124)	
Any TRAE, *n* (%)	36 (88)		38 (97)		115 (93)	
Grade ≥3	22 (54)		30 (77)		75 (61)	
TRAE leading to ABBV-706 discontinuation, *n* (%)	5 (12)		3 (8)		10 (8)	
TRAE leading to dose interruption, *n* (%)	13 (32)		25 (64)		55 (44)	
TRAE leading to dose reduction, *n* (%)	8 (20)		13 (33)		34 (27)	
TRAE leading to death, *n* (%)	3 (7)		0		3 (2)	
SAEs, *n* (%)	7 (17)		6 (15)		18 (15)	
Hematologic TRAEs occurring with an incidence of ≥20% in full monotherapy cohort						
	Any grade	Grade ≥3	Any grade	Grade ≥3	Any grade	Grade ≥3
Anemia	22 (54)	17 (42)	29 (74)	25 (64)	79 (64)	57 (46)
Red blood cell decreased	0	0	0	0	1 (1)	0
Neutropenia	1 (2)	0	3 (8)	1 (3)	5 (4)	2 (2)
Neutrophil count decreased	13 (32)	8 (20)	17 (44)	12 (31)	49 (40)	32 (26)
Thrombocytopenia	1 (2)	0	5 (13)	2 (5)	7 (6)	2 (2)
Platelet count decreased	9 (22)	6 (15)	15 (39)	9 (23)	41 (33)	27 (22)
Leukopenia	0	0	1 (3)	0	2 (2)	0
White blood cell decreased	8 (20)	3 (7)	9 (23)	8 (21)	31 (25)	17 (14)
Non-hematologic TRAEs occurring with an incidence of ≥20% in full monotherapy cohort						
	Any grade	Grade ≥3	Any grade	Grade ≥3	Any grade	Grade ≥3
Fatigue	14 (34)	1 (2)	14 (36)	2 (5)	47 (38)	4 (3)
Nausea	15 (37)	0	10 (26)	1 (3)	40 (32)	1 (1)
Decreased appetite	8 (20)	1 (2)	16 (41)	1 (3)	34 (27)	2 (2)

**a**,**b**, TRAEs in all monotherapy patients (**a**) and patients with R/R SCLC, including those in the 1.8 mg kg^−1^ and 2.5 mg kg^−1^ dose-optimization cohorts (**b**).

The frequency of grade 3 or higher TRAEs was dose dependent, with 40% at 1.3 mg kg^−1^, 40% at 1.8 mg kg^−1^, 70% at 2.5 mg kg^−1^, 87% at 3.0 mg kg^−1^ and 100% at 3.5 mg kg^−1^. Cytopenias were the most common high-grade TRAEs and were dose dependent. Treatment-related serious adverse events (SAEs) occurred in 30 (13%) patients, the most common events being platelet count decrease in six (3%) patients, pneumonitis in five (2%) patients and febrile neutropenia in four (2%) patients. In total, 111 (46%) patients received granulocyte colony-stimulating factor in the prophylactic or therapeutic setting, allowed as secondary prevention. Among 147 patients with any-grade treatment-related anemia, 105 received packed red blood cell (pRBC) blood transfusion, and 14 of 86 patients with treatment-related thrombocytopenia/platelet count increase received a thrombopoietin receptor agonist. Treatment-related gastrointestinal toxicities were mainly grade 1 or 2.

Pneumonitis/interstitial lung disease (ILD) is a known risk associated with ADC treatment. The incidence of drug-related ILD was determined by an independent adjudication committee. Of the 240 patients receiving monotherapy, 10 (4%) patients had adjudicated pneumonitis/ILD and four (2%) had grade 3 or higher pneumonitis/ILD events. Median time to onset of any-grade pneumonitis/ILD was 96 days (range, 37−291).

In all patients who received monotherapy (*N* = 240), TRAEs leading to discontinuation occurred in 16 (6.7%) patients, primarily due to pneumonitis (*n* = 9 (4%)) and platelet count decrease (2 (1%)). Treatment-related interruptions and reductions were more frequent at 2.5 mg kg^−1^ (51% and 33%) than at 1.8 mg kg^−1^ (24% and 16%). The most common TRAE of all patients who received monotherapy leading to interruption and reduction was anemia in 38 (16%) patients and 33 (14%) patients, respectively. Three events (3%) leading to death were considered related to ABBV-706 (platelet count decreased, *n* = 1; pneumonitis, *n* = 2).

In addition to the full monotherapy population, safety is also reported among the dose-optimization cohorts to facilitate determination of the RP2D. For patients with R/R SCLC in the 1.8 mg kg^−1^ and 2.5 mg kg^−1^ cohorts, any-grade TRAEs occurred in 88% and 97%, respectively; the most common were anemia (54% versus 74%) and fatigue (34% versus 36%). Grade 3 or higher TRAEs occurred in 54% and 77% of patients; anemia (42% versus 64%) and decreased neutrophil counts (20% versus 31%) were the most common (Table 2b and Extended Data Table 1b).

The median relative dose intensity decreased with increasing dose from 99.7% (range, 60−106) at the 1.3 mg kg^−1^ dose to 64.9% (range, 50−99) at the 3.5 mg kg^−1^ dose in the full monotherapy safety analysis population (*N* = 240). In patients with R/R SCLC from part 2a, the median relative dose intensity was 98.9% (range, 59−105) in the 1.8 mg kg^−1^ dose cohort and 85.3% (range, 44−106) in the 2.5 mg kg^−1^ cohort; the median treatment duration was 6.2 months and 5.5 months, respectively.

As ABBV-706 is unlikely to cause QT interval prolongation, full data on this additional prespecified exploratory endpoint are not reported.

### Clinical PK and immunogenicity

**Extended Data Fig. 1:**
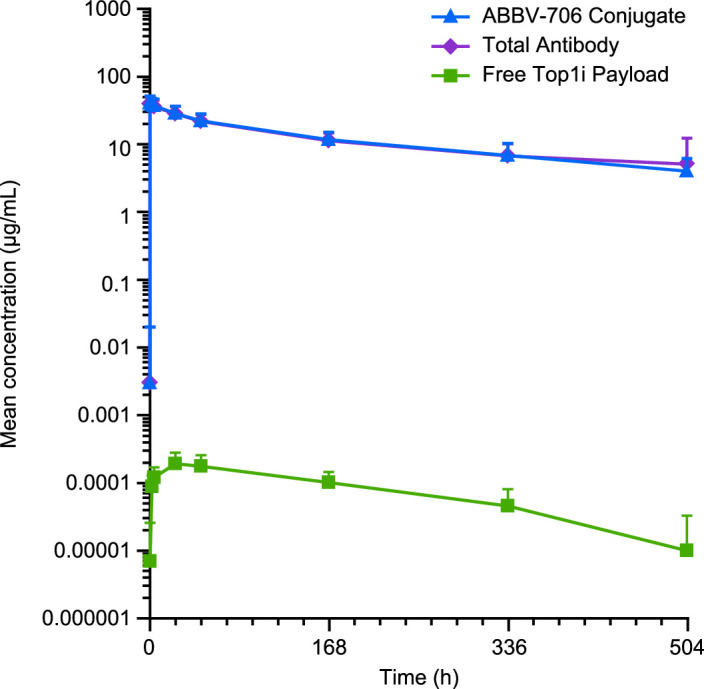
Mean (SD) concentration-time profiles of ABBV-706 conjugate, total antibody, and free Top1i payload at ABBV-706 1.8 mg kg^−1^ Q3W in cycle 1 as monotherapy. Q3W, every 3 weeks; SD, standard deviation; Top1i, topoisomerase 1 inhibitor.

Preliminary PK parameters for ABBV-706 conjugate, total antibody and unconjugated (free) Top1i payload after monotherapy administration for the PK analysis population (*N* = 240) are summarized in Extended Data Table 2. Linear dose-dependent PK with no apparent target-mediated drug disposition was observed across 1.3−3.5 mg kg^−1^ doses. After the first dose, the terminal phase elimination half-life was approximately 7 days for the ABBV-706 conjugate and total antibody and 10 days for the free Top1i payload across the studied doses. Preliminary analysis indicates minimal (accumulation ratio <1.5-fold) increase in exposure for ABBV-706 conjugate, total antibody and free Top1i payload in cycle 3 versus cycle 1. ABBV-706 conjugate and total antibody exposures are similar with essentially overlapping PK profiles (Extended Data Fig. 1), indicating a stable drug linker. Preliminary data indicate a low immunogenicity potential as monotherapy, with 8.3% (20/240) of patients testing positive for ABBV-706 ADA across all available timepoints. ADA incidence does not appear to impact the ABBV-706 conjugate PK.

### Efficacy

Efficacy parameters for all Response Evaluation Criteria in Solid Tumors (RECIST)-evaluable patients with R/R SCLC (*n* = 124) who received ABBV-706 monotherapy are summarized in Supplementary Table 1. Across all R/R SCLC cohorts (*n* = 124), ORR per RECIST version 1.1 by investigator was 52%. Median duration of response (DOR) was 5.3 months (95% confidence interval (CI): 4.1−6.7). With a median follow-up of 16.9 months, median progression-free survival (PFS) was 5.4 months (95% CI: 4.4−5.7) (Supplementary Table 1). Median OS was 11.3 months (95% CI: 9.1−14.8), and landmark OS estimate at 15 months was 40% (95% CI: 30−49).

**Fig. 2:**
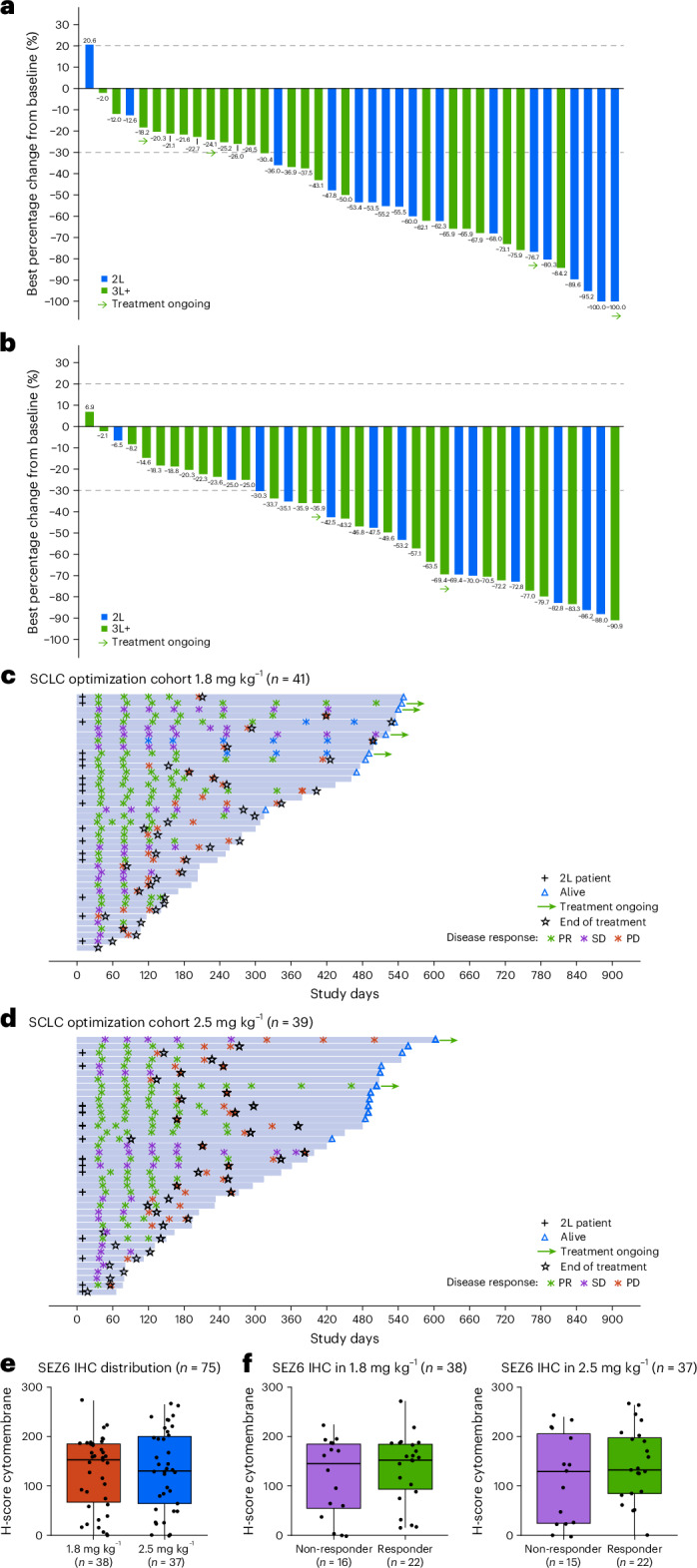
Efficacy of ABBV-706 and SEZ6 expression in R/R SCLC, including the 1.8 mg kg^−1^ and 2.5 mg kg^−1^ dose-optimization cohorts. **a**, Best percentage change in target lesion size from baseline in the 1.8 mg kg^−1^ cohort (*n* = 41). **b**, Best percentage change in target lesion size from baseline in the 2.5 mg kg^−1^ cohort (*n* = 39). **c**, Response over time while on study in the 1.8 mg kg^−1^ cohort (*n* = 41). **d**, Response over time while on study in the 2.5 mg kg^−1^ cohort (*n* = 39). Distribution of SEZ6 expression between arms (**e**) and among non-responders and responders (**f**) in the 1.8 mg kg^−1^ and 2.5 mg kg^−1^ dose cohorts. Evaluable SEZ6 expression data were available for 38 of 47 patients with response assessment in the 1.8 mg kg^−1^ cohort and for 37 of 42 patients in the 2.5 mg kg^−1^ cohort. Data are presented as median H-score and interquartile range (IQR). 3L+, third line and beyond; CR, complete response; IHC, immunohistochemistry; PD, progressive disease; PR, partial response; SD, stable disease.

**Fig. 3:**
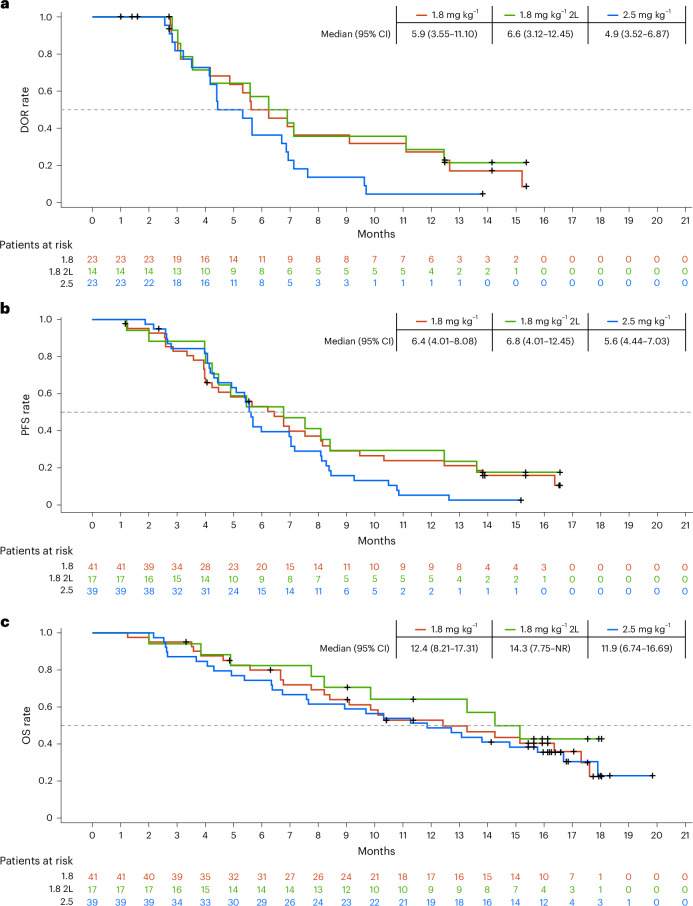
Efficacy among patients with R/R SCLC. **a**, Primary endpoint of DOR by dose for the part 2a dose-optimization cohorts. **b**, Exploratory endpoints of PFS by dose for the part 2a dose-optimization cohorts. **c**, OS by dose for the part 2a dose-optimization cohorts of 1.8 mg kg^−1^ and 2.5 mg kg^−1^. NR, not reached.

As part of the determination of the RP2D, preliminary efficacy data are further summarized by part 2a dose-optimization cohorts including the 1.8 mg kg^−1^ cohort (*n* = 41), the 1.8 mg kg^−1^ 2L cohort (*n* = 17) and the 2.5 mg kg^−1^ cohort (*n* = 39) in Table 3 and Fig. 2. Rapid and durable responses were seen across both 1.8 mg kg^−1^ and 2.5 mg kg^−1^ doses (Fig. 2a−d). The ORR was 56% in the 1.8 mg kg^−1^ cohort and 59% in the 2.5 mg kg^−1^ cohort (Table 3). The DOR at 1.8 mg kg^−1^ was 5.9 months (95% CI: 3.6−11.1) and at 2.5 mg kg^−1^ was 4.9 months (95% CI: 3.5−6.9) (Fig. 3a). With a median follow-up of 17.5 months and 16.8 months, the median PFS in the 1.8 mg kg^−1^ and 2.5 mg kg^−1^ cohorts was 6.4 months (95% CI: 4.0−8.1) and 5.6 months (95% CI: 4.4−7.0) (Table 3 and Fig. 3b), respectively. The median OS was 12.4 months (95% CI: 8.2−17.3) in the 1.8 mg kg^−1^ cohort and 11.9 months (95% CI: 6.7−16.7) in the 2.5 mg kg^−1^ cohort, respectively (Table 3 and Fig. 3c). Landmark OS estimate at 15 months was 44% (95% CI: 27−59) and 38% (95% CI: 23−53), respectively.

**Table 3:** Summary of efficacy parameters by dose in patients with R/R SCLC, including those enrolled in the 1.8 mg kg^−1^ and 2.5 mg kg^−1^ dose-optimization cohorts

	Part 2a 1.8 mg kg^−1^ (*n* = 41)	Part 2a 1.8 mg kg^−1^ 2L (*n* = 17)	Part 2a 2.5 mg kg^−1^ (*n* = 39)	Part 1 + 2a + 2c (*N* = 124)
ORR,^a^ *n* (%)	23 (56.1)	14 (82.4)	23 (59.0)	65 (52.4)
(95% CI)	(39.7−71.5)	(56.6−96.2)	(42.1−74.4)	(44.0−62.4)
Confirmed best response,^a^ *n* (%)				
CR	3 (7.3)	2 (11.8)	0	3 (2.4)
PR	20 (48.8)	12 (70.6)	23 (59.0)	62 (50.0)
SD	16 (39.0)	2 (11.8)	14 (35.9)	46 (37.1)
PD	1 (2.4)	1 (5.9)	0	6 (4.8)
NE	0	0	1 (2.6)	2 (1.6)
NA	1 (2.4)	0	1 (2.6)	3 (2.4)
Clinical benefit rate,^b^ *n* (%)	39 (95.1)	16 (94.1)	37 (94.9)	111 (89.5)
(95% CI)	(83.5−99.4)	(71.3−99.9)	(82.7−99.4)	(84.4−95.4)
Median DOR, months (95% CI)	5.93 (3.55−11.10)	6.57 (3.12−12.45)	4.88 (3.52−6.87)	5.32 (4.14−6.70)
Median PFS, months (95% CI)	6.44 (4.01−8.08)	6.77 (4.01−12.45)	5.62 (4.44−7.03)	5.39 (4.40−5.68)
Median OS, months (95% CI)	12.42 (8.21−17.31)	14.26 (7.75−NE)	11.86 (6.74−16.69)	11.27 (9.10−14.78)
OS estimate at 15 months (95% CI)	0.44 (0.27−0.59)	0.50 (0.24−0.71)	0.38 (0.23−0.53)	0.40 (0.30−0.49)

^a^Investigator-assessed confirmed CR + PR.

^b^Confirmed CR + PR + SD.

**Extended Data Fig. 2:**
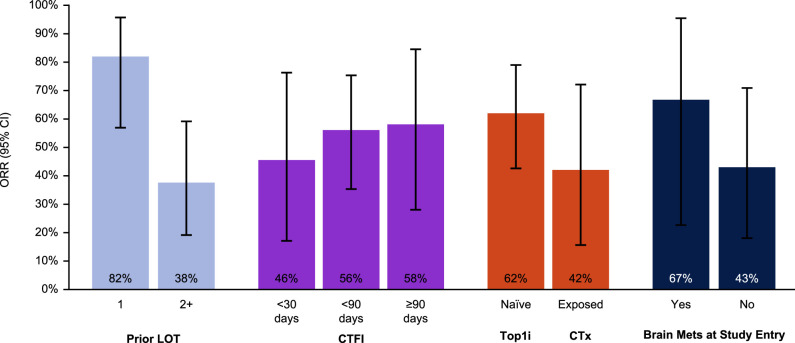
ORR by CTFI, prior LOT, prior Top1i chemotherapy exposure, and brain metastasis at study entry among patients in the 1.8 mg kg^−^^1^ cohort. CTFI, chemotherapy free interval; CTx, chemotherapy; LOT, line of therapy; Mets, metastasis; ORR, objective response rate; Top1i, topoisomerase 1 inhibitor.

**Extended Data Fig. 3:**
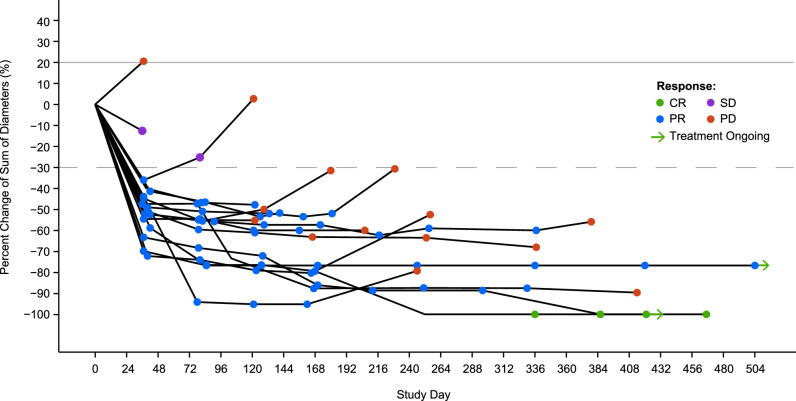
Percent change in target lesion measurement from baseline over time per RECIST v1.1 in patients in the part 2a 1.8 mg kg^−1^ 2L cohort. 2L, second line; CR, complete response; PD, progressive disease; PR, partial response; RECIST v.1.1, Response Evaluation Criteria in Solid Tumors version 1.1; SCLC, small cell lung cancer; SD, stable disease.

As a post hoc analysis, we further explored efficacy outcomes in all patients with R/R SCLC who received 1.8 mg kg^−1^. In patients at the 1.8 mg kg^−1^ dose who were Top1i naive versus Top1i exposed, the ORR was 62% versus 42%, respectively. Notably, patients with platinum-resistant (<90 days CTFI) or refractory (<30 days CTFI) disease had a similar ORR to the overall SCLC patient population (Supplementary Table 2 and Extended Data Fig. 2); this trend was observed for PFS as well in these patients. Additionally, among patients who received 1.8 mg kg^−1^ in the 2L setting (*n* = 17), the ORR was 82% and the median DOR was 6.6 months (95% CI: 3.1−12.5) (Table 3 and Fig. 3a). Rapid response rates were also observed in these patients (Extended Data Fig. 3). With a median follow-up of 17.5 months, the median PFS was 6.8 months (95% CI: 4.0−12.5) (Table 3 and Fig. 3b). Median OS was 14.3 months (95% CI: 7.8−not estimable) (Fig. 3c), and landmark OS estimate at 15 months was 50% (95% CI: 24−71).

### Exploratory biomarker analysis

**Extended Data Fig. 4:**
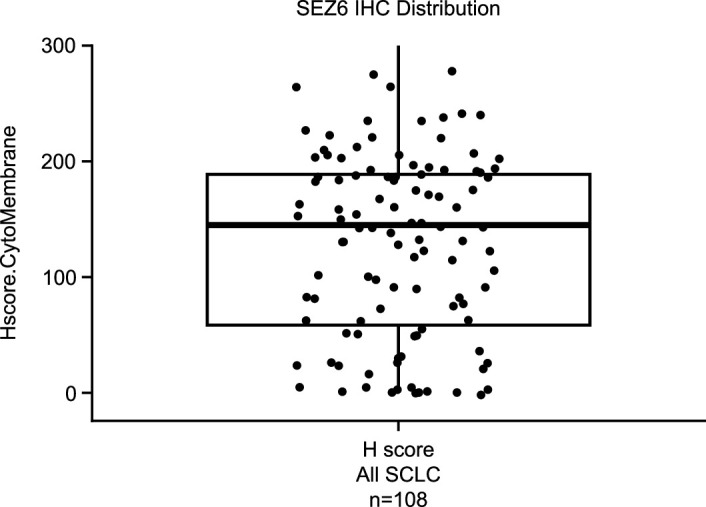
Distribution of SEZ6 expression among all patients with SCLC and available tumor sample. Data presented as median H-score and interquartile (IQR) range. IHC, immunohistochemistry; SCLC, small cell lung cancer; SEZ6, seizure-related homolog 6.

**Extended Data Fig. 5:**
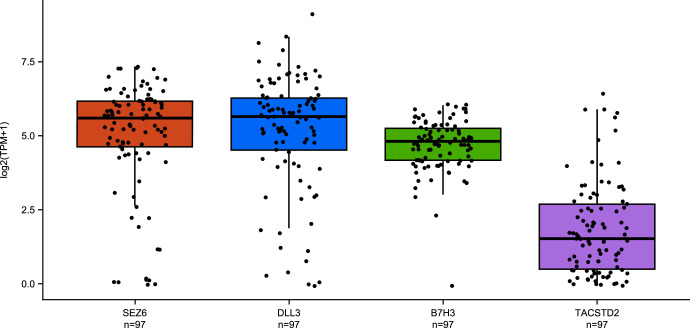
SEZ6 tumor mRNA expression compared to other known biomarkers among patients with SCLC. SEZ6, seizure-related homolog 6; SCLC, small cell lung cancer; TPM, transcripts per million. *n* = 97 SCLC tumor samples with available RNA data.

**Extended Data Fig. 6:**
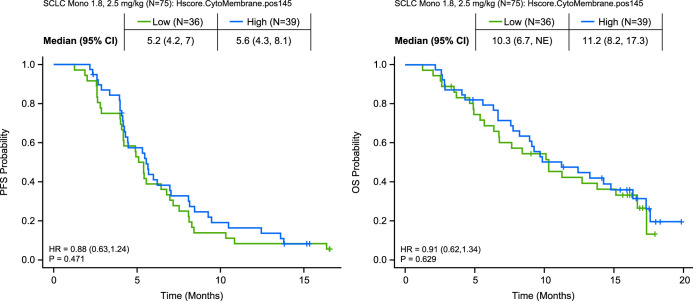
Impact of SEZ6 expression on PFS and OS among patients in the 1.8 mg kg^−^^1^ and 2.5 mg kg^−^^1^ dose cohorts, stratified by median SEZ6 cytomembrane H-score of 145 into low (*n* = 36) and high (*n* = 39) cohorts. HR, hazard ratio; NE, not estimable; PFS, progression free survival; OS, overall survival; SEZ6, seizure-related homolog 6.

**Extended Data Fig. 7:**
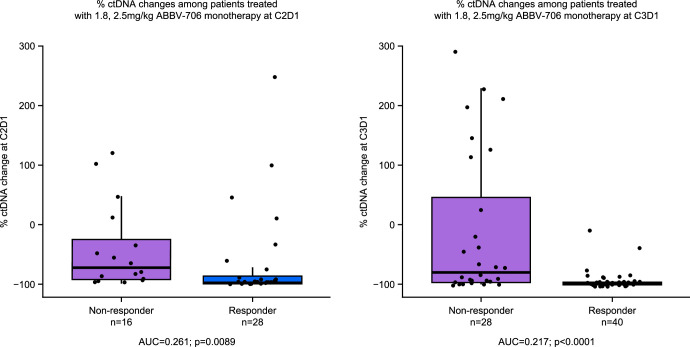
% ctDNA changes at C2D1 and C3D1 compared to baseline C1D1 among responders and non-responders. As measured by tumor-based methylation fraction. AUC, area under the curve; ctDNA, circulating tumor DNA; SCLC, small cell lung cancer. n = 44 patients at C2D1 and n = 68 patients at C3D1 with available ctDNA data.

SEZ6 expression is highly prevalent and expressed among most patients with SCLC, with tumor SEZ6 expression detected by immunohistochemistry in 93% of patients whose tissue samples were available, above or equal to 1% with 1+ intensity (108 patients from all SCLC cohorts from trial M23-385) (Extended Data Table 3). The median SEZ6 cytomembrane H-score was 145, with 25th and 75th percentile H-scores of 58.5 and 190, respectively (Extended Data Fig. 4). There was also high SEZ6 expression in patients with SCLC compared to other known biomarkers (for example, DLL3) (Extended Data Fig. 5). Complementing the efficacy data, evaluable SEZ6 expression data at baseline were available for 38 of 48 patients having responses per RECIST version 1.1 in the 1.8 mg kg^−1^ cohort and for 37 of 44 patients in the 2.5 mg kg^−1^ cohort from parts 1 and 2a of the study (Fig. 2e). In the 1.8 mg kg^−1^ cohort, the median SEZ6 cytomembrane H-score was 153, with 25th and 75th percentile H-scores of 67 and 185, respectively. This is similar (*P* = 0.7424, two-sided Wilcoxon rank-sum test) to SEZ6 expression distribution in the 2.5 mg kg^−1^ cohort (median SEZ6 cytomembrane H-score of 130, with 25th and 75th percentile H-scores of 64 and 200, respectively) (Fig. 2e). Responses based on ORR were observed across SEZ6 expression levels in both 1.8 mg kg^−1^ and 2.5 mg kg^−1^ cohorts (Fig. 2f) and did not correlate with PFS and OS (Extended Data Fig. 6). ABBV-706 triggered a rapid and deep decline in circulating tumor DNA (ctDNA) among patients of 1.8 mg kg^−1^ and 2.5 mg kg^−1^ cohorts as early as cycle 2 day 1 (C2D1) and cycle 3 day 1 (C3D1), which was significantly more pronounced in responders compared to non-responders (Extended Data Fig. 7).

## Discussion

The current phase 1 study of ABBV-706 monotherapy in SEZ6-expressing neuroendocrine lineage malignancies establishes SEZ6 as a valid therapeutic target specifically in SCLC. Although efficacy is preliminary, durable objective responses were seen across doses of 1.3−3.5 mg kg^−1^ in SCLC in dose escalation and at the two doses evaluated during dose optimization in SCLC. Doses of 1.8 mg kg^−1^ and 2.5 mg kg^−1^ were selected for dose optimization based on the preliminary safety and efficacy profile observed in dose escalation (part 1). Data from part 2 randomized dose-optimization 1.8 mg kg^−1^ and 2.5 mg kg^−1^ cohorts demonstrate that both doses had similarly high ORR. In addition, the preliminary ORR was 82% in patients who had previously received only one platinum-based line of therapy (1.8 mg kg^−1^ 2L cohort) and was 38% in patients who received two or more prior systemic chemotherapies, indicating that patients in earlier lines of treatment may derive benefit from ABBV-706, although these data need to be confirmed in future trials. Response rates higher than 60% were observed in patients who had no previous exposure to other Top1is, such as topotecan or irinotecan, whereas response rates were 42% in patients with prior exposure. These data suggest that patients previously treated with Top1is may inherently develop resistance to the payload due to a similar mechanism of action. A high ORR, similar to with the overall SCLC cohort, was also seen in the subsets of patients who had a CTFI <90 days (platinum resistant) or CTFI <30 days (platinum refractory) with prior platinum therapy. These preliminary efficacy results are highly encouraging as, historically, these patients have response rates lower than 20% with current approved standard-of-care systemic chemotherapies, such as topotecan, amrubicin or lurbinectedin^2^. In addition, the 1.8 mg kg^−1^ dose exhibited numerically higher DOR, PFS and OS than the 2.5 mg kg^−1^ dose, an important factor in the RP2D selection, with potentially increased benefit in patients with only one prior line of therapy (that is, the 1.8 mg kg^−1^ 2L cohort). Although these preliminary findings suggest that activity of ABBV-706 monotherapy in heavily pretreated patient populations may be robust and highly encouraging compared to first-line treatments, this needs to be further explored and confirmed in larger randomized trials, such as the ongoing SEZanne phase 2 trial (NCT07155174).

Adverse events were dose dependent and manageable at the optimized doses selected for further evaluation, with key adverse events similar to those reported for other Top1i payload ADCs. The most common toxicities were hematologic (cytopenias), gastrointestinal (nausea and vomiting) and fatigue. Cytopenias were dose dependent in grade and severity and were manageable with supportive care, whereas gastrointestinal toxicities were generally low grade and not dose dependent. A low rate of adjudicated pneumonitis/ILD was seen in the overall monotherapy cohort (*N* = 240); notably, this was the most common cause of grade 5 TRAE. Only two cases of pneumonitis (among 240 patients) led to death considered related to treatment. Although the maximum tolerated dose was established at 3 mg kg^−1^, this dose was not evaluated in the dose-optimization or dose-expansion cohorts, as most patients required dose interruptions and reductions in subsequent cycles that led to lower dose intensities in the higher dose levels assessed. In dose optimization, as in dose escalation, adverse events were seen to be dose dependent, and there was a lower frequency of grade 3 or higher adverse events, dose interruptions and dose reductions at 1.8 mg kg^−1^ compared to 2.5 mg kg^−1^, which also led to a lower relative dose intensity for the 2.5 mg kg^−1^ cohort that may have impacted the observed efficacy between the two doses. Given the totality of the data, the overall benefit−risk profile favored the selection of 1.8 mg kg^−1^ as the RP2D for ABBV-706 monotherapy in R/R SCLC.

Our retrospective analysis of SEZ6 expression in tissue samples confirmed the high prevalence of expression in SCLC, with more than 90% of patient samples expressing some level of SEZ6. Exploratory analyses also demonstrated high levels of SEZ6 expression compared to other known biomarkers in patients with SCLC. Responses were seen across expression levels, including those with low expression. No correlation of SEZ6 expression was observed with PFS and OS based on median H-score in this SCLC clinical dataset. Future analyses will be conducted in a randomized controlled study to further determine the correlation of SEZ6 expression with clinical response. Finally, rapid and deep ctDNA decline was observed with ABBV-706 treatment. Impact on time-to-event outcomes such as DOR, PFS and OS will need to be confirmed in larger phase 2/3 randomized controlled trials, but the preliminary data from this study support continued evaluation of SEZ6 expression in a retrospective manner in future SCLC confirmatory trials to further delineate the impact of biomarker expression on efficacy.

The strengths of our study include robust randomized dose optimization, which allowed for a well-balanced comparison of the doses tested to inform RP2D. In addition, unlike other studies, this trial did not limit the number of prior lines of therapy, thereby including a heavily pretreated population, including patients with active brain metastasis who are often excluded from other phase 1 trials, and enrolled patients whose disease was platinum refractory. All of these factors reflect the potential strength and broad clinical applicability of our data.

As a phase 1 study, this study has limitations, including that it was non-randomized and open label and was not powered to assess efficacy as there was no hypothesis testing. Intracranial response rates are of interest but were not prespecified and may be considered for future publications. One additional limitation of this study is the lack of comparator standard-of-care arms. This will be addressed in future phase 2/3 studies of ABBV-706. Furthermore, although tarlatamab has emerged as a new standard of care for patients with previously treated SCLC, few patients enrolled in our study had prior tarlatamab exposure, and increased enrollment of these patients could be explored in future trials.

The treatment landscape for SCLC is rapidly evolving. Response rates to the current standard of care, including topotecan, amrubicin and lurbinectedin, are usually modest, with limited DOR and poor OS outcomes in patients with R/R SCLC. The preliminary response rates in our study for R/R SCLC ranged from 52% to 82% and were previously reported as follows for topotecan (ORR: 17−24%), lurbinectedin (ORR: 35%) and amrubicin (ORR: 31%) in patients with R/R SCLC^12–14^. In addition, tarlatamab, a Delta-like ligand 3 bispecific T cell engager, received approval for extensive-stage SCLC after platinum-based chemotherapy and is a preferred 2L treatment, on the basis of data from the DeLLphi-301 and DeLLphi-304 studies showing an ORR of 35−40%, a median DOR of 6.9−9.7 months^15,16^, a median OS of 13.6 months (hazard ratio = 0.60) and a median PFS of 4.2 months (hazard ratio = 0.72)^16^. B7-H3-targeted ADCs have also emerged as another promising target in SCLC, with several agents currently in development^17,18^. However, there remains a large unmet need in SCLC therapy, and highly active agents such as ABBV-706 have the potential to substantially improve outcomes in patients with this disease.

In conclusion, ABBV-706 has a manageable safety profile and demonstrated promising preliminary efficacy in heavily pretreated patients with R/R SCLC, with most patients achieving durable clinical benefit. Dose optimization confirmed 1.8 mg kg^−1^ Q3W as the optimal monotherapy regimen for patients with R/R SCLC. Our data strongly support further studies of ABBV-706 in registrational trials in SCLC with the potential to positively impact the future treatment landscape in this disease. A first-line trial of ABBV-706 in combination with atezolizumab as a potential alternative to standard-of-care first-line etoposide (with or without immunotherapy) (NCT07155174) has been initiated and will provide further insights on the placement of ABBV-706 in the SCLC treatment landscape.

## Methods

### Ethical approval and consent

This study was conducted in accordance with the principles of the Declaration of Helsinki and Good Clinical Practice guidelines of the International Conference on Harmonization and was approved by the regulatory and independent ethics committees/institutional review boards (IRBs) at each site. All patients provided written informed consent. The central IRB was Advarra, and each site used either the local or central IRB for protocol approval (full listing provided in Supplementary Table 3). The protocol was approved on 12 August 2022, was submitted to ClinicalTrials.gov (NCT05599984) on 28 October 2022 and was posted on 31 October 2022.

### Trial design and treatment

This phase 1, open-label, dose-escalation and dose-expansion study of ABBV-706 in patients with advanced solid tumors with potential SEZ6 expression comprised four parts (Fig. 1): part 1 was dose escalation of ABBV-706 monotherapy in patients with advanced solid tumors; part 2 was dose optimization/expansion of ABBV-706 monotherapy in patients with R/R SCLC whose disease progressed on or after post-platinum chemotherapy; and part 4 was dose expansion of ABBV-706 monotherapy in R/R CNS tumors (4a) and high-grade NEC/NETs (4b). Part 3 was the combination of ABBV-706 with the anti-PD-1 antibody budigalimab (3a) or with platinum chemotherapy (3b) and is not reported in this paper.

In part 1, ABBV-706 was administered intravenously Q3W in escalating doses between 1.3 mg kg^−1^ and 3.5 mg/kg^−1^, following a Bayesian optimal interval design. In part 2a, patients with R/R SCLC were randomized evenly to receive ABBV-706 1.8 mg kg^−1^ or 2.5 mg kg^−1^ Q3W to determine the RP2D; no stratification factors were applied. Monotherapy dose-expansion part 2b was not pursued as part of this trial, but similar patient populations are being pursued in independent trials (NCT07155174 and NCT07365241). In part 2c, patients with R/R SCLC in China underwent a safety run-in followed by a dose expansion of ABBV-706 at 1.8 mg kg^−1^. In part 4, patients with R/R CNS tumors or high-grade NENs received ABBV-706 at 2.5 mg kg^−1^ Q3W. Patients received ABBV-706 until disease progression, until intolerable toxicity or until other treatment discontinuation criteria were met.

### Protocol amendments

A summary of all amendments that were implemented over the course of the study are provided in the Supplementary Information (amendments section). Key amendments include amendment 1, which revised the objectives and eligibility of the study after health authority review. Amendments 3 and 4 included a Japan-only cohort (part 1b) and corresponding Japan-specific eligibility, safety, biomarker and testing requirements. Amendments 7 and 8 added a China-only cohort (part 2c) and corresponding China-specific eligibility, safety, biomarker and adverse event reporting requirements; we do not report here on the biomarker data for the China-specific cohort.

### Patients

Adults (aged ≥18 years) with histologically confirmed advanced SCLC, high-grade NENs or high-grade CNS tumors who had experienced progression on or after standard-of-care treatment with no curative therapy available were enrolled in parts 1, 2 and 4 as delineated above in study design. Key inclusion criteria included measurable disease per RECIST version 1.1 or Response Assessment in Neuro-Oncology (RANO) (for patients with primary high-grade CNS tumors), ECOG PS 0 or 1 and availability of fresh or archival tumor tissue for retrospective SEZ6 expression analysis. Of note, patients with brain metastases were eligible if the metastases were either previously treated, were currently stable and not requiring steroids or anticonvulsants, or were untreated, asymptomatic, with fewer than five lesions each <5 mm in size and not requiring steroids or anticonvulsants to control associated symptoms. Key exclusion criteria included prior treatment with an ADC containing a Top1i payload for all parts and prior treatment with a SEZ6-targeted ADC in part 2. Full eligibility criteria are listed in the Protocol.

### Outcomes and assessments

The primary objectives of parts 1, 2 and 4 of the study were to evaluate the safety, tolerability, PK, immunogenicity and antitumor activity of ABBV-706 monotherapy and, from parts 1 and 2, to determine the RP2D of ABBV-706 in R/R SCLC. Exploratory objectives included evaluation of clinical benefit rate, PFS, OS and potential association of SEZ6 expression with antitumor activity.

Adverse events were summarized using Medical Dictionary for Regulatory Activities preferred terms, and severity was graded per National Cancer Institute Common Terminology Criteria for Adverse Events version 5.0, except for ILD/pneumonitis, for which American Society of Clinical Oncology grading criteria were used^19^. In addition, a central ILD adjudication committee retrospectively reviewed suspected cases of ILD. PK endpoints included maximum observed serum (or plasma for payload) concentration (*C*_max_), time to reach *C*_max_ (*T*_max_), terminal phase elimination half-life and area under the serum (or plasma for payload) concentration versus time curve (AUC) for ABBV-706 conjugate, total antibody and unconjugated (free) Top1i payload. PK parameters were determined using non-compartmental methods. Immunogenicity assessments for ABBV-706 ADAs were evaluated across multiple timepoints. Additional planned exploratory analysis not reported here include a preliminary concentration−QTc analysis to evaluate the relationship between the change in the QTc interval from baseline with correction for heart rate using QTcF (Fredericia) and free Top1i payload (A-1743332) concentrations. Triplicate 12-lead resting electrocardiograms and time‑matched PK samples were obtained.

The primary efficacy outcome was investigator-assessed ORR (complete response or partial response) per RECIST version 1.1. All ORRs were confirmed with two or more responses of partial response or better, with an initial complete response or partial response confirmed in an assessment at least 4 weeks later. For part 2, an independent central review for response assessment was also conducted.

### Statistical analysis

In this paper, the safety analysis included all patients who received one or more doses of ABBV-706 monotherapy (parts 1, 2 and 4); safety parameters were tabulated overall and by dose cohort (that is, safety analysis population). PK analysis consisted of all patients who received one or more doses of study drug as monotherapy and had one or more quantifiable concentrations of ABBV-706. Efficacy analyses were conducted in the overall R/R SCLC population (from parts 1 and 2 (2a + 2c)) and by dose level in study part 2a. Point estimates along with two-sided 95% CIs for ORR were calculated using the Clopper−Pearson (exact) method. Patients who had not experienced disease progression or died were censored at the last disease assessment prior to the data cutoff date or start of any subsequent anticancer therapy, whichever occurred earlier. Kaplan−Meier estimates for median PFS, DOR and OS were calculated with their associated two-sided 95% CIs by dose cohort.

All analyses are descriptive as no formal hypothesis testing was planned, and no formal sample size calculations or power calculations were performed. Sample size, although not formally calculated, was estimated based on the lower bound of a one-sided 80% CI for ORR that would be sufficient for providing a preliminary assessment of efficacy and was estimated at approximately *n* = 40; in total, the number of patients expected to enroll across all study parts was 317. Sex was collected and self-reported by patients but was not considered in the study design, and no analyses were stratified on the basis of sex, as this was a phase 1 proof-of-concept trial focusing on safety and preliminary efficacy across all patients. All analyses were performed using SAS version 9.4 (SAS Institute) or later.

### Exploratory biomarker analysis

In patients with R/R SCLC, SEZ6 expression was determined retrospectively using an AbbVie-developed immunohistochemistry assay on tumor biopsy specimens obtained at any time prior to ABBV-706 treatment. SEZ6 expression was measured by percentage of tumor cells with different intensities of cytomembrane staining and further described by the cytomembrane H-score, which is calculated as (1 × percentage of tumor cells with 1+ SEZ6 cytomembrane staining) + (2 × percentage of tumor cells with 2+ SEZ6 cytomembrane staining) +(3 × percentage of tumor cells with 3+ SEZ6 cytomembrane staining), ranging from 0 to 300. SEZ6 expression was compared between responders (confirmed complete response + partial response) and non-responders. Kaplan−Meier estimates for median PFS and OS were calculated with their associated 95% CIs by SEZ6 H-score dichotomized at the median. The PFS and OS distributions between SEZ6-low and SEZ6-high groups were compared using the log-rank test. Hazard ratios and corresponding two-sided 95% CIs were estimated using a Cox proportional hazards model. mRNA expression of SEZ6 and other biomarkers was evaluated with the Illumina TruSeq RNA Exome assay with RNA extracted from archived tumor tissue or formalin-fixed paraffin-embedded fresh tumor biopsy at screening. ctDNA methylation-based percentage tumor fraction change prior to treatment at C1D1 and during the treatment course was measured by the Guardant Infinity platform (Guardant Health). Extent of ctDNA reduction at C2D1 as well as C3D1 from C1D1 was compared between responders (confirmed complete response + partial response) and non-responders. Exploratory biomarker analyses for the part 2c China study cohort are not reported here.

### Reporting summary

Further information on research design is available in the Nature Portfolio Reporting Summary linked to this article.

## Online content

Any methods, additional references, Nature Portfolio reporting summaries, source data, extended data, supplementary information, acknowledgements, peer review information; details of author contributions and competing interests; and statements of data and code availability are available at 10.1038/s41591-026-04452-0.

## Supplementary information

Supplementary Information Supplementary Methods, Tables 1–3 and Protocol (summary of protocol amendments and final protocol).

Reporting Summary

## Figures and Tables

**Extended Data Table 1:**
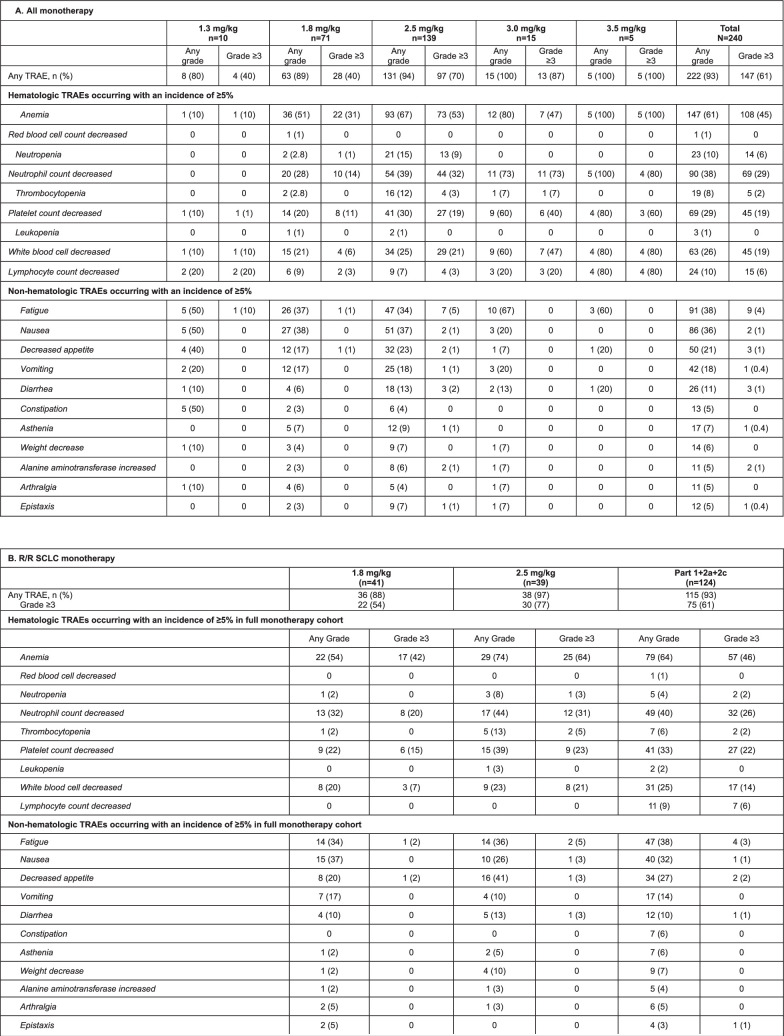
Summary of all TRAEs occurring in ≥ 5% of patients among (A) all monotherapy patients and (B) patients with SCLC in the 1.8 mg kg^−^^1^ and 2.5 mg kg^−^^1^ part 2 dose-optimization cohorts

SAE, serious adverse event; SCLC, small cell lung cancer; TRAE, treatment-related adverse event.

**Extended Data Table 2:**
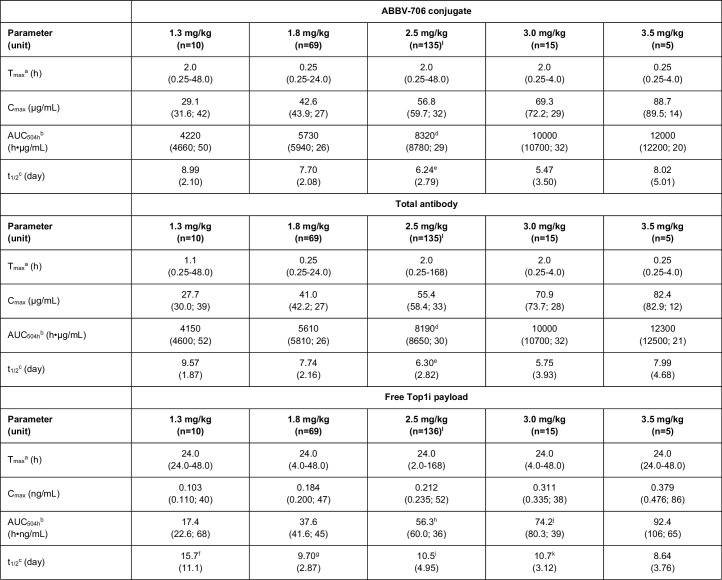
Summary of PK parameters for ABBV-706 conjugate, total antibody, and free Top1i payload in cycle 1 following Q3W dosing as monotherapy (geometric mean [arithmetic mean, %CV])

^a^T_max_ presented as median (minimum-maximum); reported as time relative to the end of infusion. ^b^AUC_504h_ = area under the concentration-time curve up to 504 hours. ^c^t_1/2_ presented as harmonic mean (pseudo-standard deviation). ^d^n = 101. ^e^n = 100. ^f^n = 4. ^g^n = 34. ^h^n = 79. ^i^n = 57. ^j^n = 13. ^k^n = 10. ^l^Sample sizes differ between analytes due to the lack of sufficient samples to include in the non-compartmental analysis. Summaries include all viable PK parameters.

C_max_, maximum observed concentration; CV, coefficient of variation; PK, pharmacokinetic; Q3W, every 3 weeks; t_1/2_, terminal phase elimination half-life; T_max_, time to C_max_; Top1i, topoisomerase 1 inhibitor.

**Extended Data Table 3:**
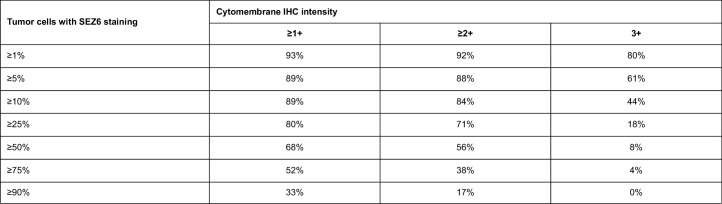
Prevalence of SEZ6 expression by immunohistochemistry among patients with SCLC

IHC, immunohistochemistry; SCLC, small cell lung cancer; SEZ6, seizure-related homolog 6.

## Data Availability

AbbVie is committed to responsible data sharing regarding the clinical trials that we sponsor. This includes access to anonymized, individual and trial-level data (analysis datasets), as well as other information (for example, protocols, clinical study reports or analysis plans), as long as the trials are not part of an ongoing or planned regulatory submission. This includes requests for clinical trial data for unlicensed products and indications. These clinical trial data can be requested by any qualified researchers who engage in rigorous, independent, scientific research and will be provided after review and approval of a research proposal and a statistical analysis plan and the execution of a data-sharing agreement. Data requests can be submitted at any time, with acknowledgement of the request within 2 weeks. The data will be accessible for 12 months, with possible extensions considered. For more information on the process or to submit a request, visit https://vivli.org/ourmember/abbvie/ and then select ‘Home’.
