# Author Correction: SEZ6-targeting antibody−drug conjugate ABBV-706 in advanced small cell lung cancer and solid tumors: a phase 1 trial

**DOI:** 10.1038/s41591-026-04544-x

**Published:** 2026-06-29

**Authors:** Lauren Averett Byers, Byoung Chul Cho, Alissa J. Cooper, Anne C. Chiang, Ji-Youn Han, Muhammad Furqan, Afshin Dowlati, Daniel Morgensztern, Kyriakos P. Papadopoulos, Noura J. Choudhury, María Vieito, Jair Bar, Joo-Hang Kim, Wallace Akerley, Tae Min Kim, Young-Chul Kim, Luis Paz-Ares, Myung-Ju Ahn, Hiroshi Yokouchi, Darius Meiman, Wijith Munasinghe, Oluwadamilola Ogunyankin, Nadine Jahchan, Song Wang, Cristiano Ferlini, Randy R. Robinson, Frederick J. Kohlhapp, Tammy Palenski, Guillermo Rivell, Pooja Hingorani, Sreenivasa Chandana

**Affiliations:** 1 The University of Texas MD Anderson Cancer Center, Houston, TX USA; 2 Division of Medical Oncology, Yonsei University College of Medicine, Yonsei Cancer Center, Seoul, Republic of Korea; 3 Department of Thoracic Oncology, Memorial Sloan Kettering Cancer Center, New York, NY USA; 4 Yale School of Medicine, New Haven, CT USA; 5 National Cancer Center, Gyeonggi-do, Republic of Korea; 6 University of Iowa Holden Comprehensive Cancer Center, Iowa City, IA USA; 7 University Hospitals Seidman Cancer Center and Case Western Reserve University, Cleveland, OH USA; 8 Washington University School of Medicine, St. Louis, MO USA; 9 START San Antonio, San Antonio, TX USA; 10 Department of Medicine, University of Chicago, Chicago, IL USA; 11 Department of Medical Oncology, Vall d’Hebron Barcelona Hospital Campus, Barcelona, Spain; 12 Vall d’Hebron Institute of Oncology, Barcelona, Spain; 13 Jusidman Cancer Center, Sheba Medical Center, Ramat Gan, Israel; 14 The Gray Faculty of Medical and Health Sciences, Tel Aviv University, Tel Aviv, Israel; 15 Department of Internal Medicine, CHA Bundang Medical Center, CHA University, Seongnam, Republic of Korea; 16 Division of Medical Oncology, Huntsman Cancer Institute, University of Utah, Salt Lake City, UT USA; 17 Department of Internal Medicine, Seoul National University Hospital, Seoul National University College of Medicine, Seoul, Republic of Korea; 18 Department of Internal Medicine, Chonnam National University Medical School, and Chonnam National University Hwasun Hospital, Jeonnam, Republic of Korea; 19 Hospital Universitario 12 de Octubre and Universidad Complutense, Madrid, Spain; 20 Section of Hematology-Oncology, Department of Medicine, Hanyang University Medical Center, Hanyang University, Seoul, Republic of Korea; 21 National Hospital Organization Hokkaido Cancer Center, Sapporo, Japan; 22 AbbVie Inc., North Chicago, IL USA; 23 START Midwest, Grand Rapids, MI USA

**Keywords:** Small-cell lung cancer, Cancer therapy

Correction to: *Nature Medicine* 10.1038/s41591-026-04452-0, published online 1 June 2026.

In the version of this article initially published, ref. 10 was cited twice and has been replaced by an omitted reference (Faivre E. J. et al. *Cancer Re*s. 10.1158/1538-7445.AM2024-3148 (2024)), now cited as ref. 11 in the last paragraph of the introduction: “Preclinical studies in patient-derived xenograft models of SEZ6-positive SCLC demonstrated superior activity of ABBV-706 compared to ABBV-011 and standard-of-care chemotherapies^10^. Preliminary results from the ongoing first-in-human phase 1 trial (NCT05599984) to evaluate the safety, tolerability, pharmacokinetics (PK), immunogenicity and antitumor activity of ABBV-706 in patients with R/R solid tumors (defined as those patients whose disease had progressed on or after treatment with standard of care), including patients with R/R SCLC, demonstrated that ABBV-706 monotherapy had a manageable safety profile and promising antitumor activity, with a confirmed ORR of 43.8% (60.9% in patients with R/R SCLC)^11^.” The reference has been updated in the HTML and PDF version of the article.

