# Supplementary material for: SEZ6-targeting antibody−drug conjugate ABBV-706 in advanced small cell lung cancer and solid tumors: a phase 1 trial
Source: Nat Med. 2026 Jun 1;32(8):2898–908. doi: 10.1038/s41591-026-04452-0 (PMC13472869; doi:10.1038/s41591-026-04452-0)
Supplement: Supplementary file 1 [file 41591_2026_4452_MOESM1_ESM.pdf]

nature medicine

Supplementary information https://doi.org/10.1038/s41591-026-04452-0

SEZ6-targeting antibody–drug conjugate ABBV-706 in advanced small cell lung cancer and solid tumors: a phase 1 trial

In the format provided by the authors and unedited

Supplementary Material

Statistical analysis

Progression-free survival was defined as the time from first study treatment to disease progression according to Response Evaluation Criteria in Solid Tumors version 1.1 (RECIST v1.1), as determined by the investigator, or death due to any cause, whichever occurred earlier. Duration of response was defined as the time from the initial response of complete response (CR) or partial response (PR) per investigator according to RECIST v1.1 to disease progression or death of any cause, whichever occurred earlier; patients without confirmed CR or PR were excluded from analysis. Overall survival (OS) was defined as time from first study treatment to death due to any cause. For patients who were not known to have died at the time of analysis cutoff, OS was censored at the last known alive date on or prior to the data cutoff.

Supplementary Tables and Figures

Supplementary Table S1. Summary of efficacy parameters[a] for all RECIST-evaluable patients with R/R SCLC who received ABBV-706 monotherapy.

	**1.3 mg/kg (n=8)**	**1.8 mg/kg (n=63)**	**2.5 mg/kg (n=44)**	**3.0 mg/kg (n=7)**	**3.5 mg/kg (n=2)**	**Total (N=124)**
ORR,[b] n (%)	3 (38)	30 (48)	25 (57)	5 (71)	2 (100)	65 (52)
Clinical benefit rate,[c] n (%)	5 (63)	57 (90)	41 (93)	6 (86)	2 (100)	111 (90)
Median DOR, months (95% CI)	8.15 (2.79, NE)	5.59 (3.12, 7.13)	4.42 (3.52, 6.70)	5.68 (2.96, NE)	4.68 (2.60, NE)	5.32 (4.40, 6.70)
Median PFS, months (95% CI)	2.76 (1.25, NE)	4.90 (3.94, 6.77)	5.42 (4.21, 5.98)	6.83 (1.25, 21.91)	6.90 (5.55, NE)	5.39 (4.40, 5.68)

[a]Investigator assessed. [b]Confirmed CR + PR. [c]Confirmed CR + PR + SD.
CI, confidence interval; CR, complete response; DOR, duration of response; NE, not estimable; ORR, objective response rate; PD, progressive disease; PFS, progression-free survival; PR, partial response; R/R, relapsed/refractory; RECIST, Response Evaluation Criteria in Solid Tumors; SCLC small cell lung cancer; SD, stable disease.

Supplementary Table S2. Select subgroup analysis in patients with SCLC enrolled at 1.8 mg/kg in the dose-optimization cohort.							
	**Platinum refractory (CTFI < 30 days) (n = 11)**	**Platinum resistant (CTFI < 90 days) (n = 25)**	**Platinum sensitive (CTFI ≥ 90 days) (n = 12)**	**Top1i exposed (n = 12)**	**Top1i naïve (n = 29)**	**Brain metastasis at study entry (Yes) (n=6)**	**Brain metastasis at study entry (No) (n=14)**
ORR,[a] n (%)<br>(95% CI)	5 (45)<br>(17, 77)	14 (56)<br>(35, 76)	7 (58)<br>(28, 84.8)	5 (42)<br>(15, 72)	18 (62)<br>(42, 79)	4 (67)<br>(22, 96)	6 (43)<br>(18, 71)
Clinical benefit rate,[b] n (%)<br>(95% CI)	10 (91)<br>(59, 100)	24 (96)<br>(80, 100)	11 (92)<br>(62, 100)	12 (100)<br>(74, 100)	27 (93)<br>(77, 99)	5 (83)<br>(36, 100)	14 (100)<br>(77, 100)
Confirmed best response,[a] n (%)							
CR	1 (9)	2 (8)	1 (8)	0	3 (10)	1 (17)	1 (7)
PR	4 (36)	12 (48)	6 (50)	5 (42)	15 (52)	3 (50)	5 (36)
SD	5 (45)	10 (40)	4 (33)	7 (58)	9 (31)	1 (17)	8 (57)
PD	1 (9)	1 (4)	0	0	1 (3)	0	0
NE	0	0	0	0	0	0	0
NA	0	0	1 (8)	0	1 (3)	1 (17)	0
Median DOR, months (95% CI)	6.24<br>(2.79, NE)	5.60<br>(2.83, 12.45)	7.13<br>(3.55, 15.21)	4.86<br>(2.76, NE)	6.24<br>(3.55, 12.65)	7.1<br>(2.83, NE)	9.0<br>(4.86, NE)
Median PFS, months (95% CI)	6.97<br>(2.00, 13.80)	5.45<br>(3.98, 8.08)	6.77<br>(2.60, 16.36)	5.93<br>(2.86, 9.46)	6.77<br>(4.01, 8.08)	4.12<br>(1.25, NE)	8.15<br>(5.65, 16.36)
Median OS, months (95% CI)	15.15<br>(3.84, 17.61)	14.26<br>(7.75, 17.31)	13.27<br>(3.52, NE)	10.32<br>(5.59, NE)	12.42<br>(7.75, 17.61)	10.51<br>(1.25, NE)	17.31<br>(8.21, NE)
OS estimate at 15-months (95% CI)	0.51<br>(0.19, 0.76)	0.47<br>(0.26, 0.66)	0.46<br>(0.16, 0.72)	0.48<br>(0.16, 0.75)	0.42<br>(0.23, 0.59)	0.33<br>(0.05, 0.68)	0.71<br>(0.41, 0.88)

[a]Investigator-assessed confirmed CR + PR. [b]Confirmed CR + PR + SD.
CI, confidence interval; CR, complete response; CTFI, chemotherapy free interval; DOR, duration of response; NA, not available; NE, not estimable; ORR, objective response rate; OS, overall survival; PFS, progression-free survival; PD, progressive disease; PR, partial response; SCLC small cell lung cancer; SD, stable disease; Top1i, topoisomerase 1 inhibitor.

Supplementary Table S3. List of institutional review boards and ethical committees.

Location	Approval Board	Type
University of Utah - Huntsman Cancer Hospital - Myeloproliferative Disorders C	Advarra	Central IRB/IEC
Yale School of Medicine - Smilow Cancer Hospital - Yale-New Haven - Prostate and Urologic Cancers Program	Advarra	Central IRB/IEC
University Hospitals Cleveland Medical Center	University of Utah Healthcare	Local IRB/IEC
University Hospitals Cleveland Medical Center	University Hospitals Cleveland Medical Center IRB	Local IRB/IEC
MD Anderson Cancer Center - Mendelsohn Faculty Center Thoracic/Head & Neck Medical Oncology	WCG IRB	Central IRB/IEC
MD Anderson Cancer Center - Mendelsohn Faculty Center Thoracic/Head & Neck Medical Oncology	The University of Texas - MD Anderson Cancer Center - Surgery	Local IRB/IEC
Tennessee Oncology PLLC: Sarah /ID# 171380	Advarra	Central IRB/IEC
University of Iowa Health	Advarra	Central IRB/IEC
South Texas Accelerated Research Therapeutics, LLC	Salus IRB	Local IRB/IEC
University of Oklahoma/Sarah Cannon Research Institute	Advarra	Central IRB/IEC
Henry Ford Hospital - Hematology/Oncology	WCG IRB	Local IRB/IEC
Memorial Sloan Kettering - I Chemotherapy Practice/Investigational Drug Service	Memorial Sloan Kettering Cancer Center	Local IRB/IEC
START Midwest, LLC	Salus IRB	Central IRB/IEC
Duke University Medical Center, Children'S Health Center	Duke University Health System Institutional Review Board	Local IRB/IEC
Washington University School of Medicine-Center for Advanced	Advarra	Central IRB/IEC
Washington University School of Medicine-Center for Advanced	Washington University Human Research Protection Office (Hrpo	Local IRB/IEC
The University of Chicago Medical Center (UCMC) - Duchossois Center for Advanced Medicine (DCAM) - Hyde Park	University of Chicago - Nephrology	Local IRB/IEC
The University of Chicago Medical Center (UCMC) - Duchossois Center for Advanced Medicine (DCAM) - Hyde Park	BSD IRB, The University of Chicago Biological Sciences Division/The University of Chicago Medical Center	Local IRB/IEC
Georgetown University Medical Center	University of Chicago - Nephrology	Local IRB/IEC

Georgetown University Medical Center	Advarra	Central IRB/IEC
City of Hope at Irvine Lennar	WCG IRB	Local IRB/IEC
Banner MD Anderson Cancer Center	Advarra	Central IRB/IEC
Fort Wayne Medical Oncology and Hematology	Advarra	Central IRB/IEC
Saint Luke's Cancer Specialists	Saint Luke's Health System IRB	Local IRB/IEC
Karmanos Cancer Institute - Oncology	Advarra	Central IRB/IEC
Northwest Medical Specialties, PLLC	Northwest Medical Specialties, PLLC	Central IRB/IEC
Northwest Medical Specialties, PLLC	Advarra	Central IRB/IEC
City of Hope	WCG IRB	Local IRB/IEC
Samsung Medical Center - Hematology & Oncology	Samsung Medical Center- IRB	Local IRB/IEC
Severance Hospital, Yonsei University Health System - Pediatric Endocrinology	Severance Hospital, Yonsei University Health System IRB	Local IRB/IEC
Seoul National University Hospital	Seoul National University Hospital - Ophthalmology	Local IRB/IEC
Seoul National University Hospital	IRB of Seoul National University Hospital	Local IRB/IEC
CHA Bundang Medical Center, CHA University	CHA Bundang Medical Center	Local IRB/IEC
Chonnam National University Hwasun Hospital - Lung cancer clinic	Chonnam National University Hwasun Hospital - Neurology	Local IRB/IEC
National Cancer Center	National Cancer Center - Center for pediatric cancer	Local IRB/IEC
National Cancer Center	National Cancer Center	Local IRB/IEC
Kyoto University Hospital - Oncology	Kyoto University Hospital - IRB/IEC	Local IRB/IEC
National Cancer Center Hospital	National Cancer Center Hospital - Local IRB/EC	Local IRB/IEC
Wakayama Medical University, Second Department of Surgery	Wakayama Medical University, Second Department of Surgery	Local IRB/IEC
Wakayama Medical University, Second Department of Surgery	Wakayama Medical University - Institutional Review Board - Institutional Review Board	Local IRB/IEC
National Cancer Center Hospital East	National Cancer Center Hospital - Local IRB/EC	Local IRB/IEC
Shizuoka Cancer Center - Respiratory Tract Medicine	Shizuoka Cancer Center - Institutional Review Board	Local IRB/IEC
National Hospital Organization - Hokkaido Cancer Center - Respiratory Medicine	National Hospital Organization Hokkaido Cancer Center - IRB/IEC	Local IRB/IEC
National Hospital Organization Shikoku Cancer Center - Respiratory Medicine	Shikoku Cancer Center - Irb	Local IRB/IEC

The Cancer Institute Hospital of JFCR	The Cancer Institute Hospital of JFCR - IRB/IEC	Local IRB/IEC
Chaim Sheba Medical Center; Rheumatology	Chaim Sheba Medical Center	Local IRB/IEC
Chaim Sheba Medical Center; Rheumatology	Helsinki Committee of the Chaim Sheba Medical Center	Local IRB/IEC
Rambam Health Care Campus - Ophthalmology	Rambam Health Care Campus - Pulmonology	Local IRB/IEC
Rambam Health Care Campus - Ophthalmology	Helsinki Committee of Rambam Health Care Campus	Local IRB/IEC
Hadassah Medical Center	Hadassah Medical Organization, Hadassah Medical Center, Ein-Karem - Ophtalmology	Local IRB/IEC
Hadassah Medical Center	Helsinki Committee (IRB) of Hadassah Medical Organization	Local IRB/IEC
Austin Hospital - Olivia Newton John Cancer Research Institute	Peter MacCallum Cancer Centre HREC	Central IRB/IEC
Chris O'Brien Lifehouse	Bellberry Limited	Central IRB/IEC
Peter MacCallum Cancer Centre - Haematology & Medical Oncology	Peter MacCallum Cancer Centre HREC	Central IRB/IEC
St Vincent’s Hospital - Kinghorn Cancer Centre	Bellberry Limited	Central IRB/IEC
Hospital Universitario HM Sanchinarro	Corporació Sanitaria Parc Taulí; Servicio de Neurología	Central IRB/IEC
Hospital Universitario 12 de Octubre - Oncología Médica	Corporació Sanitaria Parc Taulí; Servicio de Neurología	Central IRB/IEC
Hospital Universitario Vall d'Hebron - Oncología Médica	Corporació Sanitaria Parc Taulí; Servicio de Neurología	Central IRB/IEC
University Hospital Virgen del Rocío S.L. - Oncología	Corporació Sanitaria Parc Taulí; Servicio de Neurología	Central IRB/IEC
Hospital Clinico Universitario De Valencia - Oncologia Médica y Hematologia	Corporació Sanitaria Parc Taulí; Servicio de Neurología	Central IRB/IEC
Hospital Universitario Ramon y Cajal - Oncología Médica	Corporació Sanitaria Parc Taulí; Servicio de Neurología	Central IRB/IEC
H.U.M. de Terrassa - Oncologia	CEIC Hospital Vall d Hebron	Central IRB/IEC
H.U.M. de Terrassa - Oncologia	Corporació Sanitaria Parc Taulí; Servicio de Neurología	Central IRB/IEC
Hospital de la Santa Creu i Sant Pau - Oncología Médica	CEIC Hospital Vall d Hebron	Central IRB/IEC
Fundacion Instituto Valenciano de Oncologia - Oncology	Corporació Sanitaria Parc Taulí; Servicio de Neurología	Central IRB/IEC
Hospital Universitario Fundacion Jimenez Diaz	CEIC Hospital Vall d Hebron	Central IRB/IEC
START Hospital HM Nou Delfos	Corporació Sanitaria Parc Taulí; Servicio de Neurología	Central IRB/IEC
Institut Bergonie - Oncologie Medicale	CPP SE IV Centre Léon Bérard	Central IRB/IEC
Institut Gustave Roussy	CPP SE IV Centre Léon Bérard	Central IRB/IEC
ICM Val d'Aurelle - Oncologie Médicale - Oncologie Medicale	CPP SE IV Centre Léon Bérard	Central IRB/IEC
Shanghai East Hospital	Shanghai East Hospital	Local IRB/IEC

Shanghai Chest Hospital - Pneumology	Shanghai Chest Hospital	Local IRB/IEC
The First Hospital of Chinese Medical University(Hunnan Compus) - Oncology	The First Hospital of China Medical University	Local IRB/IEC
The First Hospital of Chinese Medical University(Hunnan Compus) - Oncology	China Medical University - First Affiliated Hospital	Local IRB/IEC
Cancer Hospital Chinese Academy of Medical Sciences	Cancer Hospital Chinese Academy of Medical Sciences	Local IRB/IEC
Union Hospital Tongji Medical College Huazhong University of Science and Technology - Oncology	Ethics Committee, Union Hospital, Tongji Medical College, Huazhong University of Science and Technology	Local IRB/IEC
Shanghai Pulmonary Hospital - Medical Oncology	Shanghai Pulmonary Hospital	Local IRB/IEC

Supplementary Protocol

1) Summary of protocol amendments
2) Final protocol

Protocol Amendment Summary of Changes Table

DOCUMENT HISTORY			
**Document**	**Amendment Scope; Region/Country Identifier (if applicable)**	**Protocol Version**	**Date**
Amendment 1	Global	2.0	07-October-2022
Original Protocol	Not Applicable	1.0	12-August-2022

Amendment 1 (10-October-2022)

This amendment is considered to be substantial based on the criteria set forth in Article 10(a) of Directive 2001/20/EC of the European Parliament and the Council of the EU.

This modification is considered to be substantial based on the criteria set forth in European Regulation 536/2014 Article 2(2)(13).

Overall Rationale for the Amendment:

The purpose of this version is to correct minor clerical errors for consistency throughout the protocol in addition to addressing the FDA's requests by updating the following:

- Moved the evaluation of clinical benefit, stable disease, progression-free survival, and overall survival from primary to exploratory objectives for subjects with R/R SCLC, high-grade CNS tumors and NECs (Protocol Section 1.1 and Protocol Section 3).
- Clarified that the safety Monitoring Committee will also make starting dose and dose escalation recommendations for Parts 3a and 3b (Protocol Section 1.2 and Protocol Section 4.1.6).
- Clarified in Part 1 that subjects with high-grade CNS tumors must have had prior surgical resection, received prior radiation, and temozolomide or PCV and subjects with NECs must have also received at least one prior line of standard-of-care systemic chemotherapy (Protocol Section 1.3 and Protocol Section 5.1, Eligibility Criterion #11).
- Clarified in Part 3a and Part 3b that subjects with R/R NECs must have progressed on at least one line of standard-of-care systemic chemotherapy (Protocol Section 1.3 and Protocol Section 5.1, Eligibility Criteria #13 and #14, respectively).
- Clarified in Part 4a that subjects with R/R high-grade CNS tumors must have had prior surgical resection, received prior radiation, and temozolomide or PCV (Protocol Section 1.3 and Protocol Section 5.1, Eligibility Criterion #15).

- Clarified in Part 4b that subjects with R/R NECs must have had prior line of standard-of-care systemic chemotherapy (Protocol Section 1.3 and Protocol Section 5.1, Eligibility Criterion #16).
- Removed duplicate Eligibility Criteria #17 through #20 and renumbered remaining Eligibility Criteria accordingly (Protocol Section 5.1, Eligibility Criterion #16).
- Added cautionary use of strong inducers of cytochrome P450 to allowed medications (Protocol Section 5.1, Eligibility Criterion #18; Protocol Section 6.11.1; Protocol Section 10.6, Appendix 6).
- Clarified the ABBV-706 toxicity management of Grade 3 and Grade 4 non-hematologic toxicities (Protocol Section 6.7).
- Updated definitions of dose-limiting toxicities (Protocol Section 8.2).
- Changed the AE and SAE collection period for Part 3a to 90 days (Protocol Section 8.5.1, Protocol Section 1.5, Table 1.1 footnote 'g' and Table 1.2 footnote 'i').
- Added that high-risk biopsies near any vital neurovascular structures should be avoided (Protocol Section 8.9).
- Added that budigalimab should be permanently discontinued for ≥ Grade 2 myocarditis and ≥ Grade 3 ocular toxicity (Protocol Section 10.8.1).
- Added that for any grade transverse myelitis and for ≥Grade 2 Guillain-Barre Syndrome, study treatment should be discontinued (Protocol Section 10.8.12).
- Added Day 2 to Cycle 2 to collected AEs, concomitant therapy, triplicate ECG, vital signs, and PK samples (Protocol Section 1.5, Table 1.1 and Table 1.2).
- Clarified that neurological exams are to be done whenever a physical exam is done (Protocol Section 1.5, Table 1.1 and Table 1.2).
- Added collection timepoints for PK and triplicate ECGs on Days 1 and 2 of Cycle 2 (Parts 1 and 2), PK on Days 1, 2 and 8 of Cycle 1 (Parts 3 and 4) and clarified collection for budigalimab ADA/nADA assessments (Part 3a) (Section 8.1, Table 8.1).

Protocol Amendment Summary of Changes Table

DOCUMENT HISTORY			
**Document**	**Amendment Scope; Region/Country Identifier (if applicable)**	**Protocol version**	**Date**
Amendment 2	Global	3.0	15-Jun-2023
Amendment 1	Global	2.0	07-October-2022
Original Protocol	Not Applicable	1.0	12-August-2022

Amendment 2 (15-Jun-2023)

This amendment is considered to be substantial based on the criteria set forth in Article 10(a) of Directive 2001/20/EC of the European Parliament and the Council of the European Union.

This modification is considered to be substantial based on the criteria set forth in European Regulation 536/2014 Article 2(2)(13).

Overall Rationale for the Amendment:

The purpose of this version is to add text aligned with the EU CTR guidelines, to implement changes described in response commitment letter to the Korean Health Authority, Ministry of food and drug safety, and other minor editorial changes for consistency throughout the protocol.

Section # and Name	Description of Change	Brief Rationale
**Section 1.1:** Synopsis	Changes made throughout the synopsis	To reflect changes made within the protocol
**Section 1.2:** Schema-Figure 1-2	Updated figure for accuracy	Updated figure for accuracy
**Section 1.3:** Schedule of Activities (SoA)<br>**Section 6.3**: Assignment of Study Intervention	Updated table to remove pre-screening for SEZ6 (if elected by Sponsor).	To comply with EU in vitro diagnostic medical device regulation (IVDR) requirement.
**Section 4.1.1 Part 1:** (Monotherapy Dose Escalation)	Removal of information related to SEZ6 option of implementing prospective selection.	To comply with EU IVDR requirement.
**Section 4.1.2 Part 2:** (Monotherapy Dose Optimization and Expansion in R/R SCLC)	Removal of information related to SEZ6 option of implementing prospective selection.	To comply with EU IVDR requirement.

Section # and Name	Description of Change	Brief Rationale
**Section 4.1.3.2**: Part 3b (ABBV-706 and Platinum Chemotherapy Combination Dose Escalation and Expansion)	Added text "ABBV-706 in combination with carboplatin and ABBV-706 in combination with cisplatin and will proceed independently of each other."	Per response commitment letter to Korean Health Authority, Ministry of food and drug safety updated to specify the separate dose escalation plan for carboplatin and cisplatin.
**Section 4.1.7**: Backfill Guidelines	Deleted text "The secondary escalation in subjects with CNS tumors can be opened at least one dose level below the primary escalation MTD or the highest dose that has met BOIN escalation criteria with at least 3 DLT evaluable subjects (e.g., 0 out of 3 DLTs) and will then proceed independent of the primary escalation."	For clarification.
**Section 4.4**: End-of-Study Definition	Added definition for participant's study completion.	Added for EU CTR compliance.
**Section 5.1**: Eligibility Criteria for Parts 1, 2, 3, and 4- Contraception	Added the following text "(or according to local label for carboplatin or cisplatin, whichever is longer, for Part 3b carboplatin or cisplatin combination subjects; Korea only: through at least 11 or 14 months after the last dose of cisplatin or carboplatin)"	Per response commitment letter to Korean Health Authority, Ministry of food and drug safety updated to include language to follow the contraceptive period in the local carboplatin or cisplatin label
**Section 5.1**: Eligibility Criteria for Parts 1, 2, 3, and 4- Demographic and Laboratory Assessments	Added: "For subjects with liver metastases: ALT and AST ≤ 5.0 × ULN"	Criteria refined to accommodate subjects with liver metastasis
**Section 5.1**: Eligibility Criteria 10	Removal of information related to SEZ6 option of implementing prospective selection.	To comply with EU IVDR requirement.
**Section 5.1**: Eligibility Criteria 11 and 12	Restructuring of eligibility criteria 11 and 12	To improve readability and clarify that Grade 3 NETs and medullary thyroid cancer are allowed

Section # and Name	Description of Change	Brief Rationale
**Section 5.4** Screen Failure<br>**Section 8.1.1** Study Subject Informed Consent<br>**Section 8.1.7** Archival or Fresh Tumor Biopsy<br>**Section 8.5.1** Time Period and Frequency for Collecting AE and SAE Information.<br>**Section 8.9** Biomarkers<br>**Section 9.6 9.6** Sample Size Determination	Deleted text related to SEZ6 option of implementing prospective selection	To comply with EU IVDR requirement
**Section 6.1, Table 6-1:** Study Intervention(s) Administered	Added IMP strength to the Table 6-1.<br>Updated condition of study drug administration "Platelets ≥ 75 × 109 /L (no transfusions within 5 days prior to dose)"	Clarification to include IMP strength.<br><br>Clarification added per study requirement
**Section 6.7**: Management of Drug-Related Toxicities	Added for Part 3a combination, "dose reductions for carboplatin and cisplatin based on renal function should follow local/institutional guidelines."	To specify the dose reduction plan based on renal function for carboplatin and cisplatin per response commitment letter to the Korean Health Authority, Ministry of food and drug safety.
**Section 6.7.4**: Infusion-Related Reactions (IRRs)	• Deleted "In situations where an IRR occurs prior to the end of infusion, ABBV-706 infusion should be immediately interrupted."<br>• Reflexive testing following IRRs was changed from required to recommended.	• For consistency with Table 6-6.<br>• To allow more flexibility for non-essential tests.
**Section 6.10:** Prior And Concomitant Medications Review	Updated timeline for review of prior and concomitant medication to start from "signature of consent through 80 days (for Parts 1, 2, 3b, and 4) or 90 days (for Part 3a) after last dose of study drug"	Changed per study requirement
**Section 8.4.3** Vital Signs	Added time window for vital sign assessment "(±10 minutes)" after the start of infusion	To refine study assessments
**Section 8.6.1** Blood Sample Collection	Amended text on PK blood sample collection	For clarity in PK blood sample collection
**Section 8.6.1** Blood Sample Collection Table 8-1	Sampling Schedule updated for End of Treatment of ABBV-706 PK and ADA Assessments	Changes for consistency with SoA

Section # and Name	Description of Change	Brief Rationale
**Section 8.12** Storage and Future Use of Biological Samples	Added text "AbbVie will be responsible for ensuring all biological sample collection and storage is secure and with adequate measures to protect confidentiality, per all applicable local regulations. AbbVie will store biological samples related to plasma, serum, or tumor tissue samples collected as part of the PK/PD/biomarker/immunogenicity assessments. With participants' consent, samples may be used for further research by AbbVie, per all applicable local regulations."	Added section per EU CTR requirement.
**Section 10.3.1** Definition of AE	Added text "Events of disease progression related to the primary malignancy being evaluated in this study and accompanying symptoms, if clearly associated with disease progression, will not be collected as adverse events unless the event(s) result in death or qualifies as a serious adverse event as defined in Section 10.3.2 and require reporting in the same manner as any serious adverse event"	For clarity

Section # and Name	Description of Change	Brief Rationale
**Section 10.3.2** Definition of SAE	• Modified text "All AEs and SAEs reported from the time of study drug administration ~~until~~ 80 days (5 half-lives ~~(80 days~~of ABBV-706) after ~~discontinuation~~last dose of study drug ~~administration~~ (for Parts 1, 2, 3b, and 4) or 90 days after last dose of study drug (Part 3a) will be collected, whether solicited or spontaneously reported by the subject. In addition, study procedure-related serious and nonserious AEs will be collected from the time the subject signs the study-specific informed consent. until study drug administration. After 80 days (for Parts 1, 2, 3b, and 4) or 90 days (for Part 3a) following completion of study treatment and throughout the post-treatment period, all spontaneously reported SAEs will be collected (nonserious AEs will not be collected)."<br>• Added "Suspected unexpected serious adverse reactions reporting will be in adherence to requirements of EU pharmacovigilance legislation, clinical trial legislation and guidance. EU CTR 536/2014; CT-3 and all other applicable local regulations."	• For addition of study-specific information.<br>• Per EU CTR requirement.
**Section 10.3.3** Recording and Follow-up of AE and SAE	Deleted the following "The following "binary" decision choice will be used by the investigator to describe the initial causality assessment:<br>• Related: Reasonable possibility of a relatedness.<br>• Not Related: No reasonable possibility of relatedness"	Per study requirement
**Section 10.3.4** Reporting Adverse Events and Intercurrent Illnesses	Reporting instruction and hotline details were updated	Added based on EU CTR requirement

Section # and Name	Description of Change	Brief Rationale
**Section 10.4** Appendix 4: Contraceptive and Barrier Guidance	Added underlined text "Contraception recommendations related of use of carboplatin or cisplatin in Part 3b or concomitant therapies prescribed should be based on the local label and contraception period should be based on whichever is longer considering all administered therapies. In Korea, for subjects treated with the cisplatin or carboplatin combination, female subjects of childbearing potential must practice an effective method of birth control during the treatment period of cisplatin or carboplatin and 14 months after the last dose of cisplatin or carboplatin; male subjects must practice an effective method of birth control during the treatment period of cisplatin or carboplatin and 11 months after the last dose of cisplatin or carboplatin."	To reflect the changes in Section 5.1
**Section 10.8:** Appendix 8: Part 3a Budigalimab Combination Immune-Related Adverse Event Management Guidance	All Tables had minor edits to remove "NCI CTCAE v5.0" from the header.	As the guidelines in this appendix were developed from the American Society of Clinical Oncology (ASCO) irAE management guidelines
**Section 10.11:** Appendix 11: Country Specific Requirement	Added this Appendix to record any country specific changes to the protocol in future	Per template guidance
**Section 10.12:** Appendix 12: Protocol Amendment History	Moved last protocol amendment details to Appendix	Per template guidance
Throughout the protocol	The terminology of "the NECs group" had been revised to "high-grade NECs/NETs	To more clearly capture the target population, and also be more clear that medullary thyroid cancer and Grade 3 NETs are allowed
Throughout the protocol.	Minor editorial corrections were made.	For clarity and consistency throughout the document.

Protocol Amendment Summary of Changes Table

DOCUMENT HISTORY			
**Document**	**Amendment Scope; Region/Country Identifier (if applicable)**	**Protocol version**	**Date**
Amendment 3	Japan Only	3.1	31-July-2023
Amendment 2	Global	3.0	26-May-2023
Amendment 1	Global	2.0	07-October-2022
Original Protocol	Not Applicable	1.0	12-August-2022

Amendment 3 (31-July-2023)

This amendment is considered to be substantial based on the criteria set forth in Article 10(a) of Directive 2001/20/EC of the European Parliament and the Council of the European Union.

This modification is considered to be substantial based on the criteria set forth in European Regulation 536/2014 Article 2(2)(13).

Overall Rationale for the Amendment:

The purpose of this version is to add text for a Japan only cohort during Part 1 and other minor editorial changes for consistency throughout the protocol.

Section # and Name	Description of Change	Brief Rationale
**Section 1.1 Synopsis**	Added information about Japan Cohort	To accurately summarize Japan Cohort
**Section 1.2: Schema-Figure 1-2**	Added figure for Japan Cohort	Added figure for Japan Cohort
**Section 1.3: Schema-Figure 1-3**	Changed figure to include Japan Cohort	Changed figure to include Japan Cohort
**Section 1.3: Schedule of Activities (SoA)**	Added requirements and footnotes for Japan Cohort	To describe the Japan Cohort activities requirements
**Section 4.1.1 Part 1**	Split Part 1 into Part 1a (Global) and Part 1b (Japan only)	To reflect the different designs for the Monotherapy Dose Escalation phases for the Global Cohort and the Japan Cohort
**Section 4.1.1.2 Part 1b**	Added section for Japan Cohort	To describe the Japan Cohort
**Section 4.1.5: Dose Escalation Guidelines**	Added section for Japan Cohort	To describe Dose Escalation for the Japan Cohort
**Section 4.2.2: Suitability of Subject Population**	Changed subject population to include the Japan Cohort	To accurately represent subject numbers

Section # and Name	Description of Change	Brief Rationale
**Section 5.1: Subject History**	Added section for Japan Cohort: Subjects in Japan Cohort must not have any history of ILD	To describe subject history eligibility criteria for the Japan Cohort
**Section 8.2: Dose-Limiting Toxicity Assessment**	Clarifying statement added for combination therapies meeting DLT criteria	To clarify that toxicities related to combination drugs meeting DLT criteria will be counted as DLTs
**Section 8.4: Safety Assessments**	Added section for Japan Cohort	To describe safety evaluations for the Japan Cohort
**Section 8.4.1: Physical Examination**	Added section for Japan Cohort	To describe SpO_2 evaluation requirements for the Japan Cohort
**Section 8.4.5.1: Virology Testing (Japan only)**	Added section for Japan Cohort	To describe virology testing requirements for the Japan Cohort
**Section 8.9: Biomarkers**	Added section for Japan Cohort stating biomarker research results will not be disclosed to subjects in the Japan Cohort	To describe disclosure of biomarker research results for the Japan Cohort
**Section 9.3.3: Safety Analyses**	Clarified treatment-emergent period	To accurately define the treatment-emergent period for all parts of the study
**Section 9.6: Sample Size Determination**	Changed total number of subjects to include the Japan Cohort	To accurately represent subject numbers
**Appendix 2: Table 10–1**	Added KL-6 and Hemoglobin A1C (HbA1C) testing requirements for the Japan Cohort	To add lab testing requirements for the Japan Cohort
**Appendix 2: Virology Testing (Japan only)**	Added section for Japan Cohort	To describe virology testing requirements for the Japan Cohort
**Appendix 4: Contraception Requirement for Part 1b (Japan)**	Added list of contraception methods that are not approved or certified in Japan	To describe which methods of contraception are not approved or certified in Japan
**Appendix 11: Japan Specific Requirements**	Added supplemental information for Japan Sites on scientific findings on drugs, handling of drugs used in the clinical trial, and reporting of serious adverse event and adverse event of special interest	To provide additional information to Japan sites as needed for the Japan specific regulatory requirements of the revision of Order for Enforcement of the Act on Securing Quality, Efficacy and Safety of Products Including Pharmaceuticals and Medical Devices in Japan
Throughout the protocol.	Minor editorial corrections were made.	For clarity and consistency throughout the document.

Protocol Amendment Summary of Changes Table

DOCUMENT HISTORY			
**Document**	**Amendment Scope; Region/Country Identifier (if applicable)**	**Protocol version**	**Date**
Amendment 4	Japan Only	3.1.1	25 September 2023
Amendment 3	Japan Only	3.1	31 July 2023
Amendment 2	Global	3.0	26 May 2023
Amendment 1	Global	2.0	07 October 2022
Original Protocol	Not Applicable	1.0	12 August 2022

Amendment 4 (25 September 2023)

This amendment is considered to be substantial based on the criteria set forth in Article 10(a) of Directive 2001/20/EC of the European Parliament and the Council of the European Union.

This modification is considered to be substantial based on the criteria set forth in European Regulation 536/2014 Article 2(2)(13).

Overall Rationale for the Amendment:

The purpose of this version is to add Japan-specific eligibility criteria to the protocol, to clarify eligibility criteria for subjects enrolling in Part 1b of the study, and to provide additional information about blood volumes for biomarker assessments.

Section # and Name	Description of Change	Brief Rationale
**Section 1.1 Synopsis**	Additional eligibility conditions added for subjects enrolling in Part 1b of the study (Eligibility Criterion #12).	To specify previous treatment regimens subjects must have had before enrollment in Part 1b of the study.
**Section 5.1 Eligibility Criteria for Parts 1, 2, 3, and 4**	Additional eligibility conditions added for subjects enrolling in Part 1b of the study (Eligibility Criterion #12).	To specify previous treatment regimens subjects must have had before enrollment in Part 1b of the study.
	Additional eligibility conditions for female subjects of childbearing potential in Japan (Eligibility Criterion #25).	To clarify eligibility criteria for female subjects of childbearing potential in Japan.

Section # and Name	Description of Change	Brief Rationale
**Section 5.2 Additional Exclusion Criteria for Part 3a (Budigalimab Combination)**	Added Japan-specific criterion that subjects may not have a history of hypersensitivity to the active ingredients or any excipients of ABBV-181 (Eligibility Criterion #35).	Additional Japan-specifc eligibility criterion.
**Section 8.9 Biomarkers**	Included information about where required blood volumes for biomarker assessments will be specified.	To clarify where information about required blood volumes for biomarker assessments can be found.
Throughout the protocol.	Minor editorial corrections were made.	For clarity and consistency throughout the document.

Protocol Amendment Summary of Changes Table

DOCUMENT HISTORY			
**Document**	**Amendment Scope; Region/Country Identifier (if applicable)**	**Protocol version**	**Date**
Amendment 5	EU Only	3.2	24-October-2023
Amendment 4	Japan Only	3.1.1	25-September-2023
Amendment 3	Japan Only	3.1	31-July-2023
Amendment 2	Global	3.0	15-Jun-2023
Amendment 1	Global	2.0	07-October-2022
Original Protocol	Not Applicable	1.0	12-August-2022

Amendment 5 (24-Oct-2023)

Overall Rationale for the Amendment:

The purpose of this version is to change the reference to the study EU CT Number.

Section # and Name	Description of Change	Brief Rationale
**Title page**	Updated reference from "EudraCT" to "EU CT"	Per EMA request

Protocol Amendment Summary of Changes Table

DOCUMENT HISTORY			
**Document**	**Amendment Scope; Region/Country Identifier (if applicable)**	**Protocol version**	**Date**
Amendment 6	EU Only	3.2.1	08-January-2024
Amendment 5	EU Only	3.2	24-October-2023
Amendment 4	Japan Only	3.1.1	25-September-2023
Amendment 3	Japan Only	3.1	31-July-2023
Amendment 2	Global	3.0	15-Jun-2023
Amendment 1	Global	2.0	07-October-2022
Original Protocol	Not Applicable	1.0	12-August-2022

Amendment 6 (08-Jan-2024)

This amendment is considered to be substantial based on the criteria set forth in Article 10(a) of Directive 2001/20/EC of the European Parliament and the Council of the European Union.

This modification is considered to be substantial based on the criteria set forth in European Regulation 536/2014 Article 2(2)(13).

Overall Rationale for the Amendment:

The purpose of this version is to update the protocol to address items raised in an RFI from the EMA and to make other minor editorial changes for consistency throughout the protocol.

Section # and Name	Description of Change	Brief Rationale
**Section 1.1: Synopsis – Key Eligibility Criteria**	• Updated criterion to clarify creatinine clearance required for subjects in parts 1, 2, 3a, 3b, and 4<br>• Updated criterion to include additional considerations for tumor biopsies in cases where they may pose a risk to subjects<br>• Updated criterion to clarify tumor types considered as high-grade NECs/NETs<br>• Updated criterion to remove allowance for subjects who have refused SoC therapy to be included in the study; clarified that for eligible NECs and NETs, subjects must have received and/or considered all available approved therapeutic options, if applicable, prior to enrolling on the study	Address RFI from the EMA.
**Section 1.3: Schedule of Activities**	Updated Table 1-2 as follows:<br>• Added periodic testing for coagulation parameters (aPTT/PT/INR) at an interval of every 4 cycles in addition to at screening<br>• Added amylase and lipase testing at screening; added footnote describing conditions for additional testing during the study as well as additional procedures if levels are > 3x ULN	Address RFI from the EMA.
**Section 2.3: Summary of Findings From Nonclinical Studies**	Updated to include additional pharmacology and toxicology nonclinical data for ABBV-706.	Address RFI from the EMA.
**Section 2.4: Benefit/Risk Assessment**	Updated to include additional information from ongoing clinical studies to support the use of budigalimab as monotherapy and in combination with other agents.	Address RFI from the EMA.

Section # and Name	Description of Change	Brief Rationale
**Section 4.1.3.1: Part 3a (ABBV-706 and Budigalimab Combination Dose Escalation and Expansion)**	Clarified that dose for part 3a will be at or below the highest dose that clears the DLT evaluation in combination escalation and will not exceed the highest monotherapy dose selected for Part 2a dose optimization.	Address RFI from the EMA.
**Section 4.1.3.2: Part 3b (ABBV-706 and Platinum Chemotherapy Combination Dose Escalation and Expansion)**	Clarified that dose for part 3b will be at or below the highest dose that clears the DLT evaluation in combination escalation and will not exceed the highest monotherapy dose selected for Part 2a dose optimization.	Address RFI from the EMA.
**Section 4.1.5: Dose Escalation Guidelines**	Updated definition of DLT evaluable subject.	Address RFI from the EMA.
**Section 4.1.9: Dose Expansion and Safety Rules**	Clarified that dose expansion safety rules apply to dose optimization, expansion cohorts, and backfill cohorts.	Address RFI from the EMA.

Section # and Name	Description of Change	Brief Rationale
**Section 5.1: Eligibility Criteria for Parts 1, 2, 3, and 4**	• Updated criterion #5 to clarify creatinine clearance required for subjects in parts 1, 2, 3a, 3b, and 4<br>• Updated criterion #10 to include additional considerations for tumor biopsies in cases where they may pose a risk to subjects<br>• Updated criterion #11 to clarify tumor types considered as high-grade NECs/NETs<br>• Updated criterion #12 to remove allowance for subjects who have refused SoC therapy to be included in the study; clarified that for eligible NECs and NETs, subjects must have received and/or considered all available approved therapeutic options, if applicable, prior to enrolling on the study<br>• Added criterion # 21 to require that subjects in part 3b not have a history of hypersensitivity to the active ingredients or any excipients of cisplatin or carboplatin and/or contraindication to cisplatin or carboplatin<br>• Updated criterion #21 (now shown as #22) to define meaning of cured hepatitis B<br>• Updated criterion #24 (now shown as #25) to clarify that acceptable birth control methods are *highly* effective	Address RFI from the EMA.
**Section 6.7.3: Hepatotoxicity**	• Updated Table 6-5 to specify that ABBV-706 is to be permanently discontinued if recurrence of Grade 3 toxicity occurs.<br>• Added footnote to indicate that ABBV-706 is to be permanently discontinued in cases of confirmed DILI	Address RFI from the EMA.

Section # and Name	Description of Change	Brief Rationale
**Section 6.7.5: Peripheral Neuropathy, Section 6.7.6: Dermatologic Toxicity**	Updated Tables 6-7 and 6-8 to specify that ABBV-706 is to be permanently discontinued if recurrence of Grade 3 toxicity occurs.	Address RFI from the EMA.
**Section 6.7.6: Dermatologic Toxicity**	Footnote added to Table 6-8 to specify that ABBV-706 treatment should be held for suspected TEN/SJS and permanently discontinued for confirmed TEN/SJS.	Address RFI from the EMA.
**Section 8.1.7: Archival or Fresh Tumor Biopsy**	Clarified conditions where tumor biopsies may not be available.	Address RFI from the EMA.
**Section 8.2: Dose-Limiting Toxicity Assessment**	Updated to clarify that the DLT observation period for part 3 escalation and also that the DLT are applicable for part 3a and 3b.	Address RFI from the EMA.
**Section 8.9: Biomarkers**	Included additional considerations for tumor biopsies in cases where they may pose a risk to subjects.	Address RFI from the EMA.
**Section 9.4.1: Futility Analysis**	Added Section 9.4.1 Futility Analysis to the protocol.	Address RFI from the EMA.
**Section 10.2: Appendix 2: Clinical Laboratory Tests**	Updated to include amylase and lipase testing.	Address RFI from the EMA.
**Section 10.4: Appendix 4: Contraceptive and Barrier Guidance**	Updated to specify the use of ultra-low estrogen contraceptives.	Address RFI from the EMA.
Throughout the protocol	Updated wording to list out specific types of high-grade NEC and NET tumors.	Address RFI from the EMA.
Throughout the protocol	Minor editorial corrections were made.	For clarity and consistency throughout the document.

Protocol Amendment Summary of Changes Table

DOCUMENT HISTORY			
**Document**	**Amendment Scope; Region/Country Identifier (if applicable)**	**Protocol version**	**Date**
Amendment 7	China Only	3.3	18-June-2024
Amendment 6	EU Only	3.2.1	08-January-2024
Amendment 5	EU Only	3.2	24-October-2023
Amendment 4	Japan Only	3.1.1	25-September-2023
Admin Change	Global	2.0	05-Sept-2024
Amendment 3	Japan Only	3.1	31-July-2023
Admin Change	Global	1.0	11-July-2023
Amendment 2	Global	3.0	15-June-2023
Amendment 1	Global	2.0	07-October-2022
Original Protocol	Not Applicable	1.0	12-August-2022

Amendment 7 (18-Jun-2024)

Overall Rationale for the Amendment:

The purpose of this version is to add a China only cohort (Part 2c), China-specific requirements, and other minor editorial changes for consistency throughout the protocol.

Section # and Name	Description of Change	Brief Rationale
**Section 1.1:** Synopsis	Changes made throughout the synopsis	To reflect changes made within the protocol
**Section 1.2:** Schema-Figure 1–2	Updated figure for accuracy	Updated figure for accuracy
**Section 1.3:** Schedule of Activities (SoA)<br>**Section 8.4.5.1**: Virology Testing (China only)<br>**Section 10.2:** Appendix 2: Clinical Laboratory Tests	Add virology test requirements for subjects enrolled under Part 2c	To reflect specific requirements for sites in China
**Section 1.3:** Schedule of Activities (SoA)<br>**Section 8.9.1:** China Sites Only	Specify biomarker testing requirements for sites in China	To reflect specific requirements for sites in China
**Section 3:** Objectives and Endpoints	Additional exploratory endpoints described for sites in China	To reflect specific requirements for sites in China
**Section 4.1.2:** Part 2 (Monotherapy Dose Optimization and Expansion in R/R SCLC)	Description of Part 2c added	To describe new Part 2c cohort

Section # and Name	Description of Change	Brief Rationale
**Section 5.1**: Eligibility Criteria	Added specific eligibility criteria and edited existing criteria for sites in China	To reflect specific requirements for sites in China
**Section 6.11.2:** Prohibited	Added herbal supplements and/or traditional Chinese medicines to medicines prohibited during study treatment	To reflect specific requirements for sites in China
**Section 8.5.2:** Pregnancy<br>**Section 10.3.2:** Definition of SAE<br>**Section 10.3.4:** Reporting Adverse Events and Intercurrent Illnesses	Additions from Admin Change 2	To update document with changes made in Admin Change 2
**Section 10.2:** Appendix 2: Clinical Laboratory Tests	Added specific requirements and allowances for clinical lab tests at China sites	To reflect specific requirements for sites in China
**Section 10.11:** Appendix 11: Country Specific Requirement	Added China-specific requirements	To reflect specific requirements for sites in China
**Section 10.12:** Appendix 12: Protocol Amendment History	Moved last protocol amendment details to Appendix	Per template guidance
Throughout the protocol.	Minor editorial corrections were made	For clarity and consistency throughout the document

Protocol Amendment Summary of Changes Table

DOCUMENT HISTORY			
**Document**	**Amendment Scope; Region/Country Identifier (if applicable)**	**Protocol version**	**Date**
Amendment 8	China Only	3.3.1	02 December 2024
Amendment 7	China Only	3.3	18-June-2024
Amendment 6	EU Only	3.2.1	08-January-2024
Amendment 5	EU Only	3.2	24-October-2023
Amendment 4	Japan Only	3.1.1	25-September-2023
Admin Change	Global	2.0	05-Sept-2024
Amendment 3	Japan Only	3.1	31-July-2023
Admin Change	Global	1.0	11-July-2023
Amendment 2	Global	3.0	15-June-2023
Amendment 1	Global	2.0	07-October-2022
Original Protocol	Not Applicable	1.0	12-August-2022

Amendment 8 (02-December-2024)

Overall Rationale for the Amendment:

The purpose of this version is to modify the China only cohort (Part 2c) to expand dose escalation to two doses before expansion, and other minor editorial changes for consistency throughout the protocol.

Section # and Name	Description of Change	Brief Rationale
**Section 1.2:** Schema- Figure 1-2, Figure 1-3	Added a Part 2c figure	Added a Part 2c figure to illustrate the updated study design for Part 2c
**Section 4.1.2:** Part 2c (Monotherapy Safety Run-in and Dose Expansion for China)	Updated Part 2c language	Updated Part 2c language to specify the dose levels to be evaluated.
**Section** 4.3**:** Justification for Dose	Added new language	Added dosing language to specify the MTD.
**Section 10.11.1:** For Sites in China Only	Updated HBV serology tests	Updated the HBV DNA detection limit threshold.
**Appendix 12:** Protocol Amendment History	Added Amendment 7 history	Added the latest amendment history

Title Page

Protocol Title:	A Phase 1 first-in-human study evaluating safety, pharmacokinetics and efficacy of ABBV-706 as monotherapy and in combination with budigalimab (ABBV-181), carboplatin, or cisplatin in adult subjects with advanced solid tumors.
Brief Title:	A Phase 1 study of ABBV-706 alone or in combination in adult subjects with advanced solid tumors.
Acronym	M23-385
Compound:	ABBV-706
Indication:	Advanced solid tumors
Study Sponsor:	AbbVie*
Protocol Number:	M23-385
Study Phase:	Phase 1
Parexel/Emergency Medical Contact:	[redacted], MD<br>Mobile: [redacted]<br>Email: [redacted]
Sponsor/Emergency Medical Contact:	[redacted], MD<br>Mobile:[redacted]<br>Email: [redacted]
Regulatory Agency Identifying Number:	IND 162150
EU CT No.	2023-504598-18
Pediatric Investigational Plan Number:	Not applicable
Version:	4.0
Amendment Number:	9.0
Amendment Scope; Region/Country Identifier:	Global
Approval Date:	Final, 13-February-2026

Date and Version of Previous Protocol(s):	02-December-2024, Version 3.3.1<br>18-June-2024, Version 3.3<br>08-January-2024, Version 3.2.1<br>24-October-2023, Version 3.2<br>25-September-2023, Version 3.1.1<br>31-July-2023, Version 3.1<br>15-June-2023, Version 3.0<br>07-October-2022, Version 2.0<br>12-August-2022, Version 1.0
* For European Union countries: the Sponsor is AbbVie Deutschland GmbH & Co. KG. The specific contact details of the AbbVie legal/regulatory entity (person) within the relevant country are provided within the clinical trial agreement with the investigator/Institution and in the Clinical Trial Application with the Competent Authority.	

Sponsor Signatures

Protocol Title: A Phase 1 first-in-human study evaluating safety, pharmacokinetics and efficacy of ABBV-706 as monotherapy and in combination with budigalimab (ABBV-181), carboplatin, or cisplatin in adult subjects with advanced solid tumors.

Sponsor's approval of this clinical study protocol is located at the end of the document.

Protocol Amendment Summary of Changes Table

DOCUMENT HISTORY			
**Document**	**Amendment Scope; Region/Country Identifier (if applicable)**	**Protocol version**	**Date**
Amendment 9	Global	4.0	13-February-2026
Amendment 8	China Only	3.3.1	02-December-2024
Administrative Change 3	China Only	3.0	26-September-2024
Amendment 7	China Only	3.3	18-June-2024
Amendment 6	European Union Only	3.2.1	08-January-2024
Amendment 5	European Union Only	3.2	24-October-2023
Amendment 4	Japan Only	3.1.1	25-September-2023
Administrative Change 2	Global	2.0	05-September-2023
Amendment 3	Japan Only	3.1	31-July-2023
Administrative Change 1	Global	1.0	11-July-2023
Amendment 2	Global	3.0	15-June-2023
Amendment 1	Global	2.0	07-October-2022
Original Protocol	Not Applicable	1.0	12-August-2022

Amendment 9 (13-February-2026)

This amendment is considered to be substantial based on the criteria set forth in Article 10(a) of Directive 2001/20/EC of the European Parliament and the Council of the European Union.

This modification is considered to be substantial based on the criteria set forth in European Regulation 536/2014 Article 2(2)(13).

Overall Rationale for the Amendment:

The purpose of this amendment is to remove Parts 2b and 3b expansion (and update corresponding sample sizes), update contraception requirements for ABBV-706, make corrections for operational clarity, incorporate all country-specific versions into one global version, and other minor editorial changes for consistency throughout the protocol.

Section # and Name	Description of Change	Brief Rationale
**Title Page**<br>**Section 10.3.4:** Reporting Adverse Events and Intercurrent Illnesses	Updated Sponsor/Emergency Medical Contact	Change in personnel.
**Title Page**	Updated reference from "EudraCT" to "EU CT"	Per EMA request.
**Section 1.1:** Synopsis	Changes made throughout the synopsis	To reflect changes made within the protocol.
**Section 1.2:** Schema, Figure 1-3, Figure 1–4, and Figure 1–4	Added a Part 1b figure for the Japan cohort and a Part 2c figure for the China cohort. Updated the overall schema to remove Part 2b and update Part 3b.	Updated figure for accuracy to illustrate the updated study design.
**Section 1.3:** Schedule of Activities	Updated Table 1–2 as follows:<br>• Added periodic testing for coagulation parameters (aPTT/PT/INR) at an interval of every 4 cycles in addition to at screening<br>• Added amylase and lipase testing at screening; added footnote describing conditions for additional testing during the study as well as additional procedures if levels are > 3 × ULN	Address RFI from the EMA.
**Section 1.3:** Schedule of Activities (SoA)	In Parts 2 – 4, noted that peripheral blood collection for serum biomarkers and ctDNA are only required in odd cycles after Cycle 4.<br><br>CTCs collection is no longer required at any cycle.	Correction.<br><br>To reduce patient burden and operational complexity. Sufficient exploratory biomarker results generated, no further CTC collection needed.
**Section 1.3:** Schedule of Activities (SoA)	Footnote 'b' updated to note that Day 1 visits for Cycles 2+ have a ± 2 day window for laboratory assessments only.	Clarification.
**Section 1.3:** Schedule of Activities (SoA)<br>**Section 8: Study Assessments and Procedures**	From Cycle 6 and onward, visits on Days 8 and 15, and all assessments at those visits, may be considered optional based on Investigator's medical discretion.	To reduce subject burden.
**Section 1.3:** Schedule of Activities (SoA)	Added virology and biomarker testing requirements for sites in China and Japan.	To reflect specific requirements for sites in China and Japan.

Section # and Name	Description of Change	Brief Rationale
**Section 2.3:** Summary of Findings From Nonclinical Studies	Updated to include additional pharmacology and toxicology nonclinical data for ABBV-706.	To address RFI from the EMA.
**Section 2.4:** Benefit/Risk Assessment	Updated to include additional information from ongoing clinical studies to support the use of budigalimab as monotherapy and in combination with other agents.	To address RFI from the EMA.
**Section 3:** Objectives and Endpoints	Removed reference to Appendix A for biospecimen collection.	Correction, there is no Appendix A in the document.
**Section 3:** Objectives and Endpoints	Additional exploratory endpoints described for sites in China	To reflect specific requirements for sites in China.
**Section 4: Study Design**	Updated total number of subjects to be enrolled.	To reflect changes to cohorts made elsewhere in the protocol.
**Section 4: Study Design**	Updated estimated duration of the study to include 14-day screening period, 2-year treatment period, and 2-year follow-up.	To reflect the average predicted time on study.
**Section 4.1.1:** Part 1<br>**Section 4.1.1.2:** Part 1b	Split Part 1 into Part 1a (Global) and Part 1b (Japan only)	To reflect the different designs for the Monotherapy Dose Escalation phases for the Global Cohort and the Japan Cohort.
**Section 4.1.2:** Part 2 (Monotherapy Dose Optimization and Expansion in R/R SCLC)<br>**Section 4.3:** Justification for Dose<br>**Section 6.3:** Assignment to Study Intervention<br>**Section 9.3.2:** Efficacy Analysis<br>**Section 9.3.3.1:** Analysis of Adverse Events<br>**Section 9.3.3.2:** Analysis of Laboratory Data	Removed Part 2b.	Part 2b was no longer needed to select an ABBV-706 dose for further development in the SCLC indication. A separate Phase 3 study in R/R SCLC is planned.
**Section 4.1.2.2:** Part 2c (Monotherapy Safety Run-in and Dose Expansion for China)	Description of Part 2c added	To describe Part 2c cohort for China-only.

Section # and Name	Description of Change	Brief Rationale
**Section 4.1.3.1:** Part 3a (ABBV-706 and Budigalimab Combination Dose Escalation and Expansion)<br>**Section 4.1.3.2:** Part 3b (ABBV-706 and Platinum Chemotherapy Combination Dose Escalation)	Clarified that dose for part 3a and part 3b will be at or below the highest dose that clears the DLT evaluation in combination escalation and will not exceed the highest monotherapy dose selected for Part 2a dose optimization.	To address RFI from the EMA.
**Section 4.1.3.2:** Part 3b (ABBV-706 and Platinum Chemotherapy Combination Dose Escalation)	Removed 3b expansion and updated number of subjects to reflect update.	To accurately reflect the decision made from the review of the collective safety data and SMC recommendation to stop further enrollment in Part 3b.
**Section 4.1.5:** Dose Escalation Guidelines	Added section for Japan Cohort	To describe Dose Escalation for the Japan Cohort.
**Section 4.1.5:** Dose Escalation Guidelines	Updated definition of DLT evaluable subject.	To address RFI from the EMA.
**Section 4.1.6:** Safety Monitoring Committee	Updated text to indicate that the SMC will make safety assessments based on DLT equivalent events in Part 2c.	To clarify that the SMC will make the safety assessments based on DLT equivalent events in Part 2c.
**Section 4.1.7:** Interstitial Lung Disease Adjudication Committee	New section added to outline the activities of the ILD AC.	To ensure the safety of subjects.
**Section 4.1.10:** Dose Expansion and Safety Rules	Clarified that dose expansion safety rules apply to dose optimization, expansion cohorts, and backfill cohorts.	To address RFI from the EMA.

Section # and Name	Description of Change	Brief Rationale
**Section 5.1:** Eligibility Criteria for Parts 1, 2, 3, and 4	• Updated criterion #5 to clarify creatinine clearance required for subjects in parts 1, 2, 3a, 3b, and 4<br>• Updated criterion #10 to include additional considerations for tumor biopsies in cases where they may pose a risk to subjects<br>• Updated criterion #11 to clarify tumor types considered as high-grade NECs/NETs<br>• Updated criterion #12 to remove allowance for subjects who have refused SoC therapy to be included in the study; clarified that for eligible NECs and NETs, subjects must have received and/or considered all available approved therapeutic options, if applicable, prior to enrolling on the study<br>• Added criterion #22 to require that subjects in part 3b not have a history of hypersensitivity to the active ingredients or any excipients of cisplatin or carboplatin and/or contraindication to cisplatin or carboplatin<br>• Updated criterion #21 (now shown as #23) to define meaning of cured hepatitis B<br>• Updated criterion #24 (now shown as #26) to clarify that acceptable birth control methods are *highly* effective	To address RFI from the EMA.
**Section 5.1:** Eligibility Criteria for Parts 1, 2, 3, and 4<br>**Appendix 4:** Contraceptive and Barrier Guidance	Updated female contraceptive duration to 230 days and male contraception duration to 140 days	Per the updated ABBV-706 molecule half-life based on the available data from this study.
**Section 5.1:** Eligibility Criteria	Added specific eligibility criteria and edited existing criteria for sites in China	To reflect specific requirements for sites in China.
**Section 6.6: Study Intervention Product Complaints**	Added definitions for complaints and product complaints.	Per updated AbbVie protocol template.

Section # and Name	Description of Change	Brief Rationale
**Section 6.7.3: Hepatotoxicity**	• Updated Table 6–5 to specify that ABBV-706 is to be permanently discontinued if recurrence of Grade 3 toxicity occurs.<br>• Added footnote to indicate that ABBV-706 is to be permanently discontinued in cases of confirmed DILI	To address RFI from the EMA.
**Section 6.7.5:** Peripheral Neuropathy, Section 6.7.6: Dermatologic Toxicity	Updated Table 6–7 and Table 6–8 to specify that ABBV-706 is to be permanently discontinued if recurrence of Grade 3 toxicity occurs.	To address RFI from the EMA.
**Section 6.7.6:** Dermatologic Toxicity	Footnote added to Table 6–8 to specify that ABBV-706 treatment should be held for suspected TEN/SJS and permanently discontinued for confirmed TEN/SJS.	To address RFI from the EMA.
**Section 6.11.2:** Prohibited	Added herbal supplements and/or traditional Chinese medicines to medicines prohibited during study treatment	To reflect specific requirements for sites in China.
**Section 8.1.7:** Archival or Fresh Tumor Biopsy	Clarified conditions where tumor biopsies may not be available.	To address RFI from the EMA.
**Section 8.2:** Dose-Limiting Toxicity Assessment	Updated to clarify that the DLT observation period for part 3 escalation and also that the DLT are applicable for part 3a and 3b.	To address RFI from the EMA.
**Section 8.2:** Dose-Limiting Toxicity Assessment	Clarifying statement added for combination therapies meeting DLT criteria	To clarify that toxicities related to combination drugs meeting DLT criteria will be counted as DLTs.
**Section 8.4.6**: Pregnancy Testing	Updated pregnancy testing to 230 days from the last dose of study drug	Revised based on updated understanding of molecule half-life.
**Section 8.5.2**: Pregnancy	Updated collection of pregnancy in male subject's partners to 140 days following the last dose of study drug	Revised based on updated understanding of molecule half-life.
**Section 8.5.2:** Pregnancy<br>**Section 10.3.2:** Definition of SAE<br>**Section 10.3.4:** Reporting Adverse Events and Intercurrent Illnesses	Additions from Admin Change 2	To update document with changes made in Admin Change 2.

Section # and Name	Description of Change	Brief Rationale
**Section 8.9:** Biomarkers	Included additional considerations for tumor biopsies in cases where they may pose a risk to subjects.	To address RFI from the EMA.
**Section 8.9:** Biomarkers	Included information about where required blood volumes for biomarker assessments will be specified.	To clarify where information about required blood volumes for biomarker assessments can be found.
**Section 9.3.3:** Safety Analyses	Clarified treatment-emergent period	To accurately define the treatment-emergent period for all parts of the study.
**Section 9.4.1:** Futility Analysis	Added Section 9.4.1 Futility Analysis to the protocol.	To address RFI from the EMA.
**Section 9.6:** Sample Size Determination	Changed total number of subjects to include the Japan Cohort and China Cohort and the removal of part 2b and part 3b expansion.	To accurately represent subject numbers.
**Appendix 2:** Clinical Laboratory Tests	Updated to include amylase and lipase testing.	To address RFI from the EMA.
**Appendix 4:** Contraceptive and Barrier Guidance	Updated available methods of birth control.	Per updated AbbVie protocol template.
**Appendix 4:** Contraceptive and Barrier Guidance	Updated to specify the use of ultra-low estrogen contraceptives.	To address RFI from the EMA.
**Appendix 11: Country-Specific Requirements**	Added Japan-specific requirements for eligibility, virology testing, biomarker testing, safety assessments, physical examinations, and contraception.<br>Added supplemental information for Japan Sites on scientific findings on drugs, handling of drugs used in the clinical trial, and reporting of serious adverse event and adverse event of special interest	To provide additional information to Japan sites as needed for the Japan specific regulatory requirements of the revision of Order for Enforcement of the Act on Securing Quality, Efficacy and Safety of Products Including Pharmaceuticals and Medical Devices in Japan.<br>To clarify eligibility criteria for female subjects of childbearing potential in Japan.
**Appendix 11: Country-Specific Requirements**	Added China-specific requirements for eligibility, virology testing, biomarker testing, and paper diary cards.	To reflect specific requirements for sites in China.
**Appendix 12**: Protocol Amendment History	Moved last protocol amendment details to Appendix	Per template guidance.
Throughout the protocol	Updated wording to list out specific types of high-grade NEC and NET tumors.	To address RFI from the EMA.
Throughout the protocol.	Minor editorial corrections were made	For clarity and consistency throughout the document.

Table of Contents

Title Page 1

Sponsor Signatures 2

Protocol Amendment Summary of Changes Table 3

DOCUMENT HISTORY 3

Table of Contents 10

List of Tables 15

List of Figures 16

Abbreviations and Definitions 18

1 Protocol Summary 22

1.1 Synopsis 22

1.2 Schema 28

1.3 Schedule of Activities (SoA) 30

2 Introduction 41

2.1 Study Rationale 41

2.2 Background 41

2.3 Summary of Findings from Nonclinical Studies with Potential Clinical Relevance 42

2.4 Benefit/Risk Assessment 44

3 Objectives and Endpoints 45

4 Study Design 46

4.1 Overall Design 46

4.1.1 Part 1 (Monotherapy Dose Escalation) 46

4.1.1.1 Part 1a (Monotherapy Dose Escalation) 46

4.1.1.2 Part 1b (Japan Only) 47

4.1.2 Part 2 (Monotherapy Dose Optimization and Expansion in R/R SCLC) 47

4.1.2.1 Part 2a (Monotherapy Dose Optimization) 47

4.1.2.2 Part 2c (Monotherapy Safety Run-in and Dose Expansion for China) 48

4.1.3 Part 3 (Combination Dose Escalation and Expansion) 49

4.1.3.1 Part 3a (ABBV-706 and Budigalimab Combination Dose Escalation and Expansion) 49

4.1.3.2 Part 3b (ABBV-706 and Platinum Chemotherapy Combination Dose Escalation) 49

4.1.4 Part 4 (ABBV-706 Monotherapy Dose Expansion) ... 50
4.1.4.1 Part 4a (ABBV-706 Monotherapy Dose Expansion in R/R CNS Tumors) ... 50
4.1.4.2 Part 4b (ABBV-706 Monotherapy Dose Expansion in R/R high-grade NECs/NETs) 50
4.1.5 Dose Escalation Guidelines ... 50
4.1.6 Safety Monitoring Committee ... 52
4.1.7 Interstitial Lung Disease Adjudication Committee ... 53
4.1.8 Backfill Guidelines ... 53
4.1.9 Selection of Maximum Tolerable Dose ... 53
4.1.10 Dose Expansion Safety Rules ... 54
4.2 Scientific Rationale for Study Design ... 54
4.2.1 Appropriateness of Measurements ... 54
4.2.2 Suitability of Subject Population ... 55
4.3 Justification for Dose ... 55
4.4 End-of-Study Definition ... 56
5 Study Population ... 56
5.1 Eligibility Criteria for Parts 1, 2, 3, and 4 ... 56
5.2 Additional Exclusion Criteria for Part 3a (Budigalimab Combination): ... 61
5.3 Lifestyle Considerations ... 61
5.3.1 Sunlight Exposure ... 61
5.3.2 Contraception ... 62
5.4 Screen Failure ... 62
6 Study Intervention(s) and Concomitant Therapy ... 62
6.1 Study Intervention(s) Administered ... 62
6.2 Preparation, Handling, Storage, and Accountability ... 64
6.3 Assignment to Study Intervention ... 64
6.4 Blinding/Masking ... 65
6.5 Study Intervention Compliance ... 65
6.6 Study Intervention Product Complaints ... 65
6.7 Management of Drug-Related Toxicities ... 66
6.7.1 Hematologic Toxicity ... 67
6.7.2 ILD/Pneumonitis Toxicity ... 69
6.7.3 Hepatotoxicity ... 71
6.7.4 Infusion-Related Reactions (IRRs) ... 71
6.7.5 Peripheral Neuropathy ... 72
6.7.6 Dermatologic Toxicity ... 73

6.7.7 Immune-Related Adverse Events for Part 3a Combination with Budigalimab 74
6.8 Continued Access to Study Intervention After the End of the Study 74
6.9 Treatment of Overdose 74
6.10 Prior and Concomitant Medications Review 75
6.11 Concomitant Medications/Therapy 75
6.11.1 Allowed 75
6.11.2 Prohibited 75
6.11.3 Concomitant Radiation Therapy 76
6.12 COVID-19 Pandemic-related Vaccination Guidance 77
7 Discontinuation of Study Intervention and Withdrawal From the Study 78
7.1 Subject Discontinuation From Study Intervention 78
7.1.1 COVID-19 Pandemic-Related Acceptable Protocol Modification 79
7.1.2 Interruption/Discontinuation of Study Drug Due to COVID-19 Infection 79
7.2 Subject Withdrawal From the Study 79
7.3 Lost to Follow-Up 79
7.4 Withdrawal From Biomarker Research 80
8 Study Assessments and Procedures 80
8.1 Administrative and Baseline Procedures 81
8.1.1 Study Subject Informed Consent 81
8.1.2 Informed Consent for Biomarker Research 81
8.1.3 Medical and Cancer History 81
8.1.4 Audiology Assessment - Part 3b Only 82
8.1.5 Echocardiogram or MUGA 82
8.1.6 Eastern Cooperative Oncology Group Performance Status 82
8.1.7 Archival or Fresh Tumor Biopsy 83
8.2 Dose-Limiting Toxicity Assessment 83
8.3 Tumor Response Assessments 85
8.4 Safety Assessments 86
8.4.1 Physical Examination 86
8.4.2 Height and Weight 86
8.4.3 Vital Signs 86
8.4.4 Standard 12-lead Electrocardiograms 87
8.4.5 Clinical Safety Laboratory Tests 88
8.4.6 Pregnancy Testing 89
8.5 Adverse Events, Serious Adverse Events, and Other Safety Reporting 90

8.5.1 Time Period and Frequency for Collecting AE and SAE Information 90
8.5.2 Pregnancy 91
8.6 Pharmacokinetics and Immunogenicity 91
8.6.1 Blood Sample Collection 91
8.6.2 PK/ADA Sample Disposition 94
8.6.3 Immunogenicity Assessments 94
8.7 Pharmacodynamics 94
8.8 Genetics 95
8.9 Biomarkers 95
8.10 Storage and Future Use of Biological Samples 96
9 Statistical Considerations 96
9.1 Statistical Hypotheses 96
9.2 Analysis Sets 96
9.3 Statistical Analyses 97
9.3.1 General Considerations 97
9.3.2 Efficacy Analysis 97
9.3.3 Safety Analyses 98
9.3.3.1 Analysis of Adverse Events (AEs) 98
9.3.3.2 Analysis of Laboratory Data 99
9.3.3.3 Analysis of Other Safety Data 99
9.3.4 Pharmacokinetic Analyses 99
9.4 Interim Analyses 99
9.4.1 Futility Analysis 99
9.5 Overall Type I Error Control 99
9.6 Sample Size Determination 100
10 Supporting Documentation and Operational Considerations 101
10.1 Appendix 1: Regulatory, Ethical, and Study Oversight Considerations 101
10.1.1 Regulatory and Ethical Considerations 101
10.1.2 Data Protection 101
10.1.3 Data Handling 102
10.1.4 Investigator Record Retention 103
10.1.5 Data Quality Assurance 103
10.1.6 Source Documents and Access to Source Data 104
10.1.7 Study and Site Start and Closure 105
10.1.8 Publication Policy 105

10.1.9 Protocol Approval and Amendment 106
10.2 Appendix 2: Clinical Laboratory Tests 107
10.3 Appendix 3: AEs and SAEs: Definitions and Procedures for Recording, Evaluating, Follow-up, and Reporting 109
10.3.1 Definition of AE 109
10.3.2 Definition of SAE 110
10.3.3 Recording and Follow-up of AE and SAE 112
10.3.4 Reporting Adverse Events and Intercurrent Illnesses 114
10.4 Appendix 4: Contraceptive and Barrier Guidance 117
10.5 Appendix 5: Modification of Diet in Renal Disease 119
10.6 Appendix 6: Strong CYP3A Inhibitor and Inducer List 120
10.7 Appendix 7: Guidance Regarding Drugs Known to Prolong QT Interval 121
10.8 Appendix 8: Part 3a Budigalimab Combination Immune-Related Adverse Event Management Guidance 122
10.8.1 General Guidance Regarding Management of Immune-Related Adverse Events Following Budigalimab (ABBV-181) Treatment 122
10.8.2 Skin Adverse Event Management Algorithm 123
10.8.3 Cutaneous Adverse Reactions Management Algorithm 125
10.8.4 Gastrointestinal Adverse Event Management Algorithm 130
10.8.5 Renal Adverse Event Management Algorithm 132
10.8.6 Pulmonary Adverse Event Management Algorithm 133
10.8.7 Primary Hypothyroidism Adverse Event Management Algorithm 135
10.8.8 Hyperthyroidism Adverse Event Management Algorithm 136
10.8.9 Primary Adrenal Insufficiency Adverse Event Management Algorithm 138
10.8.10 Pituitary-Hypophysitis Adverse Event Management Algorithm 141
10.8.11 Diabetes Management Algorithm 142
10.8.12 Neurological Adverse Event Management Algorithm 144
10.8.13 Hepatic Adverse Event Management Algorithm 145
10.9 Appendix 9: RECIST 1.1 149
10.9.1 Evaluation of Target Lesions 149
10.9.2 Evaluation of Non-Target Lesions 149
10.9.3 New Lesions 150
10.10 Appendix 10: RANO Response Criteria for Subjects With Primary CNS Tumor 151
10.11 Appendix 11: Country Specific Requirements 155
10.11.1 Japan-Specific Requirements 155
10.11.1.1 Latest Scientific Findings on Drugs Used in the Clinical Trial 155
10.11.1.2 Handling of Drugs used in the Clinical Trial 155

10.11.1.3 Reporting of Serious Adverse Event and Adverse Event of Special Interest 155
10.11.1.4 Eligibility (Japan Only) 156
10.11.1.5 Contraception Requirements (Japan Only) 156
10.11.1.6 Safety Assessments (Japan Only) 156
10.11.1.7 Clinical Laboratory Testing (Japan Only) 157
10.11.1.8 Biomarkers (Japan Only) 159
10.11.2 China-Specific Requirements 159
10.11.2.1 Eligibility (China Only) 159
10.11.2.2 Clinical Laboratory Tests (China Only) 160
10.11.2.3 Biomarkers (China Sites Only) 162
10.11.2.4 Subject Paper Diary Cards 162
10.12 Appendix 12: Protocol Amendment History 164
10.13 Appendix 13: Investigator Agreement 171
11 References 173

List of Tables

Table 1–1 Schedule of Activities: Part 1 (Monotherapy Dose Escalation) 31

Table 1–2 Schedule of Activities: Parts 2-4 (Monotherapy Dose Expansions, Combo Escalations and Expansions) 35

Table 3–1 Objectives, Endpoints, and Estimands 45

Table 4–1 Planned Dose Levels 51

Table 4–2 BOIN Dose Escalation Rules for M23-385 52

Table 6–1 Study Intervention(s) Administered 64

Table 6-2 ABBV-706 Toxicity Management Guidelines for Neutropenia/Febrile Neutropenia 68

Table 6-3 Toxicity Management Guidelines for Thrombocytopenia 69

Table 6–4 Management of ILD/Pneumonitis 70

Table 6–5 ABBV-706 Dose Modification Guidelines for Hepatotoxicity 71

Table 6–6 Management of Infusion-Related Reactions 72

Table 6–7 Dose Modification Guidelines for Peripheral Neuropathy 73

Table 6–8 Dose Modification Guidelines for Dermatologic Toxicity 74

Table 8–1 Sampling Schedule of ABBV-706 PK, ECG, and ADA Assessments 93

Table 9–1 Subject Analysis Sets 97

Table 9–2 Exact 1-Sided 80% Confidence Intervals From Various Observed Objective Response Rates Given a Cohort Size of 40 Subjects 100

Table 10–1 Protocol-Required Safety Laboratory Tests 107

Table 10–2 Management of Immune-Related Adverse Events Following Budigalimab (ABBV-181) Treatment.. 123

Table 10–3 Skin Adverse Event Management Algorithm 124

Table 10–4 Cutaneous Adverse Reactions Management Algorithm 128

Table 10–5 Gastrointestinal Adverse Event Management Algorithm 131

Table 10–6 Renal Adverse Event Management Algorithm 133

Table 10–7 Pulmonary Adverse Event Management Algorithm 134

Table 10–8 Primary Hypothyroidism Adverse Event Management Algorithm.................... 135

Table 10–9 Hyperthyroidism Adverse Event Management Algorithm 137

Table 10–10 Primary Adrenal Insufficiency Adverse Event Management Algorithm 139

Table 10–11 Pituitary-Hypophysitis Adverse Event Management Algorithm 141

Table 10–12 Diabetes Management Algorithm 143

Table 10–13 Neurological Management Algorithm 144

Table 10–14 Suspected Immune-Related Hepatic Adverse Event Management Algorithm ... 147

Table 10–15 Bidimensional to Volumetric Definitions of Radiographic Response and Progression.. 151

Table 10-16 Latest Scientific Findings on Drugs used in the Clinical Trial 155

Table 10-17 Protocol-Required Safety Laboratory Tests for Japan Only 157

Table 10–18 Protocol-Required Safety Laboratory Tests for China Only.............................. 161

List of Figures

Figure 1–1 Study Design (Part 1a) 29

Figure 1-2 Study Design (Part 1b) 29

Figure 1-3. Study Design (Part 2c) 30

Figure 1–4 Overall Study Schema 30

Figure 10-1 Guidelines for the Prevention of Hepatitis B Virus Reactivation in Subjects Receiving Immunosuppressive Therapy or Chemotherapy 159

Abbreviations and Definitions

Abbreviation	Description
ACTH	Adrenocorticotropic hormone
ADA	Antidrug antibody
ADC	Antibody Drug Conjugate
AE	Adverse event
ALT	Alanine aminotransferase
aPTT	Activated partial thromboplastin time
ASCO	American Society of Clinical Oncology
AST	Aspartate aminotransferase
AUC	Area under the concentration-time curve
BOIN	Bayesian optimal interval
CAR	Cutaneous adverse reactions
CBC	Complete blood count
CI	Confidence interval
C_{max}	Maximum observed serum (or plasma, for payload) concentration
CNS	Central nervous system
COVID-19	Corona virus disease 2019
CR	Complete response
CRO	Contract Research Organization
CT	Computerized tomography
CTC	Circulating tumor cell
ctDNA	Circulating tumor DNA
CTR	Clinical trials regulation
CYP	Cytochrome P450
DAR	Drug-to-antibody ratio
DIHS	Drug-induced hypersensitivity syndrome
DLT	Dose-limiting toxicities
DNA	Deoxyribonucleic acid
DOR	Duration of response
DRESS	Drug reaction with eosinophilia and systemic symptoms
DSUR	Development Safety Update Report
ECG	Electrocardiogram

Abbreviation	Description
ECOG	Eastern Cooperative Oncology Group
eCRF	Electronic case report form
EDC	Electronic data capture
EGFR	Epidermal growth factor receptor
EOT	End of treatment
ESMO	European Society for Medical Oncology
FIH	First-in-Human
FOLFIRI	Chemotherapy regimen of FOLinic acid, Fluorouracil, and IRInotecan
FOLFIRINOX	Chemotherapy regimen of FOLinic acid, Fluorouracil, IRINotecan, and OXaliplatin
FOLFOX	Chemotherapy regimen of FOLinic acid and OXaliplatin
FSH	Follicle-stimulating hormone
GBM	Glioblastoma
GCP	Good Clinical Practice
G-CSF	Granulocyte-colony stimulating factor
GEP-NEC	Gastroenteropancreatic neuroendocrine carcinoma
GI	Gastrointestinal
HIV	Human immunodeficiency virus
HNSTD	Highest Non-Severely Toxic Dose
ICE	Intercurrent event
ICF	Informed consent form
ICH	International Council for Harmonization
IEC	Independent ethics committee
IHC	Immunohistochemistry
ILD	Interstitial lung disease
IgG1	Immunoglobulin G1
IMP	Investigational Medicinal Product
INR	International normalized ratio
irAE	Immune-related adverse event
IRB	Institutional review board
IRR	Infusion-related reaction
IRT	Interactive Response Technology
IV	Intravenous

Abbreviation	Description
IVDR	In vitro diagnostic medical device regulation
IVIJ	Intravenous immunoglobulin
LCNEC	Large cell neuroendocrine carcinoma
LH	Luteinizing hormone
MAD	Maximum administered dose
MDRD	Modification of Diet in Renal Disease
MedDRA	Medical Dictionary for Regulatory Activities
MRI	Magnetic resonance imaging
MTC	Medullary thyroid cancer
MTD	Maximum tolerable dose
MUGA	Multigated acquisition
nAb	Neutralizing antibody
NCCN	National Comprehensive Cancer Network
NCI CTCAE	National Cancer Institute Common Terminology Criteria for Adverse Events
NEC	Neuroendocrine carcinoma
NEPC	Neuroendocrine prostate cancer
NET	Neuroendocrine tumor
NSCLC	Non-small cell lung cancer
ORR	Objective response rate
OS	Overall survival
PCR	Polymerase chain reaction
PCV	Chemotherapy regimen of procarbazine, lomustine, and vincristine
PD	Progressive disease
PET	Positron emission tomography
PFS	Progression-free survival
PK	Pharmacokinetics
PR	Partial response
PS	Performance status
PT	Prothrombin time or Preferred term
Q3Wk	Once every 3 weeks (21 days)
QTc	QT interval corrected for heart rate
R/R	Relapsed/refractory
RANO	Response Assessment for Neuro-Oncology

Abbreviation	Description
RBC	Red blood cells
RECIST	Response Evaluation Criteria in Solid Tumors
RP2D	Recommended Phase 2 dose
RSI	Reference Safety Information
SAE	Serious adverse event
SARS-CoV-2	Severe acute respiratory syndrome coronavirus 2
SCLC	Small cell lung cancer
SD	Stable disease
SEZ6	Seizure-related 6 homolog
SITC	Society for Immunotherapy of Cancer
SJS	Stevens-Johnson syndrome
SMC	Safety Monitoring Committee
SoA	Schedule of activities
SoC	Standard of care
$t_{½}$	Terminal phase elimination half-life
TEN	Toxic epidermal necrolysis
TKI	Tyrosine kinase inhibitor
T_{max}	Time of maximum serum (or plasma, for payload) concentration
Top1	Topoisomerase 1
ULN	Upper limit of normal
WOCBP	Women of childbearing potential

1 Protocol Summary

1.1 Synopsis

Protocol Title: A Phase 1 first-in-human study evaluating safety, pharmacokinetics and efficacy of ABBV-706 as monotherapy and in combination with budigalimab (ABBV-181), carboplatin, or cisplatin in adult subjects with advanced solid tumors.

Brief Title: A Phase 1 study of ABBV-706 alone or in combination in adult subjects with advanced solid tumors.

Sponsor Protocol No.: Error! Reference source not found.

Study Phase: Error! Reference source not found.

Sponsor: Error! Reference source not found.

Regulatory Agency Identifier Number(s): Error! Reference source not found.

Registry ID: TBC

PIP Number: Not applicable

Rationale:

ABBV-706 is an antibody drug conjugate (ADC) with an anti-seizure-related 6 homolog (SEZ6) antibody and topoisomerase 1 (Top1) inhibitor payload. Preclinical data indicate a favorable risk-benefit profile in tumors which express SEZ6. There is a large unmet medical need for more effective therapy targeting SEZ6 expressing tumors such as small cell lung cancer (SCLC), high-grade central nervous system (CNS) tumors (glioblastoma [GBM], IDH-wildtype Grade 4; oligodendroglioma, IDH-mutant, and 1p/19q-codeleted Grade 3; astrocytoma, IDH-mutant Grade 3 or Grade 4), and diverse high-grade, neuroendocrine carcinomas (NECs)/neuroendocrine tumors (NETs).

Objectives, Endpoints, and Estimands:

Objectives	Endpoints
**Primary**	
To evaluate the safety and tolerability of ABBV-706 administered as monotherapy and in combination with either budigalimab, carboplatin, or cisplatin	Safety evaluations include adverse events (AEs), vital sign measurements, electrocardiogram (ECG) variables, and clinical laboratory tests (hematology, chemistry, and urinalysis) as measures of safety and tolerability for the entire study duration.

Objectives	Endpoints
To evaluate the pharmacokinetics (PK) and immunogenicity of ABBV-706 administered as monotherapy and in combination with either budigalimab, carboplatin, or cisplatin	Pharmacokinetic parameters, including the maximum observed serum (or plasma, for payload) concentration (C_{max}), the time to C_{max} (T_{max}), terminal phase elimination half-life ($t_{1/2}$), and the area under the serum/plasma concentration-time curve (AUC), will be calculated using noncompartmental methods for ABBV-706 total antibody, ADC, and unconjugated Top1 inhibitor payload.<br><br>Additional parameters may be calculated and/or analyses may be conducted if useful in the interpretation of the data.<br><br>Antidrug antibodies (ADAs) and neutralizing antibodies (nAbs) will be determined, as appropriate.
To determine the recommended Phase 2 dose (RP2D) of ABBV-706 as monotherapy	The RP2D will be determined using all available information, including, but not limited to, AEs, dose-limiting toxicities, PK parameters, clinical laboratory tests, and efficacy measures.
To evaluate the antitumor activity of ABBV-706 in subjects with R/R SCLC, high-grade CNS tumors (GBM, IDH-wildtype Grade 4; oligodendroglioma, IDH-mutant, and 1p/19q-codeleted Grade 3; astrocytoma, IDH-mutant Grade 3 or Grade 4), and high-grade neuroendocrine carcinomas (NECs)/neuroendocrine tumors (NETs)	Confirmed complete response/partial response (CR/PR) as assessed by investigators per Response Evaluation Criteria in Solid Tumors (RECIST), version 1.1 for extracranial solid tumors and Response Assessment for Neuro-Oncology (RANO) for subjects with high-grade CNS tumors (GBM, IDH-wildtype Grade 4; oligodendroglioma, IDH-mutant, and 1p/19q-codeleted Grade 3; astrocytoma, IDH-mutant Grade 3 or Grade 4).<br><br>Duration of response (DOR) for subjects with confirmed CR/PR.

Objectives	Endpoints
**Exploratory/Other**	
To evaluate the clinical benefit rate, progression-free survival (PFS), and overall survival (OS) of ABBV-706 in subjects with R/R SCLC, high-grade CNS tumors (GBM, IDH-wildtype Grade 4; oligodendroglioma, IDH-mutant, and 1p/19q-codeleted Grade 3; astrocytoma, IDH-mutant Grade 3 or Grade 4), and high-grade NECs/NETs	Clinical benefit defined as CR/PR, stable disease (SD).<br>PFS.<br>OS.
To evaluate pharmacodynamic and predictive biomarkers for association with PK, safety, and efficacy measurements<br>For the China study cohort: To evaluate SEZ6 target expression in tumor tissue measured by IHC and RNAseq for its association with PK, safety, and antitumor activity.	Biomarker Endpoints:<br>Biospecimens will be collected to investigate known / novel biomarkers.<br>Measurement of potential predictive biomarkers and their association with PK, safety, and antitumor activity.<br>Measurement of the effect of ABBV-706 on target engagement, pharmacodynamics, and disease burden.<br>For the China study cohort, SEZ6 expression in tumor tissue will be measured by IHC and RNAseq.
To evaluate the potential effects of ABBV-706 monotherapy on QT/QT corrected for heart rate (QTc) interval prolongation	QT/QTc interval and change from baseline.

Overall Design Summary

This is a Phase 1 first-in-human (FIH) dose escalation and dose expansion study evaluating safety, PK, and antitumor activity of ABBV-706 as monotherapy and in combination with budigalimab, carboplatin, or cisplatin in adult subjects with advanced solid tumors.

Study Intervention and Intervention Form: ABBV-706 will be administered as an [redacted] until disease progression, withdrawal of consent, or unacceptable toxicity.

Condition/Disease: Adult subjects with advanced solid tumors, which include relapsed/refractory (R/R) SCLC, high-grade NECs/NETs (specific tumor types include, but not limited to, NEPC, GEP-NECs, LCNECs, SCLC transformed from EGFR mutant NSCLC, MTC, and other NECs [atypical lung carcinoids that have received prior chemotherapy are allowed]),

and high-grade CNS tumors (GBM, IDH-wildtype Grade 4; oligodendroglioma, IDH-mutant, and 1p/19q-codeleted Grade 3; astrocytoma, IDH-mutant Grade 3 or Grade 4).

Expected Study and Treatment Duration: The duration of the study for each subject will be approximately 4 years and will include a 14-day screening period followed by an approximately 2-year treatment period and up to 2-year follow-up after the last dose of study drug.

Number of Subjects: The total number of subjects will be approximately 319. For Part 1a (Monotherapy Dose Escalation), up to 60 subjects with solid tumors will be enrolled; for Part 1b (Monotherapy Dose Escalation for Japan), up to 24 subjects with solid tumors will be enrolled in Japan only. For Part 2 (Monotherapy Dose Optimization and Expansion in R/R SCLC), up to 95 subjects (up to 80 subjects globally and up to 15 subjects in China only) will be enrolled depending on the number and size of the randomized dose optimization cohorts and the requirement of a SEZ6 expression threshold and/or line of therapy. Subjects in Part 2a will be randomized evenly between at least 2 dose cohorts. For Part 2c (Monotherapy safety run-in/Dose expansion for China), up to 15 subjects with R/R SCLC will be enrolled in China only. In Parts 3a and 3b (Combination Dose Escalation with budigalimab or platinum chemotherapy, respectively), up to 30 and 30 subjects will be enrolled respectively and is sufficient for an initial safety dataset to enable future efficacy studies. Parts 4a and 4b (Monotherapy Dose Expansion in R/R CNS tumors or high-grade NECs/NETs, respectively) will enroll up to 40 subjects each.

Safety Monitoring Committee: All dose escalation decisions will be made by the Sponsor in consultation with the Safety Monitoring Committee (SMC). The SMC will meet routinely to review the emerging safety, PK, and pharmacodynamic data throughout the study, and to make dose escalation recommendations during Parts 1a and 1b, and to make starting dose and dose escalation recommendations for Parts 3a and 3b. The SMC will also review aggregate safety data in order to identify safety concerns that may emerge due to cumulative exposure beyond the DLT period.

Key Eligibility Criteria

- Age ≥ 18 years.
- ECOG performance status of 0 or 1.
- The following laboratory values criteria must be met within 7 days prior to the first dose of study drug:
    - Alanine aminotransferase (ALT) and aspartate aminotransferase (AST) ≤ 3.0 × the upper limit of normal (ULN).
        - For subjects with liver metastases: ALT and AST ≤ 5.0 × ULN
    - Total bilirubin ≤ 1.5 × ULN. (Subjects with documented Gilbert's syndrome may have a total bilirubin ≤ 3 × ULN).

- Creatinine clearance ≥ 30 mL/min for subjects in Part 1, Part 2, Part 3a, and Part 4 and > 60 mL/min for subjects in Part 3b calculated using the Modification of Diet in Renal Disease (MDRD) equation.
- Absolute neutrophil count ≥ 1,500/mm^3 (with no granulocyte-colony stimulating factor [G-CSF] in the past 10 days).
- Platelet count ≥ 100,000/µL (with no platelet transfusion in the past 7 days).
- Hemoglobin ≥ 8 g/dL (with no red blood cell [RBC] transfusion in the past 7 days).

- QT interval corrected for heart rate (QTc) ≤ 450 msec (males) or ≤ 470 msec (females) using Fridericia's correction, and an ejection fraction of ≥ 50% as measured by echocardiogram or multigated acquisition (MUGA) scan at Screening.

- Subjects must have one of the following histologically or cytologically confirmed advanced relapsed or refractory solid tumors:

  - **Part 1 only**: Solid tumors with potential SEZ6 expression including (but not limited to) SCLC, high-grade CNS tumors (GBM, IDH-wildtype Grade 4; oligodendroglioma, IDH-mutant, and 1p/19q-codeleted Grade 3; astrocytoma, IDH-mutant Grade 3 or Grade 4), high-grade NECs/NETs (e.g. Neuroendocrine prostate cancer [NEPC], GEP-NECs, LCNECs, SCLC transformed from epidermal growth factor receptor [EGFR] mutant non-small cell lung cancer [NSCLC], medullary thyroid cancer [MTC], and other NECs, or Grade 3 NETs [atypical lung carcinoids that have received prior chemotherapy are allowed])

  - **Part 2 only**: SCLC

  - **Part 3a only**: SCLC or Grade 3 NETs and poorly differentiated NECs. Specific tumor types include but not limited to NEPC, GEP-NECs, LCNECs, SCLC transformed from EGFR mutant NSCLC, MTC, and other NECs (atypical lung carcinoids that have received prior chemotherapy are allowed).

  - **Part 3b only**: SCLC who have only progressed following a frontline regimen containing a platinum-based chemotherapy (i.e., second-line SCLC subjects) or Grade 3 NETs and poorly differentiated NECs. Specific tumor types include but not limited to NEPC, GEP-NECs, LCNECs, SCLC transformed from EGFR mutant NSCLC, MTC, and other NECs (atypical lung carcinoids that have received prior chemotherapy are allowed).

  - **Part 4a only**: High-grade CNS tumors

  - **Part 4b only**: Grade 3 NETs and poorly differentiated NECs. Specific tumor types include but not limited to NEPC, GEP-NECs, LCNECs, SCLC transformed from EGFR mutant NSCLC, MTC, and other NECs (atypical lung carcinoids that have received prior chemotherapy are allowed).

- Subjects must have progressed on or after standard of care (SoC) therapy as follows and with no curative therapy available (subjects who are considered ineligible for or are intolerant of SoC therapy are eligible):
  - For SCLC, subjects must have relapsed or refractory disease following at least 1 prior platinum-containing chemotherapy. For the purposes of this study, a line of therapy is defined as ≥ 1 complete cycle of either a single agent or combination of drugs, including any planned sequential therapy of various regimens (e.g., 4 cycles of initial therapy with carboplatin-etoposide and atezolizumab or durvalumab followed by maintenance with atezolizumab or durvalumab is considered first line). For Part 1b, subjects must have previously been treated with topotecan hydrochloride or amrubicin hydrochloride in addition to platinum-based chemotherapy, or subjects are not eligible for these SoC therapies.
  - For high-grade CNS tumors, subjects must have had prior surgical resection, received prior radiation, and temozolomide or procarbazine, lomustine, and vincristine (PCV).
  - For eligible NECs and NETs, subjects must have received and/or considered all available approved therapeutic options, if applicable, prior to enrolling on the study.
- Measurable disease per RECIST version 1.1 for subjects with extracranial solid tumors or RANO for subjects with primary high-grade CNS tumors (GBM, IDH-wildtype Grade 4; oligodendroglioma, IDH-mutant, and 1p/19q-codeleted Grade 3; astrocytoma, IDH-mutant Grade 3 or Grade 4).
- Primary CNS tumors within 12 weeks from radiation therapy should have unequivocal progression as documented by either tumor recurrence predominantly outside of radiation field on magnetic resonance imaging (MRI) or confirmed on tumor biopsy.
- Subjects with brain metastases from an extracranial solid tumor are eligible if the brain metastases are:
  - Previously treated, currently stable and not requiring steroids or anticonvulsants to control associated symptoms.
  - Untreated, asymptomatic, with fewer than 5 lesions and less than 5 mm in size each and not requiring steroids or anticonvulsants to control associated symptoms.
- Fresh or archival tumor tissue available for submission, for retrospective SEZ6 expression analysis, as per tissue submission guidelines in the Laboratory Manual.
  - Subjects that have previously provided tissue sampling for SEZ6 testing in other AbbVie studies and meet the sampling requirements outlined by the Sponsor will not be required to provide further tissue samples for this study.
  - If archival tissue is not available and obtaining a fresh biopsy poses significant risk to the safety of the subject, the treating physician can discuss the case with the medical monitor or Sponsor to determine eligibility.

- No history of interstitial lung disease (ILD) or pneumonitis that required treatment with systemic steroids, nor any evidence of active ILD or pneumonitis.
- No history of idiopathic pulmonary fibrosis or organizing pneumonia (e.g., bronchiolitis obliterans).
- No prior treatment with an ADC that consists of a Top1 inhibitor payload.
- Part 2 (SCLC monotherapy dose optimization and expansion) only: No prior treatment with a SEZ6-targeted antibody drug conjugate.

1.2 Schema

This is a Phase 1, FIH, open-label, proof of concept study evaluating ABBV-706 in subjects with advanced solid tumors organized in four parts:

- Part 1 – a) – ABBV-706 monotherapy dose escalation in advanced solid tumors with potential SEZ6 expression (Figure 1–1); b) – ABBV-706 monotherapy dose escalation in advanced solid tumors with potential SEZ6 expression (Japan only) (Figure 1-2)
- Part 2 – ABBV-706 monotherapy dose optimization and expansion in R/R SCLC (Figure 1-3, Figure 1–4)
- Part 3 – a) – ABBV-706 + budigalimab (anti-PD-1) combination dose escalation and expansion in R/R SCLC and high-grade NECs/NETs (specific tumor types include, but not limited to, NEPC, GEP-NECs, LCNECs, SCLC transformed from EGFR mutant NSCLC, MTC, and other NECs [atypical lung carcinoids that have received prior chemotherapy are allowed]); b) ABBV-706 + platinum chemotherapy combination dose escalation in R/R SCLC and high-grade NECs/NETs (specific tumor types include, but not limited to, NEPC, GEP-NECs, LCNECs, SCLC transformed from EGFR mutant NSCLC, MTC, and other NECs [atypical lung carcinoids that have received prior chemotherapy are allowed]) (Figure 1–4)
- Part 4 – ABBV-706 monotherapy dose expansion in a) R/R high-grade CNS tumors (GBM, IDH-wildtype Grade 4; oligodendroglioma, IDH-mutant, and 1p/19q-codeleted Grade 3; astrocytoma, IDH-mutant Grade 3 or Grade 4) and b) R/R high-grade NECs/NETs (specific tumor types include, but not limited to, NEPC, GEP-NECs, LCNECs, SCLC transformed from EGFR mutant NSCLC, MTC, and other NECs [atypical lung carcinoids that have received prior chemotherapy are allowed]) (Figure 1–4)

Figure 1–1 Study Design (Part 1a)

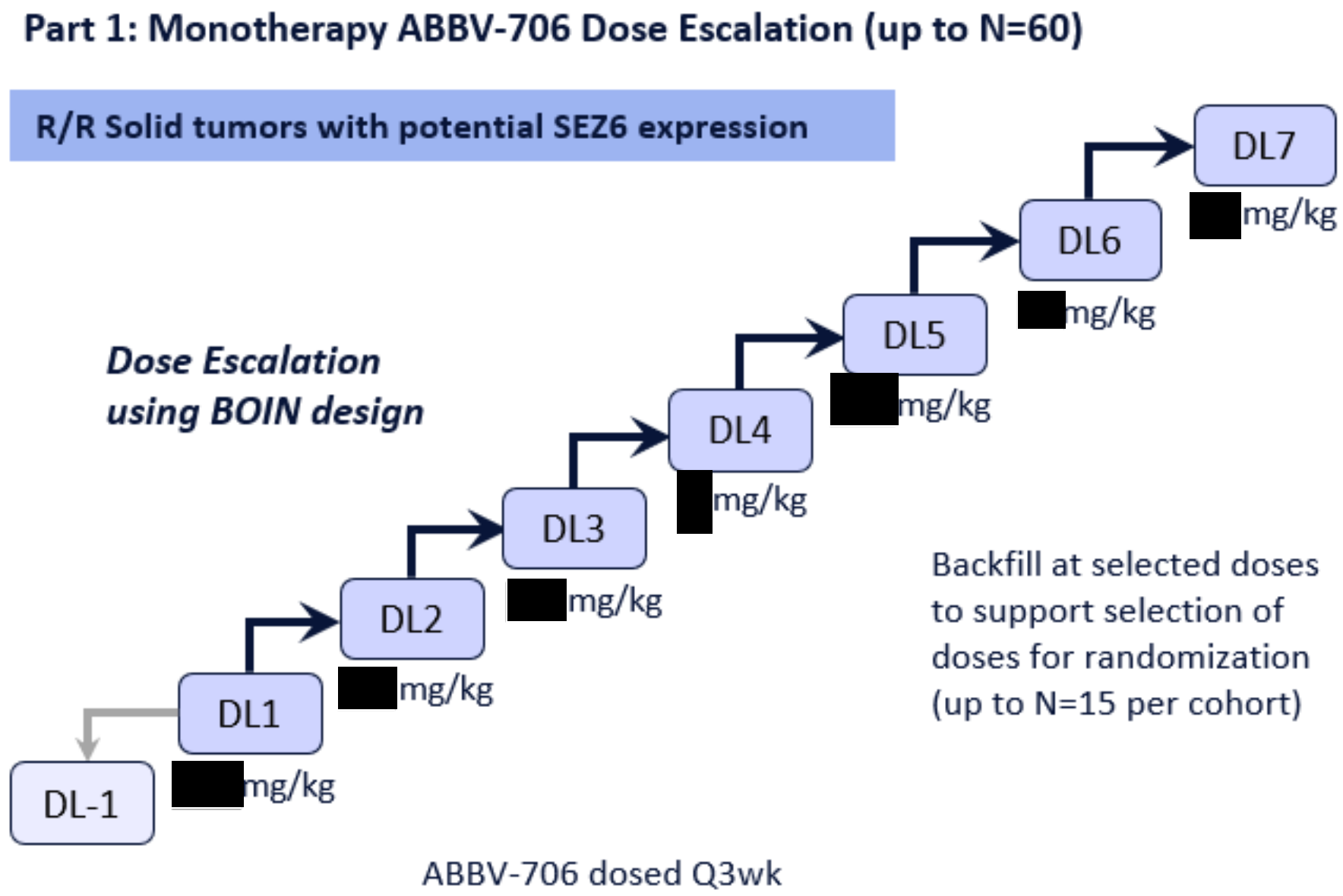

BOIN = Bayesian optimal interval; DL = dose level; [redacted] R/R = relapsed or refractory; SEZ6 = seizure-related 6 homolog

Figure 1-2 Study Design (Part 1b)

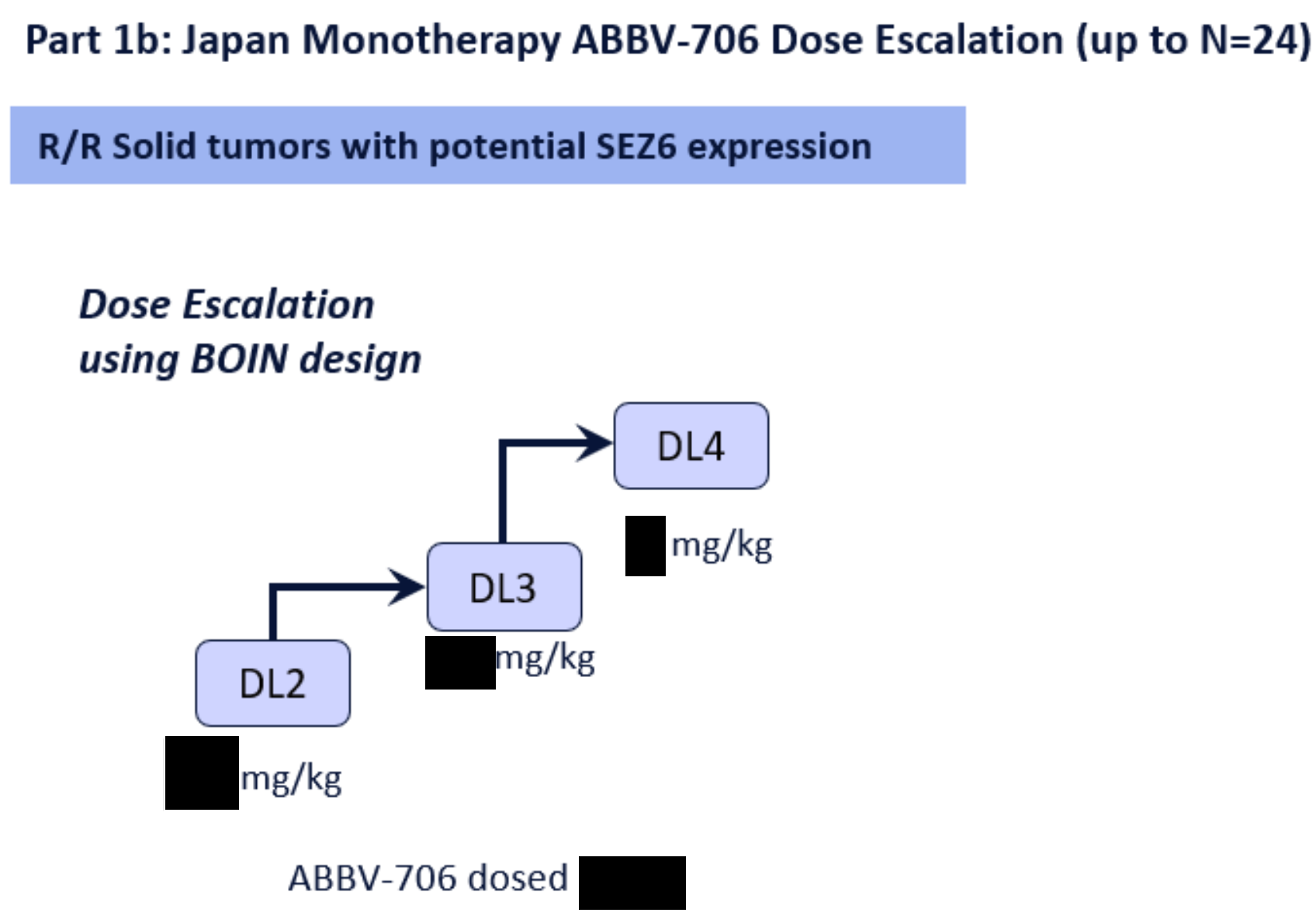

BOIN = Bayesian optimal interval; DL = dose level[redacted] R/R = relapsed or refractory; SEZ6 = seizure-related 6 homolog

Figure 1-3. Study Design (Part 2c)

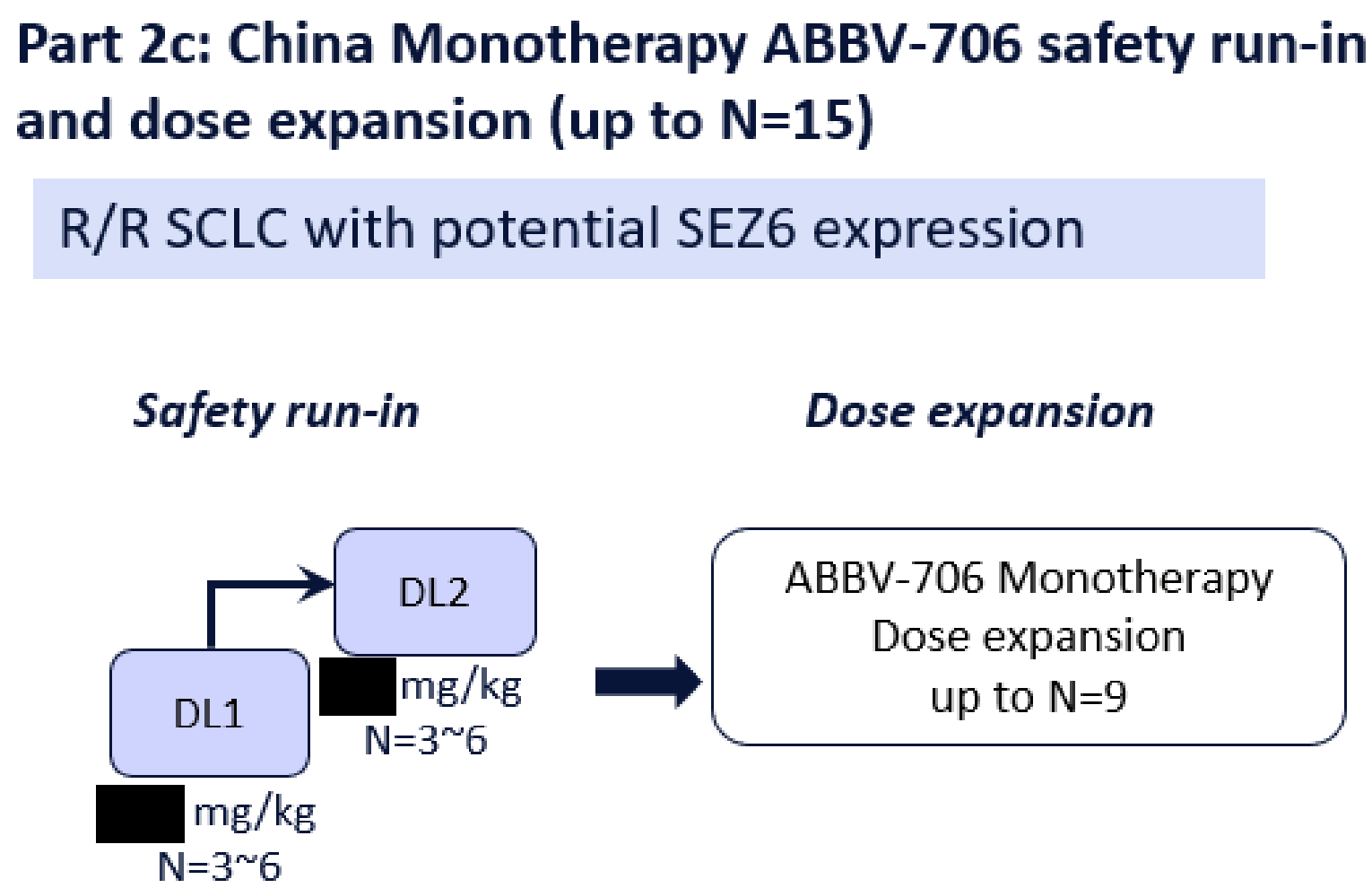

DL = dose level; R/R = relapsed or refractory; SEZ6 = seizure-related 6 homolog; SCLC = small cell lung cancer

Figure 1–4 Overall Study Schema

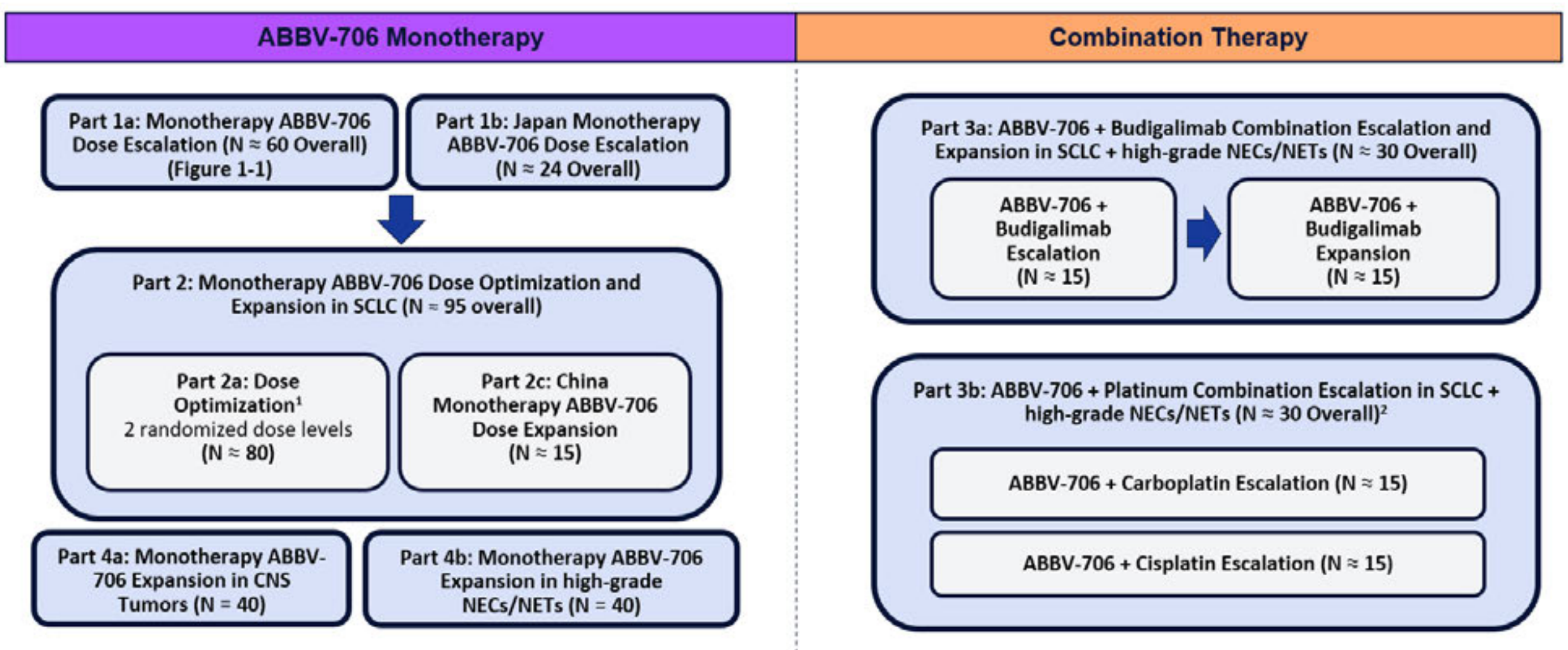

SCLC = small cell lung cancer; CNS = central nervous system; RP2D = recommended Phase 2 dose;
NECs = neuroendocrine carcinomas; NETs = neuroendocrine tumors

1. Not to exceed 40 subjects per dose level
2. Enrollment of Part 3b was halted after 18 subjects were enrolled.

1.3 Schedule of Activities (SoA)

The following tables show the required activities.

Table 1–1 Schedule of Activities: Part 1 (Monotherapy Dose Escalation)

Cycle Length: 21 days	Screening	Cycle 1					Cycle 2				Cycle 3					Section/Table
**Activity**	Day −14 to Day −1	Day 1[a]	Day 2	Day 3	Day 8 (±1 day)	Day 15 (±1 day)	Day 1[b] (+1 day)	Day 2	Day 8 (±1 day)	Day 15 (±1 day)	Day 1[b] (±1 day)	Day 2	Day 3 (+1 day)	Day 8 (±1 day)	Day 15 (±1 day)	
**INTERVIEWS & QUESTIONNAIRES**																
Subject information and informed consent	✓															8.1.1 & 8.1.2
Eligibility criteria	✓	✓														5.1 & 5.2
Medical and cancer history	✓	✓														8.1.3
Adverse event assessment	✓	✓	✓	✓	✓	✓	✓	✓	✓	✓	✓	✓	✓	✓	✓	8.5 & 10.3
Prior/concomitant therapy	✓	✓	✓	✓	✓	✓	✓	✓	✓	✓	✓	✓	✓	✓	✓	6.10 & 6.11
**LOCAL LABS & EXAMS**																
Single 12-lead ECG	✓															8.4.4
Triplicate ECG[i]		✓	✓	✓	✓	✓	✓	✓			✓	✓	✓	✓	✓	8.4.4 & 8.6.1/Table 8–1
Vital signs	✓	✓	✓	✓	✓	✓	✓	✓	✓	✓	✓	✓	✓	✓	✓	8.4.3
Height (Screening only) and weight	✓	✓					✓				✓					8.4.2
Physical examination (including neurological examination)[j]	✓	✓			✓	✓	✓		✓	✓	✓					8.4.1
Echocardiogram or MUGA	✓															8.1.5
Pregnancy test (WOCBP only; serum/urine)[c]	✓	✓					✓				✓					8.4.6
ECOG Performance Status	✓	✓					✓				✓					8.1.6
Hematology	✓	✓			✓	✓	✓		✓	✓	✓			✓	✓	8.4.5 & 10.2
Chemistry	✓	✓			✓	✓	✓		✓	✓	✓			✓	✓	8.4.5 & 10.2
Urinalysis	✓	✓					✓				✓					8.4.5 & 10.2

**Cycle Length: 21 days**	**Screening**	**Cycle 1**					**Cycle 2**				**Cycle 3**					**Section/Table**
**Activity**	**Day −14 to Day −1**	**Day 1[a]**	**Day 2**	**Day 3**	**Day 8 (±1 day)**	**Day 15 (±1 day)**	**Day 1[b] (+1 day)**	**Day 2**	**Day 8 (±1 day)**	**Day 15 (±1 day)**	**Day 1[b] (±1 day)**	**Day 2**	**Day 3 (+1 day)**	**Day 8 (±1 day)**	**Day 15 (±1 day)**	
For Japan Sites Only: Virology	✓															10.11.1
aPTT/PT/INR	✓															8.4.5 & 10.2
Tumor assessment (CT scan preferred for extracranial tumors/MRI for CNS tumors)	✓[d]															8.3
**CENTRAL LABS**																
Archive tissue or fresh biopsy for SEZ6 testing	[redacted]															
Pharmacokinetic samples[i]																
ADA and nAb samples[i]																
Peripheral blood for serum biomarkers[e]																
Peripheral blood for plasma ctDNA[e]																
Optional biomarker sample: Whole blood DNA (PG)[e]																
ABBV-706 administration																

Cycles ≥ 4 continued on next page ➡

**Cycle Length: 21 days**	**Cycles ≥4**			**Every ~6 weeks prior to dosing[f] (±7 days)**	**EOT (30 days after last dose ±15 days)**	**Safety Follow-up[g] (80 days after last dose +15 days)**	**Follow-up[h] (up to 2 years)**	**Section/Table**
**Activity**	**Day 1[b] (±2 days)**	**Day 8 (±1 day)**	**Day 15 (±1 day)**					
**INTERVIEWS & QUESTIONNAIRES**								
Adverse event assessment[k]	✓	✓	✓		✓	✓		8.5 & 10.3
Prior/concomitant therapy[k]	✓	✓	✓		✓	✓		6.10 & 6.11
Post-treatment cancer information						✓	✓	8.3
Survival status						✓	✓	7.1 & 7.3
**LOCAL LABS & EXAMS**								
Single 12-lead ECG					✓			8.4.4
Vital signs[k]	✓	✓	✓		✓			8.4.3
Weight	✓							8.4.2
Physical examination (including neurological examination)	✓				✓			8.4.1
Pregnancy test (WOCBP only; serum/urine)[c]	✓				✓			8.4.6
ECOG Performance Status	✓				✓			8.1.6
Hematology[k]	✓	✓	✓		✓			8.4.5 & 10.2
Chemistry[k]	✓	✓	✓		✓			8.4.5 & 10.2
Urinalysis	✓							8.4.5 & 10.2
Tumor assessment (CT scan preferred for extracranial tumors/MRI for CNS tumors)				✓				8.3
**CENTRAL LABS**								
Fresh tumor biopsy (optional) at progression	[redacted]							
Pharmacokinetic samples[i]								
ADA and nAb samples[i]								

Cycle Length: 21 days	Cycles ≥4			Every ~6 weeks prior to dosing[f] (±7 days)	EOT (30 days after last dose ±15 days)	Safety Follow-up[g] (80 days after last dose +15 days)	Follow-up[h] (up to 2 years)	Section/Table
**Activity**	**Day 1[b] (±2 days)**	**Day 8 (±1 day)**	**Day 15 (±1 day)**					
Peripheral blood for serum biomarkers[e]	[redacted]							
Peripheral blood for plasma ctDNA[e]	[redacted]							
**℞ TREATMENT**								
ABBV-706 administration	[redacted]							

ADA = antidrug antibody; aPTT = activated partial thromboplastin time; CNS = central nervous system; CT = computerized tomography; ctDNA = circulating tumor DNA; ECG = electrocardiogram; ECOG = Eastern Cooperative Oncology Group; EOT = end of treatment; INR = international normalized ratio; MRI = magnetic resonance imaging; MUGA = multigated acquisition; nAb = neutralizing antidrug antibody; PK = pharmacokinetic; PT = prothrombin time; SCLC = small cell lung cancer; SEZ6 = seizure-related 6 homolog; WOCBP = women of childbearing potential

a. Local laboratory assessments and exams may be performed within 1 day prior to dosing of study drug on Cycle 1 Day 1.

b. Local laboratory assessments may be performed within 1 day prior to dosing of study drug for Cycles 2 or later. The ±2 days window does not apply to non-laboratory assessments.

c. A qualitative serum pregnancy test will be performed at Screening and a urine/serum pregnancy test will be performed at baseline (or upon confinement in each period) prior to first dose of study drug for all female subjects of childbearing potential and on day 1 of every cycle.

d. Baseline tumor assessment may be performed within 28 days prior to first dose.

e. Day 1 collections on each cycle should be done predose for all the biomarkers (outside of PK/ADA).

f. Post-baseline tumor assessment will be conducted every 6 weeks (allowable window of ±7 days) from Cycle 1 Day 1 until Week 24, and every 12 weeks (±7 days) thereafter until radiographic progression, start of a new anticancer therapy, death, or subject withdrawal of consent.

g. A safety follow-up 80 days after last dose of study drug will be performed by telephone or chart review for subjects who discontinue from the study treatment unless another anticancer therapy has been started.

h. For subject who does not have documented progression by imaging, radiographic tumor assessments will continue as per the scheduled scan frequency until disease progression documented by imaging, start of a new anticancer therapy, death, or withdrawal of consent. Follow-up for subject's survival and post-treatment cancer information will be performed approximately every 3 months starting from the Safety Follow-up for up to 2 years after the last dose of study drug, or until death, or withdrawal of consent, or study closure.

i. Refer to Section 8.6.1/Table 8–1 for specific timepoints for PK/triplicate ECG/immunogenicity (ADA/nAb) sampling.

j. For Japanese subjects only, SpO2 testing and careful auscultation must be carried out on Day 1 of each cycle as part of the physical examination.

k. From Cycle 6 and onward, adverse event, prior/concomitant therapy, vital signs, hematology, and chemistry lab assessments on Day 8 and Day 15 are optional based on Investigator discretion.

Table 1–2 Schedule of Activities: Parts 2-4 (Monotherapy Dose Expansions, Combo Escalations and Expansions)

Cycle Length: 21 days	Screening	Cycle 1					Cycle 2				Cycle 3					Section/Table
**Activity**	Day –14 to day –1	Day 1[a]	Day 2[e]	Day 3[e]	Day 8 (±1 day)	Day 15 (±1 day)	Day 1[b] (+1 day)	Day 2	Day 8 (±1 day)	Day 15 (±1 day)	Day 1[b] (±1 day)	Day 2[e]	Day 3[e] (+1 day)	Day 8 (±1 day)	Day 15 (±1 day)	
**INTERVIEWS & QUESTIONNAIRES**																
Subject information and informed consent	✓															8.1.1 & 8.1.2
Eligibility criteria	✓	✓														5.1 & 5.2
Medical and cancer history	✓	✓														8.1.3
Adverse event assessment	✓	✓	✓	✓	✓	✓	✓	✓	✓	✓	✓	✓	✓	✓	✓	8.5 & 10.3
Prior/concomitant therapy	✓	✓	✓	✓	✓	✓	✓	✓	✓	✓	✓	✓	✓	✓	✓	6.10 & 6.11
**LOCAL LABS & EXAMS**																
Single 12-lead ECG (all subjects)	✓															8.4.4
Triplicate ECG[f,l] (Part 2 only, up to 30 subjects) (Not collected for subjects in mainland China)		✓	✓	✓	✓	✓	✓	✓			✓	✓	✓	✓	✓	8.4.4 & 8.6.1/Table 8–1
Single 12-lead ECG (Part 2 after first up to 30 subjects and Parts 2c, 3, and 4)		✓					✓				✓					8.4.4
Vital signs	✓	✓	✓	✓	✓	✓	✓	✓	✓	✓	✓	✓	✓	✓	✓	8.4.3
Height (Screening only) and weight	✓	✓					✓				✓					8.4.2
Physical examination (including neurological examination)[m]	✓	✓			✓	✓	✓		✓	✓	✓					8.4.1
Echocardiogram or MUGA	✓															8.1.5
Audiology assessment (Part 3b only)	✓															8.1.4

Cycle Length: 21 days	Screening	Cycle 1					Cycle 2				Cycle 3					Section/Table
**Activity**	**Day −14 to day −1**	**Day 1[a]**	**Day 2[e]**	**Day 3[e]**	**Day 8 (±1 day)**	**Day 15 (±1 day)**	**Day 1[b] (+1 day)**	**Day 2**	**Day 8 (±1 day)**	**Day 15 (±1 day)**	**Day 1[b] (±1 day)**	**Day 2[e]**	**Day 3[e] (+1 day)**	**Day 8 (±1 day)**	**Day 15 (±1 day)**	
Pregnancy test[c] (WOCBP only; serum/urine)	✓	✓					✓				✓					8.4.6
ECOG Performance Status	✓	✓					✓				✓					8.1.6
Hematology	✓	✓			✓	✓	✓		✓	✓	✓			✓	✓	8.4.5 & 10.2
Chemistry	✓	✓			✓	✓	✓		✓	✓	✓			✓	✓	8.4.5 & 10.2
Urinalysis	✓	✓					✓				✓					8.4.5 & 10.2
For Japan and China sites only: Virology	✓															10.11.2.1 & 10.11.1
For Japan sites, Part 3a only: HBA1C	✓															10.11.1
Hormone Levels (Part 3a only)	✓	✓									✓					8.4.5 & 10.2
aPTT/PT/INR	✓															8.4.5 & 10.2
Amylase and lipase[n]	✓															8.4.5 & 10.2
Tumor assessment (CT scan preferred for extracranial tumors/MRI for CNS tumors)[d]	✓															8.3
**CENTRAL LABS**																
Archive tissue or fresh biopsy for SEZ6 testing	[redacted]															
Pharmacokinetic samples[f,l]	[redacted]															
ADA and nAb samples[l]	[redacted]															
Peripheral blood for immunophenotyping[g] (Parts 2 and 3a only) (Not collected for subjects in mainland China)	[redacted]															

Cycle Length: 21 days	Screening	Cycle 1					Cycle 2				Cycle 3					Section/Table
**Activity**	**Day −14 to day −1**	**Day 1[a]**	**Day 2[e]**	**Day 3[e]**	**Day 8 (±1 day)**	**Day 15 (±1 day)**	**Day 1[b] (+1 day)**	**Day 2**	**Day 8 (±1 day)**	**Day 15 (±1 day)**	**Day 1[b] (±1 day)**	**Day 2[e]**	**Day 3[e] (+1 day)**	**Day 8 (±1 day)**	**Day 15 (±1 day)**	
Peripheral blood for serum biomarkers[g] (Not collected for subjects in mainland China)		✓			✓	✓	✓				✓					8.9
Peripheral blood for plasma ctDNA[g] (Not collected for subjects in mainland China)		✓					✓				✓					8.9
Optional biomarker sample: Whole blood DNA (PG)[g] (Not collected for subjects in mainland China)		✓														8.9
**℞ TREATMENT**																
ABBV-706 administration	[redacted]															
Budigalimab administration (Part 3a)																
Platinum administration (Part 3b)																

Cycles ≥ 4 continued on next page ➡

Cycle Length: 21 days	Cycles ≥4			Every ~6 Weeks Prior to Dosing[h] (±7 days)	EOT (30 days after last dose ±15 days)	Safety Follow-up[i] (80 [Parts 2, 3b, & 4] or 90 [Part 3a] days after last dose +15 days)	Follow-up[j] (up to 2 years)	Section
**Activity**	**Day 1[b] (±2 days)**	**Day 8 (±1 day)**	**Day 15 (±1 day)**					
**INTERVIEWS & QUESTIONNAIRES**								
Adverse event assessment[p]	✓	✓	✓		✓	✓		8.5 & 10.3
Prior/concomitant therapy[p]	✓	✓	✓		✓	✓		6.10 & 6.11
Post-treatment cancer information						✓	✓	8.3
Survival status						✓	✓	7.1 & 7.3
**LOCAL LABS & EXAMS**								
Single 12-lead ECG (all subjects)					✓			8.4.4
Single 12-lead ECG (Part 2 after first up to 30 subjects and Parts 2c, 3, and 4)	✓							8.4.4
Vital signs[p]	✓	✓	✓		✓			8.4.3
Weight	✓				✓			8.4.2
Physical examination (including neurological examination)	✓				✓			8.4.1
Pregnancy test[c] (WOCBP only; serum/urine)	✓				✓			8.4.6
ECOG Performance Status	✓				✓			8.1.6
Hematology[p]	✓	✓	✓		✓			8.4.5 & 10.2
Chemistry[p]	✓	✓	✓		✓			8.4.5 & 10.2
Urinalysis	✓							8.4.5 & 10.2
aPTT/PT/INR[o]	✓							8.4.5 & 10.2
Hormone Levels (Part 3a only)	✓[k]							8.4.5 & 10.2
Tumor assessment (CT scan preferred for extracranial tumors/MRI for CNS tumors)				✓				8.3

Cycle Length: 21 days	Cycles ≥4			Every ~6 Weeks Prior to Dosing[h] (±7 days)	EOT (30 days after last dose ±15 days)	Safety Follow-up[i] (80 [Parts 2, 3b, & 4] or 90 [Part 3a] days after last dose +15 days)	Follow-up[j] (up to 2 years)	Section
**Activity**	**Day 1[b] (±2 days)**	**Day 8 (±1 day)**	**Day 15 (±1 day)**					
**CENTRAL LABS**								
Fresh tumor biopsy (optional) at progression					✓			8.9
Pharmacokinetic samples[l]	✓				✓			8.6.1/Table 8–1
ADA and nAb samples[l]	✓				✓			8.6.1/Table 8–1
Peripheral blood for immunophenotyping[g] (Parts 2 and 3a only) (Not collected for subjects in mainland China)					✓			8.9
Peripheral blood for serum biomarkers[g] (Not collected for subjects in mainland China)	✓ Every odd cycle				✓			8.9
Peripheral blood for plasma ctDNA[g] (Not collected for subjects in mainland China)	✓ Every odd cycle				✓			8.9
**Rx TREATMENT**								
ABBV-706 administration	[redacted]							
Budigalimab administration (Part 3a)								
Carboplatin or cisplatin administration (Part 3b)								

ADA = antidrug antibody; aPTT = activated partial thromboplastin time; CNS = central nervous system; CT = computerized tomography;; ctDNA = Circulating tumor DNA; ECG = electrocardiogram; EOT = end of treatment; ECOG = Eastern Cooperative Oncology Group; INR = international normalized ratio; MRI = magnetic resonance imaging; MUGA = multigated acquisition; nAb = neutralizing antidrug antibody; PT = prothrombin time; SCLC = small cell lung cancer; WOCBP = women of childbearing potential

a. Local laboratory assessments and exams may be performed within 1 day prior to dosing of study drug on Cycle 1 Day 1.

b. Local laboratory assessments may be performed within 1 day prior to dosing of study drug for Cycles 2 or later. The ± 2 days window does not apply to non-laboratory assessments.
c. A qualitative serum pregnancy test will be performed at Screening and a urine/serum pregnancy test will be performed at baseline (or upon confinement in each period) prior to first dose of study drug for all female subjects of childbearing potential and on day 1 of every cycle.
d. Baseline tumor assessment may be performed within 28 days prior to first dose.
e. For subjects that are not required to perform triplicate ECGs and PK sample collection on Cycle 1 Day 2 and Day 3 and on Cycle 3 Day 1 and Day 2 visits, these visits are not required.
f. At the Sponsor's discretion, the totality of PK/triplicate ECG evaluations during Part 1 monotherapy dose escalation will be used to support reduction in sample collections and specific PK and triplicate ECG sampling timepoint(s) may be omitted from the ABBV-706 expansion and combination cohorts (Parts 2, 3, and 4). If elected, the Sponsor will communicate to sites PK/triplicate ECG sampling timepoints to be followed via a memo.
g. Day 1 collections on each cycle should be done predose for all the biomarkers (outside of PK/ADA).
h. Post-baseline tumor assessment will be conducted every 6 weeks (allowable window of ±7 days) from Cycle 1 Day 1 until Week 24, and every 12 weeks (±7 days) thereafter until radiographic progression, start of a new anticancer therapy, death, or subject withdrawal of consent.
i. A safety follow-up after last dose of study drug (80 days for Parts 2, 3b, and 4 or 90 days for Part 3a) will be performed by telephone or chart review for subjects who discontinue from the study treatment unless another anticancer therapy has been started.
j. For subject who does not have documented progression by imaging, radiographic tumor assessments will continue as per the scheduled scan frequency until disease progression documented by imaging, start of a new anticancer therapy, death, or withdrawal of consent. Follow-up for subject's survival and post-treatment cancer information will be performed approximately every 3 months starting from the Safety Follow-up for up to 2 years after the last dose of study drug, or until death, or withdrawal of consent, or study closure.
k. Only for every odd cycle for hormones.
l. Refer to Section 8.6.1/Table 8–1 for specific timepoints for PK/triplicate ECG/immunogenicity (ADA/nAb) sampling.
m. For Japanese subjects only, SpO_2 testing and careful auscultation must be carried out on Day 1 of each cycle as part of the physical examination.
n. Additional amylase and lipase testing can be performed during study per physician discretion as clinically indicated. If amylase and/or lipase is above 3 × ULN, radiological imaging as clinically appropriate should be performed to rule out pancreatitis.
o. Periodic testing for coagulation parameters will occur every 4 cycles, beginning with Cycle 4.
p. From Cycle 6 and onward, adverse event, prior/concomitant therapy, vital signs, hematology, and chemistry lab assessments on Day 8 and Day 15 are optional based on Investigator discretion.

2 Introduction

2.1 Study Rationale

ABBV-706 is an ADC with an anti-SEZ6 antibody and Top1 inhibitor payload. Preclinical data indicate favorable risk-benefit profile in tumors which express SEZ6. There is a large unmet medical need for more effective therapy targeting SEZ6 expressing tumors such as SCLC, high-grade CNS tumors (GBM, IDH-wildtype Grade 4; oligodendroglioma, IDH-mutant, and 1p/19q-codeleted Grade 3; astrocytoma, IDH-mutant Grade 3 or Grade 4), and diverse high-grade NECs/NETs.

2.2 Background

ADCs combine the targeting properties of monoclonal antibodies (mAbs) with the antitumor effects of potent cytotoxic drugs.[1] An advantage of ADCs is their ability to target toxic payloads to a tumor, reducing normal tissue toxicity associated with systemic exposure.

ABBV-706 is an ADC comprised of the SEZ6 targeting antibody [redacted] conjugated to AbbVie's Top1 inhibitor [redacted]. [redacted] is a humanized immunoglobulin G1 (IgG1), which is a genetically recombinant product manufactured using a Chinese hamster ovary cell line, that is also employed in ABBV-011 (ClinicalTrials.gov Identifier: NCT03639194), a calicheamicin ADC currently under clinical investigation.[2] [redacted] is a camptothecin derivative that has been previously characterized and is stably conjugated at drug-to-antibody ratio [redacted] of [redacted] via a bromoacetamide attachment and a cleavable valine-alanine linker. This linker-drug technology is identical to that used in [redacted] ABBV-706 binds to the extracellular domain of SEZ6 with high affinity: following internalization, the payload [redacted] is released and forms a ternary complex with Top1 and DNA, preventing single strand break re-ligation. Over time, the accumulation of DNA damage leads to cell cycle arrest and cell death.

SEZ6 is overexpressed in many tumor types with high unmet need, including SCLC, other neuroendocrine tumor types, and high-grade CNS tumors (GBM, IDH-wildtype Grade 4; oligodendroglioma, IDH-mutant, and 1p/19q-codeleted Grade 3; astrocytoma, IDH-mutant Grade 3 or Grade 4). This overexpression in tumors and its limited normal tissue expression suggest SEZ6 would be a good target for the development of an ADC. Top1 plays an essential role in both replication and transcription, and selective inhibitors derived from camptothecin (e.g., topotecan and irinotecan) have been approved as systemic chemotherapy in multiple cancer indications including SCLC. Although systemic Top1 inhibitors are effective cancer therapeutics, significant toxicity is observed. Toxicity associated with systemic Top1 inhibitors

supports application of ADC technology to expand therapeutic index by tumor targeted delivery of a potent camptothecin analog payload, as represented in ABBV-706.

ABBV-706 is a SEZ6 targeting Top1 inhibitor ADC that shows promising preclinical activity as a monotherapy in patient-derived xenograft models of SCLC. Preclinical testing in other SEZ6 expressing neuroendocrine and CNS tumors is ongoing.

Combinations of ABBV-706 with other agents may enhance efficacy or improve benefit in earlier lines of therapy. Platinum (carboplatin or cisplatin) plus etoposide has been the backbone of primary therapy for extensive-stage SCLC as well as for diverse poorly differentiated NECs. Recent approvals in SCLC adding immune checkpoint inhibitors (i.e., atezolizumab or durvalumab) to platinum-etoposide in the frontline setting have further extended survival.[3] Top1 inhibitors, including Top1 inhibitor-based ADCs, have been reported to demonstrate immunostimulatory activity and increased antitumor effects in combination with anti-PD-(L)1 antibodies in mouse models.[4] In this study, ABBV-706 will be combined with carboplatin or cisplatin, and with the immune checkpoint inhibitor budigalimab (anti-PD-1) to assess preliminary safety and tolerability of the combination therapy(ies).

2.3 Summary of Findings from Nonclinical Studies with Potential Clinical Relevance

ABBV-706 is an ADC comprised of a SEZ6 targeting antibody [redacted] conjugated via a cleavable linker to AbbVie's topoisomerase 1 inhibitor [redacted] ABBV-706 bound recombinant SEZ6 as measured by Biacore and an enzyme-linked immunosorbent assay [redacted] and cell surface expressed SEZ6 as measured by fluorescence-activated cell sorting [redacted] ABBV-706 treatment results in DNA damage as measured by gH2AX phosphorylation and inhibition of proliferation of SEZ6 expressing small cell lung cancer (SCLC) cells at sub-nanomolar concentration, with cytotoxicity correlated with SEZ6 expression as measured by RNA.

The in vivo antitumor activity of ABBV-706 was tested in xenograft models expressing SEZ6. ABBV-706 inhibited growth of the human SCLC cancer cell line xenografts [redacted] and patient derived xenograft [redacted] models following a single dose. ABBV-706 was highly efficacious and displayed dose-dependent efficacy, with doses [redacted] ABBV-706 efficacy was SEZ6-target dependent as evidenced by ABBV-706 treatment resulting in significantly greater efficacy than the non-targeted TOP1i-conjugated ADC.

[redacted]

[redacted]

Primary toxicities with ABBV-706 in the rat and/or monkey occurred in the gastrointestinal tract (epithelial degeneration, necrosis and ulceration), hematolymphopoietic system (myelosuppression), skin (ulceration and inflammation), reproductive tissues (testes [tubular degeneration] and uterus [epithelial cell hypertrophy and karyomegaly in endometrium]), bone (physeal disorganization), teeth (incisor degeneration), and kidney (tubular epithelial degeneration). Severe gastrointestinal toxicity in the monkey and hematolymphopoietic and skin toxicity in the rat led to mortality at the higher doses. All findings associated with ABBV-706 administration were dose-dependent, consistent with the pharmacology of the payload, and reversible with the exception of testicular and tooth degeneration in the rat that were still present at the end of the 12-week recovery period. [redacted]

The primary toxicities with budigalimab observed in the monkey consisted of reversible decreases in RBC mass accompanied by compensatory increases in reticulocytes and erythrocytic morphological and sciatic nerve degeneration that occurred only in the 4-week study and were not observed in the 13-week and 26-week studies at comparable exposures. At the NOAEL of [redacted] budigalimab in the 26-week study, exposure in monkey was [redacted] fold higher than the observed exposure of [redacted] budigalimab in human.[5]

2.4 Benefit/Risk Assessment

The benefit/risk profile of this FIH study with ABBV-706, is based on nonclinical data, including in vitro and in vivo efficacy models and Good Laboratory Practice toxicity studies in rats and monkeys. ABBV-706 exhibits potent preclinical antitumor activity across models of SCLC. ABBV-706 also demonstrates a favorable binding ratio of tumor to non-tumor tissue and will be used in subjects with advanced SCLC, high-grade CNS tumors (GBM, IDH-wildtype Grade 4; oligodendroglioma, IDH-mutant, and 1p/19q-codeleted Grade 3; astrocytoma, IDH-mutant Grade 3 or Grade 4), and diverse high-grade NECs/NETs with the goal of eliciting a robust antitumor response. [redacted]

The nonclinical pharmacology, toxicology, and PK profiles of ABBV-706 have been adequately characterized to support an acceptable rationale and risk/benefit profile for treating adult subjects with solid tumors with ABBV-706 within the context of a clinical trial.

For additional details, please refer to the ABBV-706 Investigator's Brochure.[6]

Budigalimab is an anti-PD-1 inhibitor and is currently being investigated in Phase 1 and Phase 1b/2 studies being conducted by AbbVie. Preliminary safety data are available for 143 subjects treated with budigalimab as monotherapy and preliminary safety data are also available for 408 subjects treated in the budigalimab combination with other agents. Based on safety, PK, and pharmacodynamic data, [redacted] was previously identified as the RP2D for budigalimab. Available Phase 1 budigalimab monotherapy data supports its development for the treatment of solid tumors. These data show safety profiles consistent with PD-1 inhibition and toxicity profiles similar to other approved anti-PD-1 inhibitors. The PK for budigalimab was approximately dose proportional with complete (> 95%) PD-1 receptor saturation within 2 hours postdosing and sustained saturation throughout the dosing interval.[7] Known risks of PD-1 inhibitors, such as immune-related AEs (irAEs), will be monitored throughout the study. For additional details, please refer to the budigalimab Investigator's Brochure.[8]

3 Objectives and Endpoints

Table 3–1 Objectives, Endpoints, and Estimands

Objectives	Endpoints
**Primary**	
To evaluate the safety and tolerability of ABBV-706 administered as monotherapy and in combination with either budigalimab, carboplatin, or cisplatin	Safety evaluations include AEs, vital sign measurements, electrocardiogram (ECG) variables, and clinical laboratory tests (hematology, chemistry, and urinalysis) as measures of safety and tolerability for the entire study duration.
To evaluate the pharmacokinetics (PK) and immunogenicity of ABBV-706 administered as monotherapy and in combination with either budigalimab, carboplatin, or cisplatin	Pharmacokinetic parameters, including the maximum observed serum (or plasma, for payload) concentration (C_{max}), the time to C_{max} (T_{max}), terminal phase elimination half-life ($t_{1/2}$), and the area under the serum/plasma concentration-time curve (AUC) will be calculated using noncompartmental methods for ABBV-706 total antibody, ADC, and unconjugated Top1 inhibitor payload.<br><br>Additional parameters may be calculated and/or analyses may be conducted if useful in the interpretation of the data.<br><br>Antidrug antibodies (ADAs) and neutralizing antibodies (nAbs) will be determined, as appropriate.
To determine the recommended Phase 2 dose (RP2D) of ABBV-706 as monotherapy	The RP2D will be determined using all available information, including, but not limited to, AEs, dose-limiting toxicities, PK parameters, clinical laboratory tests, and efficacy measures.
To evaluate the antitumor activity of ABBV-706 in subjects with R/R SCLC, high-grade CNS tumors (GBM, IDH-wildtype Grade 4; oligodendroglioma, IDH-mutant, and 1p/19q-codeleted Grade 3; astrocytoma, IDH-mutant Grade 3 or Grade 4) and high-grade NECs/NETs	Confirmed CR/PR as assessed by investigators per RECIST, version 1.1 for extracranial solid tumors and RANO for subjects with CNS tumors.<br><br>DOR for subjects with confirmed CR/PR.

Objectives	Endpoints
**Exploratory/Other**	
To evaluate the clinical benefit rate, PFS, and OS of ABBV-706 in subjects with R/R SCLC, high-grade CNS tumors (GBM, IDH-wildtype Grade 4; oligodendroglioma, IDH-mutant, and 1p/19q-codeleted Grade 3; astrocytoma, IDH-mutant Grade 3 or Grade 4), and high-grade NECs/NETs	Clinical benefit defined as CR/PR, SD.<br>PFS.<br>OS.
To evaluate pharmacodynamic and predictive biomarkers for association with PK, safety, and efficacy measurements<br>For the China study cohort: To evaluate SEZ6 target expression in tumor tissue measured by IHC and RNAseq for its association with PK, safety, and antitumor activity.	Biomarker Endpoints:<br>Biospecimens will be collected to investigate known / novel biomarkers.<br>Measurement of potential predictive biomarkers and their association with PK, safety, and antitumor activity.<br>Measurement of the effect of ABBV-706 on target engagement, pharmacodynamics, and disease burden.<br>For the China study cohort, SEZ6 expression in tumor tissue will be measured by IHC and RNAseq.
To evaluate the potential effects of ABBV-706 monotherapy on QT/QTc interval prolongation	QT/QTc interval and change from baseline.

4 Study Design

Phase 1, open-label, dose escalation, dose expansion, and biomarker/PK/pharmacodynamic study and proof of concept. The total number of subjects will be approximately 319. The duration of the study for each subject will be approximately 4 years and will include a 14-day screening period followed by an approximately 2-year treatment period and up to 2-year follow-up after the last dose of study drug.

4.1 Overall Design

4.1.1 Part 1 (Monotherapy Dose Escalation)

4.1.1.1 Part 1a (Monotherapy Dose Escalation)

In Part 1a (Monotherapy Dose Escalation), approximately 60 subjects with advanced solid tumors including subjects diagnosed with SCLC, high-grade CNS tumors (GBM, IDH-wildtype

Grade 4; oligodendroglioma, IDH-mutant, and 1p/19q-codeleted Grade 3; astrocytoma, IDH-mutant Grade 3 or Grade 4), and high-grade NECs/NETs (e.g. NEPC, GEP-NECs, LCNECs, SCLC transformed from EGFR mutant NSCLC, MTC, and other NECs or Grade 3 NETs [atypical lung carcinoids that have received prior chemotherapy are allowed]) will be enrolled to evaluate the safety, tolerability, and PK of ABBV-706.

Subjects will receive ABBV-706 monotherapy [redacted] in [redacted] following a Bayesian optimal interval (BOIN) dose escalation design. Enrollment of additional subjects at intermediate or lower dose levels during dose escalation may be employed to identify doses for further optimization in Part 2. At the Sponsor's discretion, escalation cohorts may be restricted to specific tumor types.

4.1.1.2 Part 1b (Japan Only)

In Part 1b (Monotherapy Dose Escalation for Japan), up to 24 subjects in Japan with advanced solid tumors including subjects diagnosed with SCLC, high-grade CNS tumors (GBM, IDH-wildtype Grade 4; oligodendroglioma, IDH-mutant, and 1p/19q-codeleted Grade 3; astrocytoma, IDH-mutant Grade 3 or Grade 4), and high-grade NECs/NETs (e.g., NEPC, GEP-NECs, LCNECs, SCLC transformed from EGFR mutant NSCLC, MTC, and other NECs or Grade 3 NETs [atypical lung carcinoids that have received prior chemotherapy are allowed]) will be enrolled to evaluate the safety, tolerability, and PK of ABBV-706.

Subjects will receive ABBV-706 monotherapy administered [redacted]

A BOIN dose escalation method will be utilized following the same rules as the escalation in Part 1a.

After Part 1b is complete, Japan subjects may enroll in other ongoing parts of the study at doses at or below the Part 1b MTD/MAD.

4.1.2 Part 2 (Monotherapy Dose Optimization and Expansion in R/R SCLC)

In Part 2 (Monotherapy Dose Optimization and Expansion in R/R SCLC), up to 95 subjects with R/R SCLC will be enrolled.

4.1.2.1 Part 2a (Monotherapy Dose Optimization)

In Part 2a, subjects will be randomized between two or more dose levels of ABBV-706 to select an RP2D with no more than 40 subjects per dose level.

4.1.2.2 Part 2c (Monotherapy Safety Run-in and Dose Expansion for China)

In Part 2c (Monotherapy safety run-in and dose expansion for China), a total of approximately 15 subjects in China diagnosed with R/R SCLC will be enrolled to evaluate the safety, tolerability, and PK of ABBV-706 administered as monotherapy.

Subjects will receive ABBV-706 monotherapy administered IV [redacted]

Guided by the cumulative global data generated from Part 1 monotherapy dose escalation, the China-specific monotherapy cohort will be conducted at two planned doses [redacted] based on comprehensive assessment. The China-specific cohort will start from [redacted] as described below. Tolerability at each dose level in Chinese subjects will be assessed in at least 3 subjects.

Tolerability and toxicity will be assessed using DLT equivalent events which will have the same definition as the DLTs used in Part 1.

Dose level toxicity equivalent events will be assessed during the first cycle of dosing with a period of [redacted] days. Dose level toxicity assessment rules, including the DLT definition, will follow the same rules as Part 1 dose escalation (Section 4.1.5).

Safety will be assessed by the SMC, and subject enrollment will occur, as follows, with no more than 15 Chinese subjects enrolled in Part 2c totally:

- If no DLTs are observed in the first 3 DLT evaluable Chinese subjects, DL1 will be considered tolerable and then escalate to DL2. If 1 DLT is observed in the first 3 DLT evaluable Chinese subjects at DL1, an additional 3 Chinese subjects may be enrolled. If only 1 subject out of the total 6 subjects experience a DLT, then DL1 will be determined as tolerable, and then escalate to DL2. An additional 3 Chinese subjects may be enrolled at DL2. Subject screening will be paused after the 3rd subject from each cohort is enrolled at each dose level and then restarted if there are no DLTs in the first 3 subjects.
    - If no DLTs are observed in the first 3 DLT evaluable subjects at DL2, then additional subjects may be enrolled in the dose expansion phase for a total of 15 subjects.
    - If 1 DLT is observed in the first 3 DLT evaluable Chinese subjects at DL2, additional 3 Chinese subjects may be enrolled at DL2. If only 1 subject out of the total 6 subjects at DL2 experiences a DLT, DL2 will be considered tolerable. Additional subjects may be enrolled in dose expansion phase for a total of 15 subjects.
- If 2 or more DLTs are observed out of either the initial 3 or 6 subjects at DL1, the China specific safety run- in phase will be paused while AbbVie and the investigators will review the available data to determine if additional subjects could be enrolled with a dose reduction or if the study should not continue with enrollment in China.

- If DL1 was determined as tolerable, and 2 or more DLTs are observed out of either the initial 3 or 6 subjects at DL2, the China specific safety run-in phase will be paused while AbbVie and the investigators review the available data to determine if additional subjects could be enrolled at an intermediate dose level according to the totality of all available data or if the study should not continue with enrollment in China.

At the sponsor's discretion, subjects in the dose expansion part may be dosed at a selected dose determined from Part 2a (the dose will not exceed the highest tolerable dose determined in the safety run-in phase of Part 2c). Dose expansion safety rules as Section 4.1.10 apply to dose expansion part in Chinese subjects in Part 2c. After the Part 2c safety run-in is complete, Chinese subjects may enroll in other parts of the study at the dose level lower than or equal to the highest tolerable dose that has been determined in the safety run-in phase of Part 2c.

4.1.3 Part 3 (Combination Dose Escalation and Expansion)

In Part 3, ABBV-706 will be combined with budigalimab (anti-PD-1), carboplatin, or cisplatin.

4.1.3.1 Part 3a (ABBV-706 and Budigalimab Combination Dose Escalation and Expansion)

In Part 3a (ABBV-706 and Budigalimab Combination Dose Escalation), approximately 30 subjects with R/R SCLC or high-grade NECs/NETs (specific tumor types include, but not limited to, NEPC, GEP-NECs, LCNECs, SCLC transformed from EGFR mutant NSCLC, MTC, and other NECs [atypical lung carcinoids that have received prior chemotherapy are allowed]) will be enrolled. The combination cohorts of ABBV-706 and budigalimab will be explored using the same BOIN dose escalation rules as Part 1 but starting at least 1 dose level below the highest dose selected for the Part 2 dose randomization cohorts and escalating no higher than that highest chosen monotherapy dose. Each ABBV-706 dose level will be administered with a fixed IV dose of budigalimab at [redacted]. Part 3a dose expansion will proceed with a dose of ABBV-706 at or below the highest dose that clears the DLT evaluation in combination escalation and will not exceed the highest monotherapy dose selected for Part 2a dose optimization.

4.1.3.2 Part 3b (ABBV-706 and Platinum Chemotherapy Combination Dose Escalation)

In Part 3b (ABBV-706 and Platinum Chemotherapy Combination Dose Escalation), up to 30 subjects with R/R SCLC or high-grade NECs/NETs (specific tumor types include, but not limited to, NEPC, GEP-NECs, LCNECs, SCLC transformed from EGFR mutant NSCLC, MTC, and other NECs [atypical lung carcinoids that have received prior chemotherapy are allowed]) will be enrolled. The combination cohorts of ABBV-706 and platinum chemotherapy will be explored using the same BOIN dose escalation rules for ABBV-706 as Part 1 but starting at least

1 dose level below the highest ABBV-706 dose selected for the Part 2 dose randomization cohorts and escalating no higher than that highest chosen monotherapy dose. Separate dose escalations or de-escalations will be performed for ABBV-706 in combination with carboplatin and ABBV-706 in combination with cisplatin and will proceed independently of each other. Each ABBV-706 dose level will be combined with platinum chemotherapy administered in accordance with SoC guidelines (i.e., carboplatin AUC 5 or cisplatin 75 mg/m^2 on Day 1 of each 21-day cycle). Platinum chemotherapy will be administered in accordance with SoC guidelines (i.e., carboplatin AUC 5 or cisplatin 75 mg/m^2 on day 1 of each 21-day cycle). Initial assignment to cisplatin or carboplatin will be left to the treating physicians' discretion but subjects may not change platinum combination partner while on study.

4.1.4 Part 4 (ABBV-706 Monotherapy Dose Expansion)

4.1.4.1 Part 4a (ABBV-706 Monotherapy Dose Expansion in R/R CNS Tumors)

In Part 4a (ABBV-706 Monotherapy Dose Expansion in R/R CNS tumors), approximately 40 subjects with R/R high-grade CNS tumors (GBM, IDH-wildtype Grade 4; oligodendroglioma, IDH-mutant, and 1p/19q-codeleted Grade 3; astrocytoma, IDH-mutant Grade 3 or Grade 4) will be enrolled at or below the monotherapy MTD/MAD from the dose escalation in Part 1a.

4.1.4.2 Part 4b (ABBV-706 Monotherapy Dose Expansion in R/R high-grade NECs/NETs)

In Part 4b (ABBV-706 Monotherapy Dose Expansion in R/R high-grade NECs/NETs), approximately 40 subjects with R/R high-grade NECs/NETs (specific tumor types include, but not limited to, NEPC, GEP-NECs, LCNECs, SCLC transformed from EGFR mutant NSCLC, MTC, and other NECs [atypical lung carcinoids that have received prior chemotherapy are allowed]) will be enrolled at or below the monotherapy MTD/MAD from the dose escalation in Part 1.

4.1.5 Dose Escalation Guidelines

[redacted]

[redacted]

Table 4–1 Planned Dose Levels

Cohort	Dose 1	Dose 2	Dose 3	Dose 4	Dose 5	Dose 6	Dose 7
Dose Level	[redacted] mg/kg	[redacted] mg/kg	[redacted] mg/kg	[redacted] mg/kg	[redacted] mg/kg	[redacted] mg/kg	[redacted] mg/kg

Dose escalation of ABBV-706 will be implemented using the BOIN design with the following parameters set:

- The target DLT rate is 30%.
- A true DLT rate of 0.6 × the target rate (= 18%) or lower is deemed potentially subtherapeutic.
- A true DLT rate of 1.227 × the target rate (= 36.8%) or higher is deemed overly toxic.
- Using non-informative prior, the pre-specified optimal escalation and de-escalation boundaries for the observed toxicity rate are 23.6% and 33.3%, respectively.
- If there is a higher than 95% posterior probability that the true toxicity rate associated with the current dose level is above the target toxicity rate, then that dose level and higher dose levels will be eliminated.
- A maximum of 15 DLT evaluable subjects can be enrolled at a single dose level.
- A maximum of 60 DLT evaluable subjects can be enrolled overall in the monotherapy dose escalation and 20 in the combination therapy dose escalations.

These boundaries produce the decision rules based on the evaluable subjects and cumulative number of subjects who experience a DLT at current dose level shown in Table 4–2.

Table 4–2 BOIN Dose Escalation Rules for M23-385

Treated Patients	**3**	4	5	**6**	7	8	**9**	10	11	**12**	13	14	**15**
Escalate if # DLT ≤	**0**	0	1	**1**	1	1	**2**	2	2	**2**	3	3	**3**
Expand if # DLT in	**1**	1	-	**2**	2	2	**3**	3	3	**3-4**	4	4	**4-5**
De-escalate if # DLT ≥	**2**	2	2	**3**	3	3	**4**	4	4	**5**	5	5	**6**
Eliminate if # of DLT ≥	**3**	3	4	**4**	5	5	**5**	6	6	**7**	7	8	**8**

At the Sponsor's discretion, escalation cohorts may be restricted to specific tumor types.

Dose Escalation for Part 1b (Japan only)

Dose escalation for Part 1b will follow the same dose escalation rules as Part 1a with the following differences:

- Dose escalation will start at [redacted] mg/kg.
- Doses levels eliminated in Part 1a will not be explored.
- Dose level will not exceed those explored in Part 1a.
- A Maximum 9 DLT evaluable subjects may be enrolled at a dose level
- A maximum of 24 DLT evaluable subjects may be enrolled.
- Escalate or expand decisions under BOIN will be subject to clinical review. The decision may be made to take a more conservative action instead.

Tolerability in Japanese subjects can be assessed in at least 3 subjects. A dose will be considered tolerable if it meets the criteria for "escalate" in the BOIN dose escalation rules or 3 DLTs observed in 9 evaluable subjects at the dose level. These results will be comprehensively evaluated by the SMC together with the results of Part 1a. Once tolerability in Japanese subjects is established, Japanese subject may be enrolled in other parts of the study at or below the highest tolerable dose.

4.1.6 Safety Monitoring Committee

All dose escalation decisions will be made by the Sponsor in consultation with the SMC. Safety will be monitored by the SMC consisting of, at a minimum, the Sponsor medical monitor, study investigators, a Sponsor safety physician, a clinical pharmacologist, and the study biostatistician.

The SMC will meet routinely to review the emerging safety, PK, and pharmacodynamic data throughout the study, and to make dose escalation recommendations during Part 1, to make safety assessments based on DLT equivalent events in Part 2c, and to make starting dose and dose escalation recommendations for Parts 3a and 3b. The SMC will also review safety data that may emerge due to cumulative exposure beyond the DLT period. The process for dose escalation decisions and the roles and responsibilities of the SMC will be detailed further in the SMC charter.

4.1.7 Interstitial Lung Disease Adjudication Committee

An ILD Adjudication Committee will review cases of potential ILD/pneumonitis. To ensure adequate evaluation, relevant additional data from within the clinical database may be provided to the adjudication committee to fully characterize medical history (e.g., smoking, radiation and pulmonary history), diagnostic evaluation, treatment, and outcome of the event. Further details can be found in the ILD Adjudication Charter.

4.1.8 Backfill Guidelines

To guide the selection of doses for Part 2, additional subjects may be enrolled at dose levels below the current head cohort of the BOIN escalation, but still limited to the total escalation sample size limits. A dose level may enroll additional subjects at the Sponsor's discretion as long as at least 6 subjects are DLT evaluable at higher dose levels and the dose level directly above the target level does not require de-escalation per the BOIN rules. At the Sponsor's discretion, these additional subjects may be restricted to specific tumor types. The additional subjects will be monitored for safety but will only impact the BOIN escalation if either the elimination threshold is reached or the active cohort of the escalation returns to that dose level.

4.1.9 Selection of Maximum Tolerable Dose

The MTD will be selected as the highest allocated dose for which the BOIN dose escalation rule does not require de-escalation and at least 6 subjects have completed the DLT period. This selection procedure ensures consistency between the BOIN dose escalation rule and the selected dose (i.e., the selected dose cannot be a dose for which the observed toxicity rate yields a de-escalation decision under the BOIN design). If none of the tested doses satisfy these criteria or the lowest dose is eliminated, then no MTD is identified. The Sponsor may choose dose levels lower than MTD/MAD to explore further in Part 2.

At the discretion of the Sponsor, additional dose levels of ABBV-706 may be explored but will continue to follow the same BOIN escalation/de-escalation and additional subjects will be enrolled in the respective cohorts as required by BOIN rules. If the BOIN rules recommend dose de-escalation, the next dose evaluated could be an intermediate dose less than the current dose.

Additionally, dose cohort '-1' can be explored at the Sponsor's discretion if the decision is to de-escalate at dose cohort '1'. Dose escalation steps beyond [redacted] mg/kg may also be added if MTD has not been reached. These will not exceed a 25% increase in dose.

4.1.10 Dose Expansion Safety Rules

For dose optimization, expansion cohorts, and backfill cohorts, the following dose expansion safety rules apply:

- Any occurrence of the same Grade 4 non-hematologic, treatment-related AE in 3 or more subjects will halt new subject enrollment until the SMC (refer to Protocol Section 4.1.6 for details) can review all relevant safety data and provide a recommendation on the relationship to treatment, any necessary risk mitigation measures, and when enrollment can resume.
- Any occurrence of a treatment-related death in 2 or more subjects will halt new subject enrollment until the SMC can review all relevant safety data and provide a recommendation on the relationship to treatment, any necessary risk mitigation measures, and when enrollment can resume.

4.2 Scientific Rationale for Study Design

This FIH study with an initial dose escalation phase followed by 3 additional subject cohorts with cohort-specific objectives is designed to determine pharmacologic profile and PK characteristics of ABBV-706 in humans, to understand the side effects associated with increasing doses, and to gain early evidence of antitumor activity. Dose escalation of ABBV-706 will be implemented using the BOIN design.[9] This design provides much more flexibility in choosing the target toxicity rate and cohort size and yields a substantially better performance. Consideration was given to the current Food and Drug Administration Guidance for Industry on the use of expansion cohorts in FIH studies.[10] The objectives of the 3 expansion cohorts include assessment of antitumor activity in a disease-specific setting, assessment of a dose with acceptable safety in subjects with specific tumor types, establishment of dose and schedule for ABBV-706 administered with other oncology drugs, and the evaluation of the predictive value of potential biomarkers.

4.2.1 Appropriateness of Measurements

Standard statistical, clinical, and laboratory procedures will be utilized in this study. All efficacy and safety-related measurements in this study are standard for assessing disease activity in subjects with solid tumors. All clinical and laboratory procedures in this study are standard and generally accepted.

4.2.2 Suitability of Subject Population

ABBV-706 is an ADC with an anti-SEZ6 antibody and Top1 inhibitor payload. Preclinical data indicate favorable responses in tumors that express SEZ6. Therefore, the subject population chosen for this study will include approximately 84 subjects with solid tumors for the dose escalation ABBV-706 monotherapy (Part 1), up to 135 subjects with R/R SCLC for the dose optimization and expansion ABBV-706 monotherapy (Part 2), approximately 30 subjects with R/R SCLC or high-grade NECs/NETs (specific tumor types include, but not limited to, NEPC, GEP-NECs, LCNECs, SCLC transformed from EGFR mutant NSCLC, MTC, and other NECs [atypical lung carcinoids that have received prior chemotherapy are allowed]) for the combination dose escalation and expansion ABBV-706 combination therapies (Part 3), and approximately 40 subjects for with R/R high-grade CNS tumors (GBM, IDH-wildtype Grade 4; oligodendroglioma, IDH-mutant, and 1p/19q-codeleted Grade 3; astrocytoma, IDH-mutant Grade 3 or Grade 4) and 40 subjects with R/R high-grade NECs/NETs (specific tumor types include, but not limited to, NEPC, GEP-NECs, LCNECs, SCLC transformed from EGFR mutant NSCLC, MTC, and other NECs [atypical lung carcinoids that have received prior chemotherapy are allowed]) for additional ABBV-706 monotherapy dose expansions (Part 4).

This is the subject population most likely to benefit from ABBV-706 therapy. As preclinical data and clinical data emerge, the spectrum of indications and the biomarker cutoffs will be reviewed and possibly modified.

4.3 Justification for Dose

The starting dose selected in this study was based on extrapolation of preclinical efficacy models and analysis of PK, pharmacodynamic, and toxicity data from animal models. [redacted]

Based on the totality of clinical safety, efficacy, and PK data from Part 1, [redacted] Furthermore, based on the accumulated clinical safety and efficacy data, [redacted] have been shown to be safe and tolerable and may provide clinical anti-cancer activity in subjects with R/R SCLC. Since the objective of Part 2c is to collect safety/tolerability and PK

data in Chinese subjects, the same two dosing regimens being evaluated in Part 2a have been selected for Part 2c (China only) for initial safety run in. [redacted]. In Part 2c dose expansion, ABBV-706 will be administered at a selected dose determined from Part 2a (the dose will not exceed the highest tolerable dose determined in the safety run-in phase of Part 2c).

In Part 3a, budigalimab will be administered at a fixed dose of [redacted] which was the RP2D previously identified based on available safety, PK, and pharmacodynamic data. In Part 3b, cisplatin and carboplatin will be administered at 75 mg/m^2 and AUC 5, respectively, consistent with SoC guidelines.

4.4 End-of-Study Definition

The end-of-study is defined as the date of the last subject's last visit or date of the last follow-up contact, in the study globally, whichever is later.

A participant is considered to have completed the study if the participant has completed all periods of the study including the last scheduled procedure shown in the SoA Table 1–1 and Table 1–2 (whichever is applicable).

5 Study Population

5.1 Eligibility Criteria for Parts 1, 2, 3, and 4

Subjects must meet all of the following criteria in order to be included in the study. Sponsor cannot provide waivers to these criteria. These criteria must continue to be met by the subject prior to the first dose of study treatment.

Consent

1. Subjects or their legally authorized representative must voluntarily sign and date an informed consent, approved by an independent ethics committee (IEC)/institutional review board (IRB), prior to the initiation of any screening or study-specific procedures.
2. Subjects are willing and able to comply with procedures required in this protocol.

Demographic and Laboratory Assessments:

3. Age ≥18 years.
4. ECOG performance status (PS) of 0 or 1.
5. The following laboratory values criteria must be met within 7 days prior to the first dose of study drug:
   - ALT and AST ≤ 3.0 × the upper limit of normal (ULN).
   - For subjects with liver metastases: ALT and AST ≤ 5.0 × ULN

- Total bilirubin ≤ 1.5 × ULN. (Subjects with documented Gilbert's syndrome may have a total bilirubin ≤ 3 × ULN).
- Creatinine clearance ≥ 30 mL/min for subjects in Part 1, Part 2, Part 3a, and Part 4 and > 60 mL/min for subjects in Part 3b calculated using the Modification of Diet in Renal Disease (MDRD) equation (refer to Appendix 5, Section 10.5 for equation).
- Absolute neutrophil count ≥ 1,500/mm^3 (with no granulocyte-colony stimulating factor [G-CSF] in the past 10 days).
- Platelet count ≥ 100,000/µL (with no platelet transfusion in the past 7 days).
- Hemoglobin ≥ 8 g/dL (with no RBC transfusion in the past 7 days).

6. QT interval corrected for heart rate (QTc) ≤ 450 msec (males) or ≤ 470 msec (females) using Fridericia's correction, and an ejection fraction of ≥ 50% as measured by echocardiogram or MUGA scan at Screening.

Disease Activity:

7. Measurable disease per RECIST version 1.1 for subjects with extracranial solid tumors or RANO for subjects with primary high-grade CNS tumors (GBM, IDH-wildtype Grade 4; oligodendroglioma, IDH-mutant, and 1p/19q-codeleted Grade 3; astrocytoma, IDH-mutant Grade 3 or Grade 4).

8. Primary CNS tumors within 12 weeks from radiation therapy should have unequivocal progression as documented by either tumor recurrence predominantly outside of radiation field on MRI or confirmed on tumor biopsy.

9. Subjects with brain metastases from an extracranial solid tumor are eligible if the brain metastases are:

   - Previously treated, currently stable and not requiring steroids or anticonvulsants to control associated symptoms.
   - Untreated, asymptomatic, with fewer than 5 lesions and less than 5 mm in size each and not requiring steroids or anticonvulsants to control associated symptoms.

10. Fresh or archival tumor tissue available for submission, for retrospective SEZ6 expression analysis, as per tissue submission guidelines in the Laboratory Manual.

    - Subjects that have previously provided tissue sampling for SEZ6 testing in other AbbVie studies and meet the sampling requirements outlined by the Sponsor will not be required to provide further tissue samples for this study.
    - If archival tissue is not available and obtaining a fresh biopsy poses significant risk to the safety of the subject, the treating physician can discuss the case with the medical monitor or Sponsor to determine eligibility.

11. Subjects must have one of the following histologically or cytologically confirmed advanced relapsed or refractory solid tumors:

- **Part 1 only**: Solid tumors with potential SEZ6 expression including (but not limited to) SCLC, high-grade CNS tumors (GBM, IDH-wildtype Grade 4; oligodendroglioma, IDH-mutant, and 1p/19q-codeleted Grade 3; astrocytoma, IDH-mutant Grade 3 or Grade 4), high-grade NECs/NETs (e.g. NEPC, GEP-NECs, LCNECs, SCLC transformed from EGFR mutant NSCLC, MTC, and other NECs or Grade 3 NETs [atypical lung carcinoids that have received prior chemotherapy are allowed])
- **Part 2 only**: SCLC
- **Part 3a only**: SCLC or Grade 3 NETs and poorly differentiated NECs. Specific tumor types include but not limited to NEPC, GEP-NECs, LCNECs, SCLC transformed from EGFR mutant NSCLC, MTC, and other NECs (atypical lung carcinoids that have received prior chemotherapy are allowed)
- **Part 3b only**: SCLC who have only progressed following a frontline regimen containing a platinum-based chemotherapy (i.e., second-line SCLC subjects) or Grade 3 NETs and poorly differentiated NECs. Specific tumor types include but not limited to NEPC, GEP-NECs, LCNECs, SCLC transformed from EGFR mutant NSCLC, MTC, and other NECs (atypical lung carcinoids that have received prior chemotherapy are allowed)
- **Part 4a only**: High-grade CNS tumors
- **Part 4b only**: Grade 3 NETs and poorly differentiated NECs. Specific tumor types include but not limited to NEPC, GEP-NECs, LCNECs, SCLC transformed from EGFR mutant NSCLC, MTC, and other NECs (atypical lung carcinoids that have received prior chemotherapy are allowed)

12. Subjects must have progressed on or after standard of care (SoC) therapy as follows and with no curative therapy available (subjects who are considered ineligible for or are intolerant of SoC therapy are eligible):

- For SCLC, subjects must have relapsed or refractory disease following at least 1 prior platinum-containing chemotherapy. In countries where SoC first line systemic treatment includes platinum containing chemotherapy in combination with PD-L1/PD-1 inhibitor, it is required that patients have failed or ineligible to PD-L1/PD-1 inhibitor as part of their first line systemic treatment. For the purposes of this study, a line of therapy is defined as ≥ 1 complete cycle of either a single agent or combination of drugs, including any planned sequential therapy of various regimens (e.g., 4 cycles of initial therapy with carboplatin-etoposide and atezolizumab or durvalumab followed by maintenance with atezolizumab or durvalumab is considered first line). For Part 1b, subjects must have previously been treated with topotecan hydrochloride or amrubicin hydrochloride in addition to platinum-based chemotherapy, or subjects are not eligible for these SoC therapies.

- For high-grade CNS tumors, subjects must have had prior surgical resection, received prior radiation, and temozolomide or procarbazine, lomustine, and vincristine (PCV).
- For eligible NECs and NETs, subjects must have received and/or considered all available approved therapeutic options, if applicable, prior to enrolling on the study.

Concomitant Medications:

13. Must not have received any investigational drug or anticancer therapy including chemotherapy, radiation therapy, immunotherapy, or biologic therapy, within 21 days or 5 half-lives of the drug (whichever is shorter) prior to the first dose of ABBV-706. Palliative radiation therapy for bone, skin, or subcutaneous metastases with 10 fractions or less is not subject to a washout period. Subject must not have received radiation therapy for CNS metastases within 14 days prior to first dose.
14. Must not have systemically used known strong cytochrome P450 (CYP)3A inhibitors and inducers within 7 days or 5-half-lives (whichever is shorter) prior to the first dose of study drug. Examples of strong CYP3A inhibitors and inducers are listed in Appendix 6, Section 10.6.
15. For China only: Must not have used herbal medications and/or traditional Chinese medicines for anti-SCLC treatment 14 days prior to C1D1.

Subject History:

16. No prior treatment with an antibody drug conjugate that consists of a Top1 inhibitor payload.
17. Part 2 (SCLC monotherapy dose optimization and expansion) only: No prior treatment with a SEZ6-targeted antibody drug conjugate.
18. No recent history (within 6 months) of congestive heart failure (defined as New York Heart Association Class 2 or higher), ischemic cardiovascular event, cardiac arrhythmia requiring pharmacological or surgical intervention (unless pacemaker was installed with no subsequent abnormalities), or pericarditis.
19. No history of ILD or pneumonitis that required treatment with systemic steroids, nor any evidence of active ILD or pneumonitis.
20. No history of idiopathic pulmonary fibrosis or organizing pneumonia (e.g., bronchiolitis obliterans).
21. No history of hypersensitivity to the active ingredients or any excipients of ABBV-706.
22. For Part 3b, no history of hypersensitivity to the active ingredients or any excipients of cisplatin or carboplatin and/or contraindication to cisplatin or carboplatin.
23. No known active viral hepatitis infection (including hepatitis B and C) or human immunodeficiency virus (HIV) with the following exceptions:
    - Subjects with a history of hepatitis B that is considered cured (subject does not need treatment for hepatitis B and has an undetectable hepatitis B viral load) may be enrolled at the discretion of the treating investigator.

- Subjects with a history of hepatitis C that is considered cured with definitive therapy may be enrolled at the discretion of the treating investigator.
- Subjects with HIV and undetectable viral load, so long as ongoing antiretroviral therapy does not pose risk of adverse drug-drug interactions, at the discretion of the treating investigator.

24. No known active severe acute respiratory syndrome coronavirus 2 (SARS-CoV-2) infection. If a subject has signs/symptoms suggestive of SARS-CoV-2 infection, the subject must have a negative molecular (e.g., polymerase chain reaction [PCR]) test or 2 negative antigen test results at least 24 hours apart. Note: SARS-CoV-2 diagnostic tests should be applied following local requirements/recommendations.

    Subjects who do not meet SARS-CoV-2 infection eligibility criteria must be screen failed and may only rescreen after they meet the following SARS-CoV-2 infection viral clearance criteria:

- At least 10 days since first PCR test result have passed in asymptomatic patients or 10 days since recovery, defined as resolution of fever without use of antipyretics and improvement in symptoms.

Contraception

25. For all female subjects of childbearing potential; a negative serum pregnancy test at the Screening Visit and a negative urine/serum pregnancy test at baseline prior to the first dose of study drug.
26. Female subjects of childbearing potential must practice at least 1 protocol-specified method of birth control that is highly effective from Study Day 1 through at least 230 days after the last dose of study drug (or according to local label for carboplatin or cisplatin, whichever is longer, for Part 3b carboplatin or cisplatin combination subjects; Korea only: through at least 14 months after the last dose of cisplatin or carboplatin) (refer to Appendix 4, Section 10.4 for contraceptive and barrier guidance). Female subjects of non-childbearing potential do not need to use birth control.
27. A female subject of childbearing potential who is not pregnant or breastfeeding and is not considering becoming pregnant or donating eggs during the study or for approximately 230 days after the last dose of study drug (or according to local label for carboplatin or cisplatin, whichever is longer, for Part 3b carboplatin or cisplatin combination subjects; Korea only: through at least 14 months after the last dose of cisplatin or carboplatin).
28. For male subjects who are sexually active with a female partner of childbearing potential, must agree to use male condoms, even if the male subject has undergone a successful vasectomy, from Day 1 through at least 140 days after the last dose of study drug (or according to local label for carboplatin or cisplatin, whichever is longer, for Part 3b carboplatin or cisplatin combination subjects; Korea only: through at least 11 months after the last dose of cisplatin or carboplatin).
29. Male subjects should not plan on fathering a child or donating sperm during the study or for approximately 140 days after the last dose of study drug (or according to local label for carboplatin or cisplatin, whichever is longer, for Part 3b carboplatin or cisplatin

combination subjects; Korea only: through at least 11 months after the last dose of cisplatin or carboplatin).

5.2 Additional Exclusion Criteria for Part 3a (Budigalimab Combination):

30. History of inflammatory bowel disease.
31. Body weight less than 35 kg.
32. Subjects who have been previously treated with an anti-PD-1/PD-L1 targeting agent must not have had during the course of their therapy:
    - Any immune-related toxicity of Grade 3 or greater severity.
    - Treatment for any irAE(s) with systemic corticosteroids.
    - Any hypersensitivity to PD-1/PD-L1 targeting agents.
    - History of pneumonitis related to PD-1/PD-L1 therapy.
    - Any treatment-related toxicity resulting in discontinuation of the PD-1/PD-L1 targeting agent.
33. Current or prior use of immunosuppressive medication within 14 days prior to the first dose of study drug. The following are exceptions to this criterion:
    - Intranasal, inhaled, topical steroids or local steroid injections (e.g., intraarticular injection).
    - Systemic corticosteroids at doses not to exceed 10 mg/day of prednisone or equivalent.
    - Steroids as premedication for hypersensitivity reactions (e.g., CT scan premedication).
34. Active autoimmune disease, with exceptions of psoriasis not requiring systemic treatment, vitiligo, and type 1 diabetes mellitus and hypothyroidism that are well controlled with medications.
35. History of primary immunodeficiency or solid organ transplantation.
36. Subjects with a history of Stevens-Johnson syndrome, toxic epidermal necrolysis, or drug reaction with eosinophilia and systemic symptoms.

5.3 Lifestyle Considerations

5.3.1 Sunlight Exposure

The phototoxicity of ABBV-706 is unknown; therefore, subjects should be cautioned not to be exposed to direct sunlight until further evaluation (e.g., high intensity ultraviolet B light sources such as tanning beds, tanning booths and sunlamps, and wear dark glasses, etc.). Subjects are to be advised to report any reaction to sun exposed skin. Subjects should be encouraged to apply sunscreen/sunblock daily.

5.3.2 Contraception

Contraceptive guidelines as specified in Appendix 4, Section 10.4 will be reviewed with male and female subjects who are sexually active. Contraception eligibility criteria are listed in Section 5.1.

5.4 Screen Failure

A screen failure occurs when a subject who has consented to participate in the main clinical study is not subsequently assigned to study intervention

A minimal set of screen failure information is required to ensure transparent reporting of screen failure subjects to meet the Consolidated Standards of Reporting Trials (CONSORT) publishing requirements and to respond to queries from regulatory authorities. Minimal information includes demography, reason for screen failure (e.g., eligibility requirements failed), and documentation of any serious AEs (SAEs) that are related to study procedures.

Subjects who do not meet the criteria for participation in this study (screen failure) may be rescreened. For subjects who rescreen, the screening number assigned at the initial screening visit should be used.

6 Study Intervention(s) and Concomitant Therapy

Study interventions are all pre-specified, investigational and non-investigational medicinal products intended to be administered to the study subjects during the study.

6.1 Study Intervention(s) Administered

Study drug information is presented in Table 6–1.

All laboratory parameters outlined in the inclusion criteria (refer to Inclusion Criterion 5, Section 5.1) must be met prior to study drug dosing on Cycle 1 Day 1.

The dose of ABBV-706 will be based on the subject's weight recorded at Cycle 1 Day 1. The dose of ABBV-706 does not need to be adjusted unless the subject's weight changes by > 10% from baseline. It is not necessary to calculate dosing based on ideal body weight. The exact dose administered should be recorded in the electronic case report form (eCRF). The time of each drug administration (start and end time of infusion) will be recorded to the nearest minute.

[redacted]

Subsequent doses of study drug(s) may be administered if the subject meets the following conditions:

- Absolute neutrophil count (ANC) ≥ 0.75 × 10^9 /L
- Platelets ≥ 75 × 10^9 /L (no transfusions within 5 days prior to dose)
- ALT and AST ≤ Grade 2
- Bilirubin ≤ Grade 1 (or baseline levels at study start)
- Resolution or management of clinically significant (including laboratory) AEs to Grade 0 or 1 (or to the baseline grade if pre-existing) (refer to Protocol Section 6.7).

ABBV-706 will be administered as an IV infusion over 1 hour (±10 minutes), [redacted] beginning on Cycle 1 Day 1. Subjects should be monitored for infusion-related reactions (IRRs) as outlined in Protocol Section 6.7.4 during infusion and for at least 1 hour after the end of infusion. If no issues are observed during or within 1 hour after the first infusion, 1-hour post-infusion monitoring is not required for subsequent cycles.

In the Part 3a combination cohorts with budigalimab, ABBV-706 will be administered first, followed by budigalimab approximately 30 minutes after completion of the ABBV-706 infusion. Budigalimab will be administered with a fixed dose of [redacted] At Cycle 1, the budigalimab infusion will be administered over 60 minutes (window 60 to 70 minutes). If a subject does not experience any IRR during the first dose of budigalimab, the duration of infusion for Cycles 2 and higher of budigalimab may be given over 30 minutes (window 30 to 40 minutes).

In the Part 3b combination cohorts with carboplatin or cisplatin, ABBV-706 will be administered first followed carboplatin or cisplatin approximately 30 minutes after completion of the ABBV-706 infusion. Carboplatin and cisplatin will be administered at AUC 5 and 75 mg/m^2, respectively, according to institutional guidelines.

The Sponsor-provided study drug should not be substituted or alternately sourced unless otherwise directed by the Sponsor. Any non-investigational medicinal products (SoC) must be obtained commercially. Sites are responsible for obtaining emergency medications to manage IRRs, per institutional guidelines.

Table 6‑1 Study Intervention(s) Administered

IMP or NIMP/AxMP	Investigational Medicinal Product	Investigational Medicinal Product	Investigational Medicinal Product	Investigational Medicinal Product
**Investigational product name**	ABBV-706	Budigalimab (ABBV-181)	Cisplatin*	Carboplatin*
**Mode/Route of Administration**	[redacted]	[redacted]	Intravenous	Intravenous
**Dose**	[redacted]	[redacted]	75 mg/m^2	AUC 5
**Frequency of administration**	[redacted]	[redacted]	Q3Wk	Q3Wk
**Strength**	[redacted]	[redacted]	100 mg/vial	150 mg, 450 mg or as locally available

AUC = area under the time concentration curve; N/A = not applicable; Q3Wk = once every 3 weeks
*Manufacturer, strength, and dosage form may vary based on source for Carboplatin and Cisplatin.

6.2 Preparation, Handling, Storage, and Accountability

Detailed study drug preparation, handling, storage, and accountability instructions will be provided to sites in the Pharmacy Manual. Study drugs will be administered at the site with appropriate medical support available to manage infusion reactions if they occur. The study site personnel will document compliance.

ABBV-706 and budigalimab will be packaged in vials with quantities sufficient to accommodate study design. Cisplatin and carboplatin will also be provided by the Sponsor.

Study drug is for investigational use only and is to be used only within the context of this study. The study drugs supplied for this study must be maintained under adequate security and stored under the conditions specified on the label until dispensed for subject use or destroyed on-site as appropriate. Returned study drugs should not be re-dispensed to the subjects.

6.3 Assignment to Study Intervention

This is an open-label, dose escalation and dose expansion study. Parts 1, 3, and 4 are non-randomized. In Part 2a (monotherapy dose optimization), subjects will be randomized evenly between at least two dose cohorts. There are no stratification factors in this study. All subjects will be assigned a unique identification number by the IRT at the Screening Visit or by the Sponsor directly until IRT is available. Eligible subjects will receive study drug(s) as described in the Protocol Section 6.1.

For subjects who do not meet the study selection criteria, the site personnel must contact the IRT system and identify the subject as a screen failure.

Subjects who are enrolled will retain their subject number assigned at the Screening Visit throughout the study. Upon receipt of study drug, the site will acknowledge receipt in the IRT system. Contact information and user guidelines for IRT use will be provided to each site.

Study intervention will be administered/dispensed at the study visits as summarized in the SoA (Table 1–1 for Part 1 and Table 1–2 for Parts 2, 3, and 4).

6.4 Blinding/Masking

Not applicable.

6.5 Study Intervention Compliance

When subjects are dosed at the site, they will receive study drug directly from the investigator or designee, under medical supervision. The date and time of each dose administered in the clinic will be recorded in the source documents.

6.6 Study Intervention Product Complaints

Complaints

A complaint is any written, electronic, or oral communication that alleges deficiencies related to the physical characteristics, identity, quality, purity, potency, durability, reliability, safety, effectiveness, or performance of a product/device. Complaints associated with any component of this investigational product must be reported to AbbVie.

Product Complaint

A product complaint is any complaint related to the biologic or treatment component of the product or to the medical device component(s).

A product complaint may include, but is not limited to, deficiencies related to the product's physical characteristics, identity and quality, damaged/broken product or packaging, product appearance whose color/markings do not match the labeling, labeling discrepancies/inadequacies in the labeling/instructions (e.g., printing illegible), missing components/product, device damage or not working properly, or packaging issues.

Product complaints concerning the investigational product and/or device must be reported within 24 hours of the study site's knowledge of the event via electronic data capture (EDC). The date the product complaint details are entered and saved in EDC represents the date reported to the Sponsor. A back-up paper form will be provided for reporting complaints related to unassigned product. In the event of an EDC system issue, a back-up paper form should be used. If a back-up paper form is used, the date the form is emailed to RD_PQC_QA@abbvie.com represents the date reported to the Sponsor.

All follow-up information is to be reported to the sponsor (or an authorized representative) and documented in source as required by the Sponsor. Product complaints associated with AEs will be reported in the study summary. All other complaints will be monitored on an ongoing basis. Product complaints occurring during the study will be followed up to a satisfactory conclusion.

6.7 Management of Drug-Related Toxicities

The dose of study drug(s) can be delayed for up to 21 days after the scheduled dosing date. For delays of > 21 days, the investigator should discuss with the Contract Research Organization's (CRO) medical monitor or the Sponsor's Therapeutic Area Medical Director if study drug should be continued, reduced, or discontinued. It is strongly recommended that the investigator discuss with the CRO medical monitor or Sponsor's Therapeutic Area Medical Director prior to dose reduction.

ABBV-706 Toxicities:

For Grade 4 non-hematologic toxicities that are not clearly related to disease progression or any underlying illness or alternative etiology, study drug should be permanently discontinued. For clinically significant Grade 3 non-hematologic toxicities not specifically described in the sections below, dosing will be held until toxicity resolves to ≤ Grade 1 or to baseline. Re-initiation of study drug may be considered at a reduced dose according to the following guidelines:

- For any subject who experiences Grade 3 non-hematologic toxicity that is not clearly related to disease progression or any underlying illness or alternative etiology, the dose will be held until the toxicity resolves to Grade 1 or lower, or to baseline if the toxicity is > Grade 1 at the time of study entry (except for toxicities that require study drug discontinuation). Upon toxicity resolution the subject can re-start study drug at a dose that is at least 25% lower than the original dose. A maximum of three ABBV-706 dose reductions of at least 25% are allowed after which the subject will be discontinued from study drug.
- Study drug interruptions for events that are clearly not related to the study drug treatment, (e.g., underlying cancer, planned surgical procedures or acute illnesses of other etiology), should not necessitate a dose reduction. The timing of dose resumption should be at the discretion of the investigator.
- If the subject must undergo emergency surgery, the study drug should be interrupted at the time of the surgery. After emergency surgery, allow reintroduction of study drug at the same dose or a reduced dose at the discretion of the physician once the physician has examined the surgical site and determined that it is appropriate to re-start the drug.

- Increase of ABBV-706 dose up to no higher than the original dose level may be considered after a dose reduction in certain circumstances upon resolution of offending toxicities to Grade 1 or less or baseline for a minimum of 2 cycles post-reduction. All dose re-escalation decisions must be made after discussion and agreement with the CRO medical monitor or the Sponsor's Therapeutic Area Medical Director.

For Part 3a combination with budigalimab, refer to Protocol Section 6.7.7 and Appendix 8, Section 10.8 for dose modification and treatment guidelines for immune-related toxicities. No dose reductions are allowed for budigalimab. Toxicity management for carboplatin and cisplatin should follow institutional guidelines. Dose reductions for carboplatin and cisplatin based on renal function should follow local/institutional guidelines. In general, for combination cohorts, if toxicities require dose hold, all study drugs should be held until adequate resolution to resume dosing. Adjustments to these guidelines may occur only in consultation with the CRO medical monitor or the Sponsor's Therapeutic Area Medical Director.

Dose modifications for specific drug-related toxicities (ABBV-706) and treatment guidelines for such toxicities are provided in the sections that follow.

6.7.1 Hematologic Toxicity

ABBV-706 may cause anemia, thrombocytopenia (see Table 6-2 for management guidelines), or neutropenia (see Table 6-3 for management guidelines). Transfusions and growth factors (e.g., G-CSF) should be provided per institutional standard, though prophylactic use of growth factors and RBC or platelet transfusions are not allowed during the DLT period. For neutropenia, antibiotic and antifungal treatment and prophylaxis may be provided without delay per institutional guidelines except for the prohibited medication (Protocol Section 6.11.2). Please note that posaconazole and itraconazole are strong CYP3A inhibitors and should be used with caution. In contrast, liposomal amphotericin and micafungin are allowed. If hematologic toxicity can be managed with supportive care, then a dose reduction will not be required.

Table 6-2 ABBV-706 Toxicity Management Guidelines for Neutropenia/Febrile Neutropenia

Toxicity	Recommended Action
ANC ≥ 1.5×10^9/L	[redacted]
ANC < 1.5 to 0.75×10^9/L	
ANC < 0.75×10^9/L	
Febrile neutropenia (ANC < 1.0×10^9/L with a single temperature of > 38.3°C (101°F) or a sustained temperature of ≥ 38°C (100.4°F) for more than 1 hour)<br>OR<br>Persistent ANC < 0.75×10^9/L resulting in delay of start of subsequent cycle by >7 days	

ANC = absolute neutrophil count; G-CSF = granulocyte-colony stimulating factor

Table 6-3 Toxicity Management Guidelines for Thrombocytopenia

Toxicity	Recommended Action
Grade 1 thrombocytopenia (platelets ≥ 75 × 10^9/L)	[redacted]
Grade 2 thrombocytopenia (platelets < 75 to 50 × 10^9/L)	
Grade 3 thrombocytopenia (platelets < 50 to 25 × 10^9/L)<br>OR<br>Grade 4 thrombocytopenia (platelets < 25 × 10^9/L)	

ANC = absolute neutrophil count; G-CSF = granulocyte-colony stimulating factor

6.7.2 ILD/Pneumonitis Toxicity

If a subject develops symptoms of treatment-emergent ILD/pneumonitis, it is recommended that the latest published guidelines are followed to work-up, diagnose, and treat ILD/pneumonitis.[11,12] An adaptation of those guidelines are presented in Table 6–4. If a subject develops a Grade 2 or greater treatment-emergent ILD/pneumonitis, ABBV-706 should be permanently discontinued.

For Part 3a combination with budigalimab, ILD/pneumonitis can also be associated with anti-PD-1 treatment. If a subject in Part 3a develops symptoms of treatment-emergent ILD/pneumonitis, refer to Appendix 8, Section 10.8.6 for budigalimab management guidelines in addition to Table 6–4 below.

Table 6–4 Management of ILD/Pneumonitis

Grading	Recommended Action
**Grade 1**: Asymptomatic, confined to one lobe of the lung or < 25% of lung parenchyma, clinical or diagnostic observations only	[redacted]
**Grade 2**: Symptomatic, involves more than one lobe of the lung or 25% to 50% of lung parenchyma, medical intervention indicated, limiting instrumental ADL	
**Grade 3**: Severe symptoms, hospitalization required, involves all lung lobes or > 50% of lung parenchyma, limiting self-care ADL, oxygen indicated	
**Grade 4**: Life-threatening respiratory compromise, urgent intervention indicated (intubation)	

ADL = activities of daily living; BAL = bronchoalveolar lavage; CT = computed tomography; DLCO = diffusing capacity of lung for carbon monoxide; IV = intravenous

6.7.3 Hepatotoxicity

Table 6–5 ABBV-706 Dose Modification Guidelines for Hepatotoxicity

	Grade 2	Grade 3	Grade 4
AST and ALT	[redacted]		
Hyperbilirubinemia			

ALT = alanine aminotransferase; AST = aspartate aminotransferase; DILI = drug induced liver injury
Note: In cases of confirmed DILI, ABBV-706 should be permanently discontinued.

6.7.4 Infusion-Related Reactions (IRRs)

IRRs may occur with ABBV-706 and budigalimab (ABBV-181), with signs and symptoms including fever, chills, nausea, vomiting, diarrhea, rash, hypotension, tachycardia, hypoxia, and respiratory failure. Reactions usually occur during or shortly after completion of the infusion. Subjects should be monitored during and for at least 1 hour after the end of infusion for the potential onset of IRRs. If no issues are observed during or within 1 hour after the first infusion, 1-hour post-infusion monitoring is not required for subsequent cycles. Longer observation periods and more frequent vital sign checks may be required for subjects who experience IRRs. Signs and symptoms related to IRRs believed to be related to ABBV-706 will be recorded using the IRR eCRF.

IRRs should be managed according to institutional SoC (see Table 6–6 for additional guidance for managing of IRRs). The specific strategies to be employed for a given subject or cohort should be discussed with the CRO's medical monitor or the Sponsor's Therapeutic Area Medical

Director prior to implementation – which will be informed by PK data from prior subjects, clinical findings from previous IRRs, and effectiveness of prophylaxis for previous subjects/cohorts – and will be specified prior to initiation of dosing. Details on doses and timing for prophylactic steps will be captured in appropriate eCRFs for each dose of ABBV-706.

Table 6–6 Management of Infusion-Related Reactions

Grading	Recommended Action
**Grade 1**: Mild-transient reaction	[redacted]
**Grade 2**: Infusion interruption indicated but responds promptly to symptomatic treatment	
**Grade 3**: Prolonged (not rapidly responsive to symptomatic medication and/or brief interruption of infusion); recurrence of symptoms following initial improvement; hospitalization indicated for clinical sequelae	
**Grade 4**: Life-threatening consequences; urgent intervention indicated	

Reflexive Testing Following IRRs

If any IRR or suspected allergic-type reaction is observed, a blood draw is recommended within 2 hours after the first sign of a reaction for the following laboratory tests:

- C3a, C5 complement functional assay, IgE level, and serum tryptase level.

6.7.5 Peripheral Neuropathy

Subjects should be monitored for peripheral neuropathy, which can be managed by a combination of dose delays and dose reductions as outlined in Table 6–7.

Table 6–7 Dose Modification Guidelines for Peripheral Neuropathy

Grade 2	Grade 3	Grade 4
[redacted]		

6.7.6 Dermatologic Toxicity

Subjects will be informed of preventive measures for dermatologic toxicities (use of sunscreens and avoiding sun exposure) during informed consent.

Subjects developing dermatologic toxicities while receiving study drug should be monitored for the development of inflammatory or infectious sequelae, and appropriate treatment of these symptoms initiated. Treatment with topical corticosteroids, oral antibiotics (e.g., doxycycline), skin moisturizers, and sunscreen should be considered. These treatments may also be employed prophylactically, per the investigator's discretion, especially if dermatologic toxicity is noted earlier during dose escalation.

In subjects with mild (Grade 1) dermatologic toxicity, treatment should continue without dose modification. If a subject experiences moderate or severe (Grades 2, 3, or 4) dermatologic toxicity, study drug treatment adjustments should be made by the investigator based on guidance outlined in Table 6–8.

For Part 3a combination with budigalimab, dermatologic toxicities can also be associated with anti-PD-1 treatment. If a subject in Part 3a develops dermatologic toxicities, refer to Appendix 8 Section 10.8.2 for budigalimab management guidelines in addition to Table 6–8 below.

Table 6–8 Dose Modification Guidelines for Dermatologic Toxicity

Grade 2	Grade 3	Grade 4
[redacted]		

6.7.7 Immune-Related Adverse Events for Part 3a Combination with Budigalimab

Approved anti-PD-1 therapies are associated with irAEs.[12,14,15] Based on the available clinical data to date, the safety profile of budigalimab is consistent with those identified with other agents targeting the PD-1 receptor, and no unique toxicities have been observed. Fatigue, pruritus, diarrhea, hypothyroidism, night sweats, infusion-related reaction, hyperglycemia, acute kidney injury, immune-mediated hepatitis, diabetic ketoacidosis, Type 1 diabetes mellitus, and ILD (pneumonitis) have been identified as adverse drug reactions for budigalimab based on the mechanism action of PD-1 inhibition and medical judgment. Data regarding the frequency treatment-emergent irAEs following treatment with budigalimab is available in the budigalimab Investigator's Brochure.[8]

In Part 3a of the current study, subjects will be monitored continuously, as an irAE with budigalimab may occur at any time during or after discontinuation of budigalimab therapy. For suspected irAEs, evaluation will be performed to confirm immune etiology or exclude other causes. Appendix 8, Section 10.8 includes guidance regarding management of irAEs including study interruption, initiation of corticosteroids and noncorticosteroid immunosuppressive therapy, and study drug discontinuation. Prophylactic antibiotics may be used to prevent opportunistic infections in subjects receiving immunosuppressive therapy.

6.8 Continued Access to Study Intervention After the End of the Study

There is no intervention planned following the end of the study.

6.9 Treatment of Overdose

In case of accidental overdose of study drug(s), periodic monitoring and assessment of the subject's status should be initiated. Monitor all body systems as required, depending on the

major toxicities of the study drug(s). Compare baseline laboratory values and review systems at regular intervals. Additional supportive care should be provided as clinically indicated.

6.10 Prior and Concomitant Medications Review

The investigator or qualified designee will review any medication or vaccine use with the subject at the time of consent and record as prior medications taken by the subject.

If a subject reports taking any over-the-counter medications, prescription medications, vitamins and/or herbal product and dietary supplements, or if administration of any medication becomes necessary from signature of consent through 80 days (for Parts 1, 2, 3b, and 4) or 90 days (for Part 3a) after last dose of study drug, the name of the medication, dosage information including dose, route, and frequency, date(s) of administration including start and end dates, and reason for use must be recorded on the appropriate eCRF.

6.11 Concomitant Medications/Therapy

6.11.1 Allowed

- Subjects should receive full supportive care during study participation (except for restricted medications listed in the Protocol Section 6.11.2), including but not limited to transfusion of blood products, fluid and electrolyte replacement, cytokines, antiemetics, antifungals, and antibiotics when appropriate as treatment or as prophylaxis. Subjects who use oral contraceptives, hormone replacement therapy, or other maintenance therapy should continue their use.
- Strong CYP3A inhibitors and inducers should be used with caution throughout the study. Alternative medications with less CYP3A inhibition should always be considered. Examples of strong CYP3A inhibitors and inducers are listed in Appendix 6, Section 10.6.

The CRO's medical monitor or the Sponsor's Therapeutic Area Medical Director should be contacted if there are any questions regarding concomitant or prior therapy(ies).

6.11.2 Prohibited

In addition to the medications listed in the eligibility criteria, the following are not allowed during the study:

- Any anticancer therapy including chemotherapy, immunotherapy, or biologic therapy.
- Any other investigational drugs.

- Radiation therapy for disease control is prohibited except for CNS radiation for treatment of brain metastases or local ablative therapy for oligo-progressive disease (progression in 3 or fewer sites of disease) for subjects with non-CNS primary cancers (see Protocol Section 6.11.3).
- Live vaccines are not allowed during the study. For non-live vaccines (including mRNA vaccines), when possible, there should be at least 3 days between administration of the vaccine and study drug administration and at least 7 days is preferred prior to first dose of study drug.
- Medications known to impact QT interval are prohibited during Cycle 1 and Cycle 3 visits on the day before and days of triplicate ECG collections (see Appendix 7, Section 10.7 for a list of QT-prolonging medications that are either prohibited or should be avoided). In the case of clinical necessity, please discuss acceptable medication alternatives and dosing regimens with the CRO's medical monitor or the Sponsor's Therapeutic Area Medical Director.
- **For subjects enrolled under Part 2c:** Herbal supplements and/or traditional Chinese medicines intending to treat SCLC must not be used within 14 days prior to the start and/or during study treatment.

6.11.3 Concomitant Radiation Therapy

Radiation therapy for disease control is prohibited except for CNS radiation for treatment of brain metastases or local ablative therapy for oligo-progressive disease for subjects with non-CNS primary cancers. A 14-day washout period is required after completion of radiation therapy to the CNS; no washout period is required for non-CNS lesions. Local control therapy for symptom palliation may be used after discussion with the CRO's medical monitor or the Sponsor's Therapeutic Area Medical Director after the DLT assessment period.

Isolated evidence of progression of CNS metastases in subjects with non-CNS primary cancers will not require study drug discontinuation; however, these subjects should still be considered progressive disease per RECIST v1.1 for the response assessment. These subjects may, at the discretion of the investigator and in consultation with the CRO's medical monitor or the Sponsor's Therapeutic Area Medical Director, suspend study drug and undergo appropriate palliative CNS therapy, and then recommence study drug at least 2 weeks after completion of radiation therapy until non-CNS progression develops.

Additionally, at the discretion of the investigator and in consultation with the CRO's medical monitor or the Sponsor's Therapeutic Area Medical Director, subjects with non-CNS primary cancers who have responded in target lesions (as defined by RECIST PR or CR) to study drug(s)

monotherapy or combination therapy, and progress with 3 or fewer sites of disease (including extracranial lesions), will be given the option to continue on study drug(s) after local ablative therapy of progressing lesions until the next documented progression, after which they must discontinue study drug. No washout period is required for non-CNS lesions. It is highly recommended that subjects have a biopsy of one or more progressing lesions prior to or concurrent with local ablative therapy if deemed safe by the investigator.

6.12 COVID-19 Pandemic-related Vaccination Guidance

Given the ongoing COVID-19 pandemic, selected non-live vaccines (e.g., mRNA, non-replicating viral vector, protein subunit, etc.) to prevent SARS-CoV-2 infection may be administered during Screening, treatment period, or follow-up as long as components of that vaccine are not contraindicated.

The decision to receive a locally available vaccine should be based on local guidance and an individual discussion between the treating physician and the subject.

The impact of different anticancer treatments (e.g., cytotoxic therapy, immunotherapy etc.) on vaccine response is not currently known for any of the available SARS-CoV-2 vaccines. Based on prior experience with non-live vaccines administered to immunocompromised patients, the main concern is impact on vaccine effectiveness.

As the potential impact of ABBV-706 on SARS-CoV-2 vaccination is unknown, administration of locally approved non-live vaccines should be performed as follows:

For new subjects being screened:

- The first dose of ABBV-706, when possible, is preferred to be given at least 7 days after SARS-CoV-2 vaccine administration.

For subjects already enrolled:

- For subjects currently on treatment with ABBV-706, the vaccine should be administered after the first month of treatment with ABBV-706 or at the end of the DLT evaluation period, whichever is longer.
- When possible, there should be at least 3 days between administration of the vaccine and study drug administration.

Note: The above guidance applies to all SARS-CoV-2 vaccine doses given as part of the complete vaccination course.

These recommendations may be subject to change based on the evolving knowledge around the use of SARS-CoV-2 vaccines in patients with solid malignancies and as more data are collected in real-world scenarios and clinical trials.

- Any SARS-CoV-2 vaccine information must be documented on the COVID-19 Vaccine eCRF. Refer to the Appendix 3, Section 10.3 for instructions on reporting any AEs associated with the COVID-19 vaccine.

7 Discontinuation of Study Intervention and Withdrawal From the Study

7.1 Subject Discontinuation From Study Intervention

Subjects will continue study treatments until radiographic disease progression (as defined by RECIST version 1.1 or RANO criteria), clinical progression, intolerable toxicity, or until other study discontinuation criteria are met.

Treatment with **study drug(s)** should be discontinued for any of the following reasons:

- Progressive disease per RECIST v1.1 or RANO (for exceptions, refer to Section 6.11.3).
- The investigator believes it is in the best interest of the subject.
- The subject requests to discontinue study drug treatment.
- Clinically significant abnormal laboratory results or AEs, which meet discontinuation criteria, as determined by the investigator or the Sponsor.
- Eligibility criteria violation was noted after the subject started study drug and continuation of the study drug would place the subject at risk.
- Introduction of prohibited medications or dosages and continuation of the study drug would place the subject at risk.
- The subject becomes pregnant while on study drug.
- The investigator determines the subject is significantly noncompliant with study procedures.

If a subject permanently discontinues from the study drug(s), all attempts must be made to determine the primary reason for discontinuation of study drug. The information will be recorded on the appropriate eCRF page. Following discontinuation of study drug, the subject will be treated in accordance with the investigator's best clinical judgment, regardless of the subject's decision to continue participation in the study.

All subjects should be encouraged to remain in the study to collect data at the time of discontinuation of study drug for any further evaluations that need to be completed and for Safety Follow-Up (see the SoA [Table 1–1] for Part 1 and [Table 1–2] for Parts 2, 3, and 4).

When a subject's withdrawal request is limited to discontinuation of the study intervention, study activities involving other types of participation for which the subject previously gave consent may continue.

7.1.1 COVID-19 Pandemic-Related Acceptable Protocol Modification

During the COVID-19 pandemic, it has been necessary to employ mitigation strategies to enable the investigator to ensure subject safety and continuity of care.

The investigator should contact the CRO's medical monitor or the Sponsor's Therapeutic Area Medical Director before discontinuing a subject from the study for a reason other than described in the protocol to ensure all acceptable mitigation steps have been explored.

7.1.2 Interruption/Discontinuation of Study Drug Due to COVID-19 Infection

The investigator should weigh the risks and benefits of continued treatment with study drug(s) in subjects with confirmed COVID-19 infection. The decision to re-start study treatment following an interruption for COVID-19 infection should be made based on investigator's assessment of a subject's clinical status, results of any COVID-19 specific testing and appropriate local treatment guidelines. In addition, the investigator should discuss plan for re-initiation or discontinuation of study drug(s) in subjects who develop COVID-19 infection while on study treatment with the CRO's medical monitor or the Sponsor's Therapeutic Area Medical Director prior to re-starting or discontinuing study treatment.

7.2 Subject Withdrawal From the Study

A subject may voluntarily withdraw or be withdrawn from the study at any time for reasons including the following:

- The subject requests withdrawal from the study
- Death of a subject
- Subject is lost to follow-up

The Sponsor may terminate this study prematurely, either in its entirety or at any site. If the Sponsor terminates the study for safety reasons, the Sponsor will promptly notify the investigators. Discontinuation of specific sites or of the study is handled as part of Appendix 1, Section 10.1.7.

An investigator may also stop the study at his/her site if he/she has any concerns.

7.3 Lost to Follow-Up

For subjects to be considered lost to follow-up, reasonable attempts must be made to obtain information on the subject's final status. At a minimum, 2 telephone calls must be made and 1 certified letter must be sent and documented in the subject's source documentation.

To minimize missing data for efficacy and safety assessments, subjects who prematurely discontinue study drug treatment due to clinically significant abnormal laboratory results or AEs as determined by the investigator or CRO's medical monitor or the Sponsor's Therapeutic Area Medical Director, should continue to be followed for all regularly scheduled visits, unless subjects have decided to discontinue the study participation entirely (withdrawal of informed consent). Subjects should be advised on the continued scientific importance of their data even if they discontinue treatment with study drug early.

If a subject prematurely discontinues study participation (withdrawal of informed consent), the procedures outlined for the End of Treatment visit (see the SoA [Table 1–1] for Part 1 and [Table 1–2] for Parts 2, 3, and 4) should be completed as soon as possible, preferably within 2 weeks.

If a subject withdraws from study follow-up or withdraws permission for the collection of their personal data, the study staff may still use available public records to obtain information about survival status only, as appropriate per local regulations.

7.4 Withdrawal From Biomarker Research

In the event a subject withdraws consent from the clinical study, biomarker research will continue unless the subject explicitly requests analysis to be stopped. When the Sponsor is informed the subject has withdrawn and no longer wishes biomarker samples research to continue, samples will not be analyzed, and no new biomarker analysis data will be collected for the withdrawn subject or added to the existing data or database(s). Data generated from biomarker research before the subject withdrew consent will remain part of the study results. A subject may withdraw consent for optional biomarker research at any time and remain in the clinical study.

8 Study Assessments and Procedures

Study visits may be impacted due to the COVID-19 pandemic. Every effort should be made to ensure the safety of subjects and site staff, while maintaining the integrity of the study. If visits cannot be conducted on-site due to travel restrictions or other pandemic-related reasons, sites should contact the study team to discuss operational changes.

For protocol Days 8 and 15 of Cycles 1-5, visits are required per the SoA (Section 1.3). From Cycle 6 and onward, visits on Days 8 and 15, and all assessments at those visits, may be considered optional based on Investigator's medical discretion. If a visit is performed at the site on Days 8 and 15 of Cycle 6 and onward, all assessments should be performed per the SoA (Section 1.3).

For unscheduled visits, subjects may have laboratory testing performed at a local laboratory, hospital, or other facility as needed. Every effort should be made to ensure the safety of subjects and site staff, while maintaining the integrity of the study.

8.1 Administrative and Baseline Procedures

8.1.1 Study Subject Informed Consent

To protect subjects' confidentiality, all subjects and their associated samples will be assigned numerical study identifiers or "codes." No identifiable information will be provided to the Sponsor. The investigator or his/her representative will explain the nature of the study, including the use and assessment of any investigational product(s), the benefits and risks anticipated from participation in the study and answer all questions regarding this study. Prior to any study-related screening procedures being performed on the subject or any medications being discontinued by the subject in order to participate in this study, the informed consent statement will be reviewed, signed, and dated by the subject or their legally authorized representative and the investigator (or designee), as well as any other signatories according to local requirements. A copy of the signed informed consent form (ICF) will be given to the subject and the original will be placed in the subject's medical record.

8.1.2 Informed Consent for Biomarker Research

Optional biomarker research samples will only be collected if the subject has voluntarily signed and dated a written consent form describing the exploratory research. The written consent may be part of the main consent form or a separate form that has been IRB/IEC approved. If the subject does not consent to providing optional samples, the subject will still be allowed to participate in the study.

If a protocol amendment is required, the ICF may need to be revised to reflect the changes to the protocol. If the ICF is revised, it must be reviewed and approved by the appropriate IEC/IRB and signed by all participants subsequently enrolled in the study as well as those currently enrolled in the study.

8.1.3 Medical and Cancer History

A complete medical and cancer history will be taken at Screening and documented including below:

- Demographics
- Past and present tobacco use, alcohol, and drug use

- Detailed oncology history with diagnosis (tumor type), date of diagnosis, method of diagnosis, any surgical procedures and treatments administered (including dates and type of modality and treatment results such as tumor response and severe AEs), dates and results of most recent previous tumor assessments, and present signs and symptoms.

8.1.4 Audiology Assessment - Part 3b Only

Hearing damage is one of the main toxic effects of platinum compounds.[15] Each subject in Part 3b will have an age-appropriate Screening audiology assessment done. A trained audiology specialist should perform the test, and an audiologist should interpret the results. Any abnormalities will be recorded at Screening. Subjects with baseline hearing deficits at study entry should be informed of potential for worsening hearing loss with platinum therapy by the treating physician. Further audiology assessments on study should be performed as clinically indicated.

8.1.5 Echocardiogram or MUGA

To be eligible for study participation, subjects must have an ejection fraction of ≥ 50% as assessed by echocardiogram or MUGA at Screening.

8.1.6 Eastern Cooperative Oncology Group Performance Status

For all subjects, ECOG performance status will be performed at Screening and verified at Cycle 1 Day 1 prior to the first dose of study drug. Only subjects with ECOG performance status of 0 or 1 are eligible for study participation.

It is recommended, when possible, that a subject's performance status be assessed by the same person throughout the study. ECOG performance status will be assessed as follows:

Grade	Description
0	Fully active, able to carry on all pre-disease performance without restriction.
1	Restricted in physically strenuous activity but ambulatory and able to carry out work of a light or sedentary nature, e.g., light housework, office work.
2	Ambulatory and capable of all self-care but unable to carry out any work activities. Up and about more than 50% of waking hours.
3	Capable of only limited self-care, confined to bed or chair more than 50% of waking hours.
4	Completely disabled. Cannot carry on any self-care. Totally confined to bed or chair.
5	Dead

8.1.7 Archival or Fresh Tumor Biopsy

Confirmation of an archival or fresh tumor biopsy specimen for submission is required during screening for enrollment and must be sent to a designated lab for retrospective SEZ6 protein expression analysis (refer to Laboratory Manual for details). This sample will be used to inform future potential SEZ6 cut-off for subject selection.

Subjects that have previously provided tissue sampling for SEZ6 testing in other Sponsor studies and meet the sampling requirements outlined by the Sponsor will not be required to provide further tissue samples for this study.

If archival tissue is not available and obtaining a fresh biopsy poses significant risk to the safety of the subject, the treating physician can discuss the case with the medical monitor or Sponsor to determine eligibility.

8.2 Dose-Limiting Toxicity Assessment

[redacted]

Dose-limiting toxicities are defined as any of the following during the DLT evaluation period, excluding toxicities clearly related to underlying disease or intercurrent illness or alternative etiology. For combination therapies in Part 3, toxicities related to combination drugs (i.e., budigalimab, cisplatin, or carboplatin) meeting DLT criteria will be counted as DLTs. The AEs are graded according to the National Cancer Institute Common Terminology Criteria for Adverse Events (NCI CTCAE) v5.0.

Any Grade 2 or higher ILD/pneumonitis and Grade 3 or higher non-hematologic toxicity will be documented as a DLT except for the following:

- Grade 3 nausea or vomiting lasting ≤ 72 hours and successfully managed with medical management.
- Grade 3 diarrhea lasting ≤ 72 hours and successfully managed with anti-diarrheals.
- Grade 3 constipation lasting ≤ 72 hours.

- Grade 3 fatigue lasting ≤ 7 days.
- Grade 3 acute infusion reaction that resolves to Grade ≤ 1 within 24 hours after the end of dosing.
- Grade 3 laboratory abnormalities that resolve, with or without intervention, to Grade ≤ 1 or baseline within 72 hours.
- Grade 3 electrolyte imbalance lasting ≤ 72 hours, unless associated with clinical symptomatology despite supplementation.

The following hematologic toxicities will be considered DLTs:

- Any Grade 4 hematologic toxicity EXCEPT for:
  - Grade 4 afebrile neutropenia lasting ≤ 7 days.
  - Grade 4 leukopenia lasting ≤ 7 days.
  - Grade 4 lymphopenia lasting ≤ 7 days.
- Grade ≥ 3 thrombocytopenia with bleeding or thrombocytopenia requiring transfusion.
- Febrile neutropenia.

Miscellaneous DLTs:

- Any increase in AST or ALT > 3 × ULN with concurrent increase in total bilirubin > 2 × ULN without alternative explanations (e.g., viral hepatitis, disease progression in the liver, etc.).
- Any increase in AST or ALT > 8 ULN without alternative explanations (e.g., viral hepatitis, disease progression in the liver etc.).
- Any increase in total bilirubin > 3 × ULN without alternative explanation.
- Grade ≥ 3 increases in creatinine.
- Any toxicity not related to the underlying disease or extraneous causes resulting in delay of Cycle 2 > 7 days (the delay due to toxicity will not include any operational or administrative delays).
- Any death, excluding accidental death and those clearly related to disease progression or intercurrent illness.

Any subject who does not complete the full 21-day DLT observation period for any reason other than DLT will be considered non-DLT evaluable for dose escalation and/or MTD assessment and will be replaced at the same dose level, if needed, to achieve a minimum cohort size. Additional subjects may be enrolled at a given dose level in the absence of a DLT to explore factors influencing AEs or to accumulate additional safety data.

8.3 Tumor Response Assessments

Planned timepoints for all tumor assessments are provided in the SoA (Table 1–1 for Part 1 and Table 1–2 for Parts 2, 3, and 4).

Baseline tumor assessments must be conducted within 28 days prior to the first dose of study treatment on Cycle 1 Day 1. Post-baseline tumor assessment will be conducted every 6 weeks (allowable window of ± 7 days) from Cycle 1 Day 1 until Week 24, and every 12 weeks (± 7 days) thereafter until radiographic progression, start of a new anticancer therapy, death, or subject withdrawal of consent.

Scheduled tumor assessments will not be affected by delays in therapy. Tumor response assessments should be conducted prior to the start of the next scheduled cycle of study treatment. The measure of response and disease progression will be determined by the investigator according to the RECIST version 1.1[17] (Appendix 9, Section 10.9) for non-CNS primary tumors or RANO criteria[18] with modifications from the 2017 RANO[19] (Appendix 10, Section 10.10) for CNS primary tumors. The same imaging technique should be used throughout the study if possible. Scans will be collected and may undergo independent central review.

For subjects who do not have documented progression by imaging, radiographic tumor assessments will continue as per the scheduled scan frequency until disease progression documented by imaging, start of a new anticancer therapy, death or withdrawal of consent. Radiographic progression per RECIST version 1.1 should be documented when possible.

For non-CNS primary tumors, diagnostic quality CT scans with intravenous contrast are recommended. MRI with contrast may be used if performed consistently throughout the study for each individual subject. Scans of the chest, abdomen, and pelvis must be obtained; scans of the neck must also be obtained if there is documented or suspected involvement in this region. Positron emission tomography (PET) may be used in an ancillary manner; however, no treatment decisions may be made based on PET alone. Baseline MRI of the brain is only required if suspected or known/documented CNS metastases are present. CT of the brain with intravenous contrast may be substituted for subjects for whom MRI is contraindicated. If no CNS metastases are present, on treatment MRI or CT of the brain is required only when clinically indicated. If CNS metastases are detected at baseline, they should be followed in accordance with scheduled tumor assessments.

For primary high-grade CNS tumors (GBM, IDH-wildtype Grade 4; oligodendroglioma, IDH-mutant, and 1p/19q-codeleted Grade 3; astrocytoma, IDH-mutant Grade 3 or Grade 4), MRI brain with and without intravenous contrast is the preferred modality unless contraindicated for a subject, in which case CT of the brain with IV contrast may be used.

8.4 Safety Assessments

Planned timepoints for all safety assessments are provided in the SoA (Table 1–1 for Part 1 and Table 1–2 for Parts 2, 3, and 4).

8.4.1 Physical Examination

A physical examination including pulmonary exam and neurological assessment (including motor, sensory, reflexes, coordination, gait, bowel/bladder/sexual function, and mental status) will be performed at visits as specified in the SoA (Table 1–1 for Part 1 and Table 1–2 for Parts 2, 3, and 4). At any time, a symptom-directed physical examination can be performed as deemed necessary by the investigator. The physical examination performed on Study Day 1 will serve as the baseline physical examination for the entire study. Physical examination abnormalities noted at the Baseline Visit prior to the first dose of study drug should be recorded in the subject's medical history. Any significant physical examination findings after the first dose will be recorded as AEs. All findings, whether related to an AE or part of each subject's medical history, will be captured on the appropriate eCRF page.

8.4.2 Height and Weight

Height will be measured at Screening only. Weight will be measured at Screening, Day 1 of every cycle (predose), and at EOT, preferably without outer garments such as hats, coats, or shoes. Measurements while wearing light indoor clothing or undergarments only are acceptable, and every effort should be made to measure subjects in the same or similar clothing at each assessment.

8.4.3 Vital Signs

Vital sign determinations of systolic and diastolic blood pressure, pulse rate, respiratory rate, and body temperature will be obtained at visits as specified in the SoA (Table 1–1 for Part 1 and Table 1–2 for Parts 2, 3, and 4). Blood pressure and pulse rate should be measured after the subject has been sitting for at least 3 minutes.

Vital signs should be assessed prior to start, 30 minutes (±10 minutes) after the start of infusion, and at the end of the infusion before leaving the site. Any new signs or symptoms which develop after the infusion should be monitored for satisfactory resolution before the subject leaves the investigative site.

Longer observation periods and more frequent vital sign checks may be required for subjects who experience IRRs.

Changes in vital signs are considered to be AEs only if they result in discontinuation from the study, necessitate therapeutic medical intervention, and/or if the investigator considers them to be AEs.

8.4.4 Standard 12-lead Electrocardiograms

A 12-lead (with a 10-second rhythm strip) tracing will be used for all ECGs. The machine used must have the capacity to automatically calculate heart rate and PR interval, QT interval, QTc interval, RR interval, and QRS complex.

ECGs will be acquired after the subject has been in the supine or semi-recumbent position for at least 5 minutes. Subjects will be instructed to remain completely stationary (no talking, laughing, deep breathing, sleeping, or swallowing) for approximately 10 seconds during the ECG recording. While ECGs are being acquired, subjects and staff are prohibited from having devices (e.g., cellular telephones, fans, heaters, etc.) that emit electrical interference in the room.

Triplicate ECGs (approximately 2 minutes apart unless noted otherwise) will be collected in Part 1 and up to 30 subjects in Part 2 and at the Sponsor's discretion (refer to Section 8.6.1 and Table 8–1 for triplicate ECG timepoints), the totality of ECG evaluations during the monotherapy dose escalation will be used to support reduction in sample collections and triplicate ECG sampling may be omitted from the ABBV-706 expansion cohorts. All triplicate ECGs are time-matched to PK sample collection and should be collected immediately prior to the PK sample collection (up to 10 minutes) when scheduled at the same time as the PK collection. The triplicate ECGs should be performed as close to the scheduled time as possible. The date and time (to the nearest minute) of each blood sample collection will be recorded. ECGs occurring near meals will take place prior to meals.

ECG Safety Review:

All ECGs must be evaluated by a qualified physician, preferably a cardiologist (the "local reader"), for the presence of abnormalities and assessment. This reading of the ECG will be used by the investigator for subject safety assessments, including AE determination and management and decision on whether a subject will be discontinued from the study.

The first ECG obtained in the last triplicate set prior to initial dose will serve as the baseline measurement for clinical safety assessment. The first ECG obtained in any triplicate post-dose will be used for clinical safety assessments as will any single ECGs (scheduled or unscheduled) obtained for reasons of safety.

The local reader will sign and date the safety ECG and provide a global interpretation using the following categories:

- Normal ECG

- Abnormal ECG – Not clinically significant (NCS)
- Abnormal ECG – Clinically significant (CS)
- Unable to evaluate

All local reader evaluations of safety ECGs will be entered into the source documents. If the global interpretation is Abnormal (NCS or CS), the local reader will provide further information of the abnormal findings (e.g., sinus bradycardia, arrhythmia). The QT interval corrected for heart rate using Fridericia's formula (QTcF) will be calculated and documented for all ECGs.

All ECG source documentation will be retained at the site. The automatic cardiograph reading (i.e., cardiograph-generated measurements and interpretations) will not be collected for analysis.

ECG Interval Collection and Measurement:

The 12-lead ECG recordings from timepoints requiring triplicate ECGs (as defined in Table 8–1) will be transferred to a third-party ECG vendor and evaluated by electronic ECG/AbbVie BIOsignal System (eECG/ABBIOS), which uses a validated automated signal analysis algorithm to measure predefined ECG intervals (RR, PR, QT, and QRS duration). Further details will be provided in a separate ECG Manual.

8.4.5 Clinical Safety Laboratory Tests

All clinical laboratory samples (Table 10–1) will be collected and assessed locally. Investigators must document their review of each laboratory safety report prior to planned dosing. The baseline laboratory test results for clinical assessment for a particular test will be defined as the last measurement prior to the initial dose of study drug.

Protocol-specific requirements for inclusion or exclusion of subjects are detailed in Protocol Section 5.1 and Section 5.2. For the timing and frequency of clinical laboratory tests to be performed, refer to the SoA (Table 1–1 for Part 1 and Table 1–2 for Parts 2, 3, and 4).

Additional tests may be performed at any time during the study as determined necessary by the investigator or required by local regulations.

Local certified laboratories will be utilized to process and provide results for the clinical laboratory tests. Laboratory reference ranges will be obtained prior to the initiation of the study.

If a laboratory test value is outside the reference range and the investigator considers the laboratory result to be clinically significant, the investigator will:

- Repeat the test to verify the out-of-range value;
- Follow the out-of-range value to a satisfactory clinical resolution; or

- Discontinue the subject from the study intervention or require the subject to receive treatment; in this case, the laboratory result will be recorded as an AE.

The investigator must review the laboratory results, document this review, and record any clinically significant changes occurring during the study as an AE. The laboratory results must be retained with source documents.

Abnormal laboratory findings associated with the underlying disease are not considered clinically significant unless judged by the investigator to be more severe than expected for the subject's condition.

All laboratory tests with values considered clinically abnormal during participation in the study or within 80 days (5 half-lives) after the last dose of study intervention should be repeated until the values return to normal or baseline or are no longer considered clinically significant by the investigator or the CRO's medical monitor.

- If clinically significant values do not return to normal/baseline within a period of time judged reasonable by the investigator, the etiology should be identified, where possible, and the Sponsor notified.
- All protocol-required laboratory tests (defined in Appendix 2, Section 10.2) must be conducted in accordance with the Laboratory Manual and the SoA (Table 1–1 for Part 1 and Table 1–2 for Parts 2, 3, and 4).
- If laboratory values from laboratory tests not specified in the protocol and performed at the institution's local laboratory result in the need for a change in participant management or are considered clinically relevant by the investigator (e.g., are considered to be an SAE or an AE or require dose modification), then the results must be recorded.

8.4.6 Pregnancy Testing

Pregnancy testing should not be performed for postmenopausal women.

A qualitative serum pregnancy test will be performed at Screening and a urine pregnancy test will be performed at baseline (or upon confinement in each period) prior to first dose of study drug for all female subjects of childbearing potential and on Day 1 of every cycle. A serum pregnancy test may be used instead of a urine pregnancy test. A negative serum and urine pregnancy test are required for all female subjects (except postmenopausal) at the Screening Visit or at baseline prior to the first dose of study drug, respectively. A urine pregnancy test will be performed up to 3 days prior to Day 1 of each cycle. The pregnancy test results must be reviewed and determined to be negative prior to dosing with study drug. Serum pregnancy test should be performed at any time a pregnancy is suspected.

If the serum pregnancy test is positive the subject is considered a screen failure. If the serum pregnancy test is borderline, it should be repeated ≥ 3 days later to determine eligibility.

- If the repeat serum pregnancy test is:
- Positive, the subject is considered a screen failure;
- Negative, the subject can be enrolled into the study;
- Still borderline, ≥ 3 days later, this will be considered documentation of continued lack of a positive result and the subject can be enrolled into the study (unless prohibited per local requirements) in the absence of clinical suspicion of pregnancy and other pathological causes of borderline results.

Additional urine pregnancy tests will be performed at visits indicated in the SoA (see Table 1–1 for Part 1 and Table 1–2 for Parts 2, 3, and 4). More frequent pregnancy tests can be performed throughout the study at the investigator's discretion or if required per local/country requirements.

- If the baseline urine pregnancy test is negative, then dosing with study drug may begin.
- If the baseline or post-baseline urine pregnancy test is positive, dosing with study drug must be withheld and a serum pregnancy test is required (as stated above).
- On Day 1 of every cycle, pregnancy testing should be performed during treatment, and then monthly for up to 230 days from the last dose of study drug.

8.5 Adverse Events, Serious Adverse Events, and Other Safety Reporting

The definitions of AEs and SAEs can be found in Appendix 3.

The investigator and any qualified designees are responsible for detecting, documenting, and reporting events that meet the definition of an AE or SAE and are responsible for following up all AEs OR SAEs (see Protocol Section 7). This includes events reported by the subject (or, when appropriate, by a caregiver, surrogate, or the subject's legally authorized representative).

The method of recording, evaluating, and assessing causality of AEs and SAEs and the procedures for completing and transmitting SAE reports are provided in Appendix 3.

8.5.1 Time Period and Frequency for Collecting AE and SAE Information

All serious and nonserious AEs that could be related to study procedures, (e.g., infection at liver biopsy site, done during Screening) will be collected from the time the subject signed the study-specific (main) ICF until study drug administration.

From the time of study drug administration until 80 days (5 half-lives of ABBV-706) after last dose of study drug (for Parts 1, 2, 3b, and 4) or 90 days after last dose of study drug (Part 3a), all AEs and SAEs will be collected whether solicited or spontaneously reported by the subject, and

during the Safety Follow-Up visit. All SAEs that occur during the study, and all SAEs occurring up to 80 days (for Parts 1, 2, 3b, and 4) or 90 days (for Part 3a) after receiving the last dose of study intervention, whether considered to be associated with the study intervention or not, must be reported within 24 hours to the CRO's Safety Contact (refer to Appendix 3, Section 10.3.4 for contact information). All SAEs will be followed until resolution, stabilization, until the event is otherwise explained, or the subject is lost to follow-up (as defined in Protocol Section 7.3).

After 80 days (for Parts 1, 2, 3b, and 4) or 90 days (for Part 3a) following completion of study treatment and throughout the post-treatment period, all spontaneously reported SAEs will be collected (nonserious AEs will not be collected).

8.5.2 Pregnancy

While not an AE, pregnancy in a study subject must be reported to AbbVie Clinical Pharmacovigilance within 24 hours after the site becomes aware of the pregnancy. Subjects who become pregnant during the study must be discontinued (Protocol Section 7). If a pregnancy occurs in a study subject or in the partner of a study subject, information regarding the pregnancy and the outcome will be collected.

In the event of pregnancy occurring in a subject's partner during the study, written informed consent from the partner must be obtained prior to collection of any such information. The Sponsor or designee will provide a separate consent form for this purpose. Pregnancy in a subject's partners will be collected from the date of the first dose through 140 days following the last dose of study drug.

The pregnancy outcome of an elective or spontaneous abortion, stillbirth or congenital anomaly is considered a SAE and must be reported to AbbVie Clinical Pharmacovigilance within 24 hours after the site becomes aware of the event.

8.6 Pharmacokinetics and Immunogenicity

8.6.1 Blood Sample Collection

Blood samples for analysis of ABBV-706 ADC and total antibody in the serum and unconjugated (free) Top1 inhibitor payload in the plasma will be collected at timepoints specified in Table 8–1. Blood samples for analysis of ADA and nAb in the serum will also be collected at timepoints specified in Table 8–1. All predose (0-hour) samples are collected prior to and relative to the respective study drug administration. All other timepoint samples are post-infusion samples and will be collected following the end of ABBV-706 or Budigalimab infusion.

Blood samples for PK should preferably be collected by venipuncture from the contralateral arm used for study drug administration. If a single lumen central catheter is used for study drug administration, blood samples should preferably not be drawn from that catheter. The timing of blood collections will take priority over all other scheduled study activities except for dose administration and ECG collection at visits with triplicate ECGs.

Instructions for the collection and handling of biological samples will be provided by the Sponsor. The actual date and time (24-hour clock time) of every study drug administration and PK sampling will be recorded to the nearest minute on the eCRF.

For Parts 2, 3 and 4, the totality of PK evaluations in Part 1 will be used support reduction in sample collections and specific PK sampling timepoints may be omitted from the dose expansion and combinations as deemed appropriate.

If a participant refuses blood collection for PK analysis, this will be considered a protocol deviation as the PK analysis is a primary objective of the study.

Table 8–1 Sampling Schedule of ABBV-706 PK, ECG, and ADA Assessments

	Cycle 1					Cycle 2		Cycle 3					Cycles 4, 6, 9, and 12	EOT
**Activity**	**Day 1**	**Day 2**	**Day 3**	**Day 8 (±1 day)**	**Day 15 (±1 day)**	**Day 1 (+1 day)**	**Day 2**	**Day 1 (+1 day)**	**Day 2**	**Day 3 (±1 day)**	**Day 8 (±1 day)**	**Day 15 (±1 day)**	**Day 1 (±1 day)**	**30 days after last dose (±15 days)**
**Part 1: Monotherapy Dose Escalation**<br>**Part 2: Monotherapy Dose Optimization & Expansion**														
ABBV-706 PK sampling[a,b]	Predose<br>15 min<br>2 hours<br>4 hours	✓	✓	✓	✓	Predose<br>15 min	✓	Predose<br>15 min<br>2 hours	✓	✓	✓	✓	Predose<br>15 min[c]	✓
Triplicate ECG[a] (Part 1 and up to 30 subjects in Part 2 only) (Not collected for subjects in mainland China)	Predose<br>15 min<br>2 hours	✓	✓	✓	✓	Predose<br>15 min	✓	Predose<br>15 min<br>2 hours	✓	✓	✓	✓		
**Part 3: Platinum and Budigalimab Combinations**<br>**Part 4: High-grade NECs/NETs and CNS tumors Expansions**														
ABBV-706 PK sampling[a,b]	[redacted]													
Budigalimab PK sampling[a,b] (Part 3a only)														
**All Subjects**														
ABBV-706 ADA/nAb														
Budigalimab ADA/nAb (Part 3a only)														

ADA = antidrug antibody; CNS = central nervous system; ECG = electrocardiogram; nAb = neutralizing antidrug antibody; NEC = neuroendocrine carcinoma; NETs = neuroendocrine tumors; PK = pharmacokinetics; Q3C = every three cycles.

Note: All timepoint samples are post-infusion samples and collected following the end of ABBV-706 or Budigalimab infusion.

a. At the Sponsor's discretion, the totality of PK/triplicate ECG evaluations during Part 1a monotherapy dose escalation will be used to support reduction in sample collections and specific PK and triplicate ECG sampling timepoint(s) may be omitted from the ABBV-706 expansion and combination cohorts (Parts 2, 3, and 4). If elected, the Sponsor will communicate to sites PK/triplicate ECG sampling timepoints to be followed via a memo.

b. Allowable sample collection windows are as follows: Predose = within 2 hours prior to next dose; 15 min ±5 min; 2 and 4 hours ±15 min; 24 and 48 hours (Days 2 and 3) ±2 hours; 168 and 336 hours (Days 8 and 15) ±24 hours.

c. Only for patients enrolled in Part 2a; collected only on Day 1 of Cycles 6 and 12.

8.6.2 PK/ADA Sample Disposition

Whole blood will be collected into appropriately labeled tubes and processed as outlined in the most current version of the Laboratory Manual for analysis. Samples will be shipped to the central laboratory. Additional information on the collection, handling/processing, and disposition methods can be found in the Laboratory Manual.

Serum concentrations of ABBV-706 (total antibody and ADC), plasma concentrations of the unconjugated Top1 inhibitor payload, and relative titers of serum ABBV-706 ADA will be determined using validated methods at or under the supervision of the Sponsor's Bioanalysis Department. Additional analytes, including possible ABBV-706 metabolite (s) may be determined with validated or non-validated methods. Serum and plasma samples collected for ABBV-706 PK and ABBV-706 ADA or nAb analysis may be used for future assay development or validation activities.

8.6.3 Immunogenicity Assessments

Antibodies to ABBV-706 will be evaluated in plasma samples collected from all subjects according to the SoA (Table 1–1 for Part 1 and Table 1–2 for Parts 2, 3, and 4). Additionally, serum samples should also be collected at the final visit from subjects who discontinued study intervention or were withdrawn from the study. These samples will be tested by the Sponsor or Sponsor's designee.

Serum samples will be screened for antibodies binding to ABBV-706 and the titer of confirmed positive samples will be reported. Other analyses may be performed to verify the stability of antibodies to ABBV-706 and/or further characterize the immunogenicity of ABBV-706. Antibodies may be further characterized and/or evaluated for their ability to neutralize the activity of the study intervention(s). Other analyses may be performed to further characterize the immunogenicity of ABBV-706.

8.7 Pharmacodynamics

Biospecimens (blood, plasma, serum, and tissue biopsy) will be collected at timepoints indicated in the SoA (see Table 1–1 for Part 1 and Table 1–2 for Parts 2, 3, and 4). Samples will be analyzed to evaluate the pharmacologic effects of ABBV-706 on its target including (but not limited to) DNA damage and changes in tumor burden. Pharmacodynamic analysis may provide information on proof of mechanism (i.e., does ABBV-706 hit its intended target) and selection of optimal dosing.

8.8 Genetics

No germline genetic testing will be performed in this study. Please refer to Protocol Section 8.9 for additional information.

8.9 Biomarkers

An archival tissue specimen or fresh tumor biopsy is required (if archival tissue is not available and obtaining a fresh biopsy poses significant risk to the safety of the subject, the treating physician can discuss the case with the medical monitor or Sponsor to determine eligibility) and must be sent to a designated lab for testing of SEZ6 protein expression (refer to Laboratory Manual for details). This sample may be used to inform selection of optimal cut-off. Biopsies of any lesions that are in proximity to any vital neurovascular structures that can be considered high risk procedures should not be performed.

Biospecimens (blood, plasma, serum and tissue biopsy) will be collected at timepoints indicated in the SoA (Table 1–1 for Part 1 and Table 1–2 for Parts 2, 3, and 4) to evaluate known and/or novel disease-related or drug-related biomarkers in circulation or at tissue sites. Types of biomarkers assessments may include nucleic acids, proteins, lipids, and/or metabolites, either free or in association with particular cell types, related to the pathway(s) targeted by the study drug, or those believed to be related to the disease(s) being studied. These assessments will include evaluation of the potential link between biomarkers of tumor tissues, such as the target of ABBV-706 (i.e., SEZ6), presence of specific molecular abnormalities and the biological activity of ABBV-706 alone or in combination with budigalimab or platinum chemotherapy. In addition, the potential link between biomarkers in blood including (but not limited to) ctDNA, circulating peptides, and the biological activity of ABBV-706 alone or in combination with budigalimab or platinum chemotherapy will be evaluated. The information learned from these assessments may be used to investigate factors influencing response to treatment, scientific questions related to SCLC and other neuroendocrine tumors and/or in the development of new therapies and diagnostic tests. Some of these sample collections may be optional.

Blood volumes required for biomarker assessments will be described in the Central Laboratory Services Manual and provided to the sites participating in the study. All biomarker samples should be labeled and shipped as outlined in the Laboratory Manual. The Sponsor (or people or companies working with the Sponsor) will store the samples and data in a secure storage space with measures to protect confidentiality. The samples may be retained while research on ABBV-706 (or drugs of this class) or advanced solid tumors and related conditions continues, but for no longer than 20 years after study completion, or per local requirement, and then destroyed.

Biomarker research may be exploratory in nature and the results may not be included with the clinical study report; the results may not be suitable for clinical decision making or patient management.

Provision of biospecimens for biomarker research is mandatory, but they will not be collected from sites where local regulations do not allow for the collection, use, and storage of samples as described in the protocol.

8.10 Storage and Future Use of Biological Samples

AbbVie will be responsible for ensuring all biological sample collection and storage is secure and with adequate measures to protect confidentiality, per all applicable local regulations. AbbVie will store biological samples related to plasma, serum, or tumor tissue samples collected as part of the PK/pharmacodynamic/biomarker/immunogenicity assessments. With participants' consent, samples may be used for further research by AbbVie, per all applicable local regulations.

9 Statistical Considerations

The analysis and reporting will be done on all data from all subjects at the time the study ends.

The statistical analysis plan (SAP) will include a more technical and detailed description of the statistical analyses described in this section. This section is a summary of the planned statistical analyses of the most important endpoints including primary and key secondary endpoints.

9.1 Statistical Hypotheses

No hypotheses are planned to be tested.

9.2 Analysis Sets

The analysis sets to be used are defined in Table 9–1: The Full analysis set (FAS) will be used for all safety and baseline analyses.

For the efficacy analyses, subjects will be included in the analyses according to the planned investigational intervention; whereas for safety and PK analyses, subjects will be included in the analyses according to the investigational intervention they actually received.

Table 9–1 Subject Analysis Sets

Subject Analysis Set	Description
Full analysis set (FAS)	All subjects who received at least 1 dose of study drug.
Response-evaluable Analysis Set	Subjects in the FAS.
Pharmacokinetic Analysis Set	Subjects in the FAS who have at least 1 quantifiable ABBV-706 concentration.

9.3 Statistical Analyses

The primary and exploratory endpoints are listed in Protocol Section 3. The statistical methods provided in this protocol will be focused on primary and secondary analyses. Complete and specific details of the statistical analysis will be described in the statistical analysis plan (SAP).

9.3.1 General Considerations

The estimand corresponding to antitumor activity endpoint is the objective response rate (ORR), defined as the proportion of subjects with a confirmed best overall response of CR and PR per investigator review according to RECIST version 1.1 for non-CNS primary tumors and RANO for CNS primary tumors, regardless of reasons for study drug discontinuation, and prior to start of subsequent therapy in the subjects receiving at least one dose of ABBV-706.

The estimand will be analyzed considering the following potential intercurrent events (ICEs):

- ICE1: Subjects with premature discontinuation of study drug due to any reason.
- ICE2: Subjects with the use of subsequent anticancer therapy and will be handled based on the following strategies:
- ICE 1: The disease assessments after ICE1 will be used for the derivation of endpoints.
- ICE 2: The disease assessments after ICE2 will not be used for the derivation of endpoints.

9.3.2 Efficacy Analysis

The FAS will be used for all efficacy analyses. Details of the analysis methods will be provided in the SAP.

The point estimates along with two-sided 90% confidence intervals (CIs) for ORR will be provided based on the Clopper-Pearson (exact) method.[20] Efficacy analysis will be presented separately by treatment, and dose level, for Parts 1, 2(a), 3a, 3b, 4a, and 4b.

PFS is defined as time from first study treatment to a documented disease progression according to RECIST version 1.1, as determined by the investigator, or death due to any cause, whichever occurs earlier. If the subject has not experienced disease progression or died, PFS will be

censored at the last adequate disease assessment prior to the data cut-off date or start of any subsequent anticancer therapy, whichever occurs earlier. For subjects without baseline disease assessment or any adequate post-baseline disease assessments who did not die, PFS will be censored at the date of the first study treatment (Day 1).

DOR is defined for subjects achieving a confirmed CR+PR as the time from the initial response of CR+PR per investigator review according to RECIST 1.1 criteria to disease progression or death of any cause, whichever occurs earlier. Subjects without progression or death of any cause will be censored using the same censoring rules defined for PFS analysis.

OS is defined as time from first study treatment to death due to any cause. For subjects who are not known to have died at the time of analysis cut-off, OS will be censored at the last known alive date on or prior to the data cut-off.

Kaplan-Meier estimates for the median of DOR, PFS, and OS and their associated two-sided 90% CIs will be provided separately by dose cohort in Part 1 and Part 2a and by expansion cohorts in Parts 3 and 4.

9.3.3 Safety Analyses

The safety endpoints are described in Protocol Section 3. The treatment-emergent period is defined as the time interval from the date of first study treatment until 80 days (5 half-lives of ABBV-706) after the last dose of study treatment (for Parts 1, 2, 3b, and 4) or 90 days after last dose of study treatment (for Part 3a). The FAS will be used for all safety analyses and details will be provided in the SAP.

9.3.3.1 Analysis of Adverse Events (AEs)

The incidence of treatment-emergent AEs will be summarized using primary Medical Dictionary for Regulatory Activities (MedDRA) System Organ Classes (SOCs) and preferred terms according to the version of the MedDRA coding dictionary used for the study at the time of database lock. The number and percentage of subjects experiencing any AEs, SAEs, drug-related AEs, AEs leading to death or study treatment discontinuation will be presented overall, then by dose cohort in Part 1 and Part 2a or by expansion cohorts in Parts 3 and 4.

Specific AEs will be counted once for each subject for calculating percentages, unless stated otherwise. In addition, if the same AE occurs multiple times within a subject, the maximum severity and level of relationship to study drug will be reported.

9.3.3.2 Analysis of Laboratory Data

Baseline values and changes from baseline will be summarized using descriptive statistics for each scheduled post-baseline visit for laboratory data overall, then by dose cohort in Part 1 and Part 2a or by expansion cohorts in Parts 3 and 4.

Changes in selected laboratory parameters will also be tabulated using shift tables either by NCI CTC criteria or categorized as low, normal, or high based on the laboratory normal ranges, in the case NCI CTC criteria is not applicable to the lab parameters.

9.3.3.3 Analysis of Other Safety Data

Baseline values and changes from baseline will be summarized for each scheduled post-baseline visit for vital signs and ECG data.

9.3.4 Pharmacokinetic Analyses

The PK parameters (C_{max}, T_{max}, $t_{1/2}$, and AUC) will be determined using noncompartmental methods for ABBV-706 ADC, total antibody and free payload, and budigalimab, and summarized. Additional parameters may be calculated if deemed appropriate and useful in data interpretation.

9.4 Interim Analyses

This is an open-label study, study data will be continuously monitored.

9.4.1 Futility Analysis

Once 20 subjects are efficacy evaluable in Part 2 (by dose level) or Part 4 (4a or 4b), futility analysis will be performed. Using the posterior probability based on the available data, if there is an 80% or more chance that the unconfirmed response (PR or CR by RECIST, PPR better by RANO) rate is 20% or less in evaluable patients, then enrollment in that group will pause and patient benefit assessment will be performed before any additional subjects are enrolled in that part of the study. The posterior probability will be calculated using a beta (0.5, 0.5) prior. This corresponds to a stopping threshold of 2 or fewer responders out of 20 subjects. Subjects will be considered efficacy evaluable if they have either discontinued treatment (assumed to be non-responsive) or have had their first post-baseline scan.

9.5 Overall Type I Error Control

Not applicable for this study.

9.6 Sample Size Determination

The total number of subjects will be approximately 319. This is distributed into the following components:

For Part 1a (Monotherapy Dose Escalation), up to 60 subjects with solid tumors will be enrolled; for Part 1b (Monotherapy Dose Escalation for Japan), up to 24 subjects with solid tumors will be enrolled in Japan only. For Part 2, up to 95 subjects (up to 80 subjects globally and up to 15 subjects in China only) will be enrolled depending on the number and size of the randomized dose optimization cohorts and/or line of therapy. Subjects in Part 2a will be randomized evenly between at least 2 dose cohorts.

In Parts 3a and 3b, up to 30 and 30 subjects will be enrolled respectively and is sufficient for an initial safety dataset to enable future efficacy studies. Parts 4a and 4b will enroll up to 40 subjects each.

Table 9–2 below provides the lower bound of a one-sided 80% CI for ORR corresponding to various observed response rates assuming a total of 40 treated subjects.[21] For example, if a 27.5% response rate is observed with 40 treated subjects, the lower bound of a one-sided 80% exact CI will exclude 20% (or lower) rates of response, providing some preliminary assessment of efficacy.

Table 9–2 Exact 1-Sided 80% Confidence Intervals From Various Observed Objective Response Rates Given a Cohort Size of 40 Subjects

Cohort size (n)	Number of Responders	Observed Objective Response Rate	Lower Bound of 1-sided 80% Exact Confidence Interval
40	10	25.0%	18.7%
	11	27.5%	20.9%
	12	30.0%	23.2%
	13	32.5%	25.5%
	14	35.0%	27.8%
	15	37.5%	30.2%
	16	40.0%	32.6%
	17	42.5%	34.9%
	18	45.0%	37.3%
	19	47.5%	39.8%
	20	50.0%	42.2%

10 Supporting Documentation and Operational Considerations

10.1 Appendix 1: Regulatory, Ethical, and Study Oversight Considerations

10.1.1 Regulatory and Ethical Considerations

The study will be conducted in accordance with the protocol, International Council for Harmonization (ICH) guidelines, applicable regulations, and guidelines governing clinical study conduct and the ethical principles that have their origin in the Declaration of Helsinki. Responsibilities of the investigator are specified in Section 10.13. Investigators should notify the Sponsor or the Sponsor's designated agent if any urgent safety measures are taken to protect the subjects against any immediate hazard.

The protocol, ICF(s), recruitment materials, and all subject materials will be submitted to the IEC/IRB for review and approval. Approval of both the protocol and the ICF(s) must be obtained before any subject is enrolled. Any amendment to the protocol will require review and approval by the IEC/IRB before the changes are implemented to the study. In addition, all changes to the ICF(s) will be IEC/IRB approved.

In the event a significant disaster/crisis (e.g., epidemic/pandemic, natural disaster, conflict/combat) occurs leading to difficulties in performing protocol-specified procedures, the Sponsor or designee may engage with study site personnel in efforts to ensure the safety of subjects, maintain protocol compliance, and minimize risks to the integrity of the study while trying to best manage subject continuity of care. This may include alternative methods for assessments (e.g., phone contacts or virtual site visits), alternative locations for data collection (e.g., use of a local lab instead of a central lab), and shipping supplies direct to subjects to ensure continuity of treatment where allowed. In all cases, these alternative measures must be allowed by local regulations and permitted by IRB/IEC. Investigators should notify the Sponsor or designee if any urgent safety measures are taken to protect the subjects against any immediate hazard.

10.1.2 Data Protection

The Sponsor will ensure that the clinical trial is conducted with a quality management system that will define quality tolerance limits in order to ensure human subject protection and reliability of study results. Data will be generated, documented, and reported in compliance with the protocol, ICH Good Clinical Practice (GCP), and applicable regulatory requirements.

The investigator will assign a unique identifier or code to each subject to be used in lieu of the subject's name in study documentation and in reporting of AEs for the purpose of ensuring the confidential treatment of the study participant's personal and health information. The investigator

will maintain a subject identifier list in a secure location at the study site consisting of the unique subject identifiers, subject names, and dates of birth to allow unambiguous identification of each subject included in the study. The subject identifier list shall be retained by the investigator for a minimum of 15 years following the completion of the study.

Any subject records or datasets that are transferred to the Sponsor will contain the identifier only; subject names or any information which would make the subject identifiable will not be transferred.

The subject must be informed that their personal study-related data will be used by the Sponsor in accordance with local data protection law. The level of disclosure must also be explained to the subject who will be required to give consent for their data to be used as described in the informed consent.

The subject must be informed that their medical records may be examined by the Sponsor, its representatives, collaborators, licensees and assigns, auditors or other authorized personnel appointed by appropriate IRB/IEC members, and regulatory authorities for purposes of conducting and monitoring the study, regulatory submissions and conducting future research related to the diseases or study drug covered by this protocol.

The contract between Sponsor and study sites specifies responsibilities of the parties related data protection, including handling of data security breaches and respective communication and cooperation of the parties.

Information technology systems used to collect, process, and store study-related data are secured by technical and organizational security measures designed to protect such data.

10.1.3 Data Handling

The investigator is responsible for ensuring the accuracy, completeness, legibility, and timeliness of the data reported. All source documents should be attributable, legible, contemporaneous, original, accurate, and complete to ensure accurate interpretation of data. Clinical site monitoring is conducted to ensure that the rights and well-being of human subjects are protected, that the reported trial data are accurate, complete, and verifiable, and that the conduct of the trial is in compliance with the currently approved protocol, ICH GCP, and applicable local regulatory requirement(s), including EU Clinical Trial Regulation.

It is recommended the eCRFs be captured in the Sponsor's EDC system within 10 business days of the subject's visit to the site. During the dose escalation phase of the study, data may be required to be captured in the EDC system more frequently to support dose escalation decision making. Data reported on the eCRF that is derived from source documents should be consistent with the source documents or the discrepancies must be explained.

Any change or correction to an eCRF must be maintained in an audit trail within the EDC system. Data changes may only be made by those individuals so authorized. The investigator must retain records of the changes and corrections, written and/or electronic. Prior to database lock, the investigator must review and approve the completed electronic eCRFs to verify their accuracy. During the COVID-19 pandemic, remote data review/verification may be employed if allowed by the local regulatory authority, IRB/IEC, and the study site.

10.1.4 Investigator Record Retention

The investigator shall retain study drug disposition records and all source documentation (such as original ECG tracings, laboratory reports, and inpatient or office patient records) for the maximum period required by the country and institution in which the study will be conducted, or for the following periods, whichever is longer: (a) 2 years after the last approval of a marketing application in an ICH region and until there are no pending or contemplated marketing applications in an ICH region, or (b) 2 years after the relevant authority has been informed of the discontinuation of clinical development of the study drug. The investigator should not destroy any records associated with the study without obtaining the prior, written consent of the Sponsor. If the investigator withdraws from the study (due to relocation, retirement, etc.), the study records should be transferred to a mutually agreed upon designee, such as another investigator or IRB/IEC. No records shall be transferred without prior, written authorization from the Sponsor or its designee.

10.1.5 Data Quality Assurance

The Sponsor or designee will ensure that the clinical study is conducted with a quality management system that will define quality tolerance limits to ensure human subject protection and reliability of study results. Data will be generated, documented, and reported in compliance with the protocol, ICH GCP, and applicable regulatory requirements, including EU Clinical Trial Regulation. During the COVID-19 pandemic, remote data review/verification may be employed if allowed by the local regulatory authority, IRB/IEC, and the study site.

Monitoring details describing strategies, including definition of study critical data items and processes (e.g., risk-based initiatives in operations and quality such as risk management and mitigation strategies and analytical risk-based monitoring), methods, responsibilities, and requirements, including handling of noncompliance issues and monitoring techniques (central, remote, or on-site monitoring) are provided in the Monitoring Plan.

All data generated by the site personnel will be captured electronically at each study center using eCRFs. Data from external sources (such as central laboratory data) will be imported into the database. The investigator is responsible for verifying that data entries are accurate and correct by physically or electronically signing the case report form (CRF). Once the eCRF clinical data

have been submitted to the central server at the independent data center, corrections to the data fields will be captured in an audit trail. The reason for change, the name of the person who performed the change, together with the time and date will be logged to provide an audit trail.

The specific procedures to be used for data entry and query resolution using the EDC system/eCRF will be provided to study sites in the Data Entry Guidelines. In addition, site personnel will receive training on the EDC system/eCRF.

10.1.6 Source Documents and Access to Source Data

Source documents provide evidence for the existence of the subject and substantiate the integrity of the data collected. Source documents are filed at the investigator's site.

Data reported in the eCRF that are transcribed from source documents must be consistent with the source documents or the discrepancies must be explained. The investigator may need to request previous medical records or transfer records, depending on the study. Also, current medical records must be available.

The investigator is responsible for ensuring the accuracy, completeness, legibility, and timeliness of the data reported. All source documents should be attributable, legible, contemporaneous, original, accurate, and complete to ensure accurate interpretation of data.

The Sponsor or designee will perform monitoring to confirm that data entered into the eCRF by authorized site personnel are accurate, complete, and verifiable from source documents; that the safety and rights of subjects are being protected; and that the study is being conducted in accordance with the currently approved protocol and any other study agreements, ICH GCP, and all applicable regulatory requirements.

During the study, a monitor will make site visits to review protocol compliance, compare eCRF entries and individual subject's medical records, assess drug accountability, and ensure that the study is being conducted according to pertinent regulatory requirements. eCRF entries will be verified with source documentation. The review of medical records will be performed in a manner to ensure that subject confidentiality is maintained.

Checking of the eCRF entries for completeness and clarity, and cross-checking with source documents, will be required to monitor the progress of the study. Moreover, regulatory authorities of certain countries, IRBs, IECs, and/or the Sponsor's Clinical Quality Assurance Group may wish to carry out such source data checks and/or on-site audit inspections. Direct access to source data will be required for these inspections and audits; they will be carried out giving due consideration to data protection and medical confidentiality. The investigator assures the Sponsor, and the CRO if involved in monitoring/data management, of the necessary support at all times.

10.1.7 Study and Site Start and Closure

First Act of Recruitment

The start-of-study is defined as the date of the first site activated.

The first act of recruitment is the first subject signing first informed consent in line with first subject first visit and will be the study start date.

The end-of-study is defined as the date of end-of-study participation by the last subject in the last country where the study was conducted.

Study/Site Termination

The Sponsor reserves the right to close the study site or terminate the study. Study sites will be closed upon study completion. A study site is considered closed when all required documents and study supplies have been collected and a study site closure visit has been performed.

The investigator may initiate study site closure at any time, provided there is reasonable cause and sufficient notice is given in advance of the intended termination.

If the study is prematurely terminated or suspended, the Sponsor shall promptly inform the investigators, the IECs/IRBs, the regulatory authorities, and any CROs used in the study of the reason for termination or suspension, as specified by the applicable regulatory requirements. The investigator shall promptly inform the subject and should ensure appropriate therapy and/or follow-up.

10.1.8 Publication Policy

The investigator and the institution shall collect and record all data and results of the study, including all information, CRFs, laboratory work sheets, slides, biospecimens, findings and reports ("Results") as required by this protocol, the Sponsor's instructions and the clinical trial agreement. Results are the exclusive property of Sponsor and are deemed its confidential information. Subject medical records and other source documentation are not included in Results. The written permission of the Sponsor is required prior to disclosure by the investigator or the study sites of Results or any other information related to the study or the study drug. After conclusion of the study at all study sites, the investigator may publish or present the Results that have been generated at the institution, provided that, the Results of the overall study have already been published or disclosed with Sponsor's permission in an abstract, manuscript or presentation; or the overall study has been completed at all sites for at least 2 years; or as otherwise approved by Sponsor in writing. The investigator will submit to Sponsor for review any proposed publication or presentation along with the name of the applicable scientific journal or presentation forum at least 60 days prior to its submission for publication or presentation. The

investigator will comply with the Sponsor's request to delete references to its confidential information in any proposed publication or presentation and the investigator or institution will withhold publication or presentation for an additional 60 days in order to allow the Sponsor to obtain patent protection. Investigators will conduct all interactions with third parties, including journal editors and reviewers, in a manner consistent with the confidentiality provisions of the clinical trial agreement in place with Sponsor. The foregoing shall not operate to restrict publication, but is intended solely to ensure the accuracy and completeness of data intended for publication, to provide an opportunity to share with the investigator any new and/or unpublished information of which he/she may be unaware and to ensure that Sponsor's confidential information is not disclosed. Sponsor shall have the right to use, share, and copy such publications and portions of such publications for its business purposes.

10.1.9 Protocol Approval and Amendment

Before the start of the study, the study protocol and/or other relevant documents will be approved by the IEC/IRB/Competent Authorities, in accordance with local legal requirements. The Sponsor must ensure that all ethical and legal requirements have been met before the first patient is enrolled in the study.

The Sponsor does not allow intentional/prospective deviations from the protocol except when necessary to eliminate an immediate hazard to study subjects. The investigator is responsible for complying with all protocol requirements, written instructions, and applicable laws regarding protocol deviations. If a protocol deviation occurs (or is identified, including those that may be due to the COVID-19 pandemic), the investigator is responsible for notifying IEC/IRB, regulatory authorities (as applicable), and the Sponsor.

If an amendment to the protocol is required, it must be written, receive approval from the appropriate personnel, and receive IRB/IEC/Competent Authority approval prior to its release to investigators for implementation (if appropriate). The approved protocol amendment(s) will also be submitted to the IND under which the study is being conducted.

Administrative changes (not affecting the patient benefit/risk ratio) may be made without the need for a formal amendment. All amendments will be distributed to all protocol recipients, with appropriate instructions.

10.2 Appendix 2: Clinical Laboratory Tests

All clinical laboratory samples (Table 10–1) will be collected and assessed locally at the scheduled time points outlined. Investigators must document their review of each laboratory safety report prior to planned dosing. Additional tests may be performed at any time during the study as determined necessary by the investigator or required by local regulations.

Protocol-specific clinical laboratory requirements for inclusion or exclusion of subjects are detailed in Protocol Section 5.1 and Section 5.2.

Table 10–1 Protocol-Required Safety Laboratory Tests

**Hematology**	
White blood cell (WBC) count	Lymphocytes (absolute count)
Red blood cell (RBC) count	Monocytes (absolute count)
Hemoglobin	Eosinophils (absolute count)
Platelet count	Basophils (absolute count)
Neutrophils (absolute count)	Band neutrophils (absolute count)
**Coagulation**	
Prothrombin time (PT)	International Normalized Ratio (INR)
Activated partial thromboplastin time (aPTT)	
**Clinical Chemistry**	
Alanine aminotransferase (ALT / SGPT)	Creatinine clearance (MDRD calculation)
Albumin	Gamma glutamyl transferase (GGT)
Alkaline phosphatase (ALP)	Glucose
Amylase[a,g]	Inorganic Phosphorus
Aspartate aminotransferase (AST / SGOT)	Lactate dehydrogenase (LDH)[a]
Bicarbonate/carbon dioxide	Lipase[a,g]
Bilirubin: Direct, Indirect, Total	Magnesium
Blood urea nitrogen (BUN)	Potassium
Calcium	Sodium
Chloride	Total protein
Creatinine	
**Urinalysis**	
Bilirubin	Blood
Glucose	pH
Ketones	Protein
Leukocyte esterase (not required unless urinary tract infection suspected)	Urobilinogen<br>Specific gravity
Nitrite	Microscopic (reflex)
**Hormone Levels (Part 3a Budigalimab combination only)**	
Thyroid stimulating hormone (TSH)	Free thyroxine (T4)[e]
Triiodothyronine (T3)[e]	Cortisol (if indicated)
	Adrenocorticotropic hormone (ACTH) (if indicated)
**Pregnancy Testing**	
Serum/urine human beta chorionic gonadotrophin[b,c,d]	
Follicle-stimulating hormone[a,b,f]	

SGOT = serum glutamic-oxaloacetic transaminase; SGPT = serum glutamic-pyruvate transaminase.

a. Performed only at Screening.
b. Females only.
c. Pregnancy testing is not required for females of non-childbearing potential.
d. A serum pregnancy test may be used instead of a urine pregnancy test during the study. Serum pregnancy test should be performed at any time a pregnancy is suspected.
e. Free T3 and Total T4 may be performed per institutional practice.
f. If needed to determine postmenopausal status.
g. Repeat after screening if clinically indicated.

10.3 Appendix 3: AEs and SAEs: Definitions and Procedures for Recording, Evaluating, Follow-up, and Reporting

10.3.1 Definition of AE

An AE is any untoward medical occurrence in a subject or clinical investigation subject administered a pharmaceutical product and which does not necessarily have a causal relationship with this treatment. An AE can therefore be any unfavorable and unintended sign (including an abnormal laboratory finding), symptom, or disease temporally associated with the use of a medicinal (investigational) product, whether or not the event is considered causally related to the use of the product.

Such an event can result from use of the drug as stipulated in the protocol or labeling, as well as from "special situations" such as accidental or intentional overdose, medication error, occupational or accidental exposure, off-label use, drug abuse, drug misuse, or drug withdrawal, all which must be reported whether associated with an AE or not. Any worsening of a pre-existing condition or illness is considered an AE. Worsening in severity of a reported AE should be reported as a new AE. Laboratory abnormalities and changes in vital signs are considered to be AEs only if they result in discontinuation from the study, necessitate therapeutic medical intervention, meets protocol-specific criteria (see Section 6.7 regarding toxicity management), and/or if the investigator considers them to be clinically significant.

The investigators will monitor each subject for clinical and laboratory evidence of AEs on a routine basis throughout the study. All AEs will be followed to a satisfactory conclusion.

An elective surgery/procedure scheduled to occur during a study will not be considered an AE if the surgery/procedure is being performed for a pre-existing condition and/or the surgery/procedure has been pre-planned prior to study entry. However, if the pre-existing condition deteriorates unexpectedly during the study (e.g., surgery performed earlier than planned), then the deterioration of the condition for which the elective surgery/procedure is being done will be considered an AE.

Events of disease progression related to the primary malignancy being evaluated in this study and accompanying symptoms, if clearly associated with disease progression, will not be collected as AEs unless the event(s) result in death or qualifies as a SAE as defined in Section 10.3.2 and require reporting in the same manner as any SAE.

If any of the following events are reported, then the following supplemental report must be completed.

Event	Supplemental Report
Discontinuation or interruption of study drug due to a hepatic AE<br>A hepatic SAE<br>ALT/AST > 8 × ULN or ALT/AST > 3 × ULN with a total bilirubin > 2 × ULN	Hepatic AE eCRF
Pneumonitis/Interstitial Lung Disease (ILD)	Pneumonitis/ILD eCRF
Infusion-related reaction (IRR)/Hypersensitivity reactions	IRR eCRF
COVID-19 infection	COVID-19 Status eCRF<br>COVID-19 Supplemental Signs and Symptoms eCRF<br>COVID-19 Vaccine Details eCRF

AE = adverse event; ALT = alanine aminotransferase; AST = aspartate aminotransferase; eCRF = electronic case report form; ULN = upper limit of normal

10.3.2 Definition of SAE

If an AE, whether associated with study drug or not, meets any of the following criteria, it is to be reported to AbbVie Clinical Pharmacovigilance as an SAE within 24 hours of the site being made aware of the SAE (refer to Appendix 3, Section 10.3.4 for reporting details and contact information):

**Death of Subject**	An event that results in the death of a subject.
**Life-Threatening**	An event that, in the opinion of the investigator, would have resulted in immediate fatality if medical intervention had not been taken. This does not include an event that would have been fatal if it had occurred in a more severe form.
**Requires inpatient hospitalization or prolongation of existing hospitalization**	An event that results in an admission to the hospital for any length of time or prolongs the subject's hospital stay. This does not include an emergency room visit or admission to an outpatient facility
**Is a congenital anomaly/birth defect**	An anomaly detected at or after birth, or any anomaly that results in fetal loss.
**Persistent or Significant Disability/Incapacity**	An event that results in a condition that substantially interferes with the activities of daily living of a study subject. Disability is not intended to include experiences of relatively minor medical significance

	such as headache, nausea, vomiting, diarrhea, influenza, and accidental trauma (e.g., sprained ankle).
**Important Medical Event Requiring Medical or Surgical Intervention to Prevent Serious Outcome**	An important medical event that may not be immediately life-threatening or result in death or hospitalization but based on medical judgment may jeopardize the subject and may require medical or surgical intervention to prevent any of the outcomes listed above (i.e., death of subject, life-threatening, hospitalization, prolongation of hospitalization, congenital anomaly, or persistent or significant disability/incapacity). Additionally, any elective or spontaneous abortion or stillbirth is considered an important medical event along with any suspected transmission of an infectious agent via a medicinal product if no other serious criterion is applicable. Examples of such events include allergic bronchospasm requiring intensive treatment in an emergency room or at home, blood dyscrasias or convulsions that do not result in inpatient hospitalization, or the development of drug dependency or drug abuse.

All AEs and SAEs reported from the time of study drug administration until 80 days (5 half-lives of ABBV-706) after last dose of study drug (for Parts 1, 2, 3b, and 4), or 90 days after last dose of study drug (Part 3a) will be collected, whether solicited or spontaneously reported by the subject. In addition, study procedure-related serious and nonserious AEs will be collected from the time the subject signs the study-specific informed consent until study drug administration. After 80 days (for Parts 1, 2, 3b, and 4) or 90 days (for Part 3a) following completion of study treatment and throughout the posttreatment period, all spontaneously reported SAEs will be collected (nonserious AEs will not be collected).

The following definitions will be used for Serious Adverse Reactions (SAR) and Suspected Unexpected Serious Adverse Reaction (SUSAR):

**SAR**	Defined as all noxious and unintended responses to an IMP related to any dose administered that result in an SAE as defined above.
**SUSAR**	Refers to individual SAE case reports from clinical trials where a causal relationship between the SAE and the IMP was suspected by either the Sponsor or designee or the investigator, is unexpected (not listed in the applicable RSI) and meets one of the above serious criteria.

AbbVie or designee will be responsible for SUSAR reporting for the Investigational Medicinal Product (IMP) in accordance with global and local requirements.

Suspected unexpected serious adverse reactions reporting will be in adherence to requirements of EU pharmacovigilance legislation, clinical trial legislation and guidance. EU CTR 536/2014; CT-3 and all other applicable local regulations.

Adverse events will be monitored throughout the study to identify any of special interest that may indicate a trend or risk to subjects.

10.3.3 Recording and Follow-up of AE and SAE

AE and SAE Recording

When an AE/SAE occurs, it is the responsibility of the investigator to review all documentation (e.g., hospital progress notes, laboratory, and diagnostics reports) related to the event.

The investigator will then record all relevant AE/SAE information (event description, date of onset, grade, judgment on continuation of ABBV-706 treatment, presence or absence of treatment, seriousness, causal relationship with ABBV-706, and outcome at the final observation [date of resolution if resolved by the final observation]) in EDC.

It is not acceptable for the investigator to send photocopies of the subject's medical records to the Sponsor in lieu of completion of the Sponsor required form.

There may be instances when copies of medical records for certain cases are requested by the Sponsor. In this case, all subject identifiers, with the exception of the subject number, will be blinded on the copies of the medical records before submission to the Sponsor.

The investigator will attempt to establish a diagnosis of the event based on signs, symptoms, and/or other clinical information. In such cases, the diagnosis (not the individual signs/symptoms) will be documented as the AE/SAE.

Assessment of Severity

The investigator will rate the severity of each AE according to the National Cancer Institute Common Terminology Criteria for Adverse Events (NCI CTCAE) Version 5.0. If a reported AE increases in severity, the initial AE should be given an outcome date and a new AE must be reported with a different onset date than the end date of the previous AE to reflect the change in severity. The dates on the AEs cannot overlap. For all reported SAEs that increase in severity, the SAE supplemental eCRFs also need to be updated to reflect any changes due to the increase in severity. Grade refers to the severity of the AE. For AEs not captured by the NCI CTCAE, the following criteria should be used:

**Grade 1**	Mild; asymptomatic or mild symptoms; clinical or diagnostic observations only; intervention not indicated.
**Grade 2**	Moderate; minimal, local or noninvasive intervention indicated; limiting age-appropriate instrumental activities of daily living (ADL); instrumental ADL refer to preparing meals, shopping for groceries or clothes, using the telephone, managing money, etc.
**Grade 3**	Severe or medically significant but not immediately life-threatening; hospitalization or prolongation of hospitalization indicated; disabling; limiting self-care ADL; self-care ADL refer to bathing, dressing and undressing, feeding self, using the toilet, taking medications, and not bedridden.
**Grade 4**	Life-threatening consequences; urgent intervention indicated.
**Grade 5**	Death related to AE.

Assessment of Causality

The investigator will use the following definitions to assess the relationship of the AE to the use of **study drugs**:

**Reasonable Possibility**	After consideration of factors including timing of the event, biologic plausibility, clinical judgment, and potential alternative causes, there is sufficient evidence (information) to suggest a causal relationship.
**No Reasonable Possibility**	After consideration of factors including timing of the event, biologic plausibility, clinical judgment, and potential alternative causes, there is insufficient evidence (information) to suggest a causal relationship.

The investigator will also consult the Investigator's Brochure and/or Product Information, for marketed products, in their assessment.

For each AE/SAE, the investigator **must** document in the medical notes that he/she has reviewed the AE/SAE and has provided an assessment of causality.

There may be situations in which an SAE has occurred, and the investigator has minimal information to include in the initial report in the electronic data collection tool. However, **it is very important that the investigator always assess the causality for every event before the initial transmission of the SAE data to the EDC system.**

The investigator may change their opinion of causality in light of follow-up information and send an SAE follow-up report with the updated causality assessment.

The causality assessment is one of the criteria used when determining regulatory reporting requirements.

Follow-up of AE and SAE

The investigator is obligated to perform or arrange for the conduct of supplemental measurements and/or evaluations as medically indicated or as requested by Sponsor to elucidate the nature and/or causality of the AE or SAE as fully as possible. This may include additional laboratory tests or investigations, histopathological examinations, or consultation with other health care professionals.

New or updated information will be recorded in the originally completed form.

The investigator will submit any updated SAE data to the Sponsor within 24 hours of receipt of the information.

10.3.4 Reporting Adverse Events and Intercurrent Illnesses

The investigator must report any SAEs to the AbbVie Clinical Pharmacovigilance within 24 hours of becoming aware of the event.

The primary mechanism for reporting an SAE will be the EDC system.

The site will enter the SAE data into the EDC system as soon as it becomes available. SAEs that occur prior to the site having access to the EDC system, or if the EDC system is not operable, should be documented on the SAE non-CRF forms and emailed (preferred route) or faxed to AbbVie Clinical Pharmacovigilance within 24 hours of the site being made aware of the SAE. Following the SAE paper report, the site will enter the SAE data into the EDC system within 24 hours of EDC availability.

Email: PPDINDPharmacovigilance@abbvie.com<br>FAX to: +1-847-806-2062   Backup: +1-847-935-2844

After the study is completed at a given site, the EDC system will be taken offline to prevent the entry of new data or changes to existing data. If a site receives a report of a new SAE from a study subject or receives updated data on a previously reported SAE after the EDC system has been taken offline, then the site can report this information on the SAE non-CRF form (as above).

For safety questions, contact the Oncology Safety Team at
SafetyManagement_Oncology@abbvie.com

For any subject safety concerns, please contact the physician listed below:

[redacted]

[redacted] MD
Parexel International
Decentralized, USA
Mobile: [redacted]
Email: [redacted]

[redacted]

EMERGENCY MEDICAL CONTACT:
[redacted] MD
AbbVie Inc.
Mobile: [redacted]
Email: [redacted]

In emergency situations involving study subjects when the Parexel Medical Monitor or AbbVie Therapeutic Area Medical Director is not available by phone, please contact the 24-hour Parexel Medical Escalation Hotline where your call will be re-directed to a designated back-up Parexel Emergency Medical Contact:

HOTLINE: +1(978)805-7613 (United States)
+44 115 855 8406 (United Kingdom)

COVID-19 Pandemic-Related Acceptable Protocol Modifications

Supplemental study case report forms should be completed in the event of COVID-19-related missed/virtual visits, study drug interruptions or discontinuations, vaccines, or AEs (including capture of specific signs/symptoms of infection and testing results).

SARS-CoV-2 infections should be captured as AEs. If the event meets the criteria for an SAE, then follow the SAE reporting directions per the protocol and above. The following COVID-19-related supplemental eCRFs should be completed (for both serious and nonserious events):

- COVID-19 Supplemental Signs/Symptoms
- COVID-19 Status Form

If a subject has a confirmed or suspected SARS-CoV-2 infection and study drug was interrupted, the investigator should contact the CRO's emergency medical monitor contact listed above before reintroducing study drug.

Reactions known to be associated with the SARS-CoV-2 vaccine should be reported as AEs. If the event meets the criteria for an SAE, then follow the SAE reporting directions. All AEs associated with the SARS-CoV-2 vaccine will be linked to the vaccine on the COVID-19 Vaccine eCRF.

10.4 Appendix 4: Contraceptive and Barrier Guidance

Contraception Requirements for Females

Female subjects must follow the following contraceptive guidelines as specified:

Females of Non-Childbearing Potential

Females do not need to use birth control during or following study drug treatment if considered of non-childbearing potential due to meeting any of the following criteria:

1. Premenopausal female with permanent sterility or permanent infertility due to one of the following:
   - Permanent sterility due to a hysterectomy, bilateral salpingectomy, bilateral oophorectomy.
   - Non-surgical permanent infertility due to Mullerian agenesis, androgen insensitivity, or gonadal dysgenesis; investigator discretion should be applied to determining study entry for these individuals.
2. Postmenopausal female
   - Age > 55 years with no menses for 12 or more months without an alternative medical cause.
   - Age ≤ 55 years with no menses for 12 or more months without an alternative medical cause AND a follicle-stimulating hormone level > 30 IU/L.

Females of Childbearing Potential:

- Females of childbearing potential should be provided information from study staff about cryopreservation of eggs prior to treatment with study drug.
- Females of childbearing potential should not donate eggs during the study or for approximately 230 days after the last dose of study drug.
- Review and document pregnancy avoidance recommendations with females of childbearing potential.
- Females of childbearing potential must avoid pregnancy while taking study drug(s) and for at least 230 days after the last dose of study drug.
- Females must commit to one of the following methods of birth control (based on availability per local regulations) on Cycle 1 Day 1 (or earlier):
  - Combined (estrogen and progestogen containing) hormonal contraception (oral, intravaginal, transdermal, injectable) associated with inhibition of ovulation initiated at least 30 days prior to study baseline Day 1. For EU and US –the injectable method (combined injectable contraception) is not allowed.

- Combined (estrogen [ultra-low] and progestogen-containing) hormonal contraception (oral, intravaginal, transdermal, injectable) associated with inhibition of ovulation initiated at least 30 days prior to study baseline Day 1.
- Progestogen-only hormonal birth control (oral, injectable, implantable) associated with inhibition of ovulation initiated at least 30 days prior to study baseline Day 1.
- Bilateral tubal occlusion/ligation (can be via hysteroscopy, provided a hysterosalpingogram confirms success of the procedure).
- Intrauterine device (IUD).
- Intrauterine hormone-releasing system.
- Vasectomized partner (provided the partner has received medical confirmation of the surgical success of the vasectomy and is the sole sexual partner of the trial subject).
- Practice true abstinence, defined as: Refraining from heterosexual intercourse when this is in line with the preferred and usual lifestyle of the subject. Periodic abstinence (e.g., calendar, ovulation, symptothermal, post-ovulation methods) and withdrawal are not acceptable.

Contraception Requirements for Males

Male subjects should be provided information to seek advice about cryopreservation of sperm prior to treatment with study drug.

Male subjects who are sexually active with a female partner of childbearing potential, must agree to use male condoms, even if the male subject has undergone a successful vasectomy, from Day 1 through at least 140 days after the last dose of study drug. His female partner(s) must also use at least 1 of the contraceptive methods defined above for Females of Childbearing Potential.

Male subjects should not donate sperm during the study or for approximately 140 days after the last dose of study drug.

Contraception Requirements for Part 3b Carboplatin or Cisplatin Combination

Contraception recommendations related to use of carboplatin or cisplatin in Part 3b or concomitant therapies prescribed should be based on the local label and contraception period should be based on whichever is longer considering all administered therapies. In Korea, for subjects treated with the cisplatin or carboplatin combination, female subjects of childbearing potential must practice an effective method of birth control during the treatment period of cisplatin or carboplatin and 14 months after the last dose of cisplatin or carboplatin; male subjects must practice a highly effective method of birth control during the treatment period of cisplatin or carboplatin and 11 months after the last dose of cisplatin or carboplatin.

10.5 Appendix 5: Modification of Diet in Renal Disease

Modification of Diet in Renal Disease (MDRD) equation for adults.

For serum creatinine SI Units (µmol/L) (calculator available at https://www.niddk.nih.gov/health-information/professionals/clinical-tools-patient-management/kidney-disease/laboratory-evaluation/glomerular-filtration-rate-calculators/mdrd-adults-si-units):

Glomerular filtration rate (GFR) in mL/min/1.73 m^2 = 175 × $(SerumCr/88.4)^{-1.154}$ × $age^{-0.203}$ × 1.212 (if Black) × 0.742 (if female)

For serum creatinine Conventional Units (mg/dL) (calculator available at https://www.niddk.nih.gov/health-information/professionals/clinical-tools-patient-management/kidney-disease/laboratory-evaluation/glomerular-filtration-rate-calculators/mdrd-adults-conventional-units):

GFR in mL/min/1.73 m^2 = 175 × $SerumCr^{-1.154}$ × $age^{-0.203}$ × 1.212 (if patient is black) × 0.742 (if female)

To calculate CrCl (mL/min) from the MDRD GFR (mL/min/1.73 m^2) value, multiply the GFR value by the individual's body surface area (BSA) calculated using the below formula and divide by 1.73.

BSA (m^2) = $\sqrt{\left(\frac{Height\ (cm) \times Weight\ (kg)}{3600}\right)}$

10.6 Appendix 6: Strong CYP3A Inhibitor and Inducer List

Prohibited/Cautionary

Strong CYP3A inhibitors	Boceprevir, ceritinib, clarithromycin, cobicistat, conivaptan, danoprevir and ritonavir, elvitegravir and ritonavir, grapefruit juice, idelaisib, indinavir, indinavir and ritonavir, itraconazole, josamycin, ketoconazole, lonafarnib, lopinavir and ritonavir, mibefradil, mifepristone, nefazodone, nelfinavir, paritaprevir and ritonavir and (ombitasvir and/or dasabuvir), posaconazole, ribociclib, ritonavir, saquinavir and ritonavir, telaprevir, tipranavir and ritonavir, telithromycin, troleandomycin, tucatinib, and voriconazole
Strong CYP3A inducers	Apalutamide, avasimibe, carbamazepine, enzalutamide, ivosidenib, lumacaftor, mitotane phenytoin, rifampin, rifapentine, and St. John's wort

CYP3A = cytochrome P450 3A enzyme

Note: This is not an exhaustive list. For an updated list of strong CYP3A inhibitors and inducers, see the following link: http://www fda.gov/Drugs/DevelopmentApprovalProcess/DevelopmentResources/DrugInteractionsLabeling/ucm093664 htm or the University of Washington Drug Interaction Database: https://www.druginteractionsolutions.org. For all concomitant medications listed above, or concomitant medications not listed above, refer to the local label for additional information. If unsure of potential interaction with ABBV-706, contact the CRO's medical monitor or the Sponsor's Therapeutic Medical Director.

10.7 Appendix 7: Guidance Regarding Drugs Known to Prolong QT Interval

The medications listed below are known to impact or have possible risk to alter QT interval and are either prohibited or should be avoided, respectively, during Cycle 1 and Cycle 3 visits on the day before and days of triplicate ECG collections. This is applicable only for subjects with triplicate ECG measurements. In the case of clinical necessity, please discuss acceptable medication alternatives and dosing regimens with the CRO's medical monitor or the Sponsor's Therapeutic Medical Director.

**Prohibited during Cycle 1 and Cycle 3 visits:**
**Medications that are known to alter the QT interval:** Aclarubicin, Amiodarone, Anagrelide, Arsenic trioxide, Astemizole, Azithromycin, Bepridil, Chloroquine, Chlorpromazine, Cilostazol, Ciprofloxacin, Cisapride, Citalopram, Clarithromycin, Cocaine, Disopyramide, Dofetilide, Domperidone, Donepezil, Dronedarone, Droperidol, Erythromycin, Escitalopram, Flecainide, Fluconazole, Gatifloxacin, Grepafloxacin, Halofantrine, Haloperidol, Ibogaine, Ibutilide, Isavuconazole, Levofloxacin, Levomepromazine (methotrimeprazine), Levomethadyl acetate, Levosulpiride, Methadone, Moxifloxacin, Ondansetron, Oxaliplatin, Papaverine HCL (Intracoronary), Pentamidine, Pimozide, Probuco, Procainamide, Propofol, Quinidine, Roxithromycin, Sevoflurane, Sotalol, Sparfloxacin, Sulpiride, Sultopride, Terfenadine, Terlipressin, Terodiline, Thioridazine, Vandetanib
**Avoid during Cycle 1 and Cycle 3 visits**
**Medications that have a possible risk to alter the QT interval:** Alfuzosin, Apomorphine, Aripiprazole, Artenimol+piperaquine, Asenapine, Atomoxetine, Bedaquiline, Bendamustine, Benperidol, Betrixaban, Bortezomib, Bosutinib, Buprenorphine, Cabozantinib, Capecitabine, Ceritinib, Clofazimine, Clomipramine, Clozapine, Crizotinib, Cyamemazine, Dabrafenib, Dasatinib, Degarelix, Delamanid, Desipramine, Deutetrabenazine, Dexmedetomidine, Dolasetron, Efavirenz, Eliglustat, Epirubicin, Eribulin mesylate, Ezogabine, Felbamate, Fingolimod, Fluorouracil, Flupentixol, Gemifloxacin, Granisetron, Hydrocodone, Iloperidone, Imipramine, Inotuzumab ozogamicin, Isradipine, Ketanserin, Lapatinib, Lenvatinib, Leuprolide, Lithium, Io-Lopinavir and ritonavir, Maprotiline, Melperone, Memantine, Midostaurin, Mifepristone, Mirabegron, Mirtazapine, Moexipril/HCTZ, Necitumumab, Nicardipine, Nilotinib, Norfloxacin, Nortriptyline, Nusinersen, Ofloxacin, Osimertinib, Oxytocin, Paliperidone, Palonosetron, Panobinostat, Pasireotide, Pazopanib, Perflutren lipid microspheres, Perphenazine, Pilsicainide, Pimavanserin, Pipamperone, Primaquine phosphate, Promethazine, Prothipendyl, Ribociclib, Rilpivirine, Risperidone, Romidepsin, Saquinavir, Sertindole, Sorafenib, Sunitinib, Tacrolimus, Tamoxifen, Telavancin, Telithromycin, Tetrabenazine, Tiapride, Tipiracil and Trifluridine, Tizanidine, Tolterodine, Toremifene, Tramadol, Trimipramine, Tropisetron, Valbenazine, Vardenafil, Vemurafenib, Venlafaxine, Vorinostat, Zotepine
**Medications that have a conditional risk to alter the QT interval:** Amantadine, Amisulpride, Amitriptyline, Amphotericin B, Amsacrine, Atazanavir, Bendroflumethiazide, Chloral hydrate, Cimetidine, Diphenhydramine, Doxepin, Esomeprazole, Famotidine, Fluoxetine, Fluvoxamine, Furosemide, Galantamine, Garenoxacin, Hydrochlorothiazide, Hydroxychloroquine, Hydroxyzine, Indapamide, Itraconazole, Ivabradine, Ketoconazole, Lansoprazole, Loperamide, Metoclopramide, Metolazone, Metronidazole, Nelfinavir, Olanzapine, Omeprazole, Pantoprazole, Paroxetine, Piperacillin/Tazobactam, Posaconazole, Propafenone, Quetiapine, Quinine sulfate, Ranolazine, Sertraline, Solifenacin, Telaprevir, Trazodone, Voriconazole, Ziprasidone

10.8 Appendix 8: Part 3a Budigalimab Combination Immune-Related Adverse Event Management Guidance

- The guidelines in this appendix were developed from the American Society of Clinical Oncology (ASCO) irAE management guidelines.[22] These guidelines are general recommendations for the clinical situations listed below. Investigators should also consult their local practices as well as European Society for Medical Oncology (ESMO),[23] National Comprehensive Cancer Network (NCCN),[24] and Society for Immunotherapy of Cancer (SITC)[25] published guidelines for management of immune-related toxicity (including events not listed in this appendix) following treatment with immune checkpoint inhibitors.
- Investigators should also review the guidance in Section 6.7.7 in response to treatment-emergent AEs.
- Additional details and guidance regarding toxicities that are not described in this appendix (e.g., musculoskeletal toxicity, hematologic toxicity, cardiovascular toxicity, and ocular toxicity) may be found in the ASCO, ESMO, NCCN and SITC guidelines for diagnostic work-up and management of irAEs following treatment with immune checkpoint inhibitors.

10.8.1 General Guidance Regarding Management of Immune-Related Adverse Events Following Budigalimab (ABBV-181) Treatment

General guidance regarding evaluating and managing treatment-emergent irAEs following budigalimab (ABBV-181) treatment is shown in Table 10–2. Additionally, investigators should consult the CRO's medical monitor or the Sponsor's Therapeutic Medical Director. In general, corticosteroids should be initiated promptly for suspected irAEs and continued until an alternate etiology for a toxicity is determined or per the guidelines below. If an alternative nonimmune-related cause is identified, it should be treated accordingly and budigalimab treatment can be re-started/continued as clinically appropriate following discussion with the CRO's medical monitor or the Sponsor's Therapeutic Medical Director.

Table 10–2 Management of Immune-Related Adverse Events Following Budigalimab (ABBV-181) Treatment

Grade of Toxicity[a]	Management	Follow-Up
Grade 1<br>Mild; asymptomatic or mild symptoms; clinical or diagnostic observations only; intervention not indicated	[redacted]	
Grade 2<br>Moderate; minimal, local or noninvasive intervention indicated; limiting age-appropriate instrumental ADL[b]		
Grade 3-4<br>Grade 3: Severe or medically significant but not immediately life-threatening; hospitalization or prolongation of hospitalization indicated; disabling; limiting self-care ADL[c]<br>Grade 4: Life-threatening consequences; urgent intervention indicated		

ADL = activities of daily living; CRO = contract research organization; IV = intravenous

Subjects on IV steroids may be switched to an equivalent dose of oral corticosteroids (e.g., prednisone) once sustained clinical improvement is observed. Lower bioavailability of oral corticosteroids should be taken into account when switching to equivalent doses of oral corticosteroids.

a. Adapted from the NCI CTCAE v5.0 general guideline regarding grading of AEs.

b. Instrumental ADL refer to preparing meals, shopping for groceries or clothes, using the telephone, managing money, etc.

c. Self-care ADL refer to bathing, dressing and undressing, feeding self, using the toilet, taking medications, and not bedridden.

In addition, budigalimab should be permanently discontinued for ≥ Grade 2 myocarditis and ≥ Grade 3 ocular toxicity.

10.8.2 Skin Adverse Event Management Algorithm

The guidance in this section and in Table 10–3 does not address all potential skin toxicities that may occur following treatment with an immune checkpoint inhibitor. Investigators should

review the section on skin toxicities in the ASCO, ESMO, NCCN, and SITC guidelines for diagnostic evaluation and management of rash and diagnostic evaluation and management of other immune-related skin toxicities.

Rule out alternative nonimmune-related causes. Corticosteroids should be initiated promptly per guidelines below for suspected irAEs and continued unless an alternate etiology for a toxicity is determined. If a nonimmune-related cause is identified, treat accordingly per institutional guidelines and continue budigalimab (ABBV-181) therapy as clinically appropriate.

Diagnostic work-up including the following:

- Pertinent history and physical examination.
- Rule out any other etiology of the skin problem, such as an infection, an effect of another drug, or a skin condition linked to another systemic disease or unrelated primary skin disorder.
- If clinically indicated; laboratory hematology, liver and kidney tests, directed serologic studies if an autoimmune condition is suspected, such as lupus or dermatomyositis: a screening antinuclear antibody test, SS-A/Anti-Ro, SS-B/Anti-La if predominantly photo-distributed/photosensitivity, anti-histone, double-stranded DNA, and other relevant serologies. Consider expanding serologic studies or diagnostic work-up if other autoimmune conditions are considered based on signs and symptoms.
- Skin biopsy.
- Consider clinical monitoring with use of serial clinical photography.
- Review full list of subject medications to rule out other drug-induced cause for photosensitivity.

Table 10–3 Skin Adverse Event Management Algorithm

Grade of Rash[a]	Management	Follow-Up
Grade 1<br>Symptoms do not affect quality of life or are controlled with topical regimen and/or oral antipruritic	[redacted]	

Grade of Rash[a]	Management	Follow-Up
Grade 2<br>Inflammatory reaction that affects quality of life and requires intervention based on diagnosis	[redacted]	
Grade 3<br>As Grade 2 but with failure to respond to indicated interventions for a Grade 2 dermatitis		
Grade 4<br>All severe rashes unmanageable with prior interventions and intolerable		

AE = adverse event; CRO = contract research organization; IV = intravenous

Subjects on IV steroids may be switched to an equivalent dose of oral corticosteroids (e,g,, prednisone) once sustained clinical improvement is observed. Lower bioavailability of oral corticosteroids should be taken into account when switching to equivalent doses of oral corticosteroids.

a. Grading according to CTCAE is a challenge for skin toxicities. Instead, severity may be based on body surface area, tolerability, morbidity, and duration. Refer to NCI CTCAE v5.0 for grading of specific skin toxicities other than rash.

10.8.3 Cutaneous Adverse Reactions Management Algorithm

The guidance in this section and in Table 10–4 are provided to manage potential severe cutaneous adverse reactions (CARs), including Stevens-Johnson syndrome (SJS), toxic epidermal necrolysis (TEN), acute generalized exanthematous pustulosis, drug reaction with

eosinophilia and systemic symptoms (DRESS) and drug-induced hypersensitivity syndrome (DIHS). Investigators should review the section on skin toxicities in the ASCO, ESMO, NCCN, and SITC guidelines for additional details on diagnostic evaluation and management of skin toxicities following treatment with immune checkpoint inhibitors.

Diagnostic work-up should include the following:

- Total body skin examination with attention to examining all mucous membranes as well as complete review of systems
- Rule out any other etiology of the skin problem, such as an infection, an effect of another drug, or a skin condition linked to another systemic disease
- A biologic checkup, including a complete blood count (CBC) with differential test, and liver and kidney function tests, including urinalysis, in addition to the blood work; if the patient is febrile, blood cultures should be considered as well
- Skin biopsies to assess for full-thickness epidermal necrosis, as is seen in SJS/TEN, as well as other possible etiologies like paraneoplastic pemphigus or other autoimmune blistering dermatoses or other drug reactions, such as acute generalized exanthematous pustulosis
- Consider following subject's clinical course closely, using serial clinical photography
- If mucous membrane involvement or blistering on the skin is observed, consider early admission to a burn center for further monitoring and management
- NOTE: In cases of suspected SJS, TEN or any mucous membrane involvement, permanently discontinue budigalimab and monitor closely for improvement, regardless of grade.

Monitoring complicated CARs:

- Review of systems: Skin pain (like a sunburn), fevers, malaise, myalgias, arthralgias, abdominal pain, ocular discomfort or photophobia, sores or discomfort in the nares, sores or discomfort in the oropharynx, odynophagia, hoarseness, dysuria, sores or discomfort in the vaginal area for women or involving the meatus of the penis for men, sores in the perianal area, or pain with bowel movements
- Physical examination:
- Vital signs and a full skin examination specifically evaluating all skin surfaces and mucous membranes (eyes, nares, oropharynx, genitals, and perianal area).

- Assess for lymphadenopathy, facial or distal extremity swelling (may be signs of DIHS/DRESS).
- Assess for pustules or blisters or erosions in addition to areas of "dusky erythema," which may feel painful to palpation.
- To assess for a positive Nikolsky sign, place a gloved finger tangentially over erythematous skin and apply friction parallel to the skin surface.
- Nikolsky sign is positive if this results in detached or sloughing epidermis demonstrating poor attachment of the epidermis to the dermis, which is the case in some autoimmune disorders (e.g., pemphigus) and SJS/TEN.

Table 10–4 Cutaneous Adverse Reactions Management Algorithm

Grade of Severe Skin Change (adapted from Brahmer et al.)[20]	Management	Follow-Up
All Grades	[redacted]	
Grade 1		
Grade 2<br>Morbilliform ("maculopapular") exanthem covering 10% to 30% BSA with systemic symptoms, lymphadenopathy, or facial swelling		
Grade 3<br>Skin sloughing covering < 10% BSA with mucosal involvement associated signs (e.g., erythema, purpura, epidermal detachment, mucous membrane detachment)		

Grade of Severe Skin Change (adapted from Brahmer et al.)[20]	Management	Follow-Up
Grade 4<br>Skin erythema and blistering/sloughing covering ≥ 10% BSA with associated signs (e.g., erythema, purpura, epidermal detachment, mucous membrane detachment) and/or systemic symptoms and concerning associated bloodwork abnormalities (e.g., liver function test elevations in the setting of DRESS/DIHS)	[redacted]	

BSA = body surface area; CAR = cutaneous adverse reaction; CRO = contract research organization; DIHS = drug-induced hypersensitivity syndrome; DRESS = drug reaction with eosinophilia and systemic symptom; irAE = immune-related adverse event; IV = intravenous; IVIG = intravenous immunoglobulin; SJS = Stevens-Johnson syndrome; TEN = toxic epidermal necrolysis

Additional Considerations:

- The usual prohibition of corticosteroids for SJS is not relevant here, as the underlying mechanism is a T-cell immuno-directed toxicity. Adequate suppression is necessary with corticosteroids or other agents and may be prolonged in cases of DRESS/DIHS.
- Subjects on IV steroids may be switched to an equivalent dose of oral corticosteroids (e.g., prednisone) once sustained clinical improvement is observed. Lower bioavailability of oral corticosteroids should be taken into account when switching to equivalent doses of oral corticosteroids.

10.8.4 Gastrointestinal Adverse Event Management Algorithm

The guidance in this section and in Table 10–5 are provided to manage potential gastrointestinal (GI) toxicities. Investigators should review the section on GI toxicities published guidelines for additional guidance regarding diagnostic evaluation of diarrhea and diagnostic evaluation and management of other GI toxicities.

Rule out alternative nonimmune-related causes. Corticosteroids should be initiated promptly per guidelines below for suspected irAEs and continued unless an alternate etiology for a toxicity is determined. If a nonimmune-related cause is identified, treat accordingly per institutional guidelines and continue budigalimab (ABBV-181) therapy as clinically appropriate. Opiates/narcotics may mask symptoms of perforation. Infliximab should not be used in cases of perforation or sepsis.

NOTE: Diagnostic evaluation of all Grade ≥ 2 diarrhea should include the following:

- Work-up of blood (CBC, comprehensive metabolic panel, TSH, ESR, CRP), stool (culture, Clostridium difficile, parasite, cytomegalovirus or other viral etiology, ova and parasite)
- Consider testing for lactoferrin (for subject stratification to determine who needs more urgent endoscopy) and calprotectin (to follow-up on disease activity)
- Screening laboratories (HIV, hepatitis A and B, and blood quantiferon for tuberculosis) to prepare subjects to start infliximab should be routinely done in patients at high risk for those infections and appropriately selected patients based on infectious disease expert's evaluation
- Imaging (e.g., CT scan of abdomen and pelvis and GI endoscopy with biopsy) should be considered as there is evidence showing that the presence of ulceration in the colon can predict a corticosteroid refractory course, which may require early infliximab
- Consider repeating endoscopy for subjects who do not respond to immunosuppressive agents; repeating endoscopy for disease monitoring can be considered when clinically indicated and when planning to resume therapy

For Grade 3 and 4 events:

- Consider repeating endoscopy for subjects who do not respond to immunosuppressive agents; repeating endoscopy for disease monitoring should only be considered when clinically indicated and when planning to resume budigalimab (ABBV-181).

Table 10–5 Gastrointestinal Adverse Event Management Algorithm

Grade of Diarrhea	Management	Follow-Up
Grade 1<br>Increase of < 4 stools per day over baseline; mild increase in ostomy output compared to baseline limiting ADL	[redacted]	
Grade 2<br>Increase of 4 to 6 stools per day over baseline; moderate increase in ostomy output compared to baseline		
Grade 3 Increase of ≥ 7 stools per day over baseline; hospitalization indicated; severe increase in ostomy output compared to baseline; limiting self-care ADL		

Grade of Diarrhea	Management	Follow-Up
	[redacted]	
Grade 4<br>Life-threatening consequences; urgent intervention indicated		

AE = adverse event; ADL = activities of daily living; CRO = contract research organization; GI = gastrointestinal; IV = intravenous; TNF = tumor necrosis factor

Subjects on IV steroids may be switched to an equivalent dose of oral corticosteroids (e.g., prednisone) once sustained clinical improvement is observed. Lower bioavailability of oral corticosteroids should be taken into account when switching to equivalent doses of oral corticosteroids.

10.8.5 Renal Adverse Event Management Algorithm

Investigators should review the section on renal toxicities in the ASCO, ESMO, NCCN and SITC guidelines for additional guidance regarding diagnostic evaluation of elevated creatinine and diagnostic evaluation and management of renal toxicities.

Please also review NCI CTCAE v5.0 criteria definition of acute kidney injury.

Please also review NCI CTCAE v.5.0 criteria definition and grading of "acute kidney injury" events versus the definition and grading of "creatinine increased" (the term "creatinine increased" was used to develop Table 10–6).

Rule out alternative nonimmune-related causes. Corticosteroids should be initiated promptly for suspected irAEs and continued until an alternate etiology for a toxicity is determined or per the guidelines below. If a nonimmune-related cause is identified, treat accordingly and continue budigalimab (ABBV-181) therapy as clinically appropriate.

Table 10–6 Renal Adverse Event Management Algorithm

Grade of Creatinine Elevation	Management	Follow-Up
Grade 1<br>Creatinine > ULN to 1.5 × ULN	[redacted]	
Grade 2<br>Creatinine > 1.5 to 3.0 × baseline; > 1.5 to 3.0 × ULN		
Grade 3<br>Creatinine > 3.0 × baseline;<br>> 3.0 to 6.0 × ULN		
Grade 4<br>Creatinine > 6.0 × ULN		

ULN = upper limit of normal

Subjects on IV steroids may be switched to an equivalent dose of oral corticosteroids (e.g., prednisone) once sustained clinical improvement is observed. Lower bioavailability of oral corticosteroids should be taken into account when switching to equivalent doses of oral corticosteroids.

10.8.6 Pulmonary Adverse Event Management Algorithm

The guidance in this section and in Table 10–7 are provided to manage potential pulmonary AEs. Investigators should review the section on pulmonary toxicities in the ASCO, ESMO, NCCN

and SITC guidelines for additional details on diagnostic evaluation of pneumonitis and diagnosis and management of other lung toxicity.

Rule out alternative nonimmune-related causes. Corticosteroids should be initiated promptly per guidelines below for suspected irAEs and continued unless an alternate etiology for a toxicity is determined. If a nonimmune-related cause is identified, treat accordingly per institutional guidelines and continue budigalimab (ABBV-181) therapy as clinically appropriate.

Diagnostic evaluation for any grade pneumonitis should include the following: chest X-ray, CT, pulse oximetry for all grades; if Grade ≥ 2, may include the following infectious work-up: nasal swab, sputum culture and sensitivity, blood culture and sensitivity, urine culture and sensitivity. Consider early consultation with pulmonologist.

Table 10–7 Pulmonary Adverse Event Management Algorithm

Grade of Pneumonitis	Management	Follow-Up
Grade 1<br>Asymptomatic; clinical or diagnostic observations only; intervention not indicated (confined to one lobe of the lung or 25% of lung parenchyma)	[redacted]	
Grade 2<br>Symptomatic; medical intervention indicated; limiting instrumental ADL (involves more than one lobe of the lung or 25%-50% of lung parenchyma)		
Grade 3-4<br>Grade 3: Severe symptoms; limiting self-care ADL; oxygen indicated (involves all lung lobes or > 50% of lung parenchyma)<br>Grade 4: Life-threatening respiratory compromise; urgent intervention indicated (e.g., tracheotomy or intubation)		

ADL = activities of daily living; BAL = bronchoalveolar lavage; CT = computed tomography; DLCO = diffusing capacity of lung for carbon monoxide; IV = intravenous; IVIG = intravenous immunoglobulin

Subjects on IV steroids may be switched to an equivalent dose of oral corticosteroids (e.g., prednisone) once sustained clinical improvement is observed. Lower bioavailability of oral corticosteroids should be taken into account when switching to equivalent doses of oral corticosteroids.

Additional considerations include the following: 1) gastrointestinal and pneumocystis prophylaxis with proton pump inhibitor and Bactrim may be offered to subjects on prolonged corticosteroid use (> 12 weeks) according to institutional guidelines, 2) consider calcium and vitamin D supplementation with prolonged corticosteroid use, 3) role of prophylactic fluconazole with prolonged corticosteroid use (> 12 weeks) remains unclear and investigator should proceed according to institutional guidelines, and 4) bronchoscopy + biopsy; if clinical picture is consistent with pneumonitis, no need for biopsy.

10.8.7 Primary Hypothyroidism Adverse Event Management Algorithm

The guidance in this section and in Table 10–8 are provided to manage primary hypothyroidism AEs. Investigators should review the section on endocrine toxicities in the ASCO, ESMO, NCCN and SITC guidelines for additional details on diagnostic evaluation and management of hypothyroidism and other immune-related endocrine AEs.

Rule out alternative nonimmune-related causes. Consider imaging as clinically indicated.

Table 10–8 Primary Hypothyroidism Adverse Event Management Algorithm

Grade of Hypothyroidism	Management	Follow-Up
Grade 1<br>Asymptomatic; clinical or diagnostic observations only; intervention not indicated (TSH < 10 mIU/L)	[redacted]	
Grade 2<br>Symptomatic; thyroid replacement indicated; limiting instrumental ADL (TSH persistently > 10 mIU/L)		

Grade of Hypothyroidism	Management	Follow-Up
Grade 3-4<br>Grade 3: Severe symptoms; limiting self-care ADL; hospitalization indicated<br>Grade 4: Life-threatening consequences;<br>urgent intervention indicated	[redacted]	

ADL = activities of daily living; AE = adverse event; CRO = contract research organization; IV = intravenous; TSH = thyroid stimulating hormone

Additional considerations include the following:

[redacted]

- Extreme elevations of TSH can be seen in the recovery phase of thyroiditis and can be watched in asymptomatic subjects to determine whether there is recovery to normal within 3-4 weeks
- Under guidance of endocrinology, consider tapering hormone replacement and retesting in subjects with a history of thyroiditis (initial thyrotoxic phase)
- Adrenal dysfunction, if present, must always be replaced before thyroid hormone therapy is initiated

10.8.8 Hyperthyroidism Adverse Event Management Algorithm

The guidance in this section and in Table 10–9 are provided to manage primary hyperthyroidism AEs. Investigators should review the section on endocrine toxicities in the ASCO, ESMO, NCCN and SITC guidelines for additional details on diagnostic evaluation and management of hyperthyroidism and other immune-related endocrine AEs.

Rule out alternative nonimmune-related causes. Consider imaging as clinically indicated.

Consider TSH receptor antibodies if there are clinical features and suspicion of Grave disease (e.g., ophthalmopathy).

Close monitoring of thyroid function every 2 to 3 weeks after diagnosis to catch transition to hypothyroidism in subjects with thyroiditis and hyperthyroidism.

Table 10–9 Hyperthyroidism Adverse Event Management Algorithm

Grade of Hyperthyroidism	Management	Follow-Up
Grade 1<br>Asymptomatic; clinical or diagnostic observations only; intervention not indicated	[redacted]	
Grade 2<br>Symptomatic; thyroid suppression therapy indicated; limiting instrumental ADL		
Grade 3-4<br>Grade 3: Severe symptoms; limiting self-care ADL; hospitalization indicated<br>Grade 4: Life-threatening consequences;<br>urgent intervention indicated		

ADL = activities of daily living; AE = adverse event; CRO = contract research organization; IV = intravenous; TSH = thyroid stimulating hormone

Additional considerations include the following:

- Thyroiditis is transient and resolves in a couple of weeks to primary hypothyroidism or normal. Hypothyroidism can be treated as above.
- Graves disease is generally persistent and is due to increased thyroid hormone production that can be treated with antithyroid medical therapy.
- Physical examination findings of ophthalmopathy or thyroid bruit are diagnostic of Graves and should prompt early endocrine referral.

10.8.9 Primary Adrenal Insufficiency Adverse Event Management Algorithm

The guidance in this section and in Table 10–10 are provided to manage primary adrenal insufficiency AEs. Investigators should review the section on endocrine toxicities in the ASCO, ESMO, NCCN and SITC guidelines for additional details on diagnostic evaluation and management of primary adrenal insufficiency and other immune-related endocrine AEs.

Rule out alternative nonimmune-related causes. Consider imaging as clinically indicated.

Diagnostic work-up if adrenal insufficiency is suspected: Adrenocorticotropic hormone (ACTH) (morning), cortisol level (morning), metabolic panel (sodium, potassium, bicarbonate/CO_2, glucose), consider ACTH stimulation test for indeterminate results.

If primary adrenal insufficiency (high ACTH, low cortisol) is found biochemically, evaluate for precipitating cause of crisis such as infection. Perform an adrenal CT for metastasis/hemorrhage.

Table 10–10 Primary Adrenal Insufficiency Adverse Event Management Algorithm

Grade of Adrenal Insufficiency	Management	Follow-Up
Grade 1<br>Asymptomatic; clinical or diagnostic observations only; intervention not indicated	[redacted]	
Grade 2<br>Moderate symptoms; medical intervention indicated		
Grade 3-4<br>Grade 3: Severe symptoms; hospitalization indicated<br>Grade 4: Life-threatening consequences;<br>urgent intervention indicated		

ACTH=adrenocorticotropic hormone; IV = intravenous

Additional considerations include the following:

- Primary and secondary adrenal insufficiency can be distinguished by the relationship between ACTH and cortisol. If the ACTH is low with low cortisol, then management is as per pituitary – hypophysitis guidance.
- Subjects on corticosteroids for management of other conditions will have low morning cortisol as a result of iatrogenic, secondary adrenal insufficiency. ACTH will also be low in these subjects. A diagnosis of adrenal insufficiency is challenging to make in these situations (see pituitary-hypophysitis guidance).
- Emergent therapy for a subject with suspected adrenal insufficiency is best done with dexamethasone as a stimulation test can still be performed. If the diagnosis is already confirmed, can use hydrocortisone 100 mg.
- All subjects need education on stress dosing and a medical alert bracelet for adrenal insufficiency to trigger stress-dose corticosteroids by emergency medical services.
- Endocrine consultation prior to surgery or any procedure for stress-dose planning.

10.8.10 Pituitary-Hypophysitis Adverse Event Management Algorithm

The guidance in this section and in Table 10–11 are provided to manage pituitary-hypophysitis AEs. Investigators should review the section on endocrine toxicities in the ASCO, ESMO, NCCN, and SITC guidelines for additional details on diagnostic evaluation and management of hypophysitis and other immune-related endocrine AEs.

Rule out alternative nonimmune-related causes.

Consider diagnosis with the following laboratory changes: Low ACTH with a low cortisol. Low or normal TSH with a low FT4. Hypernatremia and volume depletion with diabetes insipidus. Low testosterone or estradiol with low luteinizing hormone (LH) and follicle-stimulating hormone (FSH). Diagnostic testing: Evaluate ACTH, cortisol (morning), TSH, FT4, electrolytes. Consider evaluating LH, FSH, and testosterone levels in males or estrogen in premenopausal females with fatigue, loss of libido, and mood changes.

Consider MRI of the brain with or without contrast with pituitary/sellar cuts in subjects with multiple endocrine abnormalities ± new severe headaches or complaints of vision changes.

Table 10–11 Pituitary-Hypophysitis Adverse Event Management Algorithm

Grade of Hypophysitis	Management	Follow-Up
Grade 1<br>Asymptomatic or mild symptoms clinical or diagnostic observations only; intervention not indicated	[redacted]	
Grade 2<br>Moderate, minimal, local or noninvasive intervention indicated; limiting age-appropriate instrumental ADL		

Grade of Hypophysitis	Management	Follow-Up
Grade 3-4<br>Grade 3: Severe symptoms or medically significant but not immediately life-threatening; hospitalization or prolongation of existing hospitalization indicated limiting self-care; ADL<br>Grade 4: Life-threatening consequences; urgent intervention indicated	[redacted]	

ADL = activities of daily living; AE = adverse event; TSH = thyroid stimulating hormone

Additional considerations include the following:

- Be aware of the need to start corticosteroids first when planning hormone replacement therapy for multiple endocrine deficiencies
- All subjects need instruction on doubling doses for illness (stress dosing) and a medical alert bracelet for adrenal insufficiency to trigger stress-dose corticosteroids by emergency medical services
- Corticosteroid use can cause isolated central adrenal insufficiency
- Work-up cannot be done with a simple AM cortisol in a subject on corticosteroids for other conditions
- Laboratory confirmation of adrenal insufficiency should not be attempted until treatment with corticosteroids for other disease is ready to be discontinued
- For long-term exposure, consult endocrinology for recovery and weaning protocol using hydrocortisone

10.8.11 Diabetes Management Algorithm

The guidance in this section and in Table 10–12 are provided to manage pituitary-hypophysitis AEs. Investigators should review the section on endocrine toxicities in the ASCO, ESMO, NCCN and SITC guidelines for additional details on diagnostic evaluation and management of diabetes and other immune-related endocrine AEs.

Rule out alternative nonimmune-related causes. Consider imaging as clinically indicated.

Table 10–12 Diabetes Management Algorithm

Grade of Hyperglycemia	Management	Follow-Up
Grade 1<br>Fasting glucose value > ULN to 160 mg/dL; Fasting glucose value > ULN to 8.9 mmol/L	[redacted]	
Grade 2<br>Fasting glucose value > 160 to 250 mg/dL; Fasting glucose value > 8.9 to 13.9 mmol/L		
Grade 3-4<br>Grade 3: > 250 to 500 mg/dL; > 13.9 to 27.8 mmol/L; hospitalization indicated<br>Grade 4: > 500 mg/dL; > 27.8 mmol/L; life-threatening consequences		

ULN = upper limit of normal

Additional considerations include the following:

- Insulin therapy can be used as the default in any case with hyperglycemia.
- Long acting therapy alone is not usually sufficient for type 1 diabetes mellitus, where half of daily requirements are usually given in divided doses as prandial coverage and half as long acting.
- Insulin doses will be lower in type 1 diabetes mellitus because of preserved sensitivity (total daily requirement can be estimated at 0.3 to 0.4 units/kg/day).
- In type 2 diabetes, sliding-scale coverage with meals over a few days provides data to estimate a subject's daily requirements and can be used to more rapidly titrate basal needs.

10.8.12 Neurological Adverse Event Management Algorithm

The guidance in this section and in Table 10–13 describes management of peripheral motor or sensory neuropathy and does not address all potential neurologic AEs that may occur following treatment with an immune checkpoint inhibitor (i.e., myasthenia gravis, autonomic neuropathy, aseptic meningitis, encephalitis). Investigators should review the section on nervous system toxicities in the ASCO, ESMO, NCCN, and SITC guidelines for diagnostic evaluation and management of neuropathy and diagnostic evaluation and management of other immune-related neurologic toxicities.

Rule out alternative nonimmune-related causes. Corticosteroids should be initiated promptly for suspected immune-related AEs and continued until an alternate etiology for a toxicity is determined or per the guidelines below. If a nonimmune-related cause is identified, treat accordingly and continue budigalimab (ABBV-181) therapy as clinically appropriate.

Please note that per the ASCO guidelines on management of immune-related AEs following immune checkpoint inhibitor therapy, Grade 3 or 4 peripheral neuropathy should be managed per the guidance regarding treatment-emergent Guillain-Barre Syndrome and investigators should consult the ASCO guidelines for additional interventions. For any grade transverse myelitis and for ≥ Grade 2 Guillain-Barre Syndrome, study treatment should be discontinued.

Table 10–13 Neurological Management Algorithm

Grade of Peripheral Neuropathy[a]	Management	Follow-Up
Grade 1<br>Motor: Asymptomatic; clinical or diagnostic observations only<br>Sensory: Asymptomatic<br>(Note: Any cranial nerve abnormality should be managed as Grade 2)	[redacted]	
Grade 2<br>Motor and Sensory: Moderate symptoms; limiting instrumental ADL		

Grade of Peripheral Neuropathy[a]	Management	Follow-Up
Grade 3-4<br>Grade 3: Severe symptoms; limiting self-care ADL<br>Grade 4: Life-threatening consequences; urgent intervention indicated<br>Note: Grade 3-4 toxicity may be due to Guillain-Barre Syndrome and should be managed as such.	[redacted]	

ADL = activities of daily living; AE = adverse event; CRO = contract research organization; IV intravenous

Subjects on IV steroids may be switched to an equivalent dose of oral corticosteroids (e.g., prednisone) once sustained clinical improvement is observed. Lower bioavailability of oral corticosteroids should be taken into account when switching to equivalent doses of oral corticosteroids.

a. The NCI CTCAE v5.0 should be consulted for descriptions of other specific neurologic toxicities.

10.8.13 Hepatic Adverse Event Management Algorithm

The guidance in this section and in Table 10–14 are provided to manage suspected immune-related hepatic AEs. Investigators should review the section on GI /hepatic toxicities in the in the ASCO, ESMO, NCCN, and SITC guidelines for additional details on diagnostic evaluation of elevated liver tests and diagnostic evaluation and management of other immune-related GI/hepatic AEs.

Rule out alternative nonimmune-related causes. If a nonimmune-related cause is identified, treat accordingly per institutional guidelines, and continue budigalimab (ABBV-181) therapy as clinically appropriate and manage ABBV-706 dosing per Section 10.8.1 and Section 6.1. For suspected irAEs, corticosteroids should be initiated promptly per guidelines below and continued unless an alternate etiology for a toxicity is determined. Consider imaging to evaluate for obstruction.

Diagnostic work-up should include the following:

- Viral hepatitis, alcohol history, iron study, evaluation for thromboembolic event, liver ultrasound, cross-sectional imaging for potential liver metastasis from primary malignancy.
- If suspicion for primary autoimmune hepatitis is high, can consider antinuclear, anti-smooth muscle, or anti-neutrophil cytoplasmic antibodies. If a subject has elevated alkaline phosphatase alone, gamma glutamyl transferase should be tested.
- For isolated elevation of transaminases, consider checking creatine kinase for other etiologies.

Table 10–14 Suspected Immune-Related Hepatic Adverse Event Management Algorithm

Grade of Liver Test Elevation	Management	Follow-Up
Grade 1<br>ALT or AST > ULN - 3.0 × ULN if baseline was normal; 1.5 - 3.0 × baseline if baseline was abnormal and/or<br>Total bilirubin > ULN - 1.5 × ULN if baseline was normal; > 1.0 - 1.5 × baseline if baseline was abnormal (Note: the ASCO guidelines specify that Grade 1 events of immune checkpoint inhibitor related hepatitis are asymptomatic).	[redacted]	
Grade 2<br>ALT or AST > 3.0 - 5.0 × ULN if baseline was normal; > 3.0 - 5.0 × baseline if baseline was abnormal and/or<br>Total bilirubin > 1.5 - 3.0 × ULN if baseline was normal; > 1.5 to 3.0 × baseline if baseline was abnormal (Note: the ASCO guidelines specify that Grade 2 events of immune checkpoint inhibitor related hepatitis are asymptomatic).		
Grade 3<br>AST or ALT > 5.0 - 20.0 × ULN if baseline was normal; > 5.0 - 20.0 × baseline if baseline was abnormal and/or Total bilirubin > 3.0 - 10.0 × ULN if baseline was normal; > 3.0 - 10.0 × baseline if baseline was abnormal<br>(Note: the ASCO guidelines specify that Grade 3 events of immune checkpoint inhibitor related hepatitis are also characterized by symptomatic liver dysfunction, fibrosis by biopsy, compensated cirrhosis, or reactivation of chronic hepatitis).		

Grade of Liver Test Elevation	Management	Follow-Up
	[redacted]	
Grade 4<br>AST or ALT > 20.0 × ULN if baseline was normal; > 20.0 × baseline if baseline was abnormal and/or<br>Total bilirubin > 10.0 × ULN if baseline was normal; > 10.0 × baseline if baseline was abnormal<br>(Note: the ASCO guidelines specify that Grade 4 events of immune checkpoint inhibitor related hepatitis are also characterized by decompensated liver function: e.g., ascites, coagulopathy, encephalopathy, coma).		

AE = adverse event; ALT = alanine aminotransferase; ASCO = American Society of Clinical Oncology; AST = aspartate aminotransferase; LFT = liver function test; IV = intravenous; ULN = upper limit of normal

Subjects on IV steroids may be switched to an equivalent dose of oral corticosteroids (e.g., prednisone) once sustained clinical improvement is observed. Lower bioavailability of oral corticosteroids should be taken into account when switching to equivalent doses of oral corticosteroids.

A supplemental case report form should be completed by the investigator or designee for hepatic related AEs that result in discontinuation or interruption of study drug, meet the criteria for an SAE, or involve the following laboratory criteria: 1) ALT and/or AST > 8 × ULN, 2) ALT and/or AST > 8 x ULN in conjunction with a total bilirubin > 2 × ULN.

10.9 Appendix 9: RECIST 1.1

Response criteria will be assessed using RECIST (version 1.1).[16] Please see the RECIST v.1.1 guidelines[16] for detailed instructions regarding the classification of measurable and non-measurable disease, methods and guidelines for measurement, and documentation of "Target" and "Non-Target" lesions. Changes in the measurable lesions over the course of therapy must be evaluated using the criteria listed below.

If the subject discontinues treatment for symptomatic deterioration, every effort should be made to document objective progression even after discontinuation of treatment.

10.9.1 Evaluation of Target Lesions

Complete Response (CR): The disappearance of all target lesions. Any pathological lymph nodes (whether target or non-target) must have reduction in short axis to < 10 mm.

Partial Response (PR): At least a 30% decrease in the sum of diameters of target lesions, taking as reference the baseline sum diameters.

Progressive Disease (PD): At least a 20% increase in the sum of the diameters of target lesions, taking as reference the smallest sum of diameters recorded since the treatment started (baseline or after) or the appearance of one or more new lesions. In addition to the relative increase of 20%, the sum must also demonstrate an absolute increase of at least 5 mm.

Stable Disease (SD): Neither sufficient shrinkage to qualify for PR nor sufficient increase to qualify for PD, taking as reference the smallest sum of diameters since the treatment started (baseline or after).

10.9.2 Evaluation of Non-Target Lesions

Complete Response (CR): The disappearance of all non-target lesions and normalization of tumor marker level. All lymph nodes must be non-pathological in size (< 10 mm short axis).

Non-CR/Non-PD: Persistence of one or more non-target lesion(s) and/or maintenance of tumor marker level above the normal limits.

Progressive Disease (PD): Unequivocal progression of existing non-target lesions. In this setting, to achieve 'unequivocal progression' on the basis of non-target disease, there must be an overall level of substantial worsening in non-target disease such that, even in the presence of SD or PR in target disease, the overall tumor burden has increased sufficiently to merit

discontinuation of therapy. A modest 'increase' in the size of one or more non-target lesions is usually not sufficient to qualify for unequivocal progression status. The designation of overall progression solely on the basis of change in non-target disease in the face of SD or PR of target disease will therefore be extremely rare.

10.9.3 New Lesions

The appearance of new malignant lesions denotes disease progression. While there are no specific criteria for the identification of new radiographic lesions, the findings of a new lesion should be unequivocal, i.e., not attributable to differences in scanning technique, timing of scanning, phase of contrast administration, change in imaging modality or finding thought to represent something other than tumor (e.g., some 'new' bone lesions may be simply healing or flare of pre-existing lesions). A lesion identified on a follow-up study in an anatomical location that was not scanned at baseline is considered a new lesion and will indicate disease progression.

An example of this is the subject who has visceral disease at baseline and while on study has a CT or MRI brain ordered which reveals metastases. The subject's brain metastases are considered evidence of progressive disease even if he/she did not have brain imaging at baseline. If a new lesion is equivocal (i.e., too small to measure), continued therapy and follow-up evaluation will clarify if it represents truly new disease. If repeat scans confirm there is a new lesion, then progression should be declared using the date of the initial scan. In all cases, confirmation of response is not required.

10.10 Appendix 10: RANO Response Criteria for Subjects With Primary CNS Tumor

RANO Criteria for Overall Tumor Assessments

For subjects with GBM CNS primary tumors, tumor responses will be assessed using RANO criteria[17] with modifications from the 2017 RANO update[18]. Aspects of RANO response assessment are described below; please see guidelines[18] for detailed instructions on RANO radiographic response assessment.

Bidimensional and/or Volumetric Measurements

Either bidimensional or volumetric assessments of tumor size may be used, as long as they are consistently used within each subject. Two-dimensional, perpendicular measurements of contrast enhancing tumor size, excluding the resection cavity along with any cysts or areas of central macroscopic necrosis, should be used for response assessment if volumetric tools are not available. Table 10–15 outlines suggested volumetric conversions from 2- to 3-dimensional measurements for consistency in response definitions.

Table 10–15 Bidimensional to Volumetric Definitions of Radiographic Response and Progression

State of Disease	Change in Bidimensional Product	Estimated Volumetric Change
Complete response (CR)	100% Decrease	100% Decrease
Partial response (PR)	≥ 50% Decrease	≥ 65% Decrease
Progressive disease (PD)	≥ 25% Increase	≥ 40% Increase
Stable disease (SD)	< 50% Decrease to < 25% Increase	< 65% Decrease to < 40% Increase

Detailed Definitions Used for Radiographic Response Assessment Criteria

Radiographic response should be determined in comparison to the tumor measurements obtained at baseline (post-radiation scan will be baseline for newly diagnosed GBM, and pretreatment scans will be the baseline for recurrent GBM [*For the current study only subjects with recurrent CNS tumors are eligible for study entry*]) for determination of response, and the smallest tumor measurement at either pretreatment baseline or following initiation of therapy for determining progression.

Because novel treatments are likely to result in a higher than normal incidence of treatment-related increase in contrast enhancement ("pseudo-progression," PsP) or decrease in contrast enhancement ("pseudo-response," PsR), patients should continue therapy with close observation (e.g., 4- to 8-week intervals) if there is a suspicion of PsP or PsR. If subsequent imaging studies and/or clinical observations demonstrate that progression in fact has occurred, the date of confirmed progression should be noted as the scan at which the potential progression was first identified. Definitions for CR, PR, PD, and SD should be defined as follows for all target lesions.

Complete Response (CR)**:** Requires all of the following:

1. Disappearance of all enhancing measurable and non-measurable disease sustained for at least 4 weeks. The first scan exhibiting disappearance of all enhancing measurable and non-measurable disease is considered "preliminary CR." If the second scan exhibits measurable enhancing disease with respect to the "preliminary CR" scan, then the response is not sustained, noted as PsR, and is now considered "preliminary PD" (note confirmed PD requires at least 2 sequential increases in tumor volume). If the second scan continues to exhibit disappearance of enhancing disease or emergence of non-measurable disease (less than 10 mm bidimensional product), it is considered a durable CR and the patient should continue on therapy until confirmed PD is observed.
2. Patients must be off corticosteroids (or on physiologic replacement doses only).
3. Stable or improved clinical assessments (i.e., neurological examinations).

Note: Patients with non-measurable disease only at baseline cannot have CR; the best response possible is stable disease (SD). *(For the current study subjects without measurable disease at baseline are ineligible for study entry.)*

Partial Response (PR)**:** Requires all of the following:

1. ≥ 50% decrease in sum of products of perpendicular diameters or ≥ 65% decrease in total volume of all measurable enhancing lesions compared with baseline, sustained for at least 4 weeks. The first scan exhibiting ≥ 50% decrease in sum of products of perpendicular diameters or ≥ 65% decrease in total volume of all measurable enhancing lesions compared with baseline is considered "preliminary PR." If the second scan exhibits PD with respect to the "preliminary PR" scan, then the response is not sustained, noted as PsR, and is now considered "preliminary PD" (note confirmed PD requires at least 2 sequential increases in tumor volume). If the second scan exhibits SD, PR, or CR, it is considered a durable PR and the patient should continue on therapy until confirmed PD is observed.
2. Steroid dose should be the same or lower compared with baseline scan.
3. Stable or improved clinical assessments.

Note: Patients with non-measurable disease only at baseline cannot have PR; the best response possible is stable disease (SD). *(For the current study subjects without measurable disease at baseline are ineligible for study entry.)*

Progressive Disease (PD): Defined by any of the following:

1. At least 2 sequential scans separated by at ≥ 4 weeks both exhibiting ≥ 25% increase in sum of products of perpendicular diameters or ≥ 40% increase in total volume of enhancing lesions. The first scan exhibiting ≥ 25% increase in sum of products of perpendicular diameters or ≥ 40% increase in total volume of enhancing lesions should be compared to the smallest tumor measurement obtained either at baseline (if no decrease) or best response (on stable or increasing steroid dose) and is noted as "preliminary PD." If the second scan at least 4 weeks later exhibits a subsequent ≥ 25% increase in sum of products of perpendicular diameters or ≥ 40% increase in total volume of enhancing lesions relative to the "preliminary PD" scan, it is considered "confirmed PD" and the patient should discontinue therapy. If the second scan at least 4 weeks later exhibits SD or PR/CR, this scan showing "preliminary PD" is noted as PsP, and the patient should continue on therapy until a second increase in tumor size relative to the PsP scan is observed. Note that any new measurable (> 10 mm × 10 mm) enhancing lesions should not be immediately considered PD, but instead should be added to the sum of bidimensional products or total volume representing the entire enhancing tumor burden.
2. In the case where the baseline or best response demonstrates no measurable enhancing disease (visible or not visible), then any new measurable (> 10 mm × 10 mm) enhancing lesions are considered PD after confirmed by a subsequent scan ≥ 4 weeks exhibiting ≥ 25% increase in sum of products of perpendicular diameters or ≥ 40% increase in total volume of enhancing lesions relative to the scan first illustrating new measurable disease. The first scan exhibiting new measurable disease is noted as "preliminary PD." If the second scan at least 4 weeks later exhibits a subsequent ≥ 25% increase in sum of products of perpendicular diameters or ≥ 40% increase in total volume of enhancing lesions relative to the "preliminary PD" scan it is considered "confirmed PD" and the patient should discontinue therapy. If the second scan at least 4 weeks later exhibits SD, CR, PR, or becomes non-measurable, this scan showing "preliminary PD" is noted as PsP, and the patient should continue on therapy until a second increase in tumor size relative to the "preliminary PD," or PsP, scan is observed. Note that any new measurable (> 10 mm × 10 mm) enhancing lesions on the subsequent scan following the preliminary PD scan should not be immediately considered confirmed PD, but instead should be added to the sum of bidimensional products or total volume representing the entire enhancing tumor burden.
3. Clear clinical deterioration not attributable to other causes apart from tumor (e.g., seizures, medication adverse effects, therapy complications, stroke, infection) or attributable to changes in steroid dose.
4. Failure to return for evaluation as a result of death or deteriorating condition.

Stable Disease (SD): Requires all of the following:

1. Does not qualify for CR, PR, or PD as defined above. Note this also applies to patients that demonstrate PsR when the confirmation scan does not show PD or PsP when the confirmation scan does not show PR/CR.
2. In the event that corticosteroid dose was increased (for new symptoms/signs) without confirmation of disease progression on neuroimaging, and subsequent follow-up imaging shows that the steroid increase was required because of disease progression, the last scan considered to show stable disease will be the scan obtained when the corticosteroid dose was equivalent to the baseline dose.

10.11 Appendix 11: Country Specific Requirements

10.11.1 Japan-Specific Requirements

Study procedures specified under Section 10.11.1 will only be applied to sites in Japan.

Under Section 10.11.1.1, "drugs used in the clinical trial" is defined as the study drugs including the investigational drug.

10.11.1.1 Latest Scientific Findings on Drugs Used in the Clinical Trial

In this study, ABBV-706, budigalimab (ABBV-181), carboplatin, and cisplatin will be used as drugs used in the clinical trial. Table 10-16 shows the latest scientific findings on drugs used in the clinical trial for the reference to the safety evaluation by the investigators.

Table 10-16 Latest Scientific Findings on Drugs used in the Clinical Trial

Drugs used in the Clinical Trial (Generic Name)	Latest Scientific Findings
ABBV-706	Investigator Brochure
Budigalimab (ABBV-181)	Investigator Brochure
Carboplatin	Package Insert
Cisplatin	Package Insert

10.11.1.2 Handling of Drugs used in the Clinical Trial

When drugs used in the clinical trial will be provided by the sponsor, drugs used in the clinical trial will be managed in accordance with the latest scientific findings (Table 10-16) in addition to a manual of drugs used in the clinical trial that will be separately prepared by the sponsor.

When drugs used in the clinical trial will be provided by each site, drugs used in the clinical trial will be managed in accordance with the procedure of each site.

10.11.1.3 Reporting of Serious Adverse Event and Adverse Event of Special Interest

When reporting a SAE, the investigator will additionally evaluate the causal relationship with drugs used in the clinical trial.

10.11.1.4 Eligibility (Japan Only)

Regarding eligibility criterion #18, subjects in Japan must have no history of ILD.

Regarding eligibility criterion #25, for subjects in Japan, if there is a possibility of pregnancy as judged by a physician's interview, etc., the subject will be excluded from this study. Females who stop breastfeeding and will not breastfeed for 260 days after the last dose of ABBV-706 will be allowed to enroll.

Japan only: No history of hypersensitivity to the active ingredients or any excipients of ABBV-181.

10.11.1.5 Contraception Requirements (Japan Only)

Contraception recommendations are detailed in Section 10.4. However, the following methods of contraception are not approved or certified in Japan:

- Combined (estrogen and progestogen containing) hormonal birth control (intravaginal, transdermal, injectable)
- Progestogen-only hormonal birth control (injectable, implantable, oral)
- Female condom
- Cap
- Sponge with spermicide
- Bilateral tubal occlusion

Vasectomized partner (provided the partner has received medical confirmation of the surgical success of the vasectomy, and is the sole sexual partner of the study subject).

10.11.1.6 Safety Assessments (Japan Only)

In Part 1b (Japan only), safety evaluations on Cycle 1 Day 1 through Cycle 1 Day 8 will be performed under hospitalization. In principle, a one-week hospitalization is required, but if the subject or their family can properly monitor their condition at home and have access to the clinical trial sites when needed, the attending physician in charge of the clinical trial may decide to shorten the hospitalization period or choose outpatient treatment. Investigators can discharge subjects from the hospital on Cycle 1 Day 8 if the Investigator judges that the subject's safety is ensured as an outpatient considering clinical course during hospitalization, vital sign, laboratory data and findings of physical examination on Cycle 1 Day 8.

For Physical Examinations, for Japanese subjects only, SpO_2 testing and careful auscultation must be carried out on Day 1 of each cycle.

10.11.1.7 Clinical Laboratory Testing (Japan Only)

Table 10-17 details all clinical laboratory tests for sites in China.

Table 10-17 Protocol-Required Safety Laboratory Tests for Japan Only

**Hematology**	
White blood cell (WBC) count	Lymphocytes (absolute count)
Red blood cell (RBC) count	Monocytes (absolute count)
Hemoglobin	Eosinophils (absolute count)
Platelet count	Basophils (absolute count)
Neutrophils (absolute count)	Band neutrophils (absolute count)
**Coagulation**	
Prothrombin time (PT)	International Normalized Ratio (INR)
Activated partial thromboplastin time (aPTT)	
**Clinical Chemistry**	
Alanine aminotransferase (ALT / SGPT)	Creatinine clearance (MDRD calculation)
Albumin	Gamma glutamyl transferase (GGT)
Alkaline phosphatase (ALP)	Glucose
Aspartate aminotransferase (AST / SGOT)	Inorganic Phosphorus
Bicarbonate/carbon dioxide	Lactate dehydrogenase (LDH)[a]
Bilirubin: Direct, Indirect, Total	Magnesium
Blood urea nitrogen (BUN)	Potassium
Calcium	Sodium
Chloride	Total protein
Creatinine	KL-6[g]<br>Hemoglobin A1C (HbA1C)[a,h]
**Urinalysis**	
Bilirubin	Blood
Glucose	pH
Ketones	Protein
Leukocyte esterase (not required unless urinary tract infection suspected)	Urobilinogen<br>Specific gravity
Nitrite	Microscopic (reflex)
**Hormone Levels (Part 3a Budigalimab combination only)**	
Thyroid stimulating hormone (TSH)	Free thyroxine (T4)[e]
Triiodothyronine (T3)[e]	Cortisol (if indicated)
	Adrenocorticotropic hormone (ACTH) (if indicated)

**Pregnancy Testing**
Serum/urine human beta chorionic gonadotrophin[b,c,d]
Follicle-stimulating hormone[a,b,f]
**Virology Testing (Japan Only)**
Human immunodeficiency virus (HIV)[a]
Viral hepatitis serologies[a]<br>• Hepatitis B antigen (HbsAg)<br>• Hepatitis B antibody (HBsAb)<br>• Hepatitis B core antibody (HBcAb)<br>• Hepatitis C virus (HCV) antibody

SGOT = serum glutamic-oxaloacetic transaminase; SGPT = serum glutamic-pyruvate transaminase

a. Performed only at Screening.
b. Females only.
c. Pregnancy testing is not required for females of non-childbearing potential.
d. A serum pregnancy test may be used instead of a urine pregnancy test during the study. Serum pregnancy test should be performed at any time a pregnancy is suspected.
e. Free T3 and Total T4 may be performed per institutional practice.
f. If needed to determine postmenopausal status.
g. For Japanese subjects only at Screening and anytime when the Investigator detects abnormal findings which suggest early symptom of suspected ILD.
h. For Japanese subjects in Part 3a only.

Japan sites will perform a HIV screening test, and hepatitis screening tests. HIV antibody testing will be performed per local standard of practice. The results of the HIV antibody testing will be retained by the study site under confidential restriction.

In addition, for Japan subjects at screening only, if hepatitis B surface antigen (HBsAg) is negative, hepatitis B core antibody (HBcAb) and hepatitis B surface antibody (HBsAb) testing must be performed. If HBsAg is negative, and HBcAb or HBsAb is positive, and hepatitis B virus DNA value is < 20 IU/ml, the subject should be managed/monitored following the guidelines outlined in Figure 10-1.

Figure 10-1 Guidelines for the Prevention of Hepatitis B Virus Reactivation in Subjects Receiving Immunosuppressive Therapy or Chemotherapy

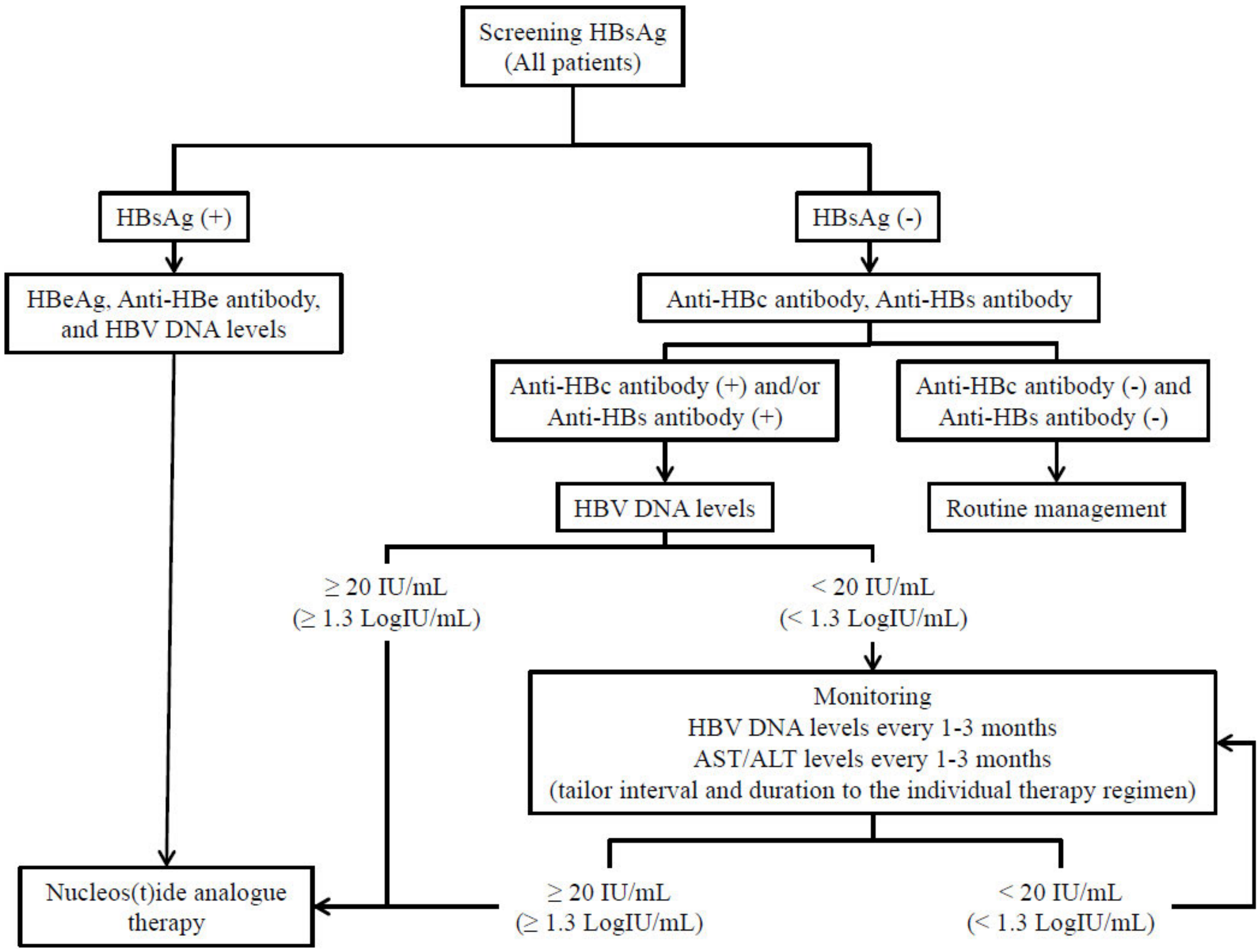

Ab = antibody; Ag = antigen; AST/ALT = aspartate aminotransferase/alanine aminotransferase; DNA = deoxyribonucleic acid; HBc = Hepatitis B core; HBe = Hepatitis B e; HBs = Hepatitis B surface; HBV = Hepatitis B virus

10.11.1.8 Biomarkers (Japan Only)

For Japan only: Biomarker research results other than SEZ6 will not be disclosed to the subjects because these results could not be leveraged for diagnostic and/or medical judgement purposes.

10.11.2 China-Specific Requirements

10.11.2.1 Eligibility (China Only)

Hepatitis serology is required at screening for subjects in China, per local standard of care.

- For China only, subjects must have documented virology status of hepatitis, as confirmed by screening HBV serology tests:
  - HBV serology: HBsAg, HBsAb, total HBcAb; if a patient has a positive HBsAg, HBsAb, and/or HBcAb test at screening, an HBV DNA test should be performed at screening. If HBV DNA is detected (≥ 20 IU/mL or above the limit of detection), subject must agree to initiate anti-HBV treatment (per local standard of care) a minimum of 14 days prior to first dose of study drug and be willing to continue treatment for the study duration and for at least 6 months after the last dose of study drug;
  - Subjects with active HBV infection will be allowed to enroll if they meet the following criteria: HBV DNA < 500 IU/mL obtained within 35 days prior to initiation of study treatment AND anti-HBV treatment (per local standard of care) for a minimum of 14 days prior to first dose and willingness to continue treatment for the study duration until at least 6 months after the last dose of study drug. Ongoing antiviral therapy that include strong CYP3A inhibitors should be used with caution and assessed for any potential for risk of adverse drug-drug interactions, at the discretion of the treating investigator;
  - Subjects with resolved HBV infection (HBsAg-negative, HBcAb-positive, and HBV DNA undetectable) are eligible if they are willing to comply with HBV DNA monitoring every cycle (Q3W) or per local guideline while on study drug and agree to initiate antiviral therapy if HBV DNA becomes detectable (≥ 20 IU/mL or above the limit of detection);
  - Subjects with a negative HBcAb, positive HBsAb, and HBV DNA undetectable at screening must agree to comply with HBV DNA monitoring every cycle (Q3W) or per local guideline if they have no prior history of receiving a complete hepatitis B vaccination series or where locally mandated.
- No active systemic or uncontrolled local bacterial, fungal, or viral infection requiring anti-microbial therapy within 2 weeks of C1D1 with the exception of antiviral therapy for an HBV infection (see above).

10.11.2.2 Clinical Laboratory Tests (China Only)

Table 10–18 details all clinical laboratory tests for sites in China.

In addition to Clinical Laboratory tests outlined in Section 8.4.5, sites enrolling subjects in mainland China will perform hepatitis tests (HBV and HCV) during Screening. Viral serology can be performed by sites locally and will only be sent for central lab testing per site requirement or if the corresponding detection cannot be performed by the site. HIV testing is not required for eligibility unless mandated by local regulatory authority or ethics committee/institutional review board.

For Clinical Chemistry:

- Blood urea nitrogen (BUN): For China sites, BUN or urea should be accepted for clinical laboratory testing.
- Inorganic phosphorus: For China sites, inorganic phosphorus or phosphorus should be acceptable for clinical laboratory testing.
- Chloride: For China sites, chloride or chlorine should be acceptable for clinical laboratory testing.
- HCV serology: HCV antibody and HCV RNA (if antibody test is positive); if a subject has a positive HCV antibody test, an HCV RNA test must also be performed to determine if the subject has an active HCV infection.

Table 10–18 Protocol-Required Safety Laboratory Tests for China Only

**Hematology**	
White blood cell (WBC) count	Lymphocytes (absolute count)
Red blood cell (RBC) count	Monocytes (absolute count)
Hemoglobin	Eosinophils (absolute count)
Platelet count	Basophils (absolute count)
Neutrophils (absolute count)	Band neutrophils (absolute count)[a]
**Coagulation**	
Prothrombin time (PT)	International Normalized Ratio (INR)
Activated partial thromboplastin time (aPTT)	
**Clinical Chemistry**	
Alanine aminotransferase (ALT / SGPT)	Creatinine clearance (MDRD calculation)
Albumin	Gamma glutamyl transferase (GGT)
Alkaline phosphatase (ALP)	Glucose
Aspartate aminotransferase (AST / SGOT)	Inorganic Phosphorus[b]
Bicarbonate/carbon dioxide	Lactate dehydrogenase (LDH)[c]
Bilirubin: Direct, Indirect, Total	Magnesium
Blood urea nitrogen (BUN)[d]	Potassium
Calcium	Sodium
Chloride[e]	Total protein
Creatinine	
**Urinalysis**	
Bilirubin	Blood
Glucose	pH
Ketones	Protein
Leukocyte esterase (not required unless urinary tract infection suspected)	Urobilinogen
	Specific gravity
Nitrite	Microscopic (reflex)

**Hormone Levels (Part 3a Budigalimab combination only)**	
Thyroid stimulating hormone (TSH)<br>Triiodothyronine (T3)[i]	Free thyroxine (T4)[i]<br>Cortisol (if indicated)<br>Adrenocorticotropic hormone (ACTH) (if indicated)
**Pregnancy Testing**	
Serum/urine human beta chorionic gonadotrophin[f,g,h]	
Follicle-stimulating hormone[c,f,j]	
**Virology Testing (China Only)**	
Human immunodeficiency virus (HIV)[c]	
Viral hepatitis serologies[c]<br>• Hepatitis B antigen (HbsAg)<br>• Hepatitis B antibody (HBsAb)<br>• Hepatitis B core antibody (HBcAb)<br>• Hepatitis C virus (HCV) antibody	

SGOT = serum glutamic-oxaloacetic transaminase; SGPT = serum glutamic-pyruvate transaminase

a. Testing for bands can be waived by China sites if testing is not available at site. Hematology detection should be performed per local standard to enable investigator to evaluate subject's eligibility to this study, as well as to support evaluation of hematological function per study/medical needs.
b. For China site, inorganic phosphorus or phosphorus are acceptable.
c. Performed only at Screening.
d. For China sites, BUN or urea are acceptable.
e. For China sites, chloride or chlorine are acceptable.
f. Females only.
g. Pregnancy testing is not required for females of non-childbearing potential.
h. A serum pregnancy test may be used instead of a urine pregnancy test during the study. Serum pregnancy test should be performed at any time a pregnancy is suspected.
i. Free T3 and Total T4 may be performed per institutional practice.
j. If needed to determine postmenopausal status

10.11.2.3 Biomarkers (China Sites Only)

Exploratory biomarker testing will be conducted according to local regulation. Evaluation of SEZ6 target expression by IHC and RNAseq of tumor tissues and potential predictive molecular signatures from RNAseq of tumor tissues and their association with PK, safety, and antitumor activity has been included in the exploratory endpoints. Plasma biomarker samples will not be collected for subjects in mainland China.

10.11.2.4 Subject Paper Diary Cards

For sites in China only as per local requirements, subject paper diary cards will be used to record adverse event symptoms and concomitant medications.

For sites in China, a paper diary card will be provided the Screening visit. Subjects will be trained on how to complete the diary cards by site staff during the Screening visit. If subjects require re-training during the study, the site staff will accommodate this requirement.

All subjects should complete their paper diary cards throughout the entire study. Subjects will be instructed to bring their paper diary cards back to the site to be reviewed and collected at each visit, including at any visit at which a dose level change may be required. If COVID-19 circumstances warrant a virtual visit, diary cards should be reviewed virtually with the subject and site should collect the paper diary card at the next on-site visit.

Subjects will also be instructed to record adverse events symptoms and concomitant medications in the paper diary cards. At each visit, the paper diary cards are to be reviewed by the investigator, assessed for any updates needed, and collected from the subject by study staff. Relevant information will be recorded in existing adverse event, drug administration, and concomitant medication forms in the eCRF as applicable. At each visit after the Screening visit, including the Final/Premature Discontinuation Visit, the paper diary cards are to be returned to the site and appropriately filed with the subject's source documents for this study. At each visit, after the paper diary card is initially dispensed, except the Final/Premature Discontinuation Visit, the subject will be provided a new diary card.

In case of missing diary card information, or when discrepancies are discovered, site personnel should discuss with the subject and document changes to data in site records and eCRF forms, if applicable. The need for completion of the paper diary card will be reinforced with the subject during study visits, as necessary, by the site personnel.

10.12 Appendix 12: Protocol Amendment History

The Protocol Amendment Summary of Changes Table for the current amendment is located directly before the table of contents.

Amendment 8 (02-Dec-2024)

Overall Rationale for the Amendment:

The purpose of this version is to modify the China only cohort (Part 2c) to expand dose escalation to two doses before expansion, and other minor editorial changes for consistency throughout the protocol.

Amendment 7 (18-Jun-2024)

This amendment is considered to be substantial based on the criteria set forth in Article 10(a) of Directive 2001/20/EC of the European Parliament and the Council of the European Union.

This modification is considered to be substantial based on the criteria set forth in European Regulation 536/2014 Article 2(2)(13).

Overall Rationale for the Amendment:

The purpose of this version is to add text for a China only cohort and other minor editorial changes for consistency throughout the protocol.

Amendment 6 (08-Jan-2024)

This amendment is considered to be substantial based on the criteria set forth in Article 10(a) of Directive 2001/20/EC of the European Parliament and the Council of the European Union.

This modification is considered to be substantial based on the criteria set forth in European Regulation 536/2014 Article 2(2)(13).

Overall Rationale for the Amendment:

The purpose of this version is to update the protocol to address items raised in an RFI from the EMA and to make other minor editorial changes for consistency throughout the protocol.

Amendment 5 (24-Oct-2023)

Overall Rationale for the Amendment:

The purpose of this version is to change the reference to the study EU CT Number.

Amendment 4 (25-Sept-2023)

This amendment is considered to be substantial based on the criteria set forth in Article 10(a) of Directive 2001/20/EC of the European Parliament and the Council of the European Union.

This modification is considered to be substantial based on the criteria set forth in European Regulation 536/2014 Article 2(2)(13).

Overall Rationale for the Amendment:

The purpose of this version is to add Japan-specific eligibility criteria to the protocol, to clarify eligibility criteria for subjects enrolling in Part 1b of the study, and to provide additional information about blood volumes for biomarker assessments.

Amendment 3 (31-Jul-2023)

This amendment is considered to be substantial based on the criteria set forth in Article 10(a) of Directive 2001/20/EC of the European Parliament and the Council of the European Union.

This modification is considered to be substantial based on the criteria set forth in European Regulation 536/2014 Article 2(2)(13).

Overall Rationale for the Amendment:

The purpose of this version is to add text for a Japan only cohort during Part 1 and other minor editorial changes for consistency throughout the protocol by updating the following:

- Added information about the Japan only cohort (Part 1b, Monotherapy Dose Escalation for Japan) to the protocol (Protocol Section 1.1)
- Added figure for the Japan only cohort (Protocol Section 1.2, Figure 1–2)
- Modified schema to include the Japan only cohort (Protocol Section 1.3, Figure 1-3)
- Added requirements and footnotes for the Japan only cohort to the Schedule of Activities (Protocol Section 1.3)
- Split Part 1 study design into Global (Part 1a) and Japan-specific (Part 1b) parts (Protocol Section 4.1.1, Protocol Section 4.1.1.1 [new], and Protocol Section 4.1.1.2 [new])
- Added text to the dose escalation guidelines for the Japan only cohort (Protocol Section 4.1.5)
- Updated subject population to include the Japan only cohort (Protocol Section 4.2.2)
- Added section for the Japan only cohort to the subject history section of the eligibility criteria to indicate that subjects in the Japan only cohort must not have any history of ILD (Protocol Section 5.1)
- Added clarifying statement for combination therapies meeting DLT criteria (Protocol Section 8.2)

- Added sections for the Japan only cohort for safety assessments (Protocol Section 8.4, Protocol Section 8.4.1, and Protocol Section 8.4.5.1)
- Added section for the Japan only cohort to indicate that biomarker research results will not be disclosed to subjects in the Japan only cohort (Protocol Section 8.9)
- Clarified the treatment-emergent period (Protocol Section 9.3.3)
- Updated the total number of subjects in the study to include the subjects from the Japan only cohort (Protocol Section 9.6)
- Added KL-6 and Hemoglobin A1C (HbA1C) testing requirements and virology testing only for the Japan only cohort (Protocol Section 10.2, Table 10-1)
- Added list of contraception methods that are not approved or certified in Japan (Protocol Section 10.4)
- Added supplemental information about Japan-specific requirements for scientific findings on drugs, handling of drugs used in the clinical trial, and reporting of serious adverse event and adverse event of special interest (Protocol Section 10.11.1)

Amendment 2 (15-Jun-2023)

This amendment is considered to be substantial based on the criteria set forth in Article 10(a) of Directive 2001/20/EC of the European Parliament and the Council of the EU.

This modification is considered to be substantial based on the criteria set forth in European Regulation 536/2014 Article 2(2)(13).

Overall Rationale for the Amendment:

The purpose of this version is to add text aligned with the EU CTR guidelines, to implement changes described in response commitment letter to the Korean Health Authority, Ministry of food and drug safety by updating the following:

- Removed pre-screening for SEZ6 (if elected by Sponsor) and information related to SEZ6 option of implementing prospective selection (Protocol Section 1.3, Figure 1–2, Protocol Section 4.1.1, Protocol Section 4.1.2, Protocol Section 5.1, Protocol Section 5.4, Protocol Section 6.3, Protocol Section 8.1.1, Protocol Section 8.1.7, Protocol Section 8.5.1, Protocol Section 8.9, and Protocol Section 9.6)
- Added "ABBV-706 in combination with carboplatin and ABBV-706 in combination with cisplatin and will proceed independently of each other." (Protocol Section 4.1.3.2)

- Deleted "The secondary escalation in subjects with CNS tumors can be opened at least one dose level below the primary escalation MTD or the highest dose that has met BOIN escalation criteria with at least 3 DLT evaluable subjects (e.g., 0 out of 3 DLTs) and will then proceed independent of the primary escalation." (Protocol Section 4.1.7)
- Added definition for participant's study completion. (Protocol Section 4.4)
- Added the following text "(or according to local label for carboplatin or cisplatin, whichever is longer, for Part 3b carboplatin or cisplatin combination subjects; Korea only: through at least 11 or 14 months after the last dose of cisplatin or carboplatin)." Added "For subjects with liver metastases: ALT and AST ≤ 5.0 × ULN". Restructured eligibility criteria 11 and 12. (Protocol Section 5.1)
- Added IMP strength and updated condition of study drug administration "Platelets ≥ 75 × 10^9/L (no transfusions within 5 days prior to dose)". (Protocol Section 6.1 and Table 6-1)
- Added for Part 3a combination, "dose reductions for carboplatin and cisplatin based on renal function should follow local/institutional guidelines." (Protocol Section 6.7)
- Deleted "In situations where an IRR occurs prior to the end of infusion, ABBV-706 infusion should be immediately interrupted." Changed reflexive testing following IRRs from required to recommended. (Protocol Section 6.7.4)
- Updated timeline for review of prior and concomitant medication to start from "signature of consent through 80 days (for Parts 1, 2, 3b, and 4) or 90 days (for Part 3a) after last dose of study drug." (Protocol Section 6.10)
- Added time window for vital sign assessment "(±10 minutes)" after the start of infusion. (Protocol Section 8.4.3)
- Amended text on PK blood sample collection and updated Sampling Schedule for End of Treatment of ABBV-706 PK and ADA Assessments (Protocol Section 8.6.1)
- Added "AbbVie will be responsible for ensuring all biological sample collection and storage is secure and with adequate measures to protect confidentiality, per all applicable local regulations. AbbVie will store biological samples related to plasma, serum, or tumor tissue samples collected as part of the PK/PD/biomarker/immunogenicity assessments. With participants' consent, samples may be used for further research by AbbVie, per all applicable local regulations." (Protocol Section 8.11)
- Added text "Events of disease progression related to the primary malignancy being evaluated in this study and accompanying symptoms, if clearly associated with disease progression, will not be collected as adverse events unless the event(s) result in death or qualifies as a serious adverse event as defined in Section 10.3.2 and require reporting in the same manner as any serious adverse event." (Protocol Section 10.3.1)

- Modified text "All AEs and SAEs reported from the time of study drug administration until 80 days (5 half-lives of ABBV-706) after discontinuation last dose of study drug administration (for Parts 1, 2, 3b, and 4) or 90 days after last dose of study drug (Part 3a) will be collected, whether solicited or spontaneously reported by the subject. In addition, study procedure-related serious and nonserious AEs will be collected from the time the subject signs the study-specific informed consent. until study drug administration. After 80 days (for Parts 1, 2, 3b, and 4) or 90 days (for Part 3a) following completion of study treatment and throughout the post-treatment period, all spontaneously reported SAEs will be collected (nonserious AEs will not be collected)." Added "Suspected unexpected serious adverse reactions reporting will be in adherence to requirements of EU pharmacovigilance legislation, clinical trial legislation and guidance. EU CTR 536/2014; CT-3 and all other applicable local regulations." (Protocol Section 10.3.2)
- Deleted the following "The following "binary" decision choice will be used by the investigator to describe the initial causality assessment: (Protocol Section 10.3.3)
  - Related: Reasonable possibility of a relatedness.
  - Not Related: No reasonable possibility of relatedness"
- Updated reporting instruction and hotline details. (Protocol Section 10.3.4)
- Added underlined text "Contraception recommendations related of use of carboplatin or cisplatin in Part 3b or concomitant therapies prescribed should be based on the local label and contraception period should be based on whichever is longer considering all administered therapies. In Korea, for subjects treated with the cisplatin or carboplatin combination, female subjects of childbearing potential must practice an effective method of birth control during the treatment period of cisplatin or carboplatin and 14 months after the last dose of cisplatin or carboplatin; male subjects must practice an effective method of birth control during the treatment period of cisplatin or carboplatin and 11 months after the last dose of cisplatin or carboplatin." (Protocol Section 10.4)

Amendment 1 (07-October-2022)

This amendment is considered to be substantial based on the criteria set forth in Article 10(a) of Directive 2001/20/EC of the European Parliament and the Council of the EU.

This modification is considered to be substantial based on the criteria set forth in European Regulation 536/2014 Article 2(2)(13).

Overall Rationale for the Amendment:

The purpose of this version is to correct minor clerical errors for consistency throughout the protocol in addition to addressing the Food and drug administrations (FDA's) requests by updating the following:

- Moved the evaluation of clinical benefit, stable disease, progression-free survival, and overall survival from primary to exploratory objectives for subjects with R/R SCLC, high-grade CNS tumors and NECs (Protocol Section 1.1 and Protocol Section 3).
- Clarified that the safety Monitoring Committee will also make starting dose and dose escalation recommendations for Parts 3a and 3b (Protocol Section 1.2 and Protocol Section 4.1.6).
- Clarified in Part 1 that subjects with high-grade CNS must have had prior surgical resection, received prior radiation, and temozolomide or PCV and subjects with NECs must have also received at least one prior line of standard of care systemic chemotherapy (Protocol Section 1.3 and Protocol Section 5.1, Eligible Criterion #11).
- Clarified in Part 3a and Part 3b that subjects with R/R and other high-grade poorly differentiate NECs must have progressed on at least one line of standard of care systemic chemotherapy (Protocol Section 1.3 and Protocol Section 5.1, Eligible Criteria #13 and #14, respectively).
- Clarified in Part 4a that subjects with R/R high-grade CNS must have had prior surgical resection, received prior radiation, and temozolomide or PCV (Protocol Section 1.3 and Protocol Section 5.1, Eligible Criterion #15).
- Clarified in Part 4b that subjects with R/R NECs and other high-grade poorly differentiated NECs must have had prior line of standard of care systemic chemotherapy (Protocol Section 1.3 and Protocol Section 5.1, Eligible Criterion #16).
- Removed duplicate Eligible Criteria #17 through #20 and renumbered remaining Eligible Criteria accordingly (Protocol Section 5.1, Eligible Criterion #16).
- Added cautionary use of strong inducers of cytochrome P450 to allowed medications (Protocol Section 5.1, Eligible Criterion #18; Protocol Section 6.11.1; Protocol Section 10.6, Appendix 6).
- Clarified the ABBV-706 toxicity management of Grade 3 and Grade 4 non-hematologic toxicities (Protocol Section 6.7).
- Updated definitions of dose-limiting toxicities (Protocol Section 8.2).

- Changed the AE and SAE collection period for Part 3a to 90 days (Protocol Section 8.5.1, Protocol Section 1.5, Table 1.1 footnote 'g' and Table 1.2 footnote 'i').
- Added that high risk biopsies near any vital neurovascular structures should be avoided (Protocol Section 8.9).
- Added that budigalimab should be permanently discontinued for ≥ Grade 2 myocarditis and ≥ Grade 3 ocular toxicity (Protocol Section 10.8.1).
- Added that for any grade transverse myelitis and for ≥Grade 2 Guillain-Barre Syndrome, study treatment should be discontinued (Protocol Section 10.8.12).
- Added Day 2 to Cycle 2 to collected AEs, concomitant therapy, triplicate ECG, vital signs, and PK samples (Protocol Section 1.5, Table 1.1 and Table 1.2).
- Clarified that neurological exams are to be done whenever a physical exam is done (Protocol Section 1.5, Table 1.1 and Table 1.2).
- Added collection timepoints for PK and triplicate ECGs on Days 1 and 2 of Cycle 2 (Parts 1 and 2), PK on Days 1, 2 and 8 of Cycle 1 (Parts 3 and 4) and clarified collection for budigalimab ADA/nAb assessments (Part 3a) (Section 8.1, Table 8.1).

10.13 Appendix 13: Investigator Agreement

Protocol M23-385: A Phase 1 first-in-human study evaluating safety, pharmacokinetics and efficacy of ABBV-706 as monotherapy and in combination with budigalimab, carboplatin, or cisplatin in adult subjects with advanced solid tumors

Protocol Date: 13 February 2026

Declaration

All documentation for this study that is supplied to me and that has not been previously published will be kept in the strictest confidence. This documentation includes this study protocol, Investigator's Brochure, eCRFs, and other scientific data.

The study will not be commenced without the prior written approval of a properly constituted IRB or IEC. No changes will be made to the study protocol without the prior written approval of the Sponsor and the IRB or IEC, except where necessary to eliminate an immediate hazard to the subjects.

I have read and understood and agree to abide by all the conditions and instructions contained in this protocol.

Responsibilities

Clinical research studies sponsored by AbbVie are subject to the International Council for Harmonization of Technical Requirements for Pharmaceuticals for Human Use (ICH) Good Clinical Practices (GCP) and local regulations and guidelines governing the study at the site location. In signing the Investigator Agreement, the investigator is agreeing to the following:

1. Conducting the study in accordance with ICH GCP, the applicable regulatory requirements and current protocol, and making changes to a protocol only after notifying AbbVie and the appropriate Institutional Review Board (IRB)/Independent Ethics Committee (IEC), except when necessary to protect the subject from immediate harm.
2. Personally conducting or supervising the described investigation(s).
3. Informing all subjects, or persons used as controls, that the drugs are being used for investigational purposes and complying with the requirements relating to informed consent and ethics committees (e.g., IEC or IRB) review and approval of the protocol and its amendments.
4. Reporting complaints that occur in the course of the investigation(s) to the Sponsor.
5. Reading the information in the Investigator's Brochure/safety material provided, including the instructions for use and the potential risks and side effects of the investigational product(s).
6. Informing all associates, colleagues, and employees assisting in the conduct of the study about their obligations in meeting the above commitments.
7. Maintaining adequate and accurate records of the conduct of the study, making those records available for inspection by representatives of the Sponsor and/or the appropriate

regulatory agency, and retaining all study-related documents until notification from the Sponsor.

8. Maintaining records demonstrating that an ethics committee reviewed and approved the initial clinical protocol and all of its amendments.
9. Reporting promptly, within 1 calendar day to the Sponsor, the ethics committees/institutional review boards (as required) and other appropriate individuals (e.g., coordinating investigator, institution director):
   - All changes in the research activity and all unanticipated problems involving risks to human subjects or others,
   - Any departure from relevant clinical trial law or regulation, GCP, or the trial protocol that has the potential to affect the following:
     - Rights, safety, physical or mental integrity of the subjects in the clinical trial
     - Scientific value of the clinical trial, reliability or robustness of data generated
10. Providing direct access to source data documents for study-related monitoring, audits, IEC/IRB review, and regulatory inspection(s).

Responsible Investigator of the Local Study Center

Signature | Date

Name (block letters)

Title (block letters)

Institution (block letters)

Phone number

11 References

1. Gauzy-Lazo L, Sassoon I, Brun MP. Advances in Antibody-Drug Conjugate Design: Current Clinical Landscape and Future Innovations. SLAS Discov. 2020 Sep;25(8):843-868.

2. Wiedemeyer WR, Gavrilyuk J, Schammel A, et al. ABBV-011, A Novel, Calicheamicin-Based Antibody-Drug Conjugate, Targets SEZ6 to Eradicate Small Cell Lung Cancer Tumors. Mol Cancer Ther. 2022 Jun 1;21(6):986-998.

3. McKenzie JA, Mbofung RM, Malu S, et al. The effect of topoisomerase I inhibitors on the efficacy of T-cell-based cancer immunotherapy. J Natl Cancer Inst. 2018 Jul 1;110(7):777-86.

4. Iwata TN, Ishii C, Ishida S, et al. A HER2-targeting antibody drug conjugate, trastuzumab deruxtecan (DS-8201a), enhances antitumor immunity in a mouse model. Mol Cancer Ther. 2018 Jul; 17(7):1494–503.

5. Calvo E, Spira A, de Miguel M, et al. Safety, pharmacokinetics, and efficacy of budigalimab with rovalpituzumab tesirine in patients with small cell lung cancer. Cancer Treat Res Commun. 2021; 28:100405.

6. ABBV-706 Investigator's Brochure

7. Powderly J, Spira A, Kondo S, et al. Model informed dosing regimen and Phase 1 safety results of the anti-PD-1 antibody budigalimab (ABBV-181). Clin Transl Sci. 2021 Jan;14(1):277-87.

8. Budigalimab (ABBV-181) Investigator's Brochure

9. Yuan Y, Hess KR, Hilsenbeck SG, Gilbert MR. Bayesian Optimal Interval Design: A Simple and Well-Performing Design for Phase I Oncology Trials. Clin Cancer Res. 2016 Sep 1;22(17):4291-301.

10. Food and Drug Administration. Expansion Cohorts: Use in First-in-Human Clinical Trials to Expedite Development of Oncology Drugs and Biologics - Guidance for Industry. March 2022. Office of Oncologic Diseases in the Center for Drug Evaluation and Research. Available at https://www.fda.gov/media/115172/download.

11. Brahmer JR, Lacchetti C, Schneider BJ, et al. Management of Immune-Related Adverse Events in Patients Treated With Immune Checkpoint Inhibitor Therapy: American Society of Clinical Oncology Clinical Practice Guideline. J Clin Oncol. 2018;36(17):1714-68.

12. Haanen JBAG, Carbonnel F, Robert C, et al. Management of toxicities from immunotherapy: ESMO Clinical Practice Guidelines for diagnosis, treatment and follow-up. Ann Oncol. 2018;29(Suppl 4):iv264-iv6.

13. Opdivo (nivolumab) [summary of product characteristics]. Uxbridgem United Kingdom; Bristol-Myers Squibb Pharma EEIG, 2015.

14. OPDIVO (nivolumab) [prescribing information]. Princeton, New Jersey; Bristol-Myers Squibb Co., 2016.

15. Keytruda (pembrolizumab) [summary of product characteristics]. Hoddesdon, United Kingdom; Merck Sharp Dohme Ltd., 2015.

16. Hausheer FH, Kanter P, Cao S, et al. Modulation of platinum-induced toxicities and therapeutic index: mechanistic insights and first- and second-generation protecting agents. Semin Oncol. 1998 Oct;25(5):584-99.

17. Eisenhauer EA, Therasse P, Bogaerts J, et al. New response evaluation criteria in solid tumours: revised RECIST guideline (version 1.1). Eur J Cancer. 2009;45(2):228-47.

18. Wen PY, Macdonald DR, Reardon DA, et al. Updated response assessment criteria for high-grade gliomas: response assessment in Neuro-Oncology Working Group. J Clin Oncol. 2010;28(11):1963-72.

19. Ellingson BM, Wen PY, Cloughesy TF. Modified criteria for radiographic response assessment in glioblastoma clinical trials. Neurotherapeutics. 2017;14(2):307-20.

20. Clopper CJ, Pearson ES. The Use of Confidence or Fiducial Limits Illustrated in the Case of the Binomial. Biometrika. 1934;26(4):404-13.

21. Mokdad A, Xie X, Zhu H, et al. Statistical Justification of Expansion Cohorts in Phase 1 Cancer Trials. Cancer 2018; 124(16):3339-3345.

22. Brahmer JR, Lacchetti C, Schneider BJ, et al. Management of immune-related adverse events in patients treated with immune checkpoint inhibitor therapy: American Society of Clinical Oncology Clinical Practice Guideline. J Clin Oncol. 2018;36(17):1714-68.

23. Haanen J, Carbonnel F, Robert C, et al. Management of toxicities from immunotherapy: ESMO Clinical Practice Guidelines for diagnosis, treatment and follow-up. Ann Oncol. 2017;28(suppl_4):iv119-iv142.

24. National Comprehensive Cancer Network. NCCN Clinical Practice Guidelines in Oncology: Management of Immune-Related Toxicities, Version 1.2018. Plymouth Meeting, PA: NCCN; 2018.

25. Puzanov I, Diab A, Abdallah K, et al. Managing toxicities associated with immune checkpoint inhibitors: consensus recommendations from the Society for Immunotherapy of Cancer (SITC) Toxicity Management Working Group. J Immunother Cancer. 2017;5(1):95.
