# Supplementary material for: SEZ6-targeting antibody−drug conjugate ABBV-706 in advanced small cell lung cancer and solid tumors: a phase 1 trial
Source: Nat Med. 2026 Jun 1;32(8):2898–908. doi: 10.1038/s41591-026-04452-0 (PMC13472869; doi:10.1038/s41591-026-04452-0)
Supplement: Supplementary file 2 [file 41591_2026_4452_MOESM2_ESM.pdf]

nature portfolio

Corresponding author(s): Lauren Byers

Last updated by author(s): May 6, 2026

Reporting Summary

Nature Portfolio wishes to improve the reproducibility of the work that we publish. This form provides structure for consistency and transparency in reporting. For further information on Nature Portfolio policies, see our Editorial Policies and the Editorial Policy Checklist.

Statistics

For all statistical analyses, confirm that the following items are present in the figure legend, table legend, main text, or Methods section.

n/a	Confirmed	
☐	☒	The exact sample size (*n*) for each experimental group/condition, given as a discrete number and unit of measurement
☐	☒	A statement on whether measurements were taken from distinct samples or whether the same sample was measured repeatedly
☐	☒	The statistical test(s) used AND whether they are one- or two-sided<br>*Only common tests should be described solely by name; describe more complex techniques in the Methods section.*
☒	☐	A description of all covariates tested
☒	☐	A description of any assumptions or corrections, such as tests of normality and adjustment for multiple comparisons
☐	☒	A full description of the statistical parameters including central tendency (e.g. means) or other basic estimates (e.g. regression coefficient) AND variation (e.g. standard deviation) or associated estimates of uncertainty (e.g. confidence intervals)
☒	☐	For null hypothesis testing, the test statistic (e.g. *F*, *t*, *r*) with confidence intervals, effect sizes, degrees of freedom and *P* value noted<br>*Give P values as exact values whenever suitable.*
☒	☐	For Bayesian analysis, information on the choice of priors and Markov chain Monte Carlo settings
☒	☐	For hierarchical and complex designs, identification of the appropriate level for tests and full reporting of outcomes
☒	☐	Estimates of effect sizes (e.g. Cohen's *d*, Pearson's *r*), indicating how they were calculated

Our web collection on statistics for biologists contains articles on many of the points above.

Software and code

Policy information about availability of computer code

Data collection	Data are collected within the electronic case report form within the sponsor's EDC system. All data generated by the site personnel will be captured electronically at each study center using eCRFs. Data from external sources (such as central laboratory data) will be imported into the database. The investigator is responsible for verifying that data entries are accurate and correct. Once the eCRF clinical data have been submitted to the central server at the independent data center, corrections to the data fields will be captured in an audit trail. The reason for change, the name of the person who performed the change, together with the time and date will be logged to provide an audit trail. The Sponsor or designee will perform monitoring to confirm that data entered into the eCRF by authorized site personnel are accurate, complete, and verifiable from source documents; that the safety and rights of subjects are being protected; and that the study is being conducted in accordance with the currently approved protocol and any other study agreements, ICH GCP, and all applicable regulatory requirements.<br>For PK, immunogenicity, and biomarker assessments - blood, plasma, serum and tissue samples were collected at pre-specified timepoints.
Data analysis	Data analysis used standard tools as outlined in the methods section. All analyses were performed using SAS Version 9.4 (SAS Institute Inc., Cary, NC) or later.

For manuscripts utilizing custom algorithms or software that are central to the research but not yet described in published literature, software must be made available to editors and reviewers. We strongly encourage code deposition in a community repository (e.g. GitHub). See the Nature Portfolio guidelines for submitting code & software for further information.

Data

Policy information about availability of data

All manuscripts must include a data availability statement. This statement should provide the following information, where applicable:

- Accession codes, unique identifiers, or web links for publicly available datasets
- A description of any restrictions on data availability
- For clinical datasets or third party data, please ensure that the statement adheres to our policy

AbbVie is committed to responsible data sharing regarding the clinical trials we sponsor. This includes access to anonymized, individual, and trial-level data (analysis data sets), as well as other information (eg, protocols, clinical study reports, or analysis plans), as long as the trials are not part of an ongoing or planned regulatory submission. This includes requests for clinical trial data for unlicensed products and indications.
These clinical trial data can be requested by any qualified researchers who engage in rigorous, independent, scientific research, and will be provided following review and approval of a research proposal, Statistical Analysis Plan (SAP), and execution of a Data Sharing Agreement (DSA). Data requests can be submitted at any time, with acknowledgement of the request within two weeks. The data will be accessible for 12 months, with possible extensions considered. For more information on the process or to submit a request, visit the following link: https://vivli.org/ourmember/abbvie/
then select "Home."

This study does not include any publicly available datasets.

Research involving human participants, their data, or biological material

Policy information about studies with human participants or human data. See also policy information about sex, gender (identity/presentation), and sexual orientation and race, ethnicity and racism.

Reporting on sex and gender	Our manuscript does not specifically report on the collection of patient sex or gender as no analyses, including efficacy or safety, were stratified by these characteristics given the safety and efficacy focused, first-in-human nature of the phase 1 trial. Results are reported for overall population and by dose cohorts where applicable; no formal comparisons by sex were made or specified in the protocol. Sex (male or female) was patient-reported and collected as part of baseline demographics.
Reporting on race, ethnicity, or other socially relevant groupings	The study did not include pre-specified analyses of safety or efficacy by race, ethnicity, or any other socio-demographics. Data on participant race was collected as part of the baseline demographic data. Patients were primarily White (64%) or Asian (28%).
Population characteristics	Across all R/R SCLC cohorts, median age was 64 (range 35–85), 59% were male, 81% had ECOG PS of 1, and 34% had brain metastasis at study entry. Among the 1.8 mg/kg and 2.5 mg/kg cohorts, 39% and 31% had brain metastasis at study entry, respectively. Median number of prior lines of therapies was 2 (range 1–6) for both 1.8 mg/kg and 2.5 mg/kg cohorts. For 1.8 mg/kg and 2.5 mg/kg cohorts, 59% and 64% had two or more prior lines; 61% and 44% of patients had platinum-resistant disease (<90 days chemotherapy-free interval [CTFI]), 27% and 21% were platinum refractory (<30 days CTFI); and 29% and 28% had previously received treatment with a Top1i, respectively.
Recruitment	Recruitment was performed locally at the site level for all regions, and driven by staff and investigators with experience in treating patients with advanced solid tumors and trained on the clinical trial protocol. Patients provided informed consent.
Ethics oversight	The study was conducted in accordance with the principles of the Declaration of Helsinki and Good Clinical Practice Guidelines of the International Conference on Harmonization and was approved by the regulatory and independent ethics committees/institutional review boards at each site. All patients provided written informed consent. The central IRB as Advarra and each site either used the local or central IRB for protocol approval (full listing provided in the Supplementary Information Table S3).

Note that full information on the approval of the study protocol must also be provided in the manuscript.

Field-specific reporting

Please select the one below that is the best fit for your research. If you are not sure, read the appropriate sections before making your selection.

☒ Life sciences ☐ Behavioural & social sciences ☐ Ecological, evolutionary & environmental sciences

For a reference copy of the document with all sections, see nature.com/documents/nr-reporting-summary-flat.pdf

Life sciences study design

All studies must disclose on these points even when the disclosure is negative.

Sample size	All analyses are descriptive as no formal hypothesis testing was planned and no formal sample size calculations were performed. Sample size, while not formally calculated was estimated based on the lower bound of a one-sided 80% CI for ORR that would be sufficient for providing a preliminary assessment of efficacy, and was estimated at approximately 40; in total the number of patients expected to enroll across all study parts was 317.

Data exclusions	Patient exclusion criteria was pre-specified in the protocol. Key exclusion criteria included prior treatment with an ADC containing a Top1i payload for all parts, and prior treatment with a SEZ6-targeted ADC in part 2.
Replication	This was a first in human clinical study and findings were not replicated.
Randomization	This was a phase 1, open label, nonrandomized study overall. In part 2a, patients with R/R SCLC were randomized evenly to receive ABBV-706 1.8 mg/kg or 2.5 mg/kg Q3W to determine the recommended phase 2 dose (RP2D); no stratification factors were applied.
Blinding	As this was an open-label, dose-escalation and dose-expansion study, blinding was not applicable.

Reporting for specific materials, systems and methods

We require information from authors about some types of materials, experimental systems and methods used in many studies. Here, indicate whether each material, system or method listed is relevant to your study. If you are not sure if a list item applies to your research, read the appropriate section before selecting a response.

Materials & experimental systems

n/a	Involved in the study
☒	☐ Antibodies
☒	☐ Eukaryotic cell lines
☒	☐ Palaeontology and archaeology
☒	☐ Animals and other organisms
☐	☒ Clinical data
☒	☐ Dual use research of concern
☒	☐ Plants

Methods

n/a	Involved in the study
☒	☐ ChIP-seq
☒	☐ Flow cytometry
☒	☐ MRI-based neuroimaging

Clinical data

Policy information about clinical studies

All manuscripts should comply with the ICMJE guidelines for publication of clinical research and a completed CONSORT checklist must be included with all submissions.

Clinical trial registration	Clinicaltrials.gov identifier: NCT05599984
Study protocol	Final study protocol is available in the supplementary materials.
Data collection	Clinical data was collected at local sites (active sites are available on clincialtrials.gov) and reported in the eCRFs. There were 66 locations. The first patient enrollment date was December 28, 2022 (part 1) and last patient enrollment date was August 29, 2025 (part 2c).
Outcomes	The primary objectives of parts 1, 2, and 4 of the study were to evaluate the safety, tolerability, PK, immunogenicity, and antitumor activity of ABBV-706 monotherapy, and from parts 1 and 2 to determine the RP2D of ABBV-706 in R/R SCLC. Adverse events (AEs) were summarized using Medical Dictionary for Regulatory Activities preferred terms and severity was graded per National Cancer Institute Common Terminology Criteria for Adverse Events v5.0, except for interstitial lung disease (ILD)/pneumonitis, for which the American Society of Clinical Oncology grading criteria were used (11). In addition, a central ILD adjudication committee retrospectively reviewed suspected cases of ILD. The primary efficacy outcome was investigator-assessed ORR (complete response [CR] or partial response [PR]) per RECIST v1.1. Point estimates along with two-sided 90% confidence intervals (CIs) for ORR were calculated using the Clopper-Pearson (exact) method. Patients who had not experienced disease progression or died were censored at the last disease assessment prior to the data cutoff date or start of any subsequent anticancer therapy, whichever occurred earlier. PK endpoints included maximum observed serum (or plasma for payload) concentration (Cmax), time to reach Cmax (Tmax), terminal phase elimination half-life, and area under the serum (or plasma for payload) concentration vs time curve (AUC) for ABBV-706 conjugate, total antibody, and unconjugated (free) Top1i payload. PK parameters were determined using non-compartmental methods. Immunogenicity assessments for ABBV-706 anti-drug antibodies (ADA) were evaluated across multiple timepoints.

Plants

Seed stocks	Not applicable.
Novel plant genotypes	Not applicable.
Authentication	Not applicable.
